# Metavisitor, a Suite of Galaxy Tools for Simple and Rapid Detection and Discovery of Viruses in Deep Sequence Data

**DOI:** 10.1371/journal.pone.0168397

**Published:** 2017-01-03

**Authors:** Guillaume Carissimo, Marius van den Beek, Kenneth D. Vernick, Christophe Antoniewski

**Affiliations:** 1 Institut Pasteur, Unit of Insect Vector Genetics and Genomics, Department of Parasites and Insect Vectors, Paris, FRANCE; 2 CNRS, Unit of Hosts, Vectors and Pathogens (URA3012), Paris, FRANCE; 3 Laboratory of Microbial Immunity, Singapore Immunology Network, A*STAR, 8A Biomedical Grove, Biopolis, Singapore, Singapore; 4 Sorbonne Universités, Université Pierre et Marie Curie (UPMC), CNRS, Institut de Biologie Paris Seine (IBPS), Developmental Biology Department, Paris, France; 5 Sorbonne Universités, Université Pierre et Marie Curie (UPMC), CNRS, Institut de Biologie Paris Seine (IBPS), ARTbio Bioinformatics Analysis Facility, Paris, France; 6 Department of Microbiology, University of Minnesota, Minneapolis, MN, United States of America; Institut de Biologie Moleculaire et Cellulaire, FRANCE

## Abstract

Metavisitor is a software package that allows biologists and clinicians without specialized bioinformatics expertise to detect and assemble viral genomes from deep sequence datasets. The package is composed of a set of modular bioinformatic tools and workflows that are implemented in the Galaxy framework. Using the graphical Galaxy workflow editor, users with minimal computational skills can use existing Metavisitor workflows or adapt them to suit specific needs by adding or modifying analysis modules. Metavisitor works with DNA, RNA or small RNA sequencing data over a range of read lengths and can use a combination of *de novo* and guided approaches to assemble genomes from sequencing reads. We show that the software has the potential for quick diagnosis as well as discovery of viruses from a vast array of organisms. Importantly, we provide here executable Metavisitor use cases, which increase the accessibility and transparency of the software, ultimately enabling biologists or clinicians to focus on biological or medical questions.

## Introduction

Viruses infect cells and manipulate the host machinery for their replication and transmission. Genomes of viruses show high diversity and can consist of single- or double-stranded RNA or DNA. Many types of viral replication cycles exist which may involve various cellular compartments, various DNA or RNA replication intermediates, and diverse strategies for viral RNA transcription and viral protein translation. Thus, deep-sequencing has become a powerful approach for virologists in their quest to detect and identify viruses in biological samples, even when they are present at low levels. Plants and invertebrates use RNA interference as an antiviral mechanism [1,2]. Antiviral RNAi activity results in accumulation of viral interfering small RNAs (viRNAs), whose extent depends on several factors such as the ability of a virus to replicate in the host and to evade the host RNAi machinery. Moreover, viRNAs derived from a variety of viruses can be detected in host organisms, regardless if these viruses have positive single strand, negative single strand or double-stranded RNA genomes, or DNA genomes [2]. Together, these features make small RNA deep sequencing a potent approach to detect viruses regardless of their genomic specificities, and different bioinformatic tools have been developed for detection or *de novo* assembly of viral genomes.

Accordingly, viRNAs produced by the insect model *Drosophila melanogaster* in response to viral infections were sufficient to reconstruct and improve the genomic consensus sequence of the Nora virus [3] using the Paparazzi software [4] which is based on the SSAKE assembler [5]. In that study, Paparazzi improved the consensus sequence and the coverage of the Nora virus genome by ~20%, as compared to the previous Nora virus reference genome. SearchSmallRNA, a standalone tool with a graphical interface, used a similar approach to reconstruct viral genomes [6]. Importantly, both programs require known, closely related viral references for proper guidance of genome reconstructions from viRNAs, precluding the identification of more distant viral species or discovery of novel or unexpected viruses.

To circumvent the need for viral reference sequences, Velvet [7] *de novo* assembled contigs from plant [8], fruit fly and mosquito [9] have been aligned to NCBI sequence databases, allowing the identification of partial or complete viral genomes. Several studies improved this strategy by combining two *de novo* assemblers [10–13], or scaffolding virus-aligned contigs using an additional translation-guided assembly step [14].

Collectively, these studies allowed important progress in virus assembly and identification from deep sequencing data. However, the existing computational workflows require specialist skills for installation, execution and adaptation to specific research, making them poorly accessible to a broad user base of biologists. In some cases, the tools lack documentation or their source codes are not available.

In this context, we developed Metavisitor as an open source set of tools and preset workflows [15,16] which allow effective implementation of the computational strategies in the Galaxy framework, with short read as well as long read sequence datasets. In addition, Metavisitor workflows can be easily adapted to suit specific needs, by adding analysis steps or replacing/modifying existing ones with the numerous tools available in the Galaxy tool sheds. Here, we report a series of use cases of Metavisitor and we show that it provides biologists and medical practitioners with an easy-to-use and adaptable software for the detection or identification of viruses from high-throughput sequence datasets.

## Experimental Procedures

**Fig 1:**
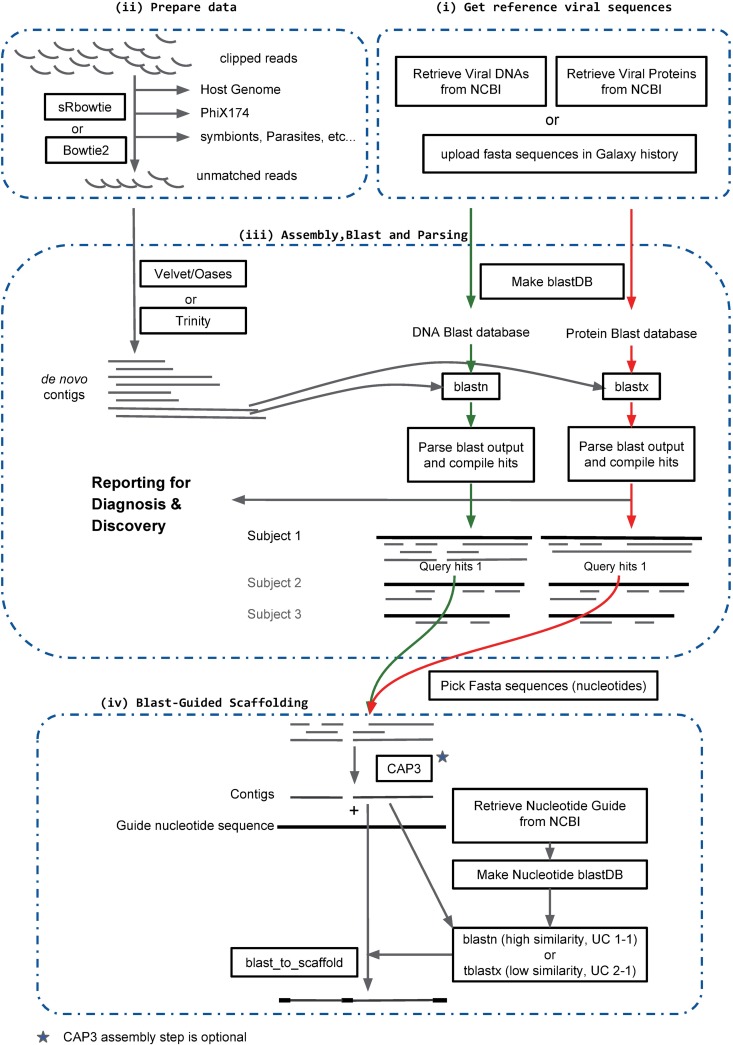
Global view of the Metavisitor workflow. The workflow is organised in sub workflows (dashed line) corresponding to functional tasks as described in the manuscript. All Galaxy Tools (square boxes) are available in the main Galaxy tool shed (https://toolshed.g2.bx.psu.edu/).

Metavisitor consists of a set of Galaxy tools (Fig 1) that can be combined to (i) retrieve up-to-date nucleotide as well as protein sequences of viral genomes deposited in Genbank [17] and index these sequences for subsequent alignments; (ii) extract sequencing reads that do not align to the host genomes, known symbionts or parasites; (iii) perform *de novo* assembly of these reads using assembly tools available in Galaxy, align the *de novo* contigs against the viral nucleotide or protein blast databases using blastn or blastx, respectively, and generate reports from blast outputs to help in known viruses diagnosis or in candidate virus discovery; (iv) use CAP3 (optional, see Use Case 3–3), blast and viral scaffolds for selected viruses to generate guided final viral sequence assemblies of blast sequence hits. Below, we group analysis steps in functional tasks i to iv and provide details on the Metavisitor tools. These tasks are linked together to build full workflows adapted to the analysis of the use cases described in the result section.

### (i) Get reference viral sequences

The “Get reference viral sequences” task is performed using the “Retrieve FASTA from NCBI” tool that sends a query string to the Genbank database [17] and retrieves the corresponding nucleotide or protein sequences. For the viral nucleotide and protein sequences referred to as “vir1”, we used the tool to query Genbank (oct 2015) and retrieve viruses sequences filtered out from cellular organisms and bacteriophage sequences (see S1 Fig). However, users can change the tool settings by entering query strings that fit their specific needs. As retrieving vir1 from NCBI takes several hours, we allow users to skip the step by directly accessing the nucleotides or protein vir1 datasets on the Mississippi server (http://mississippi.fr) or to download them from figshare (https://dx.doi.org/10.6084/m9.figshare.3179026). For convenience, nucleotide and protein blast indexes of vir1 are also available in the public library of the Mississippi server,but can also be generated using the “NCBI BLAST+ makeblastdb” Galaxy tool [18]. Bowtie [19] as well as bowtie2 [20] indexes of the vir1 nucleotide sequences have been generated in the Mississippi Galaxy instance using the corresponding “data manager” Galaxy tools.

Finally, users can upload their own viral nucleotide and protein sequences using ftp and transfer them to a Galaxy history (Fig 1), where they can use the Galaxy data manager tools to produce the blast and bowtie indexes necessary for Metavisitor.

### (ii) Prepare data

The “Prepare data” task (Fig 1) processes Illumina sequencing datasets in order to optimize the subsequent *de novo* assembly of viral sequencing reads. Fastq files of sequence reads are first clipped from library adapters and converted to fasta format using our tool “Clip adapter” tool (S1 Table). The clipped reads may be further converted to a fasta file of unique sequences headed by a character string that contains a unique identifier and the number of times that the sequences were found in the dataset, thus reducing the size of the dataset without loss of information. This optional treatment removes sequence duplicates and drastically reduces the workload of the next steps as well as the coverage variations after *de novo* assembly (see Use Cases 1–1 to 1–3). Clipped reads are then depleted from non-viral sequences by sequential alignments to the host genome, to other genomes from known or potential symbionts and parasites, as well as to PhiX174 genome sequences which are commonly used as internal controls in Illumina sequencing and may contaminate the datasets (Fig 1). The sequence reads that did not match the reference genomes are retained and returned as a fasta file that can be used subsequently by a *de novo* assembly tool. Note that these subtraction steps can be skipped when the host genome is not known or if the aim of the user is to discover endogenous viral elements [21].

### (iii) Assembly, Blast and Parsing

#### *De novo* assembly

In the task “Assemble, Blast and Parse” (iii), retained RNA sequences are subjected to *de novo* assembly. For short reads (<50 nt), we tested several rounds of *de novo* assembly by Velvet [7] using the Oases software package [22] (Fig 1) and k-mer lengths ranging from 15 to 35 (S1 Table). For reads between 50 nt and 100 nt, we also used the Oases with k-mer lengths ranging from 13 to 69. Finally in Use Case 3–3, we used the Trinity assembly software which is available as a Galaxy tool and was reported to performs well with long reads [23]. Trinity as well as SPAdes [24] assembly softwares were also tested as alternate option to Oases in the Use Case 2–2 (S1 Table), giving similar outputs. It is noteworthy that users can adapt a Metavisitor workflow using any assembly software available in the Galaxy tool shed.

#### Blast

Next, *de novo* assembled contigs are aligned to both nucleotide and protein vir1 BLAST databases built from the viral reference sequences (Fig 1) using the blastn or blastx Galaxy tools [18]. These tools search nucleotide or protein databases using nucleotide or translated nucleotide queries, respectively [25]. The default parameters are adjusted in order to report only the 5 best alignments per contig (Maximum hits option is set to 5) and to generate a tabular blast output that includes the 12 standard columns plus a column containing the length of the aligned subject sequences (extended columns option, “slen” checked). Note that this additional column in the blast output is required for subsequent parsing of the blast output by the “Parse blast output and compile hits” tool.

#### Parsing

Tabular outputs generated by blastn and blastx alignments are processed by the “Parse blast output and compile hits” tool (S1 Table), which returns 4 files, namely “blast analysis, by subjects”, “hits”, “Blast aligned sequences” and “Blast unaligned sequences”.

In the “blast analysis, by subjects” file (S2 Fig), the subject sequences in the viral nucleotide or protein blast databases that produced significant blast alignments (hits) with *de novo* assembled contigs are listed, together with those contigs and hit information (% Identity, Alignment Length, start and end coordinates of hits relatively to the subject sequence, percentage of the contig length covered by the hit, E-value and Bit Score of the hit). In addition, for each subject sequence in the list, the length in nucleotide or amino-acid of the subject sequence (Subject Length), the summed coverage of the subject by all contig hits (Total Subject Coverage) as well as the fraction of the subject length that this coverage represents (Relative Subject Coverage), and the best (Best Bit Score) and mean (Mean Bit Score) bit scores produced by contig hits are computed and indicated. A simplified output can be generated without contigs and blast information by using the “compact” option for the reporting mode of the “Parse blast output and compile hits” tool. Note that the total and relative subject coverages indicate how much of the virus sequence is covered by the reconstructed contigs, whereas the Bit scores allow to estimate the distances between the reconstructed contigs and the subject sequence.

The “hits” file contains the sequences of contig portions that produced significant alignment in the BLAST step (i.e. query hit sequences), flanked by additional contig nucleotides 5’ and 3’ to the hit (the size of these margins is set to 5 by default and can be modified by the user). These margins allow to include sequences that might not have significant homology but could still be of viral origin.

Finally, the “Blast aligned sequences” file contains contigs that produced significant blast hits, whereas the “Blast unaligned sequences” file contains those that did not.

### (iv) Blast-Guided Scaffolding

This last task allows to integrate hit sequences matching a candidate virus into a virus scaffold (Fig 1). First, blastn or blastx hits are retrieved from the “hits” file using the tool “Pick Fasta sequences” (S1 Table) and the appropriate query string (for instance, “Dengue” will retrieve hit sequences that significantly blast aligned with Dengue virus sequences). Next, these hit sequences can be further clustered in longer contigs using the “cap3 Sequence Assembly” Galaxy tool (S1 Table) adapted from CAP3 [26]. Finally, if there are still multiple unlinked contigs at this stage, they can be integrated (uppercase characters) in the matched viral sequence taken as a scaffold (lowercase characters). This scaffolding is achieved by (a) retrieving the viral sequence from the NCBI nucleotide database to be used as the backbone of the scaffold, generating a blast index from this sequence and aligning the contigs to this index with blastn or tblastx tools (b) running the “blast_to_scaffold” tools (S1 Table), taking as inputs the contigs, the viral guide sequence and the blastn or blastx output (Fig 1, bottom).

### Availability of Metavisitor

All Metavisitor tools, workflows and use cases are available on the Galaxy server http://mississippi.snv.jussieu.fr. Readers can import in their personal account the published Metavisitor use case histories and their corresponding workflows to re-run the described analyses or adapt them to their studies.

We made all tools and workflows that compose Metavisitor available from the main Galaxy tool shed (https://toolshed.g2.bx.psu.edu/), in the form of a tool suite (suite_metavisitor_1_2) which thus can be installed and used on any Galaxy server instance. The Metavisitor workflows are also available from the *myexperiment* repository (http://www.myexperiment.org/) They can be freely modified or complemented with additional analysis steps within the Galaxy environment.

The Metavisitor tool codes are accessible in our public GitHub repository (https://github.com/ARTbio/tools-artbio/). We also provide a Docker image artbio/metavisitor:1.2 as well as an ansible playbook that both allow to deploy a Galaxy server instance with preinstalled Metavisitor tools and workflows in local infrastructures. Extensive documentation on how to install and use Metavisitor is available at https://artbio.github.io/Metavisitor-manual/.

## Results

The strategy implemented by Metavisitor (Fig 1) is to perform *de novo* assembly of sequencing reads and to detect contigs of viral origin through blast alignments to a nucleotide or protein sequence database of known viruses (vir1). These contig alignments can be further clustered to reconstruct a viral genome.

Below, we report use cases to demonstrate the use of Metavisitor in specific situations. For each use case, we briefly present the purpose of the original study from which the datasets originate and we describe an adapted Metavisitor workflow as well as its main outputs. Readers can further examine the workflows (https://mississippi.snv.jussieu.fr/workflow/list_published) and use case analyses (https://mississippi.snv.jussieu.fr/history/list_published) in every detail at http://mississippi.fr. Indicative execution times of the workflows are given in S2 Table.

### 1. Detection of known viruses

#### Use Cases 1–1, 1–2 and 1–3: detection and reconstruction of the Nora virus genome in small RNA sequencing datasets

Using small RNA sequencing libraries SRP013822 (EBI ENA) and the Paparazzi software [4] we were previously able to propose a novel reference genome (NCBI JX220408) for the Nora virus strain infecting *Drosophila melanogaster* stocks in laboratories [3]. This so-called rNora genome differed by 3.2% nucleotides from the Nora virus reference NC_007919.3 and improved the alignment rate of viral siRNAs by ~121%. Thus, we first tested Metavisitor on the small RNA sequencing datasets SRP013822 using the Oases *de novo* assembly tool which is well suited to assembly of short read [9].

Three Metavisitor workflows were run on the merged SRP013822 small RNA sequence reads and the NC_007919.3 genome as a guide for final scaffolding. The workflow for Use Case 1–1 (S3 Fig) used raw reads collapsed to unique sequences (experimental procedures) to reconstruct a Nora virus genome referred to as Nora_MV (S1 File). In a second workflow for Use Case 1–2 (S4 Fig), we did not collapse the SRP013822 reads to unique sequences, which allowed the reconstruction of a Nora_raw_reads genome (S2 File). Finally, the workflow for Use Case 1–3 (S5 Fig) normalized the abundances of SRP013822 sequence reads using the Galaxy tool “Normalize by median” [27] and reconstructed a Nora_Median-Norm-reads genome (S3 File).

**Fig 2:**
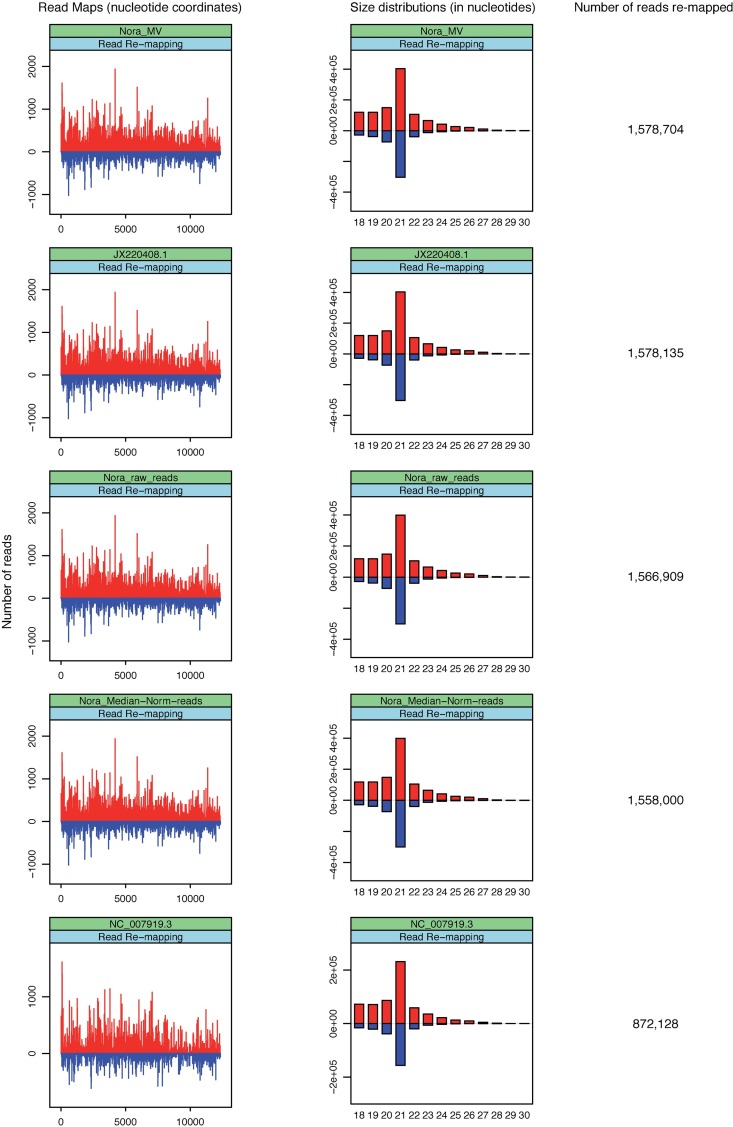
Realignments of small RNA sequence reads to reconstructed (Nora_MV, Nora_raw_reads and Nora_Median−Norm−reads) or published (JX220408.1 and NC_007919.3) Nora virus genomes. Plots (left) show the abundance of 18–30-nucleotide (nt) small RNA sequence reads matching the genome sequences and histograms (middle) show length distributions of these reads. Positive and negative values correspond to sense and antisense reads, respectively. Total read counts are indicated to the right hand side.

All three reconstructed genomes as well as the Paparazzi-reconstructed JX220408 genome had a high sequence similarity (>96.6% nucleotide identity) with the NC_007919.3 guide genome (S4 File). The final *de novo* (capital letters) assemblies of both the Nora_raw_reads and Nora_Median-Norm-reads genomes entirely covered the JX220408 and NC_007919.3 genomes (both 12333 nt), whereas the *de novo* assembled part of the Nora_MV genome was marginally shorter (12298 nt, the 31 first 5’ nucleotides are in lowercase to indicate that they were not *de novo* assembled but instead recovered from the guide genome). To evaluate the quality of assemblies, we remapped the SRP013822 reads to the 3 reconstituted Nora virus genomes as well as to the JX220408 guide genome using the “workflow for remapping in Use Cases 1–1,2,3” (S6 Fig). As can be seen in Fig 2, SRP013822 reads matched the genomes with almost identical profiles and had characteristic size distributions of viral siRNAs with a major peak at 21 nucleotides. Importantly, the numbers of reads re-matched to the Nora virus genomes were 1,578,704 (Nora_MV) > 1,578,135 (Paparazzi—JX220408) > 1,566,909 (Nora_raw_reads) > 1,558,000 (Nora_Median-Norm-reads) > 872,128 (NC_007919.3 reference genome guide).

Thus, Metavisitor reconstructed a Nora virus genome Nora_MV whose sequence maximizes the number of vsiRNA read alignments which suggests it is the most accurate genome for the Nora virus present in the datasets. Of note, the Nora_MV genome differs from the JX220408 rNora genome generated by Paparazzi by only two mismatches at positions 367 and 10707, and four 2nt-deletions at positions 223, 365, 9059 and 12217 (see S4 File). These variations did not change the amino acid sequence of the 4 ORFs of the Nora virus. We conclude that Metavisitor performs slightly better than Paparazzi for a known virus, using *de novo* assembly of small RNA reads followed by blast-guided scaffolding. We did not observe any benefits of using raw reads or normalized-by-median reads for *de novo* assembly with Oases, but rather a decrease in the accuracy of the reconstructed genome as measured by the number of reads re-mapped to the final genomes (Fig 2).

#### Use Case 1–4: detection of multiple viruses in small RNA sequencing datasets

In order to show the ability of Metavisitor in detecting multiple known viruses in small RNA sequencing datasets, we built another workflow Case that performs blastn alignments of Oases contigs on the vir1 reference and reports for all significant alignments without filtering (S7 Fig). Applying this workflow to the SRP013822 sequence datasets produced a list of alignments which contains, as expected, the Nora virus. In addition, contigs were found to align with high significance (Mean BitScore > 200) to the Drosophila A virus and to the Drosophila C virus (S5 File and Table 1), strongly suggesting that the fly stocks analyzed in our previous work were also subject to persistent infection by these viruses [3].

**Table 1:** Report table generated by the “Parse blast output and compile hits” tool in Use Case 1–4 showing the presence of *Drosophila* A virus and *Drosophila* C virus in addition to the Nora virus in the small RNA sequencing of laboratory *Drosophila*. See Method section for a description of the columns.

subject	subject length	Total Subject Coverage (nt)	Relative Subject Coverage	Best Bit Score	Mean Bit Score
gi|157325505|gb|DQ321720.2|_Nora_virus,_complete_genome	11908	10211	0.857	11840	4041
gi|822478532|gb|KP970099.1|_Nora_virus_isolate_RAKMEL13_gp1_(gp1)_gene,_partial_cds;_and_replicatio	11416	8736	0.765	11441	3673
gi|822478537|gb|KP970100.1|_Nora_virus_isolate_GEO58_gp1_(gp1)_gene,_partial_cds;_and_replication_p	11416	2463	0.216	4028	3607
gi|346421290|ref|NC_007919.3|_Nora_virus,_complete_genome	12333	10530	0.854	11809	2653
gi|284022350|gb|GQ257737.1|_Nora_virus_isolate_Umea_2007,_complete_genome	12333	10530	0.854	11809	2573
gi|822478527|gb|KP970098.1|_Nora_virus_isolate_AM04_gp1_(gp1)_gene,_partial_cds;_and_replication_po	11413	7654	0.671	5745	2489
gi|822478512|gb|KP970095.1|_Nora_virus_isolate_RAK11_gp1_(gp1)_and_replication_polyprotein_(gp2)_ge	11416	6174	0.541	5368	2419
gi|822478141|gb|KP969947.1|_Drosophila_A_virus_isolate_ywiP_DrosophilaA_RNA-dependent_RNA_polymeras	4516	4157	0.921	6980	2361
gi|402295620|gb|JX220408.1|_Nora_virus_isolate_FR1,_complete_genome	12333	12302	0.997	12720	2324
gi|822478147|gb|KP969949.1|_Drosophila_A_virus_isolate_delta11_DrosophilaA_RNA-dependent_RNA_polyme	4481	4442	0.991	7081	2264
gi|822478417|gb|KP970078.1|_Nora_virus_isolate_D167_gp1_(gp1),_replication_polyprotein_(gp2),_gp3_(	11895	7315	0.615	5503	2192
gi|822478517|gb|KP970096.1|_Nora_virus_isolate_K09_gp1_(gp1)_gene,_partial_cds;_and_replication_pol	11419	2027	0.178	3490	2050
gi|822478144|gb|KP969948.1|_Drosophila_A_virus_isolate_XIB_DrosophilaA_RNA-dependent_RNA_polymerase	4516	4507	0.998	7092	2009
gi|822478150|gb|KP969950.1|_Drosophila_A_virus_isolate_Qdelta_DrosophilaA_RNA-dependent_RNA_polymer	4476	4446	0.993	7092	1847
gi|822478440|gb|KP970082.1|_Nora_virus_isolate_RAKMEL12_gp1_(gp1),_replication_polyprotein_(gp2),_g	11968	7214	0.603	6396	1815
gi|822478497|gb|KP970092.1|_Nora_virus_isolate_delta11_gp1_(gp1)_gene,_partial_cds;_replication_pol	11157	2347	0.210	3314	1695
gi|822478522|gb|KP970097.1|_Nora_virus_isolate_JJ17_gp1_(gp1)_gene,_partial_cds;_and_replication_po	11420	993	0.087	1674	1659
gi|822478482|gb|KP970089.1|_Nora_virus_isolate_IM13_gp1_(gp1)_gene,_partial_cds;_replication_polypr	11103	1828	0.165	1977	1565
gi|225356593|gb|FJ150422.1|_Drosophila_A_virus_isolate_HD,_complete_genome	4806	4753	0.989	6902	1419
gi|822478430|gb|KP970080.1|_Nora_virus_isolate_MONSIM03_gp1_(gp1),_replication_polyprotein_(gp2),_g	11968	2086	0.174	2993	1416
gi|822478445|gb|KP970083.1|_Nora_virus_isolate_SAF04_gp1_(gp1),_replication_polyprotein_(gp2),_gp3_	11142	649	0.058	1121	1121
gi|822478135|gb|KP969945.1|_Drosophila_A_virus_isolate_XID_DrosophilaA_RNA-dependent_RNA_polymerase	4516	4074	0.902	3103	1112
gi|822478403|gb|KP970076.1|_Nora_virus_isolate_ATH56_gp1_(gp1)_and_replication_polyprotein_(gp2)_ge	11965	2344	0.196	2812	1025
gi|822478412|gb|KP970077.1|_Nora_virus_isolate_IM09_gp1_(gp1),_replication_polyprotein_(gp2),_gp3_(	11965	3689	0.308	2989	1023
gi|822478435|gb|KP970081.1|_Nora_virus_isolate_MON28_gp1_(gp1)_and_replication_polyprotein_(gp2)_ge	11967	5859	0.490	5445	1004
gi|822478507|gb|KP970094.1|_Nora_virus_isolate_K02_gp1_(gp1)_gene,_partial_cds;_replication_polypro	11160	1289	0.116	957	778
gi|822478132|gb|KP969944.1|_Drosophila_A_virus_isolate_wipe_DrosophilaA_RNA-dependent_RNA_polymeras	4516	3070	0.680	3097	742
gi|822478477|gb|KP970088.1|_Nora_virus_isolate_IM12_gp1_(gp1)_gene,_partial_cds;_replication_polypr	11413	952	0.083	1153	732
gi|253761971|ref|NC_012958.1|_Drosophila_A_virus,_complete_genome	4806	607	0.126	1045	674
gi|822478542|gb|KP970101.1|_Nora_virus_isolate_SAFSIM01_gp1_(gp1)_gene,_partial_cds;_and_replicatio	11413	384	0.034	661	661
gi|822478138|gb|KP969946.1|_Drosophila_A_virus_isolate_LJ35_DrosophilaA_RNA-dependent_RNA_polymeras	4468	959	0.215	848	502
gi|9629650|ref|NC_001834.1|_Drosophila_C_virus,_complete_genome	9264	6345	0.685	1276	445
gi|2388672|gb|AF014388.1|_Drosophila_C_virus_strain_EB,_complete_genome	9264	6587	0.711	1276	431
gi|300871949|gb|GU983882.2|_Drosophila_C_virus_isolate_ZW141_polyprotein_gene,_partial_cds	500	272	0.544	482	395
gi|300871965|gb|GU983892.2|_Drosophila_C_virus_isolate_psjmg_polyprotein_gene,_partial_cds	500	310	0.620	491	353
gi|300871979|gb|GU983900.2|_Drosophila_C_virus_isolate_AL7_polyprotein_gene,_partial_cds	500	271	0.542	489	342
gi|300871957|gb|GU983888.2|_Drosophila_C_virus_isolate_Bam73_H_polyprotein_gene,_partial_cds	500	453	0.906	491	323
gi|300871941|gb|GU983878.2|_Drosophila_C_virus_isolate_mel15_H_polyprotein_gene,_partial_cds	500	453	0.906	489	321
gi|300871955|gb|GU983885.2|_Drosophila_C_virus_isolate_16a9_polyprotein_gene,_partial_cds	490	151	0.308	273	262
gi|300871953|gb|GU983884.2|_Drosophila_C_virus_isolate_Tam15_polyprotein_gene,_partial_cds	500	151	0.302	273	262

### 2. Discovery of novel viruses

#### Use Case 2–1: identification of new viruses in small RNA sequencing datasets

Using Metavisitor, we recently discovered two novel viruses infecting a laboratory colony of *Anopheles coluzzii* mosquitoes [28]. In this case, a workflow (S8 Fig) was used to process small RNA datasets from these mosquitoes (EBI SRA ERP012577) and to assemble a number a Oases contigs that show significant blastx hits with *Dicistroviridae* proteins, including *Drosophila* C virus (DCV) and Cricket paralysis virus (CrPV) proteins (S6 File).

The viral family of *Dicistroviridae* was named from the dicistronic organisation of their genome with a 5’ open reading frame encoding a non-structural polyprotein and a second non-overlapping 3’ open reading frame encoding the structural polyprotein. In order to construct a potential new *A*. *coluzzii* dicistrovirus genome, the “Pick Fasta Sequences” tool (S8 Fig) collected blastx hits showing significant alignment with both *Drosophila* C virus and Cricket paralysis viral polyproteins (S7 File) that were further clustered with the “cap3 Sequence Assembly” tool in 4 contigs of 1952, 341, 4688 and 320 nt, respectively (S8 File). These 4 contigs were further aligned to the DCV genome NC_001834.1 sequence with tblastx and integrated in this scaffold sequence with the “blast_to_scaffold” tool to produce a final assembly (S9 File). Re-mapping of the ERP012577 small RNA reads using the “Workflow for remapping in Use Cases 1–1,2,3” (S6 Fig) showed that they mostly align to *de novo* assembled regions (uppercase nucleotides) of this chimeric genome and have a typical size distribution of viral derived siRNA (S9 Fig), suggesting that the NC_001834.1 DCV sequences of the scaffold (lowercase nucleotides) are loosely related to the actual sequence of the novel *A*. *coluzzii* dicistrovirus. Nevertheless, the composite assembly allowed designing primers in the *de novo* assembled regions to PCR amplify and sequence the regions of the viral genome that could not be *de novo* assembled [28].

Several teams have used siRNA signature (a peak at 21 nt in the size distribution of re-aligned small RNA sequences) as an alternate approach to sequence similarity to identify contigs of potential viral origin [12,13]. In order to further illustrate the flexibility of Metavisitor for implementing this strategy, we built a workflow (S10 Fig) to realign ERP012577 small RNA sequences to Oases contigs of length higher than 300 nt and to generate in batch read maps and read size distributions for these contigs using the “Generate readmap and histograms from alignment files” tool (S1 Table). We manually inspected these read maps and size distributions (S10 File) and collected all contigs with a clear siRNA signature (a pick at 21nt for both forward and reverse strands of contig sequences), 2 sets of contigs with a modest excess of 21nt reads from the forward strand only and 3 sets of contigs with no siRNA signature as negative controls (S3 Table). With the notable exceptions of loci 3 and 46 contigs, all contigs with a clear siRNA signature blastx aligned to vir1 viral sequences (S3 Table and see here the public Galaxy history for details). Loci 3 and 46 contigs did not align either to the non-redundant protein database of the NCBI and may therefore be of potential viral origin (S3 Table). All 5 negative control contigs with unclear or no RNA signature only aligned significantly to non-viral proteins (S3 Table). Together, these results illustrate the use of Metavisitor to implement a sequence-independent strategy based on siRNAs for virus identification [12,13].

#### Use Case 2–2: identification of new viruses in mRNA sequencing datasets

**Fig 3:**
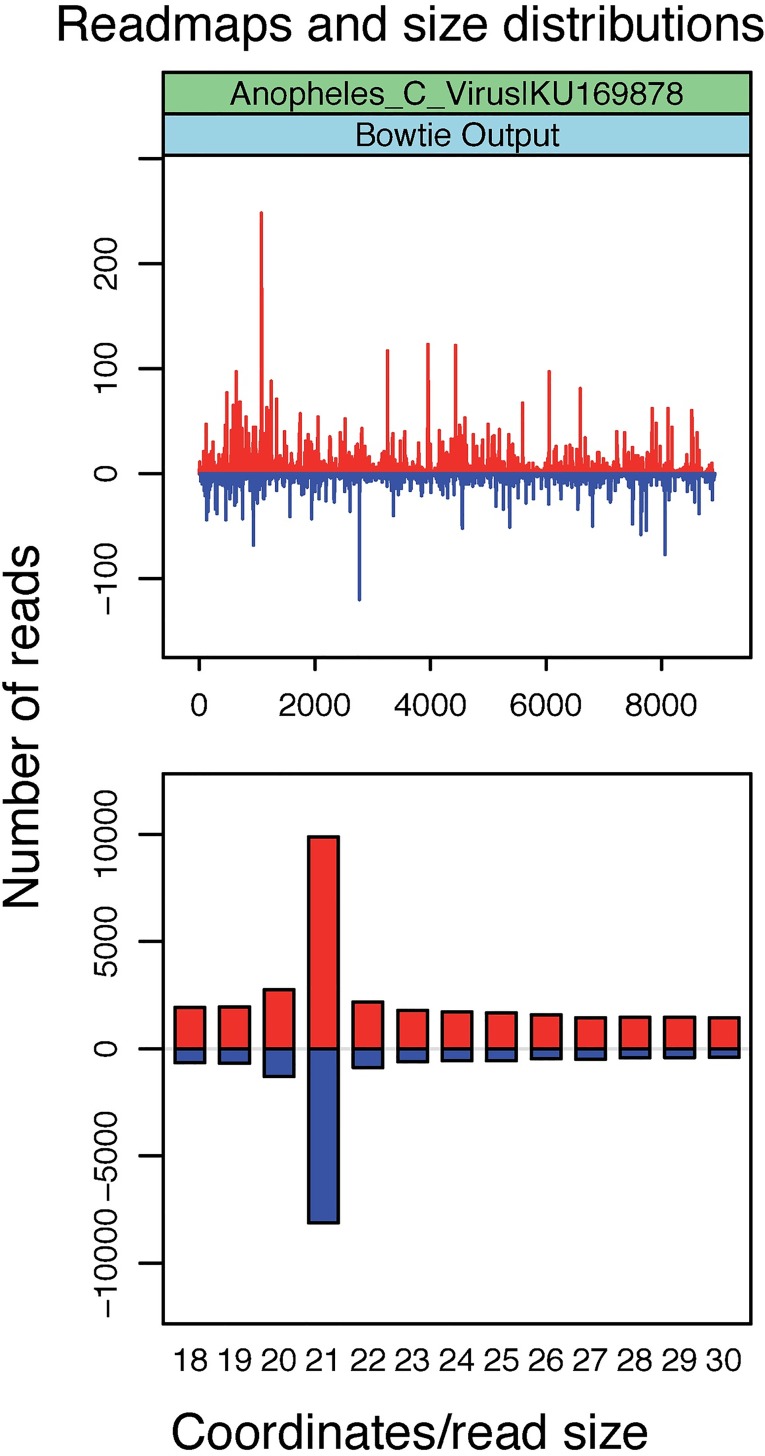
Alignments of small RNA sequence reads to the Anopheles C virus genome reconstructed in Use Case 2–2. Plot shows the abundance of 18–30-nucleotide (nt) small RNA sequence reads matching the genome sequence and histogram shows the length distribution of these reads. Positive and negative values correspond to sense and antisense reads, respectively.

In our study [28], we also used RNAseq libraries from the same *A*. *coluzzii* colony (EBI-SRA, ERS977505), demonstrating the use of a Metavisitor workflow for long RNA sequencing read datasets (S11 Fig). Thus, 100nt reads were aligned without adapter clipping to the *Anopheles gambiae* genome using bowtie2, and unmatched read were subjected to Oases assembly (kmer range 25 to 69, to take into account longer reads). Oases contigs were then filtered for a size > 5000 nt and aligned to the protein viral reference using blastx. Parsing of blastx alignments with the “blast analysis, by subjects” tool repeatedly pointed to a 8919nt long Oases contig that matched to structural and non-structural polyproteins of DCV and CrPV (S11 File). This 8919nt contig (S12 File) completely includes the contigs generated with the small RNA datasets (S8 File) and shows a dicistronic organization which is typical of Dicistroviridae and is referred to as a novel *Anopheles* C Virus [28]. The sequence of this *Anopheles* C Virus is deposited to the NCBI nucleotide database under accession number KU169878. As expected, when realigned to this genome (S12 Fig), the ERP012577 small RNA reads now show a typical alignment profile all along the AnCV genome sequence with a size distribution peaking at the 21nt length of viral derived siRNAs and no gap (Fig 3). Of note, we tested in Use Case 2–2 two alternate workflows substituting the Oases assembly tool with Trinity (S13 Fig) and SPAdes (S14 Fig), respectively. Both these workflows were equally able to assemble the genome KU169878 of the Anopheles C Virus (S13 File).

Taken together, the Metavisitor Use Cases 2–1 and 2–2 illustrate that when short read datasets do not provide enough sequencing information, an adapted Metavisitor workflow (S11 Fig) is able to exploit long reads of RNA sequencing datasets, if available, to assemble a complete viral genome [28].

### 3. Virus detection in human RNAseq libraries

Having illustrated that Metavisitor is able to generate robust genome assemblies from known and novel viruses in *Drosophila* and *Anopheles* sequencing datasets, we tested whether it can be used to diagnose viruses in RNA sequencing datasets of human patients from three different studies [29–31].

#### Use Case 3–1

Innate lymphoid cells (ILCs) play a central role in response to viral infection by secreting cytokines crucial for immune regulation, tissue homeostasis, and repair. Therefore, the pathogenic effect of HIV on these cells was recently analyzed in infected or uninfected patients using various approaches, including transcriptome profiling [30]. ILCs are unlikely to be infected *in vivo* by HIV as they lack expression of the CD4 co-receptor of HIV and they are refractory *in vitro* to HIV infection. However, we reasoned that ILCs samples could still be contaminated by infected cells. This might allow Metavisitor to detect and assemble HIV genomes from patient’s ILC sequencing data (EBI SRP068722).

We imported 40 ICL sequence datasets from the EBI SRP068722 archive and merged the datasets belonging to the same patients. As the data contained short 32 nt reads that in addition had to be 3’ trimmed to 27 nt to retain acceptable sequence quality, we designed a workflow for Use Case 3–1 (S15 Fig) that is similar to the workflows used in cases 1–1 and 2–1 for small RNA sequencing data. Thus, the sequencing datasets were depleted from reads aligning to the human genome (hg19) and viral reads were selected by alignment to the NCBI viral sequences using the sRbowtie tool (S1 Table). These reads were further submitted to Oases assembly (kmers 11 to 27, to take into account short reads) and the resulting contigs were aligned to the Nucleotide Viral Blast Database using blastn. Alignments were parsed using the “Parse blast output and compile hits” tool, removing alignments to NCBI sequences related to patents to simplify the report (“Patent” term in the filter option of the “Parse blast output and compile hits” tool). A final report was generated by concatenating the reports produced by this tool for each patient (S14 File and Table 2). In summary, we were able to detect HIV RNAs in samples from 3 out of 4 infected patients whereas all samples from control uninfected patients remained negative for HIV. This Metavisitor workflow was able to accurately detect HIV RNA, even in samples where the number of sequence reads was expected to be low.

**Table 2:** HIV detection in ILC patient samples of Use Case 3–1. The table summarizes the report generated by Metavisitor from a batch of 40 sequence datasets (S14 File). Metadata associated with each indicated sequence dataset as well as the ability of Metavisitor to detect HIV in datasets and patients are indicated.

# GSM ID	ID-1	Patient	Treatment (SRR annotations)	HIV status	Days post HIV infection	Treat. status	SRR ID	Metavisitor HIV detection by sample	number of raw reads	number of raw reads by patient	History for Use Case 3–1
GSM2043730	110314	0450–318	ILC2	HIV+	1	untreated	SRR3111582	+	7 013 962	34 252 732	gi|45357423|gb|AY535449.1, gi|45357419|gb|AY535447.1
GSM2043731	110314	0450–318	ILC3	HIV+	1	untreated	SRR3111583	-	3 246 980		
GSM2043732	180314	0450–318	ILC2	HIV+	7	untreated	SRR3111584	+	2 833 634		
GSM2043733	180314	0450–318	ILC3	HIV+	7	untreated	SRR3111585	+	2 989 628		
GSM2043734	170414	0450–318	ILC2	HIV+	38	untreated	SRR3111586	-	16 248 912		
GSM2043735	170414	0450–318	ILC3	HIV+	38	untreated	SRR3111587	-	1 919 616		
GSM2043736	110614	0387–272	ILC2	HIV+	1	untreated	SRR3111588	-	60 342 796	227 307 414	-
GSM2043737	110614	0387–272	ILC3	HIV+	1	untreated	SRR3111589	-	34 189 278		
GSM2043738	170614	0387–272	ILC2	HIV+	7	untreated	SRR3111590	-	38 030 394		
GSM2043739	170614	0387–272	ILC3	HIV+	7	untreated	SRR3111591	-	29 100 534		
GSM2043740	290714	0387–272	ILC2	HIV+	49	untreated	SRR3111592	-	43 022 506		
GSM2043741	290714	0387–272	ILC3	HIV+	49	untreated	SRR3111593	-	22 621 906		
GSM2043742	41214	0629–453	Acute ART+ ILC2	HIV+	1	ART	SRR3111594	+	5 061 920	54 052 098	gi|296033826|gb|GU474419.1, gi|269294806|dbj|DM461231.1, gi|269294805|dbj|DM461230.1, gi|296556482|gb|AF324493.2gi|296556485|gb|M19921.2, gi|45357419|gb|AY535447.1, gi|45357423|gb|AY535449.1
GSM2043743	41214	0629–453	Acute ART+ ILC3	HIV+	1	ART	SRR3111595	-	8 455 026		
GSM2043744	101214	0629–453	Acute ART+ ILC2	HIV+	6	ART	SRR3111596	-	12 451 684		
GSM2043745	101214	0629–453	Acute ART+ ILC3	HIV+	6	ART	SRR3111597	+	6 419 868		
GSM2043746	130115	0629–453	Acute ILC2	HIV+	40	ART	SRR3111598	+	6 837 584		
GSM2043747	130115	0629–453	Acute ILC3	HIV+	40	ART	SRR3111599	+	14 826 016		
GSM2043748	150714	0444–312	3dR10 ILC2	HIV+	2	ART	SRR3111600	+	15 618 282	39 610 902	gi|45357423|gb|AY535449.1, gi|45357419|gb|AY535447.1
GSM2043749	150714	0444–312	3dR10 ILC3	HIV+	2	ART	SRR3111601	+	13 491 804		
GSM2043750	220814	0444–312	ILC2	HIV+	41	ART	SRR3111602	-	5 259 104		
GSM2043751	220814	0444–312	ILC3	HIV+	41	ART	SRR3111603	-	5 241 712		
GSM2043752	10814	0500-355neg	ILC2	HIV-	uninfected	none	SRR3111604	-	802 632	11 691 304	-
GSM2043753	10814	0500-355neg	ILC3	HIV-	uninfected	none	SRR3111605	-	10 888 672		
GSM2043754	80814	0292-xxxneg	ILC2	HIV-	uninfected	none	SRR3111606	-	5 418 958	19 222 152	-
GSM2043755	80814	0292-xxxneg	ILC3	HIV-	uninfected	none	SRR3111607	-	13 803 194		
GSM2043756	90714	0394–274	ILC2	HIV-	uninfected	none	SRR3111608	-	13 779 570	15 991 428	-
GSM2043757	90714	0394–274	ILC3	HIV-	uninfected	none	SRR3111609	-	2 211 858		
GSM2043758	170714	0218-162neg	ILC2	HIV-	uninfected	none	SRR3111610	-	9 838 776	18 939 560	-
GSM2043759	170714	0218-162neg	ILC3	HIV-	uninfected	none	SRR3111611	-	9 100 784		
GSM2043760	180314	0311-217HIVneg	ILC2	HIV-	uninfected	none	SRR3111612	-	2 281 560	7 490 832	-
GSM2043761	180314	0311-217HIVneg	ILC3	HIV-	uninfected	none	SRR3111613	-	5 209 272		
GSM2043762	230514	0440-307neg	ILC2	HIV-	uninfected	none	SRR3111614	-	11 816 186	21 714 164	-
GSM2043763	230514	0440-307neg	ILC3	HIV-	uninfected	none	SRR3111616	-	9 897 978		
GSM2043764	240614	0518-370neg	ILC2	HIV-	uninfected	none	SRR3111617	-	16 135 602	16 671 200	-
GSM2043765	240614	0518-370neg	ILC3	HIV-	uninfected	none	SRR3111618	-	535 598		
GSM2043766	290714	0560-420neg	ILC2	HIV-	uninfected	none	SRR3111619	-	1 235 766	12 912 002	-
GSM2043767	290714	0560-420neg	ILC3	HIV-	uninfected	none	SRR3111620	-	11 676 236		
GSM2043768	290714	0575-419neg	ILC2	HIV-	uninfected	none	SRR3111621	-	8 713 816	11 833 416	-
GSM2043769	290714	0575-419neg	ILC3	HIV-	uninfected	none	SRR3111622	-	3 119 600		

#### Use Case 3–2

Yozwiak *et al*. searched the presence of viruses in RNA Illumina sequencing data from serums of children suffering from fevers of unknown origins [29]. In this study, paired-end sequencing datasets were depleted from reads aligning to the human genome and the human transcriptome using BLAT and BLASTn, respectively, and the remaining reads were aligned to the NCBI nucleotide database using BLASTn. A virus was considered identified when 10 reads or more aligned to a viral genome which was not tagged as a known lab contaminant.

For a significant number of Patient IDs reported in Table 1 of the article [29], we were not able to find the corresponding sequencing files in the deposited EBI SRP011425 archive. In addition, we did not find the same read counts for these datasets as those indicated by the authors. With these limitations in mind, we downloaded 86 sequencing datasets that could be further concatenated and assigned to 36 patients in Yozwiak *et al* [29]. As sequence reads in SRP011425 datasets are 97 nt long, we adapted a workflow for this Use Case 3–2 (S16 Fig) from the one used in the Use Case 3–1 with the following modifications: (i) sequences reads were depleted from human sequences and viral reads were selected by alignment to the NCBI viral sequences using the Galaxy bowtie2 tool (S1 Table) instead of the sRbowtie tool; (ii) viral reads were submitted to Oases assembly using kmer values ranging from 13 to 69 to take into account long reads; (iii) the SAM file with reads alignments to the vir1 bowtie2 index was parsed using the “join” and “sort” Galaxy tools in order to detect putative false negative datasets with viral reads that fails to produce significant Oases viral contigs.

This workflow generated a report file (S15 File) summarized in Table 3. The results show that Metavisitor detected the same viruses as those reported by Yozwiak *et al*. in 17 patients. Although viral reads were detected in 16 other patients, they were not covering sufficient portions of viral genomes to produce significant viral assemblies. Finally, in the three remaining patients (patients 363, 330 and 345 in Table 3), we detected viruses (Dengue virus 2, Stealth virus 1 and Dengue virus 4, respectively) other than those identified by Yozwiak *et al*. These discrepancies are most likely due to misannotation of some of the deposited datasets, which precludes further detailed comparisons.

**Table 3:** Summary of virus detection in 36 traceable patients of the Use Case 3–2. The Data of this table were extracted from the Metavisitor report file available as S15 File. Values of the column “Coverage of complete viral genome (%)” correspond to the fractions (in %) of the complete viral genomes that are covered by blast hits of viral contigs to these genomes and values of the column “Mean blast bit score” correspond to the mean values of the bit scores observed for these blast hits. Note that blast alignments to incomplete viral genomes were not taken into account. For detection of false positives, reads were aligned to the bowtie2 vir1 index before *de novo* assembly and counts of these reads were reported in the column “Read mapping to vir1 using bowtie2”).

Extracted from Yozwiak et al. Table 1					Metavisitor					
Patient ID	# virus reads	# initial reads	Fraction virus reads	Yozwiak et al. Virus detection	# reads in NGS datasets	Metavisitor Virus detection	Coverage of complete viral genome (%)	Mean blast bit score	Read mapping to vir1 using bowtie2	ENA-RUN
566	206	1.90E+06	1.08E-04	Torque teno mini virus 4	1.07E+06	none	-	-	No Significant alignments	SRR453487
438	72	4.40E+06	1.64E-05	Human herpesvirus 6	3.20E+06	Human herpesvirus 6	0.36	239.3	49 Human_herpesvirus_6	SRR453437
401	2164	1.80E+05	1.20E-02	Hepatitis A virus	9.94E+05	Hepatitis A virus	69.74	735.2	6154 reads Hepatitis_A_virus	SRR453443,SRR453458
382	44	9.60E+05	4.58E-05	Human herpesvirus 4	2.33E+06	none	-	-	38 Human_herpesvirus_4	SRR453430
377	81	6.60E+06	1.23E-05	Cyclovirus PK5034	4.91E+06	Circovirus-like_NI/2007-3	20.45	212.0	58 reads Circovirus-like_NI/2007-376 Torque teno virus	SRR453491
375	53	3.80E+06	1.39E-05	Porcine circovirus 1	2.65E+06	Circovirus-like_NI/2007-3	20.63	214.0	66 reads Circovirus-like_NI/2007-3	SRR453499
350	48	3.00E+06	1.60E-05	Human herpesvirus 6, Torque teno mini virus 4	1.93E+06	none	-	-	29 Human_herpesvirus_6128 Torque teno mini virus 4	SRR453484
349	47	1.60E+06	2.94E-05	Torque teno midi virus	1.03E+06	none	-	-	No Significant alignments	SRR453464
345	62	2.20E+06	2.82E-05	Beak and feather disease virus	1.29E+06	Dengue_virus_4	0.81	156.0	159 Dengue_virus_465 Circovirus-like_NI/2007	SRR453506
344	303	1.30E+06	2.33E-04	Human herpesvirus 6	7.48E+05	Human herpesvirus 6	0.17	304.0	184 Human_herpesvirus_6	SRR453417
335	47	1.70E+06	2.76E-05	Torque teno virus	9.77E+05	none	-	-	24 Torque teno virus	SRR453490
331	113	1.40E+06	8.07E-05	Torque teno midi virus 2	8.99E+05	UNVERIFIED:_Torque_teno_virus_isolate_S55,_complete_genome	20.93	353.4	772 Torque teno virus	SRR453478
330	77	1.80E+06	4.28E-05	TTV-like mini virus	1.85E+06	AF191073_Stealth_virus_1_clone_3B43	2.68	143.0	14 reads Stealth_virus_1_clone_C16130_T324 reads Dengue virus 2	SRR453465,SRR453480
329	14	2.20E+06	6.36E-06	Gull circovirus	2.93E+06	Circovirus-like_NI/2007-3	53.64	339.5	5 reads Circovirus-like_NI/2007-312 reads Dengue virus 2	SRR453489,SRR453505
322	206	3.80E+06	5.42E-05	Cyclovirus PK5222	2.63E+06	Circovirus-like_NI/2007-3	25.35	257.0	208 reads Circovirus-like_NI/2007-3	SRR453498
321	30	4.60E+06	6.52E-06	Porcine circovirus 1	3.54E+06	none	-	-	10 reads Circovirus-like_NI/2007-317 reads Dengue virus 2	SRR453446
315	42	1.90E+06	2.21E-05	African swine fever virus	1.59E+06	none	-	-	22 reads Dengue virus 2	SRR453427,SRR453440
282	699	1.60E+06	4.20E-04	Dengue virus 2	9.66E+05	Dengue virus 2	3.63	495.0	651 reads Dengue virus 2	SRR453438
275	1511	1.60E+06	9.70E-04	Dengue virus 2	1.11E+06	Dengue virus 2	10.23	539.2	1436 reads Dengue virus 2	SRR453450
274	27	1.20E+06	2.30E-05	Dengue virus 1	6.24E+05	none	-	-	28 reads Dengue virus 1/2	SRR453460
270	28	1.20E+06	2.33E-05	Human herpesvirus 6	6.76E+05	none	-	-	20 Human_herpesvirus_6	SRR453485
266	135749	4.80E+06	2.80E-02	Dengue virus 2	3.36E+06	Dengue virus 2	98.65	1852.3	121347 reads Dengue virus 2	SRR453448
263	ND	ND	ND	TTV (virochip)	3142332	Dengue virus 2	39.19	302.0	75 densovirus92 Dengue virus	SRR453424,SRR453457
193	56	1.60E+06	3.50E-05	Torque teno mini virus 2	9.13E+05	none	-	-	6 reads Torque_teno_virus_isolate_TTV-S34	SRR453510
187	4280	1.10E+06	3.90E-03	Dengue virus 2	5.55E+05	Dengue virus 2	39.30	496.8	3970 reads Dengue virus 2	SRR453456
186	1701	2.00E+06	8.51E-04	Torque teno virus 15	1.32E+06	Torque teno virus (SEN virus)	55.85	389.4	541 SEN virus AY449524.127 reads Torque_teno_virus_15	SRR453425,SRR453469
183	66	3.00E+06	2.20E-05	Human herpesvirus 6	1.97E+06	none	-	-	57 Human_herpesvirus_6B	SRR453481
180	42	8.00E+05	5.25E-05	GB virus C	4.91E+06	GB virus C	1.26	185.0	41 reads GB virus	SRR453531
179	17	1.80E+06	9.44E-06	Torque teno mini virus 1	1.31E+06	none	-	-	No Significant alignments	SRR453474
171	18	1.20E+06	1.50E-05	Torque teno mini virus 2	7.81E+05	none	-	-	No Significant alignments	SRR453509
168	ND	ND	ND	TTV (virochip)	135412	none	-	-	No Significant alignments	SRR453451
161	14	3.00E+06	4.67E-06	Human parvovirus B19	2.75E+06	Human parvovirus B19	2.89	242.0	79 densovirus12 reads Human parvovirus B19	SRR453495,SRR453504
159	143	2.60E+06	5.50E-05	Torque teno mini virus 5	1.73E+06	Uncultured virus DNA	88.14	339.0	No Significant alignments	SRR453500
156	213	2.30E+06	9.26E-05	Torque teno midi virus 1	1.54E+06	Torque_teno_virus	31.01	314.7	550 reads Torque_teno_virus_isolate_S54	SRR453493
131	24	1.20E+06	2.00E-05	Human herpesvirus 6	5.46E+05	Human herpesvirus 6	0.18	263.0	16 reads Human_herpesvirus_6	SRR453444
78	113	1.20E+06	9.42E-05	Human herpesvirus 6	5.91E+05	none	-	-	68 reads Human_herpesvirus_6B	SRR453426

#### Use case 3–3

Matranga *et al*. recently improved library preparation methods for deep sequencing of Lassa and Ebola viral RNAs in clinical and biological samples [31]. Accordingly, they were able to generate sequence datasets of 150 nt reads providing high coverage of the viral genomes. We used these datasets, relevant in the context of Lassa and Ebola outbreak and epidemic response, to demonstrate the versatility of Metavisitor as well as its ability to generate high throughput reconstruction of viral genomes.

In order to take into account longer reads and higher viral sequencing depths in the available datasets [31], we adapted a Metavisitor workflow for Use Case 3–3 (S17 Fig) as follows: (i) sequencing reads were directly aligned to vir1 sequences using bowtie2, without prior depletion by alignment to the human or rodent hosts; (ii) the Trinity *de novo* assembler [23] that performs well with longer reads was used instead of Oases (S1 Table); (iii) reconstruction of Lassa and Ebola genomes from the sequences of the blast hits with the nucleotide viral blast database was directly performed with the “blast to scaffold” tool without CAP3 assembly since the Trinity contigs were already covering a significant part of the viral genomes; (iv) the reports generated by our “Parse blast output and compile hits” tool as well as the reconstructed genome generated for each sample were merged in single datasets for easier browsing and subsequent phylogenetic or variant analyses; (v) for adaptability of this workflow to any type of virus, we allowed users to specify two input variables at runtime: the name of the virus to be searched for in the analysis and the identifier of the sequence to be used as guide in genome reconstruction steps.

We imported 63 sequence datasets available in the EBI SRA PRJNA254017 and PRJNA257197 archives [31] and grouped these datasets in Lassa virus (55 fastq files) and Ebola virus (8 fastq files) dataset collections (see Table 4 for description of the sequence datasets). On the one hand, we executed the workflow (S17 Fig) taking the Lassa virus dataset collection as input sequences, “Lassa” as a filter term for the “Parse blast output and compile hits” tool and the NCBI sequence NC_004297.1 as a guide for reconstruction of the Lassa virus segment L. On the other hand, we executed the workflow taking the Ebola virus dataset collection as input sequences, “Ebola” as a filter term for the “Parse blast output and compile hits” tool and the NCBI sequence NC_002549.1 as a guide for reconstruction of the Ebola virus genome.

**Table 4:** Summary of detection of Ebola and Lassa viruses in Use Case 3–3. The table summarizes the Metavisitor report files available as S16 and S17 Files.

Virus	BioProject	BioSample id	SRX number	SRR number	Sample ID	BAM file name	Source	Data Type, Selection	Figure, Table from Matranga et al.	Metavisitor detection (Trinity)
EBOV	PRJNA257197	SAMN03099684	SRX733660	SRR1613381	G3676-2	G3676-2_S6_L001_001.bam	Human	RNase H	Figure 5	**+**
EBOV	PRJNA257197	SAMN03099684	SRX733656	SRR1613377	G3676-2	G3676-2-std_S13_L001_001.bam	Human	RNA seq	Figure 5	**+**
EBOV	PRJNA257197	SAMN03099685	SRX733661	SRR1613382	G3677-1	G3677-1_S3_L001_001.bam	Human	RNase H	Figure 5	**+**
EBOV	PRJNA257197	SAMN03099685	SRX733657	SRR1613378	G3677-1	G3677-1-std_S10_L001_001.bam	Human	RNA seq	Figure 5	**+**
EBOV	PRJNA257197	SAMN03099686	SRX733662	SRR1613383	G3677-2	G3677-2_S2_L001_001.bam	Human	RNase H	Figure 5	**+**
EBOV	PRJNA257197	SAMN03099686	SRX733658	SRR1613379	G3677-2	G3677-2-std_S9_L001_001.bam	Human	RNA seq	Figure 5	**+**
EBOV	PRJNA257197	SAMN03099687	SRX733663	SRR1613384	G3682-1	G3682-1_S4_L001_001.bam	Human	RNase H	Figure 5	**+**
EBOV	PRJNA257197	SAMN03099687	SRX733659	SRR1613380	G3682-1	G3682-1-std_S11_L001_001.bam	Human	RNA seq	Figure 5	**+**
LASV	PRJNA254017	SAMN02927412	SRX719120	SRR1595772	G2431	LASV678_ERCC117	Human	RNase H	Figure 2	**+**
LASV	PRJNA254017	SAMN02927412	SRX719079	SRR1595696	G2431	LASV678_ERCC12	Human	RNA seq	Figure 2	**+**
LASV	PRJNA254017	SAMN02927488	SRX719056	SRR1595665	ISTH1003	LASV347_ERCC126	Human	RNase H	Figure 2	**+**
LASV	PRJNA254017	SAMN02927488	SRX718926	SRR1595500	ISTH1003	LASV347_ERCC17	Human	RNA seq	Figure 2	**+**
LASV	PRJNA254017	SAMN02927485	SRX718761	SRR1594619	ISTH0531	LASV334_ERCC136	Human	RNase H	Figure 2	**+**
LASV	PRJNA254017	SAMN02927485	SRX719205	SRR1595943	ISTH0531	LASV334_ERCC31	Human	RNA seq	Figure 2	**+**
LASV	PRJNA254017	SAMN02927498	SRX719063	SRR1595673	ISTH1121	LASV363_ERCC69	Human	RNase H	Figure 2	**+**
LASV	PRJNA254017	SAMN02927498	SRX719134	SRR1595797	ISTH1121	LASV363_ERCC43	Human	RNA seq	Figure 2	**+**
LASV	PRJNA254017	SAMN02927489	SRX719117	SRR1595763	ISTH1038	LASV349_ERCC62	Human	RNase H	Figure 2	**+**
LASV	PRJNA254017	SAMN02927489	SRX718979	SRR1595558	ISTH1038	LASV349_ERCC42	Human	RNA seq	Figure 2	**+**
LASV	PRJNA254017	SAMN02927510	SRX718802	SRR1594664	ISTH2050	LASV386_ERCC84	Human	RNase H	Figure 2	**+**
LASV	PRJNA254017	SAMN02927503	SRX719192	SRR1595909	ISTH2020	LASV368_ERCC112	Human	RNase H	Figure 2	**+**
LASV	PRJNA254017	SAMN02927484	SRX718789	SRR1594651	ISTH0230	LASV435_ERCC96	Human	RNase H	Figure 2	**+**
LASV	PRJNA254017	SAMN02927592	SRX719159	SRR1595835	LM032.dep	LM032_Depleted	Mastomys	RNase H	Figure 3	**+**
LASV	PRJNA254017	SAMN02927592	SRX718836	SRR1594698	LM032.std	LM032_Standard	Mastomys	RNA seq	Figure 3	**+**
LASV	PRJNA254017	SAMN03099734	SRX733666	SRR1613388	NHP_DK9W-AG.dep	728_Depleted	Macaque	RNase H	Figure 3	**+**
LASV	PRJNA254017	SAMN03099735	SRX733667	SRR1613389	NHP_DK9W-AG.std	728_Standard	Macaque	RNA seq	Figure 3	**+**
LASV	PRJNA254017	SAMN03099736	SRX733668	SRR1613390	NHP_DK9W-AL.dep	729_Depleted	Macaque	RNase H	Figure 3	**+**
LASV	PRJNA254017	SAMN03099737	SRX733669	SRR1613391	NHP_DK9W-AL.std	729_Standard	Macaque	RNA seq	Figure 3	**+**
LASV	PRJNA254017	SAMN03099738	SRX733670	SRR1613392	NHP_DK9W-B.dep	734_Depleted	Macaque	RNase H	Figure 3	**+**
LASV	PRJNA254017	SAMN03099739	SRX733671	SRR1613393	NHP_DK9W-B.std	734_Standard	Macaque	RNA seq	Figure 3	**+**
LASV	PRJNA254017	SAMN03099740	SRX733672	SRR1613394	NHP_DK9W-K.dep	733_Depleted	Macaque	RNase H	Figure 3	**+**
LASV	PRJNA254017	SAMN03099741	SRX733673	SRR1613395	NHP_DK9W-K.std	733_Standard	Macaque	RNA seq	Figure 3	**+**
LASV	PRJNA254017	SAMN03099742	SRX733674	SRR1613396	NHP_DK9W-L.dep	731_Depleted	Macaque	RNase H	Figure 3	**+**
LASV	PRJNA254017	SAMN03099743	SRX733675	SRR1613397	NHP_DK9W-L.std	731_Standard	Macaque	RNA seq	Figure 3	**+**
LASV	PRJNA254017	SAMN03099744	SRX733676	SRR1613398	NHP_DK9W-S.dep	732_Depleted	Macaque	RNase H	Figure 3	**+**
LASV	PRJNA254017	SAMN03099745	SRX733677	SRR1613399	NHP_DK9W-S.std	732_Standard	Macaque	RNA seq	Figure 3	**+**
LASV	PRJNA254017	SAMN02927592	SRX719168	SRR1595853	LM032	LASV68_BLC	Mastomys	RNA seq	Figure 4, Table 1	**+**
LASV	PRJNA254017	SAMN02927476	SRX727329	SRR1606288	G733	LASV_90	Human	RNA seq	Figure 4, Table 1	**+**
LASV	PRJNA254017	SAMN02927592	SRX733690	SRR1613412	LM032	LM032_HS	Mastomys	Hybrid Selection	Figure 4, Table 1	**+**
LASV	PRJNA254017	SAMN02927476	SRX733681	SRR1613403	G733	G733_HS	Human	Hybrid Selection	Figure 4, Table 1	**+**
LASV	PRJNA254017	SAMN02927593	SRX727318	SRR1606277	LM222	LASV_74	Mastomys	RNA seq	Table 1	**+**
LASV	PRJNA254017	SAMN03099732	SRX733664	SRR1613386	Z002	LASV_77	Mastomys	RNA seq	Table 1	**-**
LASV	PRJNA254017	SAMN03099733	SRX733665	SRR1613387	G090	LASV_79	Human	RNA seq	Table 1	**+**
LASV	PRJNA254017	SAMN02927477	SRX727310	SRR1606267	G771	LASV94	Human	RNA seq	Table 1	**+**
LASV	PRJNA254017	SAMN02927399	SRX734464	SRR1614275	G2230	Solexa-100929.tagged_332	Human	RNA seq	Table 1	**+**
LASV	PRJNA254017	SAMN02927483	SRX731079	SRR1610580	ISTH0073	Solexa-106870.tagged_851	Human	RNA seq	Table 1	**+**
LASV	PRJNA254017	SAMN02927500	SRX719163	SRR1595846	ISTH1137	LASV353_BLC	Human	RNA seq	Table 1	**+**
LASV	PRJNA254017	SAMN02927503	SRX718749	SRR1594606	ISTH2020	LASV368_ERCC03	Human	RNA seq	Table 1	**+**
LASV	PRJNA254017	SAMN02927504	SRX727274	SRR1606236	ISTH2025	LASV374_ERCC58	Human	RNA seq	Table 1	**+**
LASV	PRJNA254017	SAMN02927510	SRX718860	SRR1594723	ISTH2050	LASV386_ERCC48	Human	RNA seq	Table 1	**+**
LASV	PRJNA254017	SAMN02927484	SRX718809	SRR1594671	ISTH0230	LASV435_ERCC53	Human	RNA seq	Table 1	**+**
LASV	PRJNA254017	SAMN02927593	SRX733692	SRR1613414	LM222	LM222_HS	Mastomys	Hybrid Selection	Table 1	**+**
LASV	PRJNA254017	SAMN03099732	SRX733678	SRR1613400	Z002	Z002_HS	Mastomys	Hybrid Selection	Table 1	**+**
LASV	PRJNA254017	SAMN03099733	SRX733679	SRR1613401	G090	G090_HS	Human	Hybrid Selection	Table 1	**+**
LASV	PRJNA254017	SAMN02927477	SRX733682	SRR1613404	G771	G771_HS	Human	Hybrid Selection	Table 1	**+**
LASV	PRJNA254017	SAMN02927399	SRX733680	SRR1613402	G2230	G2230_HS	Human	Hybrid Selection	Table 1	**+**
LASV	PRJNA254017	SAMN02927483	SRX733683	SRR1613405	ISTH0073	ISTH0073_HS	Human	Hybrid Selection	Table 1	**+**
LASV	PRJNA254017	SAMN02927500	SRX733685	SRR1613407	ISTH1137	ISTH1137_HS	Human	Hybrid Selection	Table 1	**+**
LASV	PRJNA254017	SAMN02927503	SRX733686	SRR1613408	ISTH2020	ISTH2020_HS	Human	Hybrid Selection	Table 1	**+**
LASV	PRJNA254017	SAMN02927504	SRX733687	SRR1613409	ISTH2025	ISTH2025_HS	Human	Hybrid Selection	Table 1	**+**
LASV	PRJNA254017	SAMN02927510	SRX733688	SRR1613410	ISTH2050	ISTH2050_HS	Human	Hybrid Selection	Table 1	**+**
LASV	PRJNA254017	SAMN02927484	SRX733684	SRR1613406	ISTH0230	ISTH0230_HS	Human	Hybrid Selection	Table 1	**+**
LASV	PRJNA254017	SAMN02927592	SRX733689	SRR1613411	LM032	LM032_Depleted	Mastomys	cDNA	ND, manually added to the original sup file 3	**+**
LASV	PRJNA254017	SAMN02927592	SRX733691	SRR1613413	LM032	LM032_Standard	Mastomys	cDNA	ND, manually added to the original sup file 3	**-**

The results of both analyses are summarized in Table 4. Metavisitor was able to detect Ebola virus in all corresponding sequence datasets (S16 File) as well as Lassa virus in 53 out the 55 sequence datasets generated from Lassa virus samples (S17 File). Consistently, Matranga *et al* did not report reconstructed Lassa genomic segments from the two remaining datasets, which likely reflects high read duplication levels in the corresponding libraries [31]. The reconstructed Lassa virus L segments and Ebola virus genomes are compiled in S18 and S19 Files, respectively. In these sequences, *de novo* assembled segments in uppercases are integrated in the reference guide sequence (lowercase) used for the reconstruction. To note, for viruses with segmented genomes such as Lassa virus, the workflow has to be used separately with appropriate guide sequences for the segment to be reconstructed. As an example, we used this workflow with the filter term “Lassa” for the “Parse blast output and compile hits” tool and the Lassa S segmentNC_004296.1 for guiding the reconstruction (S20 File).

At this stage, users can use the genomic fasta sequences for further analyses. For instance, multiple sequence alignments can be performed for phylogenetics or variant analyses, or reads in the original datasets can be realigned to the viral genomes to visualize their coverages, as has been done in Use Cases 1 and 2.

## Discussion

Metavisitor performs *de novo* assembly of sequencing reads and detects contigs of viral origin through blast alignments, which then can be clustered to reconstruct a viral genome.

On the one hand, this strategy reduces the rate of false positives since the ability to form contigs that align to known viral sequences is a strong evidence of the presence of a full viral genome in the analyzed datasets. In addition, we advise Metavisitor users to remove sequence reads that align to genomes of hosts, symbionts or parasites, if these are known and available (see Fig 1). Although this treatment can be skipped (as in Use Case 3–3), it avoids chimeric assemblies of viral and nonviral sequences, while speeding up the assembly of contigs of potential viral origin. It also ensures that sequences of the host genome that have been annotated as Endogenous Viral Elements (EVEs) are not retained for viral contigs assembly. Users should keep in mind that EVEs that have not yet be identified as such may be retained by Metavisitor as potential viral contigs. Should this happen, Webster et al. [13] have demonstrated that, when available, mapping of host genome sequencing reads to these contigs allows to discriminate between EVE and virus sequences. As illustrated in Use Case 2–1, when a host is known for having antiviral RNAi pathways, re-mapping small RNA reads and plotting their length distribution can also add support to the infectious origin of candidate viral contigs (21nt read peak, sense and antisense reads aligning along the contig).

On the other hand, Metavisitor workflows may fail to assemble viral contigs when the abundance of viral reads is too low in sequenced samples, or when these reads align to short, scattered regions of viral genomes. However, as illustrated in the Use Case 3–2, it is possible to keep track of these false negatives by implementing a workflow that annotates and counts viral reads before the *de novo* contig assembly steps.

We developed Metavisitor in Galaxy in order to benefit from a well supported framework allowing execution of computational tools and workflows through a user-friendly web interface. In addition, the advanced Galaxy functionalities ensure the highest levels of computational analyses, through rigorous recording of the produced data and metadata and of the used parameters as well as the ability to share, publish and reproduce these analyses (see Metavisitor availability section in Experimental Procedures). Another major benefit is that, as any Galaxy workflow, the Metavisitor workflows may be modified or extended by users. If they are already available in a Galaxy tool shed, integration of new tools in a workflow is straightforward, thanks to the Galaxy workflow editor. Although it requires coding skills, any other freely available software can be adapted to the Galaxy framework and used in a Metavisitor workflow.

Through use cases, we have shown that Metavisitor is adaptable: short or longer reads from small RNAseq, RNAseq or DNAseq can be used as input data with or without adapter clipping; read datasets can be used as is, or compressed using reads-to-sequences or normalization by median procedures [27]; a variety of alignment and *de novo* assembly tools can be used, provided that they have been adapted for their execution in the Galaxy framework; finally, although we provide the vir1 nucleotide and protein references to identify sequences of viral origin users are free to upload and work with their own viral references. Thus, Metavisitor provides biologists and clinicians with an accessible framework for detection, reconstruction or discovery of viruses.

Viral sequences reconstructed by Metavisitor can be used in a large range of subsequent analyses, including phylogenetic or genetic drift analyses in contexts of epidemics or virus surveillance in field insect vectors, animal or human populations, or systematic identification of viruses for evaluation of their morbidity. In Use Cases 3–1 to 3–3, we have shown that Metavisitor allows analysis of numerous datasets in batch with consistent tracking of individual samples. Thus, we are confident that Metavisitor is scalable to large epidemiological studies or to clinical diagnosis in hospital environments. For instance, it could be used to analyse RNAseq data from Zika infected patients [32,33]. Finally, we wish to stress that Metavisitor has the potential for integrating detection or diagnosis of non-viral, microbial components in biological samples. Eukaryotic parasites or symbionts and bacteria are mostly detectable in sequencing datasets from their abundant ribosomal RNAs whose sequences are strongly conserved in the main kingdoms. This raises specific issues for their accurate identification and their taxonomic resolution that are not currently addressed by Metavisitor. However, many tools and databases [34] addressing these metagenomics challenges can be adapted, when not already, to the Galaxy framework. For instance, Qiime [35] and the SILVA database of ribosomal RNAs [36] can be used within Galaxy and could thus be integrated in future Metavisitor workflows aiming at detection and discovery of non virus organisms in deep sequence datasets.

## Supporting Information

S1 Fig Screenshot of the “Retrieve FASTA from NCBI” tool form to retrieve viral nucleotide (A) or protein (B) vir1 sequences. The query string “txid10239[orgn] NOT txid131567[orgn] NOT phage” retrieves viruses sequences (txid10239) while filtering out cellular organisms sequences (txid131567) and phage sequences.
(PDF)

S2 Fig Screenshot of an output produced by the “Parse blast output and compile hits” Metavisitor tool. (PDF)

S3 Fig Screenshot of Metavisitor workflow for Use Case 1-1. (PDF)

S4 Fig Screenshot of Metavisitor workflow for Use Case 1–2. (PDF)

S5 Fig Screenshot of Metavisitor workflow for Use Case 1–3. (PDF)

S6 Fig Screenshot of Metavisitor workflow for remapping for use cases 1–1, 1–2, 1–3. (PDF)

S7 Fig Screenshot of Metavisitor workflow for Use Case 1–4. (PDF)

S8 Fig Screenshot of Metavisitor workflow for Use Case 2–1. (PDF)

S9 Fig Alignments of small RNA sequence reads to the partially reconstructed Anopheles C virus genome (Use Case 2–1). Plot shows the abundance of 18–30-nucleotide (nt) small RNA sequence reads matching the genome sequences and histogram shows length distributions of these reads. Positive and negative values correspond to sense and antisense reads, respectively.
(PDF)

S10 Fig Screenshot of Metavisitor workflow for small RNA profiling of contigs. (PDF)

S11 Fig Screenshot of Metavisitor workflow for Use Case 2–2. (PDF)

S12 Fig Screenshot of Metavisitor workflow for remapping in use cases 2–1 and 2–2. (PDF)

S13 Fig Screenshot of Metavisitor workflow for Trinity test in Use Case 2–2. (PDF)

S14 Fig Screenshot of Metavisitor workflow for SPAdes test in Use Case 2–2. (PDF)

S15 Fig Screenshot of Metavisitor workflow for Use Case 3–1. (PDF)

S16 Fig Screenshot of Metavisitor workflow for Use Case 3–2. (PDF)

S17 Fig Screenshot of Metavisitor workflow for Use Case 3–3. (PDF)

S1 File Nora_MV sequence of Nora virus reconstructed by Metavisitor using reads collapsed to unique sequences (Use Case 1–1). (TXT)

S2 File Nora_raw_reads sequence of Nora virus reconstructed by Metavisitor using raw reads (Use Case 1–2). (TXT)

S3 File Nora_Median-Norm-reads sequence of Nora virus reconstructed by Metavisitor using normalisation of read abundance by median procedure (Use Case 1–3). (TXT)

S4 File MAFFT (http://www.ebi.ac.uk/Tools/msa/mafft/) Multiple Alignment of the Nora virus genome sequences published (JX220408.1 and NC_007919.3) or generated in Use Cases 1-1 to 1-3 (Nora_MV, Nora_raw_reads and Nora_Median−Norm−reads). A view of the alignments was produced by MView (http://www.ebi.ac.uk/Tools/msa/mview/). The html file can be visualized by opening it locally with a web browser.
(HTML)

S5 File Output of the “parse blast output and compile hits” tool in Use Case 1–4. (TXT)

S6 File Output of the “parse blast output and compile hits” tool in Use Case 2–1. (TXT)

S7 File Output of the “Pick Fasta Sequences” tool in Use Case 2–1. (TXT)

S8 File Sequences of the 4 contigs generated by the “CAP3 sequence assembly” tool in Use Case 2–1. (TXT)

S9 File Integration of the 4 assembled contigs (S8 File) in the DCV genome scaffold NC_001834.1 by the “blast_to_scaffold” tool. Lowercase correspond to NC_001834.1 sequences while uppercase correspond to contig sequences.
(TXT)

S10 File siRNA profiling of *de novo* assembled contigs in Use Case 2–1. Small RNA sequences reads were aligned to the contigs and size distribution and read maps were generated using the “Generate readmap and histograms from alignment files” tool. Plots show the map and abundance of 18–30 nt small RNA reads for indicated contigs and histograms show length distributions of these reads. Positive and negative values correspond to sense and antisense reads, respectively.
(PDF)

S11 File Parsing of blastx alignments with the “blast analysis, by subjects” tool in Use Case 2–2. (TXT)

S12 File Sequence of the 8919 nt contig in Use Case 2–2. (TXT)

S13 File MAFFT (http://www.ebi.ac.uk/Tools/msa/mafft/) Multiple Alignment in Clustal format of 3 AnCV genomes reconstructed with Oases, Trinity and SPAdes assembly programs. (TXT)

S14 File Merge of all reports generated by the “Parse blast output and compile hits” tool in Use Case 3–1. (TXT)

S15 File Merge of all reports generated by the “Parse blast output and compile hits” tool in Use Case 3–2. These reports are summarized in Table 3.
(TXT)

S16 File Merge of all reports for Ebola virus generated by the “Parse blast output and compile hits” tool in Use Case 3–3. These reports are summarized in Table 4.
(TXT)

S17 File Merge of all reports for Lassa virus generated by the “Parse blast output and compile hits” tool in Use Case 3–3. These reports are summarized in Table 4.
(TXT)

S18 File Lassa virus segment L reconstructed sequences in NC_004297.1 scaffold in Use Case 3–3. (TXT)

S19 File Ebola virus reconstructed sequences in NC_002549.1 scaffold in Use Case 3–3. (TXT)

S20 File Lassa virus segment S reconstructed sequences in NC_004296.1 scaffold in Use Case 3–3. (TXT)

S1 Table Galaxy tools used in Metavisitor. (PDF)

S2 Table Duration of execution of the Metavisitor workflows. The times given correspond to execution of the workflows on a 16-core (2GHz) machine with 96 Mo RAM, Galaxy release 16.04.
(PDF)

S3 Table Sequence-independent strategy to identify candidate viral contigs (Use Case 2–1). Set of contigs (Loci) with clear (+), unclear (?) or no siRNA signature were manually selected from S10 Fig and tested for significant blastx alignment against the vir1 index and the Non-redundant NCBI protein database (october 201).
(PDF)
