# Supplementary material for: Metavisitor, a Suite of Galaxy Tools for Simple and Rapid Detection and Discovery of Viruses in Deep Sequence Data
Source: PLoS One. 2017 Jan 3;12(1):e0168397. doi: 10.1371/journal.pone.0168397 (PMC5207757; doi:10.1371/journal.pone.0168397)

Supplementary Figure S1

A

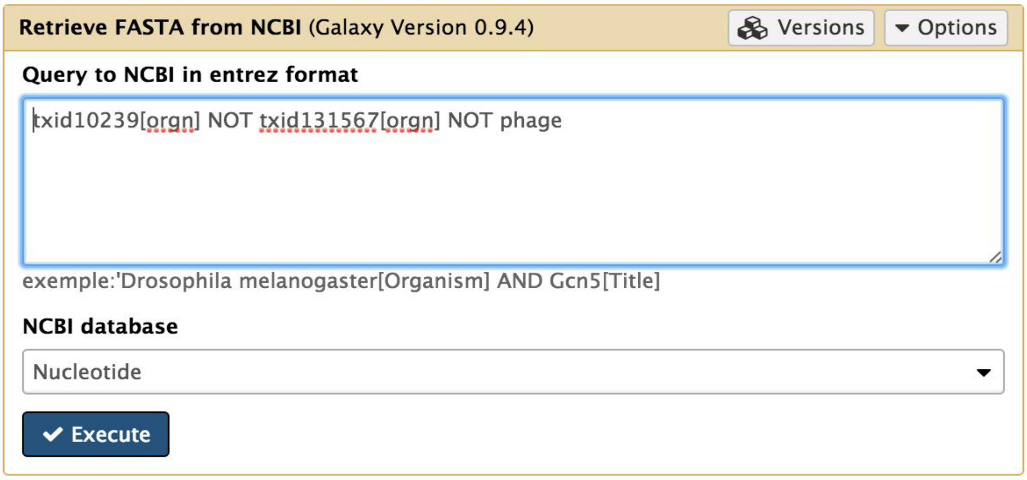

B

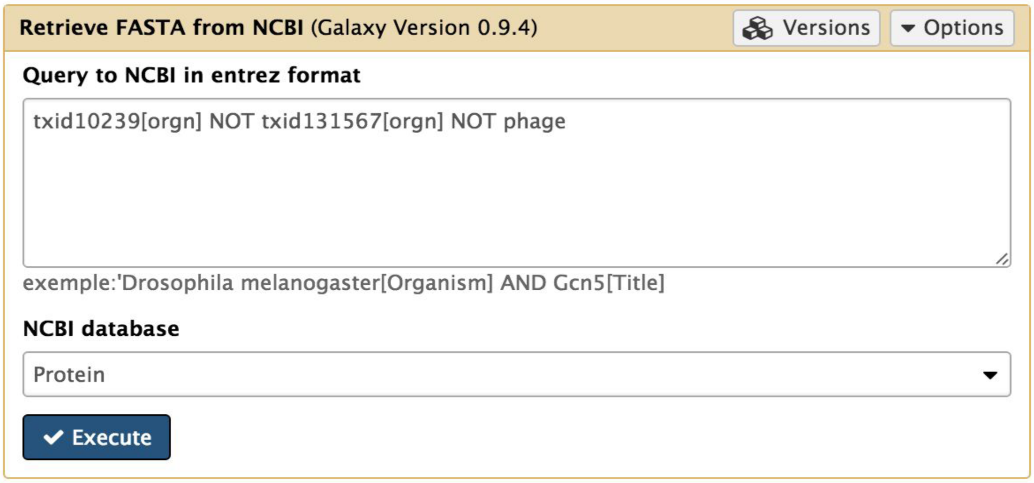

Supplement: S1 Fig — Screenshot of the “Retrieve FASTA from NCBI” tool form to retrieve viral nucleotide (A) or protein (B) vir1 sequences. The query string “txid10239[orgn] NOT txid131567[orgn] NOT phage” retrieves viruses sequences (txid10239) while filtering out cellular organisms sequences (txid131567) and phage sequences. (PDF) [file pone.0168397.s001.pdf]
