# Supplementary material for: Metavisitor, a Suite of Galaxy Tools for Simple and Rapid Detection and Discovery of Viruses in Deep Sequence Data
Source: PLoS One. 2017 Jan 3;12(1):e0168397. doi: 10.1371/journal.pone.0168397 (PMC5207757; doi:10.1371/journal.pone.0168397)
Supplement: S2 Fig — (PDF) [file pone.0168397.s002.pdf]

Supplementary Figure S2

1	2	3	4	5	6	7	8
# SeqId	%Identity	AlignLength	StartSubject	EndSubject	%QueryHitCov	E-value	BitScore
#							
# gi|157325505|gb|DQ321720.2|_Nora_virus,_complete_genome							
# Suject Length: 11908							
# Total Subject Coverage: 8338							
# Relative Subject Coverage: 0.70020154518							
# Best Bit Score: 10502.0							
# Mean Bit Score: 4066.30769231							
Locus_3_Transcript_33/33_Confidence_0.000_Length_1728	97.11	1730	11890	10161	99.9	0.0	2892.0
Locus_3_Transcript_3/33_Confidence_0.029_Length_1261	98.1	1261	6840	5580	99.9	0.0	2167.0
Locus_3_Transcript_2/33_Confidence_0.057_Length_1264	98.1	1261	6840	5580	99.7	0.0	2167.0
Locus_3_Transcript_31/33_Confidence_0.114_Length_3832	96.16	3724	11890	8167	97.1	0.0	6066.0
Locus_3_Transcript_31/33_Confidence_0.114_Length_3832	96.09	128	1807	1680	3.3	1e-50	208.0
Locus_3_Transcript_30/33_Confidence_0.171_Length_4066	96.26	4038	11890	7853	99.2	0.0	6596.0
Locus_3_Transcript_6/33_Confidence_0.057_Length_1925	97.66	1925	5605	3681	99.9	0.0	3268.0
Locus_3_Transcript_29/33_Confidence_0.171_Length_4089	96.24	4093	11890	7798	100.0	0.0	6682.0
Locus_3_Transcript_32/33_Confidence_0.057_Length_6307	96.93	6311	11890	5580	100.0	0.0	10502.0
Locus_3_Transcript_1/33_Confidence_0.057_Length_650	98.77	649	7463	6815	99.7	0.0	1135.0
Locus_3_Transcript_4/33_Confidence_0.086_Length_2045	98.18	2033	6840	4808	99.4	0.0	3499.0
Locus_3_Transcript_24/33_Confidence_0.086_Length_2244	98.22	2244	7823	5580	100.0	0.0	3867.0
Locus_3_Transcript_5/33_Confidence_0.086_Length_2212	98.24	2212	6840	4629	100.0	0.0	3813.0
#							
# gi|822478537|gb|KP970100.1|_Nora_virus_isolate_GEO58_gp1_(gp1)_gene,_partial_cds;_and_replication_p							
# Suject Length: 11416							
# Total Subject Coverage: 2463							
# Relative Subject Coverage: 0.215749824807							
# Best Bit Score: 4028.0							
# Mean Bit Score: 3607.0							
Locus_3_Transcript_22/33_Confidence_0.114_Length_2104	96.29	2105	9693	7589	100.0	0.0	3442.0
Locus_3_Transcript_23/33_Confidence_0.143_Length_2464	96.31	2463	9693	7231	99.9	0.0	4028.0
Locus_3_Transcript_20/33_Confidence_0.086_Length_2049	96.29	2050	9693	7644	100.0	0.0	3351.0
