# Supplementary figures and images for: Metavisitor, a Suite of Galaxy Tools for Simple and Rapid Detection and Discovery of Viruses in Deep Sequence Data
Source: PLoS One. 2017 Jan 3;12(1):e0168397. doi: 10.1371/journal.pone.0168397 (PMC5207757; doi:10.1371/journal.pone.0168397)

## Supplementary Figure S3

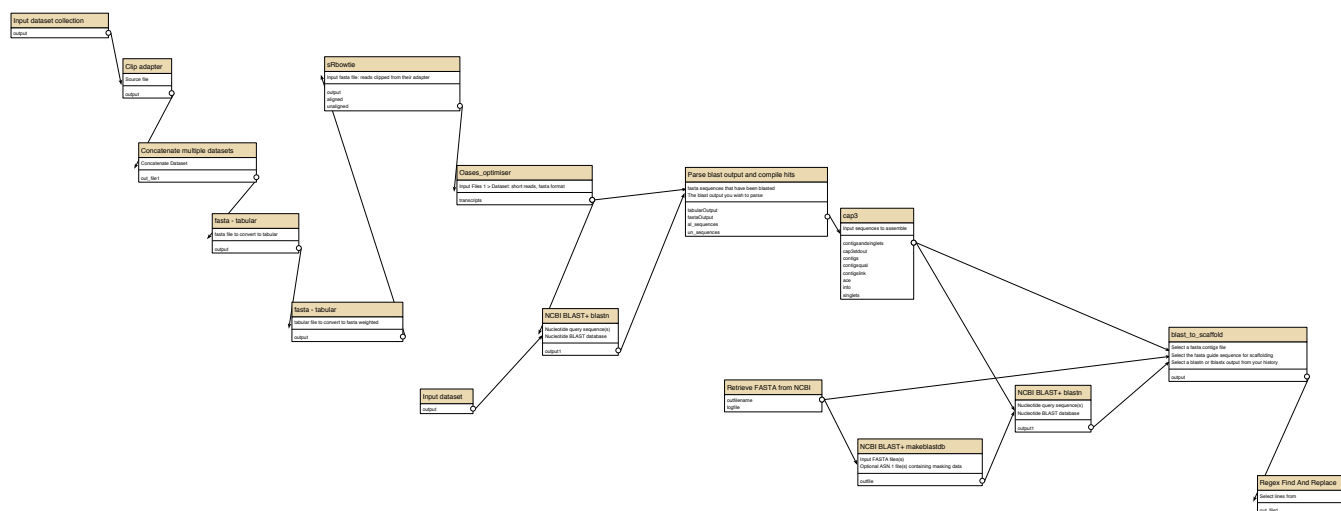

Metavisitor: Workflow for Use Case 1-1

Supplement: S3 Fig — (PDF) [file pone.0168397.s003.pdf]

Supplementary Figure S4

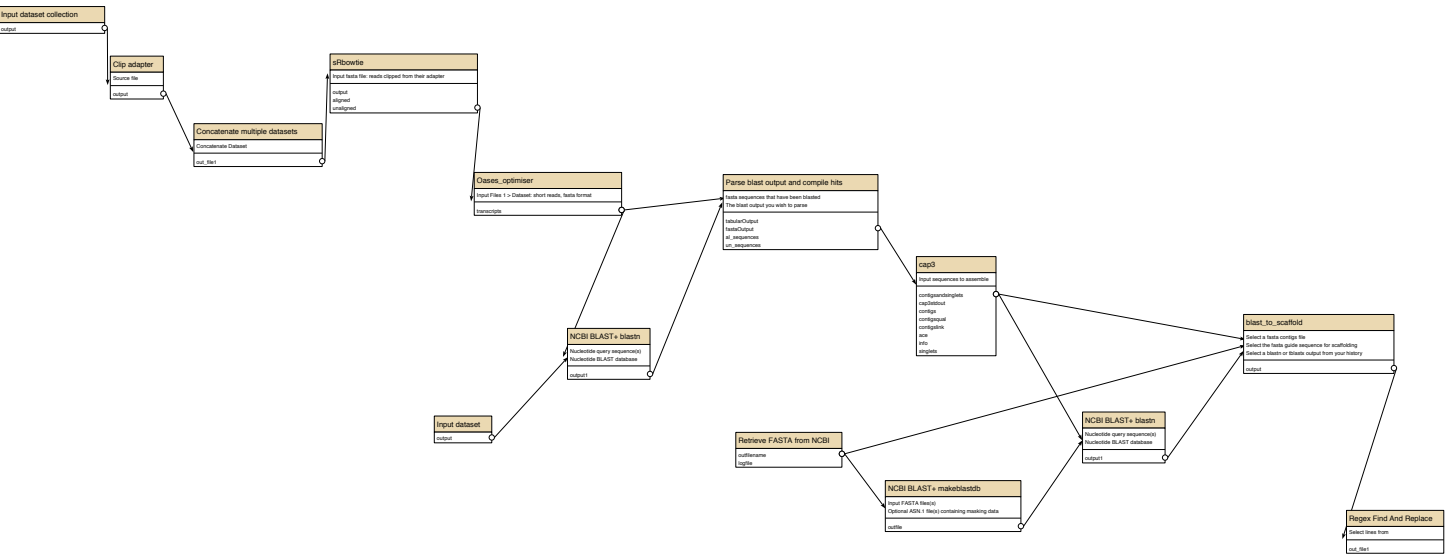

Metavisor: Workflow for Use Case 1-2

Supplement: S4 Fig — (PDF) [file pone.0168397.s004.pdf]

Supplementary Figure S5

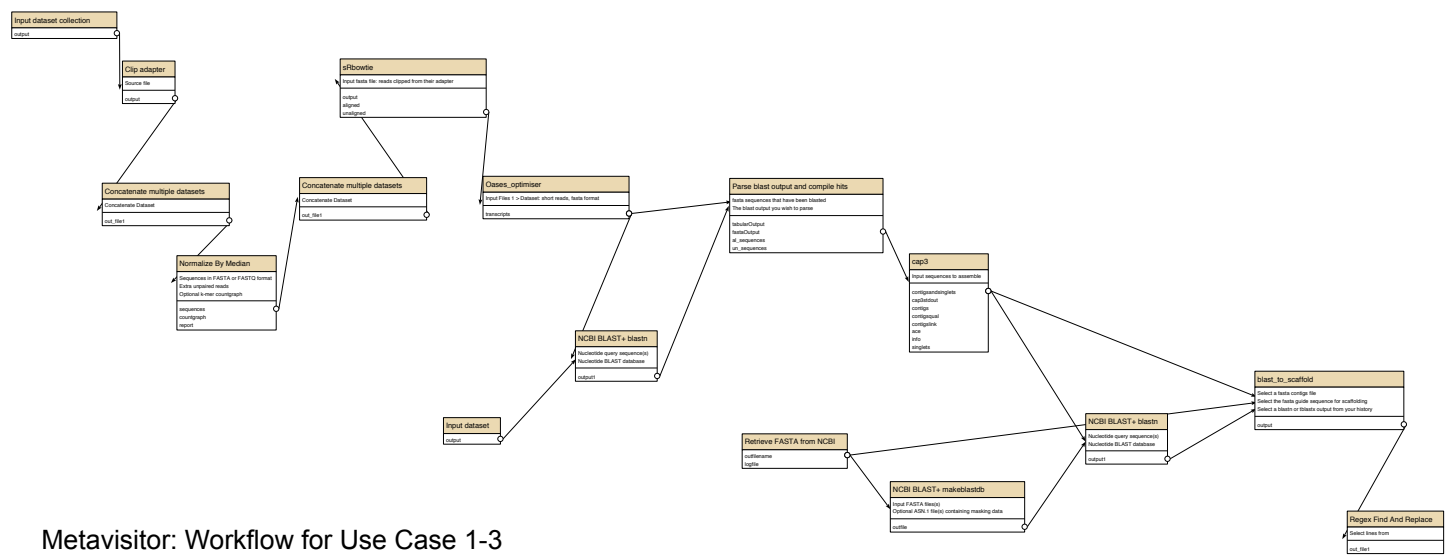

Metavisitor: Workflow for Use Case 1-3

Supplement: S5 Fig — (PDF) [file pone.0168397.s005.pdf]

Supplementary Figure S6

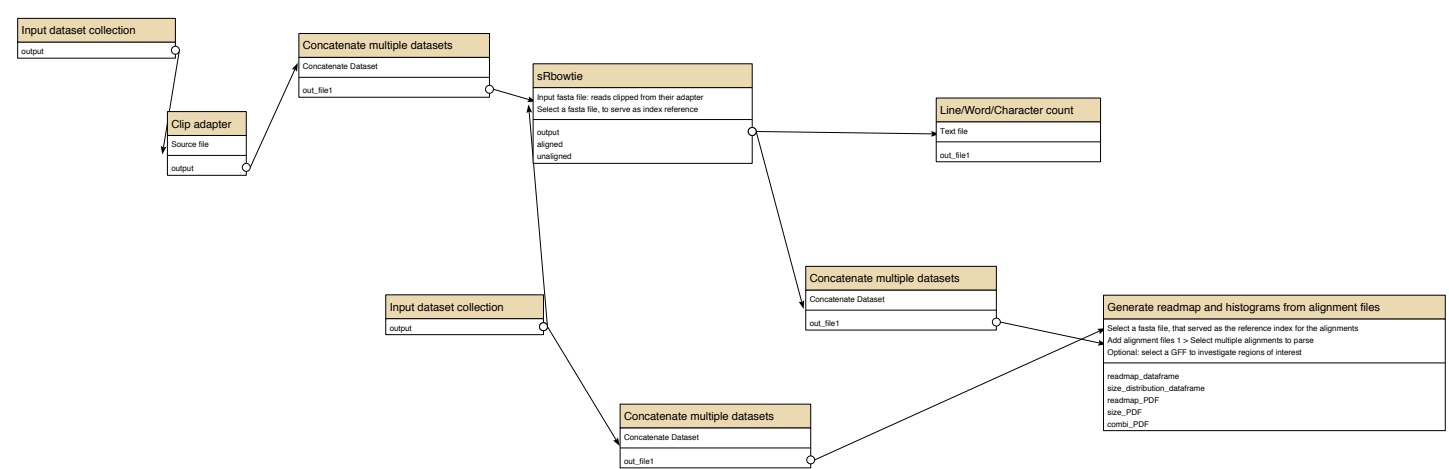

Metavisitor: Workflow for remapping in Use Cases 1-1,2,3

Supplement: S6 Fig — (PDF) [file pone.0168397.s006.pdf]

Supplementary Figure S7

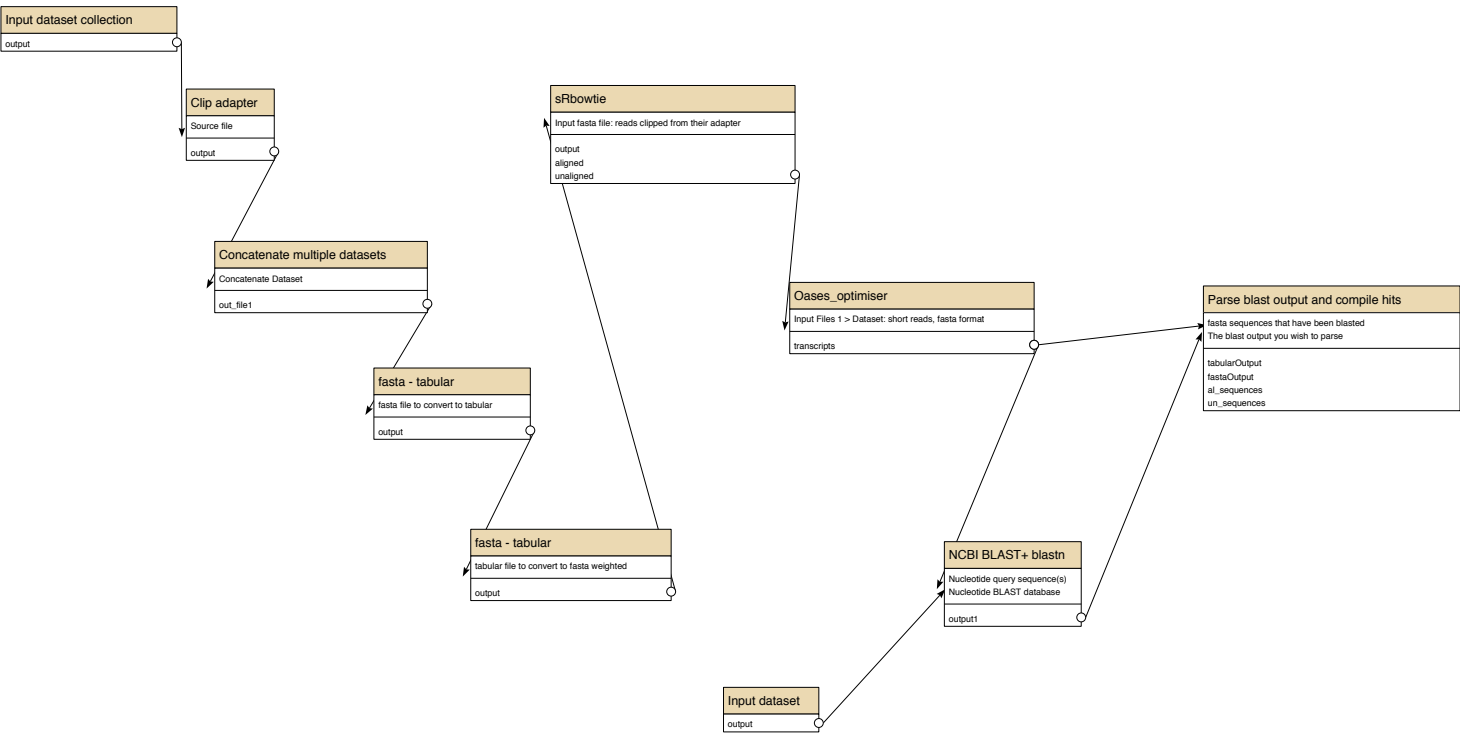

Metavisitor: Workflow for Use Case 1-4

Supplement: S7 Fig — (PDF) [file pone.0168397.s007.pdf]

## Supplementary Figure S8

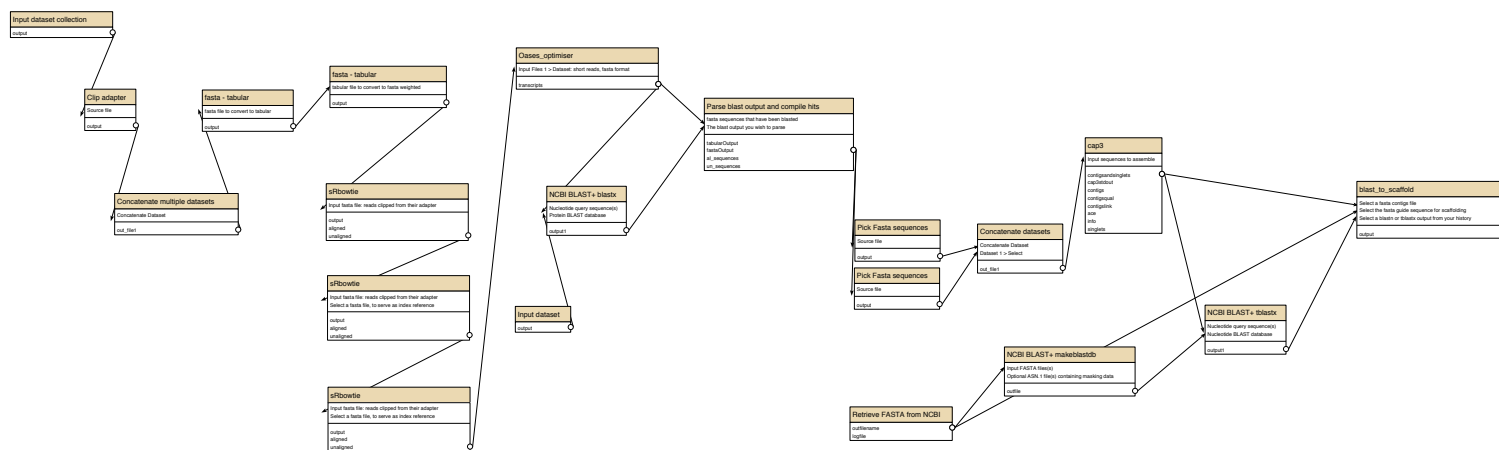

## Metavisitor: Workflow for Use Case 2-1

Supplement: S8 Fig — (PDF) [file pone.0168397.s008.pdf]

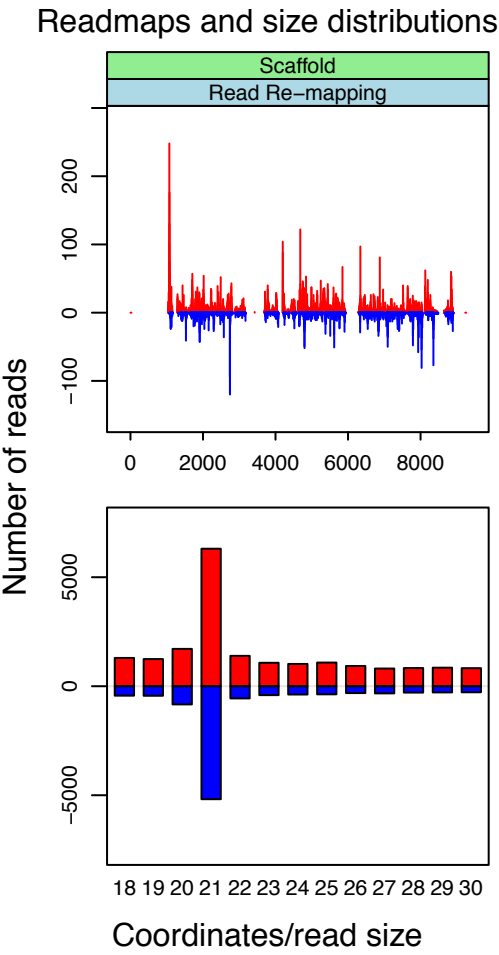

Supplement: S9 Fig — Plot shows the abundance of 18–30-nucleotide (nt) small RNA sequence reads matching the genome sequences and histogram shows length distributions of these reads. Positive and negative values correspond to sense and antisense reads, respectively. (PDF) [file pone.0168397.s009.pdf]

Supplementary Figure S10

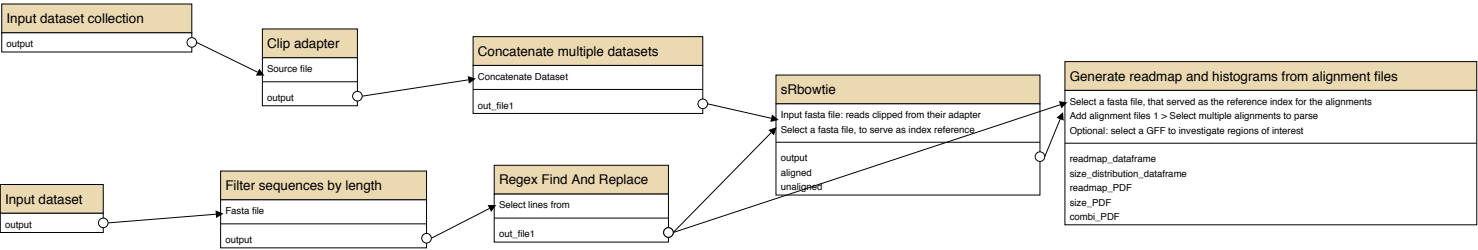

Metavisitor: Workflow for small RNA profiling of contigs

Supplement: S10 Fig — (PDF) [file pone.0168397.s010.pdf]

Supplementary Figure S11

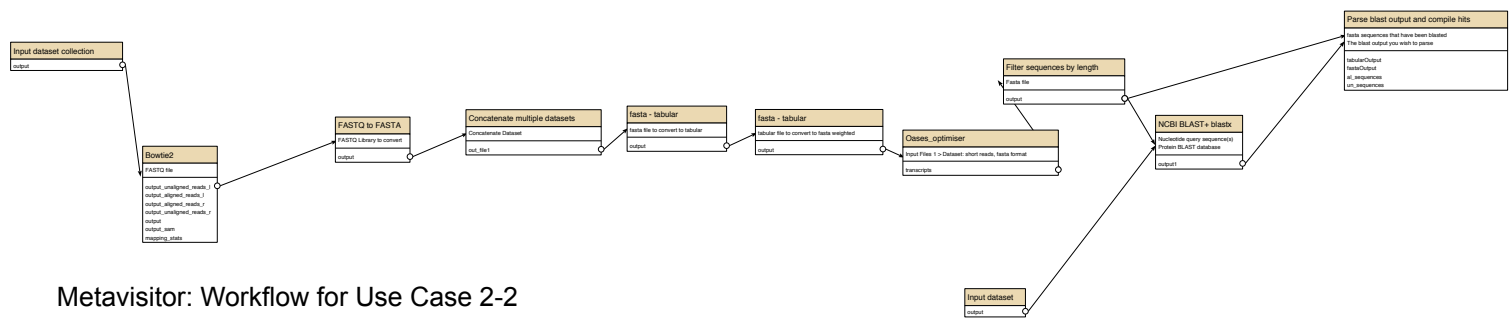

Supplement: S11 Fig — (PDF) [file pone.0168397.s011.pdf]

Supplementary Figure S12

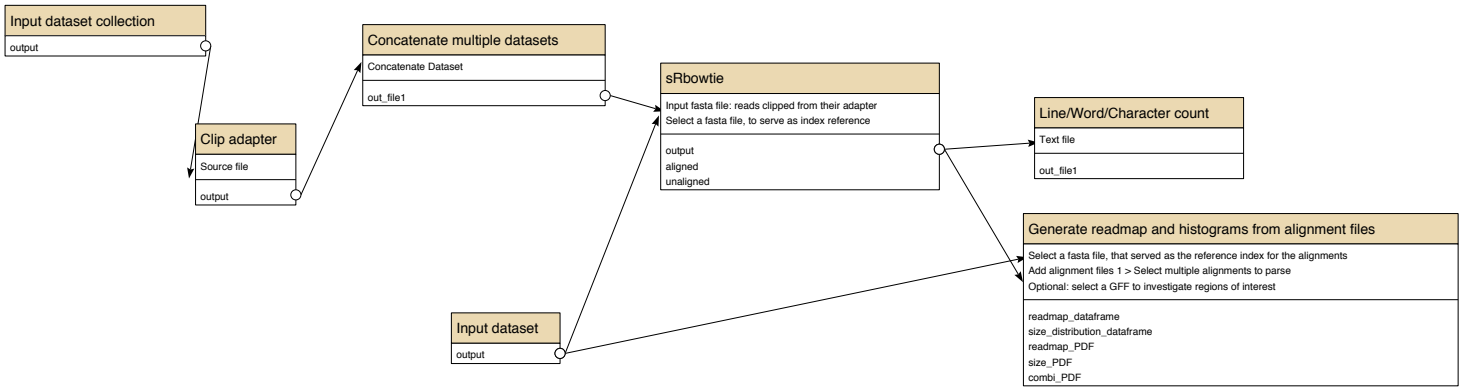

Metavisitor: Workflow for remapping in Use Cases 2-1,2

Supplement: S12 Fig — (PDF) [file pone.0168397.s012.pdf]

Supplementary Figure S13

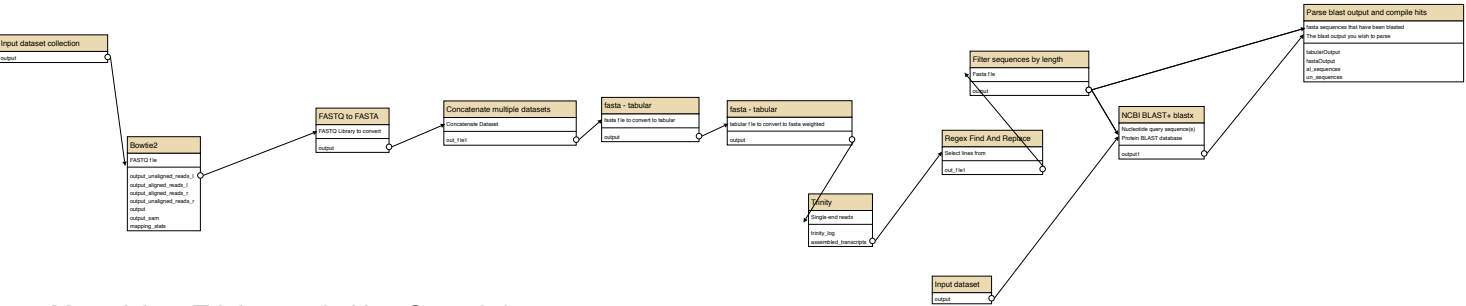

Metavisitor: Trinity test in Use Case 2-2

Supplement: S13 Fig — (PDF) [file pone.0168397.s013.pdf]

Supplementary Figure S14

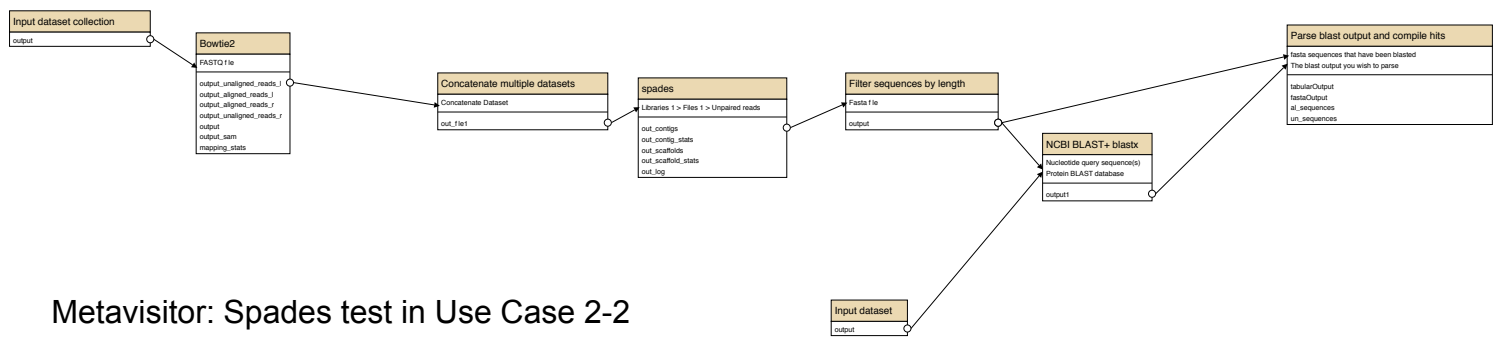

Metavisitor: Spades test in Use Case 2-2

Supplement: S14 Fig — (PDF) [file pone.0168397.s014.pdf]

Supplementary Figure S15

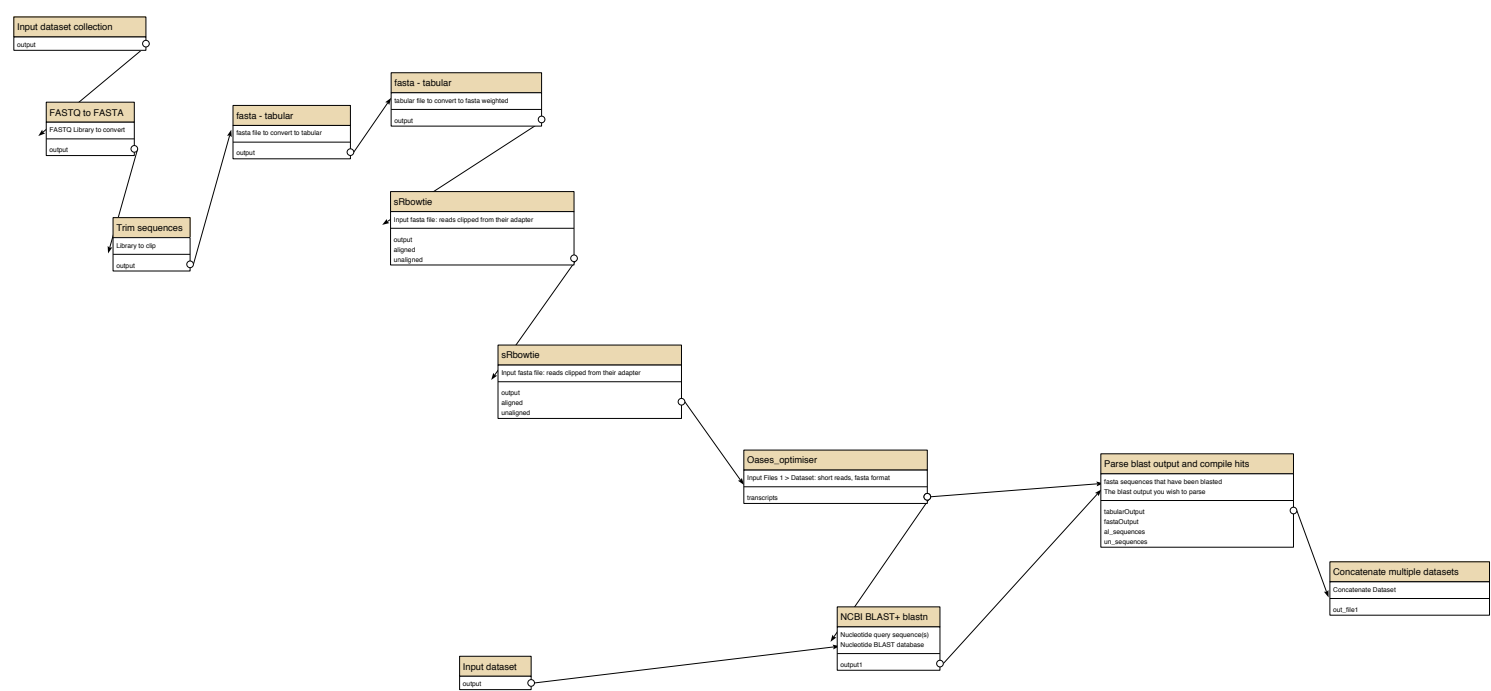

Metavisitor: Workflow for Use Case 3-1

Supplement: S15 Fig — (PDF) [file pone.0168397.s015.pdf]

Supplementary Figure S16

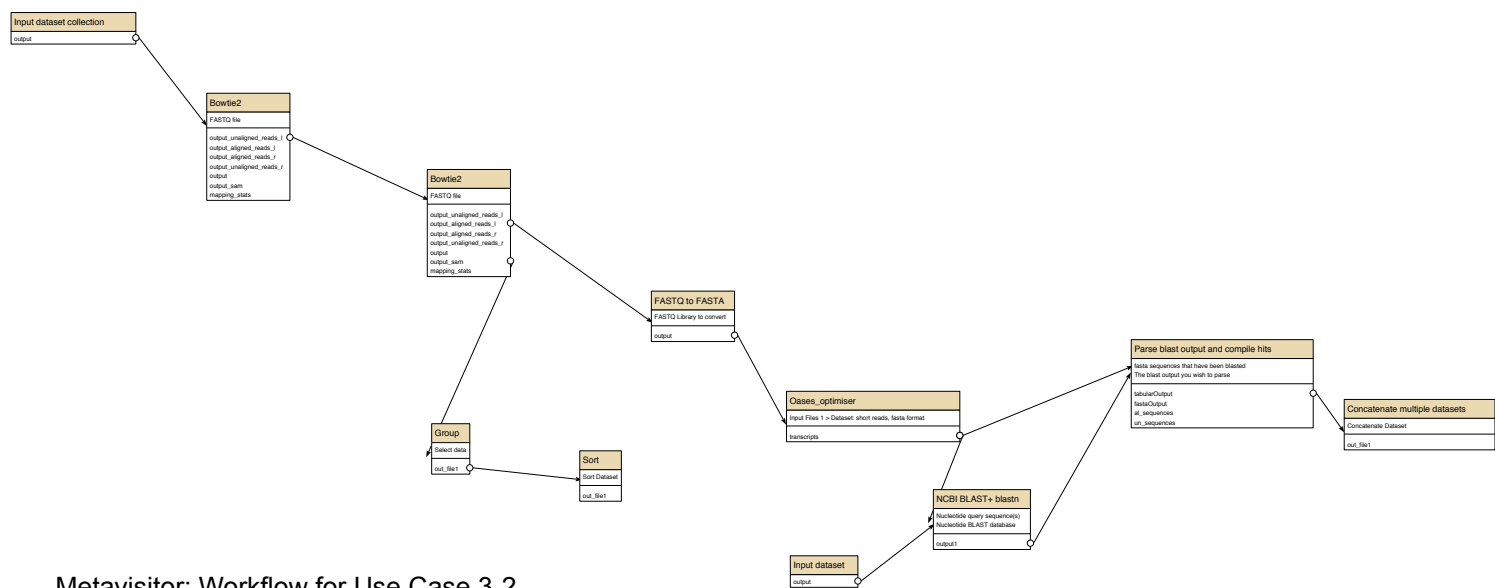

Metavisitor: Workflow for Use Case 3-2

Supplement: S16 Fig — (PDF) [file pone.0168397.s016.pdf]

Supplementary Figure S17

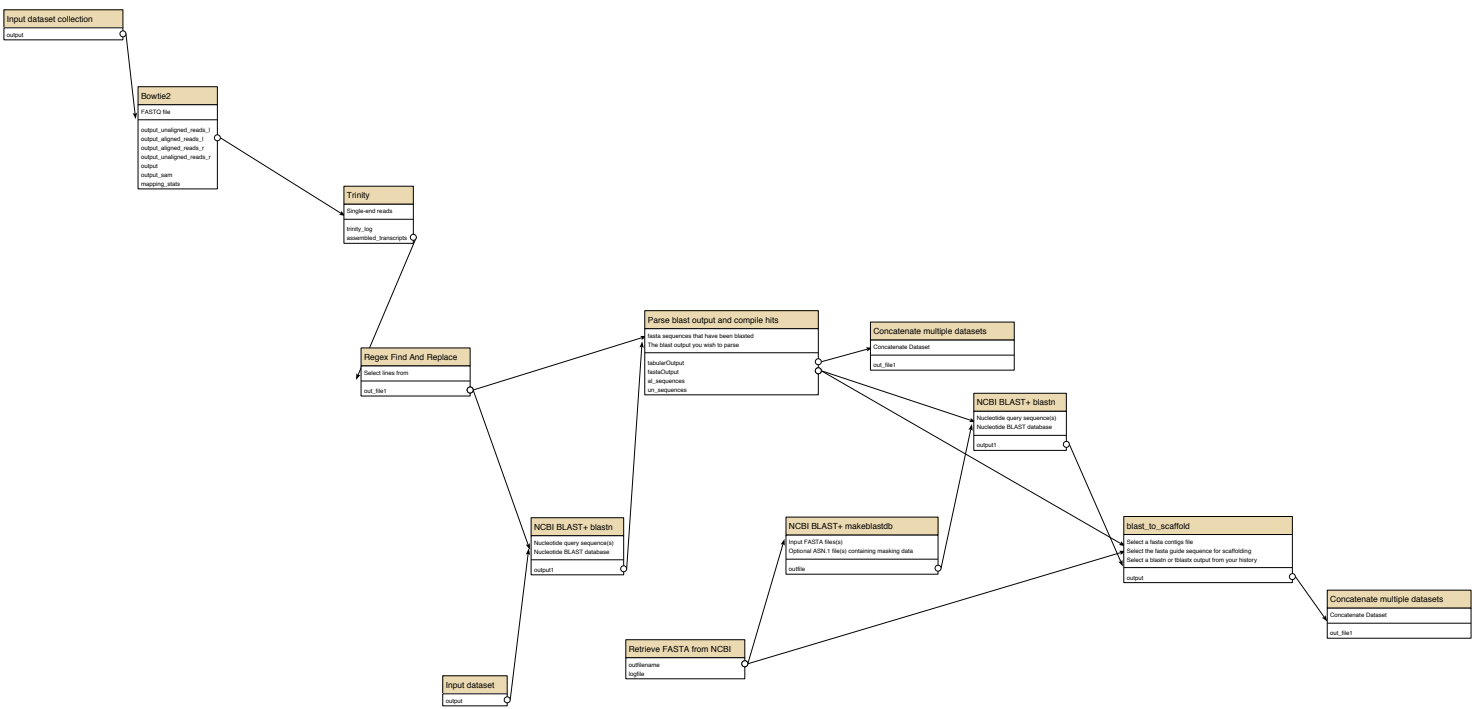

Metavisor: Workflow for Use Case 3-3

Supplement: S17 Fig — (PDF) [file pone.0168397.s017.pdf]
