# Supplementary material for: Metavisitor, a Suite of Galaxy Tools for Simple and Rapid Detection and Discovery of Viruses in Deep Sequence Data
Source: PLoS One. 2017 Jan 3;12(1):e0168397. doi: 10.1371/journal.pone.0168397 (PMC5207757; doi:10.1371/journal.pone.0168397)
Supplement: S4 File — A view of the alignments was produced by MView (http://www.ebi.ac.uk/Tools/msa/mview/). The html file can be visualized by opening it locally with a web browser. (HTML) [file pone.0168397.s021.html]

```
Reference sequence (1): NC_007919.3
Identities normalised by aligned length.
Colored by: identity + property
```

|  |
| --- |
| ```                              1 [        .         .         .         .         :         .         .         .         .         1 100   1 NC_007919.3     100.0%       GCAAAAAGGCCCCTGGGGGGGGGTTAATGAGTACTGGAAAAAGAAGCGCGAGATACCACTTCGCTGATTATGCTGATTCTGGTATTAAGGATTAGTAGAG       2 Nora_Median-Nor  96.8%       GCAAAAAGGCCCCTGGGGGGGGGTTAATGAGTACTGGAAAAAGAAGCGCGAGATACCACTTCGCTGATTATGCTGATTCTGGTATTAAGGATTAGTAGAG       3 Nora_raw_reads   96.7%       GCAAAAAGGCCCCTGGGGGGGGGTTAATGAGTACTGGAAAAAGAAGCGCGAGATACCACTTCGCTGATTATGCTGATTCTGGTATTAAGGATTAGTAGAG       4 JX220408.1       96.9%       GCAAAAAGGCCCCTGGGGGGGGGTTAATGAGTACTGGAAAAAGAAGCGCGAGATACCACTTCGCTGATTATGCTGATTCTGGTATTAAGGATTAGTAGAG       5 Nora_MV          96.6%       gcaaaaaggcccctgggggggggttaatgagTACTGGAAAAAGAAGCGCGAGATACCACTTCGCTGATTATGCTGATTCTGGTATTAAGGATTAGTAGAG                                   101          .         .         .         .         :         .         .         .         .         2 200   1 NC_007919.3     100.0%       TATCGGAAGTGCCGAAGGGAATAATGCTGGTCCGTTGTAAACAGGATACTTATGAAACCTCTTAACAGAAACTTTGAGCAAACAACAAATAAACGAACAA       2 Nora_Median-Nor  96.8%       TATCGGAAGTGCCGAAGGGAATAATGCTGGTCCGTTGTAAACAGGATACTTATGAGACCTCTTAACAGAAACTTTGAGCAAACAACAAATAAACGAACAA       3 Nora_raw_reads   96.7%       TATCGGAAGTGCCGAAGGGAATAATGCTGGTCCGTTGTAAACAGGATACTTATGAGACCTCTTAACAGAAACTTTGAGCAAACAACAAATAAACGAACAA       4 JX220408.1       96.9%       TATCGGAAGTGCCGAAGGGAATAATGCTGGTCCGTTGTAAACAGGATACTTATGAGACCTCTTAACAGAAACTTTGAGCAAACAACAAATAAACGAACAA       5 Nora_MV          96.6%       TATCGGAAGTGCCGAAGGGAATAATGCTGGTCCGTTGTAAACAGGATACTTATGAGACCTCTTAACAGAAACTTTGAGCAAACAACAAATAAACGAACAA                                   201          .         .         .         .         :         .         .         .         .         3 300   1 NC_007919.3     100.0%       AAAGAGTTATATATTGAACTTG--AAAAATCAATATCTTTTATTTAGATTGATTTATATTAGTCTTTATAATCGTAGGGAATAATTCTCATCTAATGATA       2 Nora_Median-Nor  96.8%       AAAGAGTTATACATTGAACTTGGAAAAAATCAATGTC--TTATTTAGACTGATTTATATTGGTCTTTATAATCGTAAGGAATAATTCTCATCTAATGATA       3 Nora_raw_reads   96.7%       AAAGAGTTATACATTGAACTTG--AAAAATCAATGTCTTTTATTTAGACTGATTTATATTGGTCTTTATAATCGTAAGGAATAATTCTCATCTAATGATA       4 JX220408.1       96.9%       AAAGAGTTATACATTGAACTTG--AAAAATCAATGTCTTTTATTTAGACTGATTTATATTGGTCTTTATAATCGTAAGGAATAATTCTCATCTAATGATA       5 Nora_MV          96.6%       AAAGAGTTATACATTGAACTTGAAAAAAATCAATGTCTTTTATTTAGACTGATTTATATTGGTCTTTATAATCGTAAGGAATAATTCTCATCTAATGATA                                   301          .         .         .         .         :         .         .         .         .         4 400   1 NC_007919.3     100.0%       TCGATGTAAAATGTATCATTCTAGCTATTCAATTATATTAATTGAGCTTAGCATCTGATTAAATTACTATTGTAAAATAATATTTAATCCCCATCTTTGC       2 Nora_Median-Nor  96.8%       TCGATGTAAAATGTATCATTCTAGCTATTCAATTATATTAATTGAGCTTAGCATCTGATTAAATTAcTATTATAAAATAATATTTAATCCCCATCTTTGC       3 Nora_raw_reads   96.7%       TCGATGTAAAATGTATCATTCTAGCTATTCAATTATATTAATTGAGCTTAGCATCTGATTAAATTATTATTATAAAATAATATTTAATCCCCATCTTTGC       4 JX220408.1       96.9%       TCGATGTAAAATGTATCATTCTAGCTATTCAATTATATTAATTGAGCTTAGCATCTGATTAAATTATTATTATAAAATAATATTTAATCCCCATCTTTGC       5 Nora_MV          96.6%       TCGATGTAAAATGTATCATTCTAGCTATTCAATTATATTAATTGAGCTTAGCATCTGATTAAAT--cTATTATAAAATAATATTTAATCCCCATCTTTGC                                   401          .         .         .         .         :         .         .         .         .         5 500   1 NC_007919.3     100.0%       AAGATGGAAACTCTTAATAAGTCAGGAGTATAATGGATTTAAACCCTCACACCGAATCATGAGTATAAACAGATTCAAATATGATTAACAATCAAACAAA       2 Nora_Median-Nor  96.8%       AAGATGGAAACTCTCAATAAATCAGGAGTATAATAGATTTAAACCCTCACACTGAATCACGAGTATAAACAGATTCAAATATGATTAACAATCAAACAAA       3 Nora_raw_reads   96.7%       AAGATGGAAACTCTCAATAAATCAGGAGTATAATAGATTTAAACCCTCACACTGAATCACGAGTATAAACAGATTCAAATATGATTAACAATCAAACAAA       4 JX220408.1       96.9%       AAGATGGAAACTCTCAATAAATCAGGAGTATAATAGATTTAAACCCTCACACTGAATCACGAGTATAAACAGATTCAAATATGATTAACAATCAAACAAA       5 Nora_MV          96.6%       AAGATGGAAACTCTCAATAAATCAGGAGTATAATAGATTTAAACCCTCACACTGAATCACGAGTATAAACAGATTCAAATATGATTAACAATCAAACAAA                                   501          .         .         .         .         :         .         .         .         .         6 600   1 NC_007919.3     100.0%       CAAAAAGGGACCACAACTAGAGAGAGTACATTTTGGTAGTACGCAGGTTGTGGGAAAGAGTACCAAACGACGACAACGCGGAACTAAACTTGACATTGAA       2 Nora_Median-Nor  96.8%       CAAAAAGGGACCACAACTAGAGAGAGTACATTTTGGTAGTGCGCAGGTTGTGGGAAAAAGTACCAAACGACGACAACGCGGAACCAAATTTGACATTGAA       3 Nora_raw_reads   96.7%       CAAAAAGGGACCACAACTAGAGAGAGTACATTTTGGTAGTGCGCAGGTTGTGGGAAAAAGTACCAAACGACGACAACGCGGAACCAAATTTGACATTGAA       4 JX220408.1       96.9%       CAAAAAGGGACCACAACTAGAGAGAGTACATTTTGGTAGTGCGCAGGTTGTGGGAAAAAGTACCAAACGACGACAACGCGGAACCAAATTTGACATTGAA       5 Nora_MV          96.6%       CAAAAAGGGACCACAACTAGAGAGAGTACATTTTGGTAGTGCGCAGGTTGTGGGAAAAAGTACCAAACGACGACAACGCGGAACCAAATTTGACATTGAA                                   601          .         .         .         .         :         .         .         .         .         7 700   1 NC_007919.3     100.0%       TATACTGTTAGAAGGAACGATGCACCAAAAGAGCAGAAATTCTTGATTTCAGAAATTTTTGATGAAAAGTTGGATAAACAAATAAAATATGAGAAGAAGC       2 Nora_Median-Nor  96.8%       TATACTGTTAAAAGGAACGATGCACCAAAAGAGCAGAAATTCTTAGTTTCAGAAATTTTTGATGAAAAGTTGGATAAACAAATAAAATATGAGAAGAAGC       3 Nora_raw_reads   96.7%       TATACTGTTAAAAGGAACGATGCACCAAAAGAGCAGAAATTCTTAGTTTCAGAAATTTTTGATGAAAAGTTGGATAAACAAATAAAATATGAGAAGAAGC       4 JX220408.1       96.9%       TATACTGTTAAAAGGAACGATGCACCAAAAGAGCAGAAATTCTTAGTTTCAGAAATTTTTGATGAAAAGTTGGATAAACAAATAAAATATGAGAAGAAGC       5 Nora_MV          96.6%       TATACTGTTAAAAGGAACGATGCACCAAAAGAGCAGAAATTCTTAGTTTCAGAAATTTTTGATGAAAAGTTGGATAAACAAATAAAATATGAGAAGAAGC                                   701          .         .         .         .         :         .         .         .         .         8 800   1 NC_007919.3     100.0%       AAAACCATACTTTTATTAAACCGAAATTGAATCTAGTTATTAAAGAAGAACAACATATAACTAAGAAGGTTTTAAGAGGTAAGGAACGAGCTGCAACTCA       2 Nora_Median-Nor  96.8%       AAAATCATACTTTTATTAAACCGAAATTAAATTTAGTTACTAGAGAAGAACAACACGTGACTAAGAAGGTTTTAAGAGGTAAAGAACGAGCTGCAACTCA       3 Nora_raw_reads   96.7%       AAAATCATACTTTTATTAAACCGAAATTAAATTTAGTTACTAGAGAAGAACAACACGTGACTAAGAAGGTTTTAAGAGGTAAAGAACGAGCTGCAACTCA       4 JX220408.1       96.9%       AAAATCATACTTTTATTAAACCGAAATTAAATTTAGTTACTAGAGAAGAACAACACGTGACTAAGAAGGTTTTAAGAGGTAAAGAACGAGCTGCAACTCA       5 Nora_MV          96.6%       AAAATCATACTTTTATTAAACCGAAATTAAATTTAGTTACTAGAGAAGAACAACACGTGACTAAGAAGGTTTTAAGAGGTAAAGAACGAGCTGCAACTCA                                   801          .         .         .         .         :         .         .         .         .         9 900   1 NC_007919.3     100.0%       TGCTTTTATGAAGGAAATGGTTGAATCTAACAAGATACAACCTAGTTGGAATGTTGAATACGAAAAAGAAATAGATGAGGTTGATCTATTTTTTATGAAG       2 Nora_Median-Nor  96.8%       TGCTTTTATGAAGGAAATGGTTGAATCTAACAAGATACAACCTAGTTGGAATGTCGAATACGAAAAAGAAATAGATGAGGTTGATCTATTTTTTATGAAG       3 Nora_raw_reads   96.7%       TGCTTTTATGAAGGAAATGGTTGAATCTAACAAGATACAACCTAGTTGGAATGTCGAATACGAAAAAGAAATAGATGAGGTTGATCTATTTTTTATGAAG       4 JX220408.1       96.9%       TGCTTTTATGAAGGAAATGGTTGAATCTAACAAGATACAACCTAGTTGGAATGTCGAATACGAAAAAGAAATAGATGAGGTTGATCTATTTTTTATGAAG       5 Nora_MV          96.6%       TGCTTTTATGAAGGAAATGGTTGAATCTAACAAGATACAACCTAGTTGGAATGTCGAATACGAAAAAGAAATAGATGAGGTTGATCTATTTTTTATGAAG                                   901          .         .         .         .         :         .         .         .         .         0 1000  1 NC_007919.3     100.0%       AAGAAAACTAAGCCTTTCTCAGGTTTTTCTATTAAGGAATTAAGAGATAGTCTAATTGTGCAGTCAGACGATAAAAACATGGCACAGCCAACCGTGATGA       2 Nora_Median-Nor  96.8%       AAGAGAACCAAACCTTTCTCAGGTTTTTCTATTAAGGAATTAAGAGATAGTCTAATTGTGCAGTCAGACGATAAAAACATGGCACAGCCAACCGTGATGA       3 Nora_raw_reads   96.7%       AAGAGAACCAAACCTTTCTCAGGTTTTTCTATTAAGGAATTAAGAGATAGTCTAATTGTGCAGTCAGACGATAAAAACATGGCACAGCCAACCGTGATGA       4 JX220408.1       96.9%       AAGAAAACCAAACCTTTCTCAGGTTTTTCTATTAAGGAATTAAGAGATAGTCTAATTGTGCAGTCAGACGATAAAAACATGGCACAGCCAACCGTGATGA       5 Nora_MV          96.6%       AAGAAAACCAAACCTTTCTCAGGTTTTTCTATTAAGGAATTAAGAGATAGTCTAATTGTGCAGTCAGACGATAAAAACATGGCACAGCCAACCGTGATGA                                  1001          .         .         .         .         :         .         .         .         .         1 1100  1 NC_007919.3     100.0%       GTTCAATCGATGAAATTGTTACACCTCGTGAGGAGATAAGCGTTTCTGCTATCTCTGAACAACTGGCATCTTTGATGGAGAGAGTTGACAAACTCGAGAA       2 Nora_Median-Nor  96.8%       GTTCAACCAATGAAATTGTTACACCCCGTGAGGAGATAAGCGTTTCTGCTATCTCTGAACAACTGGCATCCTTGATGGAGAGAGTTGATAAACTCGAGAA       3 Nora_raw_reads   96.7%       GTTCAACCAATGAAATTGTTACACCCCGTGAGGAGATAAGCGTTTCTGCTATCTCTGAACAACTGGCATCCTTGATGGAGAGAGTTGATAAACTCGAGAA       4 JX220408.1       96.9%       GTTCAACCAATGAAATTGTTACACCCCGTGAGGAGATAAGCGTTTCTGCTATCTCTGAACAACTGGCATCCTTGATGGAGAGAGTTGATAAACTCGAGAA       5 Nora_MV          96.6%       GTTCAACCAATGAAATTGTTACACCCCGTGAGGAGATAAGCGTTTCTGCTATCTCTGAACAACTGGCATCCTTGATGGAGAGAGTTGATAAACTCGAGAA                                  1101          .         .         .         .         :         .         .         .         .         2 1200  1 NC_007919.3     100.0%       GATGAATGCTGCTTTGGAAGAAGAAAACAAGCAGTTAAAGAAGGAGAGAGAAGCGACTATTAAGTCAGTTAAGAAAGAGGCAAAGAAGATTAAACAAGAG       2 Nora_Median-Nor  96.8%       GATGAATGCTGCTTTGGAGGAAGAAAACAAGCAGCTAAAGAAAGAGAGAGAGGCGACTATTAAGTCAGTTAAGAAAGAGGCAAAGAGGACTAAACAAGAG       3 Nora_raw_reads   96.7%       GATGAATGCTGTTTTGGAGGAAGAAAACAAGCAGCTAAAGAAAGAGAGAGAGGCGACTATTAAGTCAGTTAAGAAAGAGGCAAAGAGGACTAAACAAGAG       4 JX220408.1       96.9%       GATGAATGCTGCTTTGGAGGAAGAAAACAAGCAGCTAAAGAAAGAGAGAGAGGCGACTATTAAGTCAGTTAAGAAAGAGGCAAAGAGGACTAAACAAGAG       5 Nora_MV          96.6%       GATGAATGCTGCTTTGGAGGAAGAAAACAAGCAGCTAAAGAAAGAGAGAGAGGCGACTATTAAGTCAGTTAAGAAAGAGGCAAAGAGGACTAAACAAGAG                                  1201          .         .         .         .         :         .         .         .         .         3 1300  1 NC_007919.3     100.0%       AAGCCTCAGATTGTGAAGAAAACGCAGCACAAGAGTTTAGGAGTAAATCTTAAAATCACCAAGACCAAAGTAGTTGGTCAGGAACAATGTTTGGAAATTG       2 Nora_Median-Nor  96.8%       AAGCCTCAGATTGCGAAGAAAACGCAACACAAGAGTTTAGGAGTAAATCTTAAAATCACCAAGACCAAAGTAGTTGGTCAGGAACAATGTTTGGAAATTG       3 Nora_raw_reads   96.7%       AAGCCTCAGATTGCGAAGAAAACGCAACACAAGAGTTTAGGAGTAAATCTTAAAATCACCAAGACCAAAGTAGTTGGTCAGGAACAATGTTTGGAAATTG       4 JX220408.1       96.9%       AAGCCTCAGATTGCGAAGAAAACGCAACACAAGAGTTTAGGAGTAAATCTTAAAATCACCAAGACCAAAGTAGTTGGTCAGGAACAATGTTTGGAAATTG       5 Nora_MV          96.6%       AAGCCTCAGATTGCGAAGAAAACGCAACACAAGAGTTTAGGAGTAAATCTTAAAATCACCAAGACCAAAGTAGTTGGTCAGGAACAATGTTTGGAAATTG                                  1301          .         .         .         .         :         .         .         .         .         4 1400  1 NC_007919.3     100.0%       AAAACACTCAGCATAAGAAATTTGTTGAGAAGCCAAGCATGCCACTTAAAGTGAGCAAGAAGATGACGGAACACCAGTTGAAAAAGACTATTCGTACTTG       2 Nora_Median-Nor  96.8%       AAAATACTCAGCATAAGAAATTTGTTGAGAAGCCAAGCATGCCATCCAAAGTGAGCAAGAAGATGAAGGGACAACAGTTGAAAAAGACTATTCGTACTTG       3 Nora_raw_reads   96.7%       AAAATACTCAGCATAAGAAATTTGTTGAGAAGCCAAGCATGCCATCCAAAGTGAGCAAGAAGATGAAGGGACAACAGTTGAAAAAGACTATTCGTACTTG       4 JX220408.1       96.9%       AAAATACTCAGCATAAGAAATTTGTTGAGAAGCCAAGCATGCCATCCAAAGTGAGCAAGAAGATGAAGGGACAACAGTTGAAAAAGACTATTCGTACTTG       5 Nora_MV          96.6%       AAAATACTCAGCATAAGAAATTTGTTGAGAAGCCAAGCATGCCATCCAAAGTGAGCAAGAAGATGAAGGGACAACAGTTGAAAAAGACTATTCGTACTTG                                  1401          .         .         .         .         :         .         .         .         .         5 1500  1 NC_007919.3     100.0%       GTATGAATTTGATCCCTCTAAGCTCGTTCAGCATCAAAAAGAAGTGTTGAACAGTGTTGTTACTAACACAACTTTCGCAGATAAAGTCCGTGAAACTGGT       2 Nora_Median-Nor  96.8%       GTATGAATTTGATCCCTCTAAACTCGTTCAGCATCAAAAAGAAGTGTTGAACAGTGTTGTTACCAACACAACCTTCGCAGATAAAGTCCGTGAAACTGGT       3 Nora_raw_reads   96.7%       GTATGAATTTGATCCCTCTAAACTCGTTCAGCATCAAAAAGAAGTGTTGAACAGTGTTGTTACTAACACAACCTTCGCAGATAAAGTCCGTGAAACTGGT       4 JX220408.1       96.9%       GTATGAATTTGATCCCTCTAAACTCGTTCAGCATCAAAAAGAAGTGTTGAACAGTGTTGTTACTAACACAACCTTCGCAGATAAAGTCCGTGAAACTGGT       5 Nora_MV          96.6%       GTATGAATTTGATCCCTCTAAACTCGTTCAGCATCAAAAAGAAGTGTTGAACAGTGTTGTTACTAACACAACCTTCGCAGATAAAGTCCGTGAAACTGGT                                  1501          .         .         .         .         :         .         .         .         .         6 1600  1 NC_007919.3     100.0%       ATACCTAAACAAAAGATTAGGTATGTTGCAAAACCACCAGCAGAGGAGAAAAGGAGTATCCATTTTTATGGTTACAAACCAAAAGGAATCCCTAACAAAG       2 Nora_Median-Nor  96.8%       ATACCTAAACAAAAGATTAGGTATACTGCAAAACCACCAGCAGAGGAGAAGAGGAGTATCCATTTCTATGGTTATAAGCCAAAAGGAATCCCTAACAAAG       3 Nora_raw_reads   96.7%       ATACCTAAACAAAAGATTAGGTATACTGCAAAACCACCAGCAGAGGAGAAGAGGAGTATCCATTTCTATGGTTATAAGCCAAAAGGAATCCCTAACAAAG       4 JX220408.1       96.9%       ATACCTAAACAAAAGATTAGGTATACTGCAAAACCACCAGCAGAGGAGAAGAGGAGTATCCATTTCTATGGTTATAAGCCAAAAGGAATCCCTAACAAAG       5 Nora_MV          96.6%       ATACCTAAACAAAAGATTAGGTATACTGCAAAACCACCAGCAGAGGAGAAGAGGAGTATCCATTTCTATGGTTATAAGCCAAAAGGAATCCCTAACAAAG                                  1601          .         .         .         .         :         .         .         .         .         7 1700  1 NC_007919.3     100.0%       TTTGGTGGAACTGGGTCACCACTGGCACAGCTATGGACGCTTATGAAAAAGCTGACCGTTATCTGTATCATCAATTTAAACGAGAAATGATGATATACAG       2 Nora_Median-Nor  96.8%       TTTGGTGGAATTGGGTCACCACTGGCACAGCTATGGACGCTTATGAAAAAGCTGACCATTATCTGTATCACCAATTTAAACGAGAAATGATGGTATACAG       3 Nora_raw_reads   96.7%       TTTGGTGGAATTGGGTCACCACTGGCACAGCTATGGACGCTTATGAAAAAGCTGACCATTATCTGTATCACCAATTTAAACGAGAAATGATGGTATACAG       4 JX220408.1       96.9%       TTTGGTGGAATTGGGTCACCACTGGCACAGCTATGGACGCTTATGAAAAAGCTGACCATTATCTGTATCACCAATTTAAACGAGAAATGATGGTATACAG       5 Nora_MV          96.6%       TTTGGTGGAATTGGGTCACCACTGGCACAGCTATGGACGCTTATGAAAAAGCTGACCATTATCTGTATCACCAATTTAAACGAGAAATGATGGTATACAG                                  1701          .         .         .         .         :         .         .         .         .         8 1800  1 NC_007919.3     100.0%       AAATAAATGGGTCAAATTTAGTAAGGAGTTCAATCCATACCTATCAAAACCGAAAATGGTGTGGGAAGAGAATACATGGGAATATGAATATAAAACGGAC       2 Nora_Median-Nor  96.8%       AAATAAATGGGTCAAGTTTAGTAAGGAGTTCAATCCGTACCTATCGGAACCGAAAATGGTATGGGAAGAGAATACATGGGAATATGAATATAAAACAGAC       3 Nora_raw_reads   96.7%       AAATAAATGGGTCAAGTTTAGTAAGGAGTTCAATCCGTACCTATCGGAACCGAAAATGGTATGGGAAGAGAATACATGGGAATATGAATATAAAACAGAC       4 JX220408.1       96.9%       AAATAAATGGGTCAAGTTTAGTAAGGAGTTCAATCCGTACCTATCGGAACCGAAAATGGTATGGGAAGAGAATACATGGGAATATGAATATAAAACAGAC       5 Nora_MV          96.6%       AAATAAATGGGTCAAGTTTAGTAAGGAGTTCAATCCGTACCTATCGGAACCGAAAATGGTATGGGAAGAGAATACATGGGAATATGAATATAAAACAGAC                                  1801          .         .         .         .         :         .         .         .         .         9 1900  1 NC_007919.3     100.0%       GTTCCCTATAATTTTATTCTCAAATGGCGCCAGTTAGTACAGACTTACAAACCTAACACACCAATCCAGGCTGATTGGTATAAAATCTCGCAGAAACAAC       2 Nora_Median-Nor  96.8%       GTTCCCTACAATTTTATTCTCAAATGGCGCCAGTTAGTGCAGACCTACAAGCCTAACACACCAATCCAGGCTGATTGGTACAAAATCTCGCAGAAACAAC       3 Nora_raw_reads   96.7%       GTTCCCTACAATTTTATTCTCAAATGGCGCCAGTTAGTGCAGACCTACAAGCCTAACACACCAATCCAGGCTGATTGGTACAAAATCTCGCAGAAACAAC       4 JX220408.1       96.9%       GTTCCCTACAATTTTATTCTCAAATGGCGCCAGTTAGTGCAGACCTACAAGCCTAACACACCAATCCAGGCTGATTGGTACAAAATCTCGCAGAAACAAC       5 Nora_MV          96.6%       GTTCCCTACAATTTTATTCTCAAATGGCGCCAGTTAGTGCAGACCTACAAGCCTAACACACCAATCCAGGCTGATTGGTACAAAATCTCGCAGAAACAAC                                  1901          .         .         .         .         :         .         .         .         .         0 2000  1 NC_007919.3     100.0%       AATGTTAATTGAAGCTTTCATCAATTCTTTGTTGCAAAATCTAGGTATCATGATGTCTTTCCGTGACCTAGTGGCGAGCCCATGGATATTGCTAGTAATA       2 Nora_Median-Nor  96.8%       AATGTTAATTGAAGTTTTCATTAATTCTTTGTTGCAAAATCTAGGTATCATGATGTCTTTCCGTGACCTAGTGGCGAGCCCATGGATACTGCTAGTAATA       3 Nora_raw_reads   96.7%       AATGTTAATTGAAGTTTTCATTAATTCTTTGTTGCAAAATCTAGGTATCATGATGTCTTTCCGTGACCTAGTGGCGAGCCCATGGATACTGCTAGTAATA       4 JX220408.1       96.9%       AATGTTAATTGAAGTTTTCATTAATTCTTTGTTGCAAAATCTAGGTATCATGATGTCTTTCCGTGACCTAGTGGCGAGCCCATGGATACTGCTAGTAATA       5 Nora_MV          96.6%       AATGTTAATTGAAGTTTTCATTAATTCTTTGTTGCAAAATCTAGGTATCATGATGTCTTTCCGTGACCTAGTGGCGAGCCCATGGATACTGCTAGTAATA                                  2001          .         .         .         .         :         .         .         .         .         1 2100  1 NC_007919.3     100.0%       GCTATACCCTTGTGTGCATTTGCCAGTTCAGCATCTATGGTTAGGGAGATGCTTTTCCGTCATAAAATTACAGAAAATATTCTAAAAGGAACAGGAGTAG       2 Nora_Median-Nor  96.8%       GCTATACCCTTGTGTGCATTTGCCAGTTCAGCGTCTATGGTTAGGGAGATGCTTTTCCGTCATAAAATTACAGAAAATATTTTAAAAGGAACAGGAGTAG       3 Nora_raw_reads   96.7%       GCTATACCCTTGTGTGCATTTGCCAGTTCAGCGTCTATGGTTAGGGAGATGCTTTTCCGTCATAAAATTACAGAAAATATTTTAAAAGGAACAGGAGTAG       4 JX220408.1       96.9%       GCTATACCCTTGTGTGCATTTGCCAGTTCAGCGTCTATGGTTAGGGAGATGCTTTTCCGTCATAAAATTACAGAAAATATTTTAAAAGGAACAGGAGTAG       5 Nora_MV          96.6%       GCTATACCCTTGTGTGCATTTGCCAGTTCAGCGTCTATGGTTAGGGAGATGCTTTTCCGTCATAAAATTACAGAAAATATTTTAAAAGGAACAGGAGTAG                                  2101          .         .         .         .         :         .         .         .         .         2 2200  1 NC_007919.3     100.0%       AAGAATTGTTTAATCCATTCGGGATAATTATTAAATATTTCCTTTATTTTGCAATTTTGTATGCTTTTATTAAATATATTAGAAATAACATTAACGTAAT       2 Nora_Median-Nor  96.8%       AAGAATTGTTTAATCCATTCGGGATAATTATTAAATATTTCCTTTATTTTGCAATTTTGTATGCTTTTATTAAATATATTAGAAATAACATTAACGTAAT       3 Nora_raw_reads   96.7%       AAGAATTGTTTAATCCATTCGGGATAATTATTAAATATTTCCTTTATTTTGCAATTTTGTATGCTTTTATTAAATATATTAGAAATAACATTAACGTAAT       4 JX220408.1       96.9%       AAGAATTGTTTAATCCATTCGGGATAATTATTAAATATTTCCTTTATTTTGCAATTTTGTATGCTTTTATTAAATATATTAGAAATAACATTAACGTAAT       5 Nora_MV          96.6%       AAGAATTGTTTAATCCATTCGGGATAATTATTAAATATTTCCTTTATTTTGCAATTTTGTATGCTTTTATTAAATATATTAGAAATAACATTAACGTAAT                                  2201          .         .         .         .         :         .         .         .         .         3 2300  1 NC_007919.3     100.0%       AACAGAAAAAGTTAATTTTATACGGAGAGTAGTCTCAAACCCAACTGGAACAACAGGACGTAGAGGTGTGTTGGGGCGATGTGTAGAACAAATCATAGAA       2 Nora_Median-Nor  96.8%       AACAGAGAAAGTTAATTTTATACGGAGAATAGTATCGAACCCAACTGGAACAACAGGACGTAGAGGTGTGTTAGGGCGATGTGTAGAACAAATCATAGAA       3 Nora_raw_reads   96.7%       AACAGAGAAAGTTAATTTTATACGGAGAATAGTATCGAACCCAACTGGAACAACAGGACGTAGAGGTGTGTTAGGGCGATGTGTAGAACAAATCATAGAA       4 JX220408.1       96.9%       AACAGAGAAAGTTAATTTTATACGGAGAATAGTATCGAACCCAACTGGAACAACAGGACGTAGAGGTGTGTTAGGGCGATGTGTAGAACAAATCATAGAA       5 Nora_MV          96.6%       AACAGAGAAAGTTAATTTTATACGGAGAATAGTATCGAACCCAACTGGAACAACAGGACGTAGAGGTGTGTTAGGGCGATGTGTAGAACAAATCATAGAA                                  2301          .         .         .         .         :         .         .         .         .         4 2400  1 NC_007919.3     100.0%       TATCCAACATTCTTTATTACGATGATCTACGAACTACAGCAAATTAAGAACAAAAAAGATCTTATCTCAAAAATTACGATGATAAGTAGCATTCTTAAGT       2 Nora_Median-Nor  96.8%       TATCCAACATTCTTTATCACGATGGTCTACGAACTACAGCAAATTAAGAACAAAAAAGATCTTATCTCGAAAATTACGATGATAAGTAGTATTCTCAAGT       3 Nora_raw_reads   96.7%       TATCCAACATTCTTTATCACGATGGTCTACGAACTACAGCAAATTAAGAACAAAAAAGATCTTATCTCGAAAATTACGATGATAAGTAGTATTCTCAAGT       4 JX220408.1       96.9%       TATCCAACATTCTTTATCACGATGGTCTACGAACTACAGCAAATTAAGAACAAAAAAGATCTTATCTCGAAAATTACGATGATAAGTAGTATTCTCAAGT       5 Nora_MV          96.6%       TATCCAACATTCTTTATCACGATGGTCTACGAACTACAGCAAATTAAGAACAAAAAAGATCTTATCTCGAAAATTACGATGATAAGTAGTATTCTCAAGT                                  2401          .         .         .         .         :         .         .         .         .         5 2500  1 NC_007919.3     100.0%       TACCACTTGGTATTTGGGAAAGTACTGTAGGGCGGATGCTAGATCGACCGGCGATAGAGGGAACAGAAGAAATGTTGGAAGATGTTCTACCTATAGTAGC       2 Nora_Median-Nor  96.8%       TACCACTTGGTATTTGGGAAAGTACTGTAGGACGGATGCTAGATCGACCGGCGATAGAAGGAACAGAAGAAATGTTGGAAGATGTTCTACCTATGGTAGC       3 Nora_raw_reads   96.7%       TACCACTTGGTATTTGGGAAAGTACTGTAGGACGGATGCTAGATCGACCGGCGATAGAAGGAACAGAAGAAATGTTGGAAGATGTTCTACCTATGGTAGC       4 JX220408.1       96.9%       TACCACTTGGTATTTGGGAAAGTACTGTAGGACGGATGCTAGATCGACCGGCGATAGAAGGAACAGAAGAAATGTTGGAAGATGTTCTACCTATGGTAGC       5 Nora_MV          96.6%       TACCACTTGGTATTTGGGAAAGTACTGTAGGACGGATGCTAGATCGACCGGCGATAGAAGGAACAGAAGAAATGTTGGAAGATGTTCTACCTATGGTAGC                                  2501          .         .         .         .         :         .         .         .         .         6 2600  1 NC_007919.3     100.0%       AATGGGATTAACGATTACAAAGACTCAAATTGGAGATGTTCCAGTTGAAAGTTTTCTTGTGAATTTGGACCGTAATCAAAAAGCTTGCGAAAATATAATA       2 Nora_Median-Nor  96.8%       AATGGGATTGACGATTACAAAGACTCAAATTGGAGATGTTCCAGTTGAAAGTTTTCTTGTGAATTTGGACCGTAATCAAAAGGCTTGCGAAAATATAATA       3 Nora_raw_reads   96.7%       AATGGGATTGACGATTACAAAGACTCAAATTGGAGATGTTCCAGTTGAAAGTTTTCTTGTGAATTTGGACCGTAATCAAAAGGCTTGCGAAAATATAATA       4 JX220408.1       96.9%       AATGGGATTGACGATTACAAAGACTCAAATTGGAGATGTTCCAGTTGAAAGTTTTCTTGTGAATTTGGACCGTAATCAAAAGGCTTGCGAAAATATAATA       5 Nora_MV          96.6%       AATGGGATTGACGATTACAAAGACTCAAATTGGAGATGTTCCAGTTGAAAGTTTTCTTGTGAATTTGGACCGTAATCAAAAGGCTTGCGAAAATATAATA                                  2601          .         .         .         .         :         .         .         .         .         7 2700  1 NC_007919.3     100.0%       AAACGTATGCAGCCATTGATGATTAAAATGGGAATGATGAAAGATAGCTCATATGACACTATTTTGCAAGTAGCGAAAGAAGTTAACGAGTTATCGGAAG       2 Nora_Median-Nor  96.8%       AAACGTATGCAGCCGTTGATGATTAAAATGGGAATGATGAAAGATAGTTCATATGATACTATTTTGCAAGTTGCAAAAGAAGTTAATGAATTATCGGAAG       3 Nora_raw_reads   96.7%       AAACGTATGCAGCCGTTGATGATTAAAATGGGAATGATGAAAGATAGTTCATATGATACTATTTTGCAAGTTGCAAAAGAAGTTAATGAATTATCGGAAG       4 JX220408.1       96.9%       AAACGTATGCAGCCGTTGATGATTAAAATGGGAATGATGAAAGATAGTTCATATGATACTATTTTGCAAGTTGCAAAAGAAGTTAATGAATTATCGGAAG       5 Nora_MV          96.6%       AAACGTATGCAGCCGTTGATGATTAAAATGGGAATGATGAAAGATAGTTCATATGATACTATTTTGCAAGTTGCAAAAGAAGTTAATGAATTATCGGAAG                                  2701          .         .         .         .         :         .         .         .         .         8 2800  1 NC_007919.3     100.0%       CGGAAACATGGATGAAAACAACGCTTAAATTAAACCCAAATGAGTTCTTACAAACACAAGGAGCTGTAAGAGTTGGCGAAATCAGGGAAAAGGTTGCAAC       2 Nora_Median-Nor  96.8%       CGGAAACATGGATGAAAACAACGCTCAAATTAAACCCAAATGAGTTCTTACAAACACAAGGGGCTGTAAGAGTTGGCGAAATCAGGGAAAAAGTTGCAAC       3 Nora_raw_reads   96.7%       CGGAAACATGGATGAAAACAACGCTCAAATTAAACCCAAATGAGTTCTTACAAACACAAGGGGCTGTAAGAGTTGGCGAAATCAGGGAAAAAGTTGCAAC       4 JX220408.1       96.9%       CGGAAACATGGATGAAAACAACGCTCAAATTAAACCCAAATGAGTTCTTACAAACACAAGGGGCTGTAAGAGTTGGCGAAATCAGGGAAAAAGTTGCAAC       5 Nora_MV          96.6%       CGGAAACATGGATGAAAACAACGCTCAAATTAAACCCAAATGAGTTCTTACAAACACAAGGGGCTGTAAGAGTTGGCGAAATCAGGGAAAAAGTTGCAAC                                  2801          .         .         .         .         :         .         .         .         .         9 2900  1 NC_007919.3     100.0%       TTTGAGAAATAAGTTAAACACTTTGCAAACAAAAGAACTTCGATCAGATAAAGTAGTAACAGAGTGCCAAAAACATCTGGCGTCACTCGAAGTACTACTT       2 Nora_Median-Nor  96.8%       TTTGAGAAATAAGTTAAACACTTTGCAAACGAAAGAACTTCGATCAGATAAAGTAGTAACAGAGTGCCAAAAACATCTGGCATCACTCGAAGTGCTACTT       3 Nora_raw_reads   96.7%       TTTGAGAAATAAGTTAAACACTTTGCAAACGAAAGAACTTCGATCAGATAAAGTAGTAACAGAGTGCCAAAAACATCTGGCATCACTCGAAGTGCTACTT       4 JX220408.1       96.9%       TTTGAGAAATAAGTTAAACACTTTGCAAACGAAAGAACTTCGATCAGATAAAGTAGTAACAGAGTGCCAAAAACATCTGGCATCACTCGAAGTGCTACTT       5 Nora_MV          96.6%       TTTGAGAAATAAGTTAAACACTTTGCAAACGAAAGAACTTCGATCAGATAAAGTAGTAACAGAGTGCCAAAAACATCTGGCATCACTCGAAGTGCTACTT                                  2901          .         .         .         .         :         .         .         .         .         0 3000  1 NC_007919.3     100.0%       ATTGAAGTTAAGGTGTTAGAGAACAGCAACCAAACCCGAGTAAAACCCGTAGGAGTTACCATACAAGGTGAAAAACAAATCGGAAAGACAAACCTCGTAG       2 Nora_Median-Nor  96.8%       ATTGAAGTTAAGGTGTTAGAGAACAGTAACCAAACCCGAGTAAAACCCGTAGGAGTTACCATACAAGGCGAAAAACAAATCGGAAAGACAAACCTCGTAG       3 Nora_raw_reads   96.7%       ATTGAAGTTAAGGTGTTAGAGAACAGTAACCAAACCCGAGTAAAACCCGTAGGAGTTACCATACAAGGCGAAAAACAAATCGGAAAGACAAACCTCGTAG       4 JX220408.1       96.9%       ATTGAAGTTAAGGTGTTAGAGAACAGTAACCAAACCCGAGTAAAACCCGTAGGAGTTACCATACAAGGCGAAAAACAAATCGGAAAGACAAACCTCGTAG       5 Nora_MV          96.6%       ATTGAAGTTAAGGTGTTAGAGAACAGTAACCAAACCCGAGTAAAACCCGTAGGAGTTACCATACAAGGCGAAAAACAAATCGGAAAGACAAACCTCGTAG                                  3001          .         .         .         .         :         .         .         .         .         1 3100  1 NC_007919.3     100.0%       CAATCCTCTCAAGAAAAATATGCGAGTATGTTCAGGAACATGGCGATATTTCTTTTAGAAACGCCACTAAATGGACAACTTGGTCTAGGCAGTGCAGAGA       2 Nora_Median-Nor  96.8%       CAATCCTCTCAAGAAAAGTGTGCGAGTATGTTCAGGAACATGGCGATATTTCTTTTAGAAACGCTACTAAATGGACAACTTGGTCTAGGCAGTGCAGAGA       3 Nora_raw_reads   96.7%       CAATCCTCTCAAGAAAAGTGTGCGAGTATGTTCAGGAACATGGCGATATTTCTTTTAGAAACGCTACTAAATGGACAACTTGGTCTAGGCAGTGCAGAGA       4 JX220408.1       96.9%       CAATCCTCTCAAGAAAAGTGTGCGAGTATGTTCAGGAACATGGCGATATTTCTTTTAGAAACGCTACTAAATGGACAACTTGGTCTAGGCAGTGCAGAGA       5 Nora_MV          96.6%       CAATCCTCTCAAGAAAAGTGTGCGAGTATGTTCAGGAACATGGCGATATTTCTTTTAGAAACGCTACTAAATGGACAACTTGGTCTAGGCAGTGCAGAGA                                  3101          .         .         .         .         :         .         .         .         .         2 3200  1 NC_007919.3     100.0%       TGAATTTGATACAGGATACACCGGACAAGAAATAACTTATGTAGATGATGCTTTTCAACAAAAAGACAACAAGGATCATTTGATGTGGTTTACTTTTATT       2 Nora_Median-Nor  96.8%       TGAATTTGATACAGGATACACCGGACAAGAAATAACTTACGTAGATGATGCCTTTCAACAAAAAGACAACAAGGATCATTTGATGTGGTTTACTTTTATT       3 Nora_raw_reads   96.7%       TGAATTTGATACAGGATACACCGGACAAGAAATAACTTACGTAGATGATGCCTTTCAACAAAAAGACAACAAGGATCATTTGATGTGGTTTACTTTTATT       4 JX220408.1       96.9%       TGAATTTGATACAGGATACACCGGACAAGAAATAACTTACGTAGATGATGCCTTTCAACAAAAAGACAACAAGGATCATTTGATGTGGTTTACTTTTATT       5 Nora_MV          96.6%       TGAATTTGATACAGGATACACCGGACAAGAAATAACTTACGTAGATGATGCCTTTCAACAAAAAGACAACAAGGATCATTTGATGTGGTTTACTTTTATT                                  3201          .         .         .         .         :         .         .         .         .         3 3300  1 NC_007919.3     100.0%       TCTAATACAGCAGTAGGAACAAACCAAGCTGATTTGAAACAAAAAGGTTTACCATACAGAAGTAAGTTGGTGTTTACGACCTGTAACAAGTTGCCAGACA       2 Nora_Median-Nor  96.8%       TCTAATACAGCAGTGGGAACAAACCAAGCTGATTTGAAACAAAAAGGTTTACCATACAGAAGTAAGTTGGTGTTTACGACCTGTAACAAGTTGCCGGACA       3 Nora_raw_reads   96.7%       TCTAATACAGCAGTGGGAACAAACCAAGCTGATTTGAAACAAAAAGGTTTACCATACAGAAGTAAGTTGGTGTTTACGACCTGTAACAAGTTGCCGGACA       4 JX220408.1       96.9%       TCTAATACAGCAGTGGGAACAAACCAAGCTGATTTGAAACAAAAAGGTTTACCATACAGAAGTAAGTTGGTGTTTACGACCTGTAACAAGTTGCCGGACA       5 Nora_MV          96.6%       TCTAATACAGCAGTGGGAACAAACCAAGCTGATTTGAAACAAAAAGGTTTACCATACAGAAGTAAGTTGGTGTTTACGACCTGTAACAAGTTGCCGGACA                                  3301          .         .         .         .         :         .         .         .         .         4 3400  1 NC_007919.3     100.0%       AAAGTGTGACGATTGAGGATATCGAAGCATTGCATGCTCGATTCCCTCATACAATTTGCTTGAGGAGGAACAAGAACAAAATGCCAAAACGAGGAGCGAT       2 Nora_Median-Nor  96.8%       AAAGCGTAACGATCGAGGATATCGAAGCATTACATGCTCGATTCCCTCATACAATTTGCTTGAGGAGGAACAAGAACAAAATGCCAAAACGAGGAGCGAT       3 Nora_raw_reads   96.7%       AAAGCGTAACGATTGAGGATATCGAAGCATTACATGCTCGATTCCCTCATACAATTTGCTTGAGGAGGAACAAGAACAAAATGCCAAAACGAGGAGCGAT       4 JX220408.1       96.9%       AAAGCGTAACGATTGAGGATATCGAAGCATTACATGCTCGATTCCCTCATACAATTTGCTTGAGGAGGAACAAGAACAAAATGCCAAAACGAGGAGCGAT       5 Nora_MV          96.6%       AAAGCGTAACGATTGAGGATATCGAAGCATTACATGCTCGATTCCCTCATACAATTTGCTTGAGGAGGAACAAGAACAAAATGCCAAAACGAGGAGCGAT                                  3401          .         .         .         .         :         .         .         .         .         5 3500  1 NC_007919.3     100.0%       TGACGAGAGCTATGACTGGGTCGATTTTTATTACGGACCAATGTCGAAAGCTGTTAGTGCAATTGGAAGCAACACGACCAGCACACTAAAAACCATGTCT       2 Nora_Median-Nor  96.8%       TGACGAGAGCTATGACTGGGTCGATTTTTATTACGGACCAATGTCGAAAGCCGTCAGCGCTATTGGAAGCAACACGACCAGCACGCTAAAAACCATGTCT       3 Nora_raw_reads   96.7%       TGACGAGAGCTATGACTGGGTCGATTTTTATTACGGACCAATGTCGAAAGCCGTCAGCGCTATTGGAAGCAACACGACCAGCACGCTAAAAACCATGTCT       4 JX220408.1       96.9%       TGACGAGAGCTATGACTGGGTCGATTTTTATTACGGACCAATGTCGAAAGCCGTCAGCGCTATTGGAAGCAACACGACCAGCACGCTAAAAACCATGTCT       5 Nora_MV          96.6%       TGACGAGAGCTATGACTGGGTCGATTTTTATTACGGACCAATGTCGAAAGCCGTCAGCGCTATTGGAAGCAACACGACCAGCACGCTAAAAACCATGTCT                                  3501          .         .         .         .         :         .         .         .         .         6 3600  1 NC_007919.3     100.0%       TTGAGTGAAATAGTGAAGATAATTGGAGATGATTTGATTATTCAAAATAATTTCTATAACTCCACTATTAAAGATGTGGGAATCACCGGACAAGAACAAA       2 Nora_Median-Nor  96.8%       TTGAGTGAAATAGTGAAGATAATTGGAGATGATTTGATTATTCAAAATAATTTCTATAACTCCACTATTAAAGATGTGGGAATCACCGGACAAGAACAAA       3 Nora_raw_reads   96.7%       TTGAGTGAAATAGTGAAGATAATTGGAGATGATTTGATTATTCAAAATAATTTCTATAACTCCACTATTAAAGATGTGGGAATCACCGGACAAGAACAAA       4 JX220408.1       96.9%       TTGAGTGAAATAGTGAAGATAATTGGAGATGATTTGATTATTCAAAATAATTTCTATAACTCCACTATTAAAGATGTGGGAATCACCGGACAAGAACAAA       5 Nora_MV          96.6%       TTGAGTGAAATAGTGAAGATAATTGGAGATGATTTGATTATTCAAAATAATTTCTATAACTCCACTATTAAAGATGTGGGAATCACCGGACAAGAACAAA                                  3601          .         .         .         .         :         .         .         .         .         7 3700  1 NC_007919.3     100.0%       TGGATGGAGCGCAATTAGAGAGAAGACAACGAATGCGCGAATTGAGGGATCACTTGTTGAGAATCCGCTCAGGAGACGAAAACATGCCTTTCCTTGACGA       2 Nora_Median-Nor  96.8%       TGGATGGAGCGCAATTAGAAAGAAGACAACGAATGCGCGAATTGAGGGATCACTTGTTGAGAATCCGCCCAGGAGACGAAAACATGCCTTTCCTTGATGA       3 Nora_raw_reads   96.7%       TGGATGGAGCGCAATTAGAAAGAAGACAACGAATGCGCGAATTGAGGGATCACTTGTTGAGAATCCGCCCAGGAGACGAAAACATGCCTTTCCTTGATGA       4 JX220408.1       96.9%       TGGATGGAGCGCAATTAGAAAGAAGACAACGAATGCGCGAATTGAGGGATCACTTGTTGAGAATCCGCCCAGGAGACGAAAACATGCCTTTCCTTGATGA       5 Nora_MV          96.6%       TGGATGGAGCGCAATTAGAAAGAAGACAACGAATGCGCGAATTGAGGGATCACTTGTTGAGAATCCGCCCAGGAGACGAAAACATGCCTTTCCTTGATGA                                  3701          .         .         .         .         :         .         .         .         .         8 3800  1 NC_007919.3     100.0%       AACATTTGAGTTGAATAGCCGACCTATCCAAACAGATGAGAAATTTATACCGCTCAAAGATAACTTAGATGAAGAAGTTATGTATGGCGGTATTTCAGAT       2 Nora_Median-Nor  96.8%       AACATTTGAGTTGAATAGCCGACCTATCCAGACAGATGAGAAATTTATACCGCTCAAAGATAACTTAGATGAAGAAGTTATGTATGGCGGTATTTCAGAT       3 Nora_raw_reads   96.7%       AACATTTGAGTTGAATAGCCGACCTATCCAGACAGATGAGAAATTTATACCGCTCAAAGATAACTTAGATGAAGAAGTTATGTATGGCGGTATTTCAGAT       4 JX220408.1       96.9%       AACATTTGAGTTGAATAGCCGACCTATCCAGACAGATGAGAAATTTATACCGCTCAAAGATAACTTAGATGAAGAAGTTATGTATGGCGGTATTTCAGAT       5 Nora_MV          96.6%       AACATTTGAGTTGAATAGCCGACCTATCCAGACAGATGAGAAATTTATACCGCTCAAAGATAACTTAGATGAAGAAGTTATGTATGGCGGTATTTCAGAT                                  3801          .         .         .         .         :         .         .         .         .         9 3900  1 NC_007919.3     100.0%       CAGTTGTTAACTAGATTTGATAATATCATTGAAAGGAGTTTGGAAGGTTATGATGTTGAGTCGCGGGAGCTAGGTGTTGAACCTTTAACTACATTAAATC       2 Nora_Median-Nor  96.8%       CAGTTGTTAACTAGATTTGATAATATCATCGAAAGGAGTTTAGAAGGCTATGATGTTGAGTCGCGAGAGCTAGGTGTTGAACCTTTAACTACACTAAATC       3 Nora_raw_reads   96.7%       CAGTTGTTAACTAGATTTGATAATATCATCGAAAGGAGTTTAGAAGGCTATGATGTTGAGTCGCGAGAGCTAGGTGTTGAACCTTTAACTACACTAAATC       4 JX220408.1       96.9%       CAGTTGTTAACTAGATTTGATAATATCATCGAAAGGAGTTTAGAAGGCTATGATGTTGAGTCGCGAGAGCTAGGTGTTGAACCTTTAACTACACTAAATC       5 Nora_MV          96.6%       CAGTTGTTAACTAGATTTGATAATATCATCGAAAGGAGTTTAGAAGGCTATGATGTTGAGTCGCGAGAGCTAGGTGTTGAACCTTTAACTACACTAAATC                                  3901          .         .         .         .         :         .         .         .         .         0 4000  1 NC_007919.3     100.0%       ATGTAAGAAGTAATATGTTGAGTTATAGAGCGTGGAATCTCATTAATTCCATGTGTATTAACAAAACAGAAACTTTTAGTGCTTGGTTAGGAAGGTACAT       2 Nora_Median-Nor  96.8%       ACGTGAGAAGTAATATGTTGAGTTATAGAGCGTGGAATCTCATTAATTCCATGTGTATTAACAAAACAGAAACTTTCAATGCTTGGTTAGGAAGGTACAT       3 Nora_raw_reads   96.7%       ACGTGAGAAGTAATATGTTGAGTTATAGAGCGTGGAATCTCATTAATTCCATGTGTATTAACAAAACAGAAACTTTCAGTGCTTGGTTAGGAAGGTACAC       4 JX220408.1       96.9%       ACGTGAGAAGTAATATGTTGAGTTATAGAGCGTGGAATCTCATTAATTCCATGTGTATTAACAAAACAGAAACTTTCAGTGCTTGGTTAGGAAGGTACAT       5 Nora_MV          96.6%       ACGTGAGAAGTAATATGTTGAGTTATAGAGCGTGGAATCTCATTAATTCCATGTGTATTAACAAAACAGAAACTTTCAGTGCTTGGTTAGGAAGGTACAT                                  4001          .         .         .         .         :         .         .         .         .         1 4100  1 NC_007919.3     100.0%       CACCGAGTGTGTAGAAGGAGTAGCAGAGAATCTAGTTACGACAAAAGTCAAAATTAGAATTAATCCTTTTACAGGTTTGCAGTTGATCGCAGCCAAACGA       2 Nora_Median-Nor  96.8%       CACCGAATGTGTAGAAGGAGCAGCAGAAAACCTAGTTACGACGAAAGTCAAAATTAGAGTTAATCCTTTTACAGGTTTGCAGTTGATCGCGGCCAAACGA       3 Nora_raw_reads   96.7%       CCGTCTTGTAACAGAAGGAGCAGCAGAAAATCTAGTTACGACGAAAGTCAAAATTAGAGTTAATCCTTTTACAGGTTTGCAGTTGATCGCGGCCAAACGA       4 JX220408.1       96.9%       CACCGAATGTGTAGAAGGAGCAGCAGAAAATCTAGTTACGACGAAAGTCAAAATTAGAGTTAATCCTTTTACAGGTTTGCAGTTGATCGCGGCCAAACGA       5 Nora_MV          96.6%       CACCGAATGTGTAGAAGGAGCAGCAGAAAATCTAGTTACGACGAAAGTCAAAATTAGAGTTAATCCTTTTACAGGTTTGCAGTTGATCGCGGCCAAACGA                                  4101          .         .         .         .         :         .         .         .         .         2 4200  1 NC_007919.3     100.0%       ATGTTGCAAGAGAATAAATTGATCGATATGGATGAAATTCCTTCGACATCAGCGAATTCATATGAAACAGTTTACGATCAAATCAAAAATTTCGTGAACG       2 Nora_Median-Nor  96.8%       ATGTTGCAAGAGAATAAATTGATCGATATGGATGAAATTCCATCGACATCAGCGAATTCATATGAAACAGTTTACGATCAAATCAAAAATTTCGTGAACG       3 Nora_raw_reads   96.7%       ATGTTGCAAGAGAATAAATTGATCGATATGGATGAAATTCCATCGACATCAGCGAATTCATATGAAACAGTTTACGATCAAATCAAAAATTTCGTGAACG       4 JX220408.1       96.9%       ATGTTGCAAGAGAATAAATTGATCGATATGGATGAAATTCCATCGACATCAGCGAATTCATATGAAACAGTTTACGATCAAATCAAAAATTTCGTGAACG       5 Nora_MV          96.6%       ATGTTGCAAGAGAATAAATTGATCGATATGGATGAAATTCCATCGACATCAGCGAATTCATATGAAACAGTTTACGATCAAATCAAAAATTTCGTGAACG                                  4201          .         .         .         .         :         .         .         .         .         3 4300  1 NC_007919.3     100.0%       ATGAGCTGAGTTTGATGGAAACAGATATAGTTGATTTGGCCTTAGCAAAAATTAGCTTATCGCAAATCCGAGGCAACATCAAAAGATCAACCTGGCTGGA       2 Nora_Median-Nor  96.8%       ATGAACTGAGTCTGATGGAAACAGATATAGTTGATTTAGCTTTAGCAAAAATTAGCTTATCGCAAATCCGAGGCAACATCAAGAGATCAACCTGGCTGGA       3 Nora_raw_reads   96.7%       ATGAACTGAGTCTGATGGAAACAGATATAGTTGATTTAGCTTTAGCAAAAATTAGCTTATCGCAAATCCGAGGCAACATCAAAAGATCAACCTGGCTGGA       4 JX220408.1       96.9%       ATGAACTGAGTCTGATGGAAACAGATATAGTTGATTTAGCTTTAGCAAAAATTAGCTTATCGCAAATCCGAGGCAACATCAAAAGATCAACCTGGCTGGA       5 Nora_MV          96.6%       ATGAACTGAGTCTGATGGAAACAGATATAGTTGATTTAGCTTTAGCAAAAATTAGCTTATCGCAAATCCGAGGCAACATCAAAAGATCAACCTGGCTGGA                                  4301          .         .         .         .         :         .         .         .         .         4 4400  1 NC_007919.3     100.0%       CGTGAATGATTGGATATTAGCTTTGAAACACAAAATCTCAGGAAAAAGCTTTGCCAAACATATGGATCTGTATCCTAGCTCGCTAGATTCTTTCTTACTT       2 Nora_Median-Nor  96.8%       CGTAAATGATTGGATATTAGCTTTGAAACATAAAATTTCAGGAAAAAGCTTTGCCAAACATATGGATCTGTATCCTAGCTCGCTAGATTCTTTTCTTCTT       3 Nora_raw_reads   96.7%       CGTAAATGATTGGATATTAGCTTTGAAACATAAAATTTCAGGAAAAAGCTTTGCCAAACATATGGATCTGTATCCTAGCTCGCTAGATTCTTTTCTTCTT       4 JX220408.1       96.9%       CGTAAATGATTGGATATTAGCTTTGAAACATAAAATTTCAGGAAAAAGCTTTGCCAAACATATGGATCTGTATCCTAGCTCGCTAGATTCTTTTCTTCTT       5 Nora_MV          96.6%       CGTAAATGATTGGATATTAGCTTTGAAACATAAAATTTCAGGAAAAAGCTTTGCCAAACATATGGATCTGTATCCTAGCTCGCTAGATTCTTTTCTTCTT                                  4401          .         .         .         .         :         .         .         .         .         5 4500  1 NC_007919.3     100.0%       ACACTCAAAGATTGGGAAGTTGAAGACCGTATAAAGTTTAACTCTATTTACAAGCAAAAAGTATTGTTTGTTCAATCTAGATTTTCACTTTACTGTTGGT       2 Nora_Median-Nor  96.8%       ACACTCAAAGATTGGGAGGTTGAAGACCGTATAAAGTTTAACTCTATTTACAAGCAAAAGGTATTGTTTGTTCAATCTAGATTTTCACTTTACTGTTGGT       3 Nora_raw_reads   96.7%       ACACTCAAAGATTGGGAGGTTGAAGACCGTATAAAGTTTAACTCTATTTACAAGCAAAAGGTATTGTTTGTTCAATCTAGATTTTCACTTTACTGTTGGT       4 JX220408.1       96.9%       ACACTCAAAGATTGGGAGGTTGAAGACCGTATAAAGTTTAACTCTATTTACAAGCAAAAGGTATTGTTTGTTCAATCTAGATTTTCACTTTACTGTTGGT       5 Nora_MV          96.6%       ACACTCAAAGATTGGGAGGTTGAAGACCGTATAAAGTTTAACTCTATTTACAAGCAAAAGGTATTGTTTGTTCAATCTAGATTTTCACTTTACTGTTGGT                                  4501          .         .         .         .         :         .         .         .         .         6 4600  1 NC_007919.3     100.0%       CTCCTTTTATATCTCGTGGAACACGATTTGTGAAAGTGACTTCTCGATTTAGAGAGTTAGTGGACAAGTTAGAGACTGGCATTCTATTCCATGAAATAAA       2 Nora_Median-Nor  96.8%       CTCCTTTTATATCTCGTGGAACACGATTTGTGAAAGTGACTTCTCAGTTTAGAGAGTTAGTGGATAAGTTAGAAACTGGCATTCTATTCCATGAAATAAA       3 Nora_raw_reads   96.7%       CTCCTTTTATATCTCGTGGAACACGATTTGTGAAAGTGACTTCTCAGTTTAGAGAGTTAGTGGATAAGTTAGAAACTGGCATTCTATTCCATGAAATAAA       4 JX220408.1       96.9%       CTCCTTTTATATCTCGTGGAACACGATTTGTGAAAGTGACTTCTCAGTTTAGAGAGTTAGTGGATAAGTTAGAAACTGGCATTCTATTCCATGAAATAAA       5 Nora_MV          96.6%       CTCCTTTTATATCTCGTGGAACACGATTTGTGAAAGTGACTTCTCAGTTTAGAGAGTTAGTGGATAAGTTAGAAACTGGCATTCTATTCCATGAAATAAA                                  4601          .         .         .         .         :         .         .         .         .         7 4700  1 NC_007919.3     100.0%       ATCAGTCACAAATGGAATTAGATGGTTAGGAGGAGCAGGGAACAATGGACACGTTGGAGAAAGAGTAAGAGTTATTGCTCACACAGCTCAATTTCCGAAA       2 Nora_Median-Nor  96.8%       ATCGGTTACAAATGGAATTAGATGGTTAGGAGGAGCAGGGAACAATGGACACGTTGGAGAAAGAGTAAGAGTTATTGCTCATACAGCCCAATTTCCGAAA       3 Nora_raw_reads   96.7%       ATCGGTTACAAATGGAATTAGATGGTTAGGAGGAGCAGGGAACAATGGACACGTTGGAGAAAGAGTAAGAGTTATTGCTCATACAGCCCAATTTCCGAAA       4 JX220408.1       96.9%       ATCGGTTACAAATGGAATTAGATGGTTAGGAGGAGCAGGGAACAATGGACACGTTGGAGAAAGAGTAAGAGTTATTGCTCATACAGCCCAATTTCCGAAA       5 Nora_MV          96.6%       ATCGGTTACAAATGGAATTAGATGGTTAGGAGGAGCAGGGAACAATGGACACGTTGGAGAAAGAGTAAGAGTTATTGCTCATACAGCCCAATTTCCGAAA                                  4701          .         .         .         .         :         .         .         .         .         8 4800  1 NC_007919.3     100.0%       AAGAGTTACCCTCAGAATGGATTCCCTATAAACGAAGAACTACATAGGGAATGGATCCAGCTCGTCATAAACTCGGACTATAAGTATCACTCACTTATAG       2 Nora_Median-Nor  96.8%       AAGAGTTACCCTCAGAATGGATTCCCTATAAACGAAGAACTACATAGGGAATGGATCCAACTTGTTATGAACTCGGACTATAAGTATCACTCACTTATAG       3 Nora_raw_reads   96.7%       AAGAGTTACCCTCAGAATGGATTCCCTATAAACGAAGAACTACATAGGGAATGGATCCAACTTGTTATGAACTCGGACTATAAGTATCACTCACTTATAG       4 JX220408.1       96.9%       AAGAGTTACCCTCAGAATGGATTCCCTATAAACGAAGAACTACATAGGGAATGGATCCAACTTGTTATGAACTCGGACTATAAGTATCACTCACTTATAG       5 Nora_MV          96.6%       AAGAGTTACCCTCAGAATGGATTCCCTATAAACGAAGAACTACATAGGGAATGGATCCAACTTGTTATGAACTCGGACTATAAGTATCACTCACTTATAG                                  4801          .         .         .         .         :         .         .         .         .         9 4900  1 NC_007919.3     100.0%       GAGAAGAAAAAGTAAATATTCTTTGGAACCTTATTAGGCTCCAACCTCAACATGAGGTGGAGAATTTTAAGGTTTACTTAGAAGATTTGCAGGCGTCCCC       2 Nora_Median-Nor  96.8%       GAGAAGAAAAAGTAAATATTCTTTGGAACCTTATTAGGCTCCAACCTCAACATGAGGTGGAGAATTTTAAGGTTTACTTAGAAGATTTGCAGGCGTCCCC       3 Nora_raw_reads   96.7%       GAGAAGAAAAAGTAAATATTCTTTGGAACCTTATTAGGCTCCAACCTCAACATGAGGTGGAGAATTTTAAGGTTTACTTAGAAGATTTGCAGGCGTCCCC       4 JX220408.1       96.9%       GAGAAGAAAAAGTAAATATTCTTTGGAACCTTATTAGGCTCCAACCTCAACATGAGGTGGAGAATTTTAAGGTTTACTTAGAAGATTTGCAGGCGTCCCC       5 Nora_MV          96.6%       GAGAAGAAAAAGTAAATATTCTTTGGAACCTTATTAGGCTCCAACCTCAACATGAGGTGGAGAATTTTAAGGTTTACTTAGAAGATTTGCAGGCGTCCCC                                  4901          .         .         .         .         :         .         .         .         .         0 5000  1 NC_007919.3     100.0%       GCCGAAAACTGGGACAATTTGTGCAAAAGTAGTGAATGATATAAAAGCCGAAGTAACTTCAAGTTATAGGCAATTTAACAATTACTACACTCGTTTAACA       2 Nora_Median-Nor  96.8%       GCCGAAAACTGGGACAATTTGTGCAAAAGTAGTGAATGATATAAAAGCCGAAGTAACTTCAAGTTATAGGCAATTTAACAATTACTACACTCGTTTAACA       3 Nora_raw_reads   96.7%       GCCGAAAACTGGGACAATTTGTGCAAAAGTAGTGAATGATATAAAAGCCGAAGTAACTTCAAGTTATAGGCAATTTAACAATTACTACACTCGTTTAACA       4 JX220408.1       96.9%       GCCGAAAACTGGGACAATTTGTGCAAAAGTAGTGAATGATATAAAAGCCGAAGTAACTTCAAGTTATAGGCAATTTAACAATTACTACACTCGTTTAACA       5 Nora_MV          96.6%       GCCGAAAACTGGGACAATTTGTGCAAAAGTAGTGAATGATATAAAAGCCGAAGTAACTTCAAGTTATAGGCAATTTAACAATTACTACACTCGTTTAACA                                  5001          .         .         .         .         :         .         .         .         .         1 5100  1 NC_007919.3     100.0%       AAAGATGGTATGCACACACTTTTATCAATGCTTTCCAGGATAGGTGTTCCAATTTCAGATTATTGGAATGACCTTCTTGTCGATAAAGCACCAGCTATTA       2 Nora_Median-Nor  96.8%       AAAGATGGTATGCACACACTTTTATCAATGCTTTCTAGGATAGGTGTTCCAATTTCAGATTATTGGAATGACCTTCTTGTCGACAAAGCGCCAGCTATTA       3 Nora_raw_reads   96.7%       AAAGATGGTATGCACACACTTTTATCAATGCTTTCTAGGATAGGTGTTCCAATTTCAGATTATTGGAATGACCTTCTTGTCGACAAAGCGCCAGCTATTA       4 JX220408.1       96.9%       AAAGATGGTATGCACACACTTTTATCAATGCTTTCTAGGATAGGTGTTCCAATTTCAGATTATTGGAATGACCTTCTTGTCGACAAAGCGCCAGCTATTA       5 Nora_MV          96.6%       AAAGATGGTATGCACACACTTTTATCAATGCTTTCTAGGATAGGTGTTCCAATTTCAGATTATTGGAATGACCTTCTTGTCGACAAAGCGCCAGCTATTA                                  5101          .         .         .         .         :         .         .         .         .         2 5200  1 NC_007919.3     100.0%       CAGCGGTTACTGTTGGAGCAATAACAAGTCTAGCTATTATCACAATAGTAAAAACTTTCCAATATGCTATAGCTGGAGAAGAGCAAAGTAAAGGTGAAAA       2 Nora_Median-Nor  96.8%       CAGCGGTTACTGTTGGAGCAATAACAAGTTTAGCTATTATTACAATAGTAAAAACTTTTCAATATGCTATAGCTGGAGAAGAGCAAAGTAAAGGTGAAAA       3 Nora_raw_reads   96.7%       CAGCGGTTACTGTTGGAGCAATAACAAGTTTAGCTATTATTACAATAGTAAAAACTTTTCAATATGCTATAGCTGGAGAAGAGCAAAGTAAAGGTGAAAA       4 JX220408.1       96.9%       CAGCGGTTACTGTTGGAGCAATAACAAGTTTAGCTATTATTACAATAGTAAAAACTTTTCAATATGCTATAGCTGGAGAAGAGCAAAGTAAAGGTGAAAA       5 Nora_MV          96.6%       CAGCGGTTACTGTTGGAGCAATAACAAGTTTAGCTATTATTACAATAGTAAAAACTTTTCAATATGCTATAGCTGGAGAAGAGCAAAGTAAAGGTGAAAA                                  5201          .         .         .         .         :         .         .         .         .         3 5300  1 NC_007919.3     100.0%       ACGAGCCAAACAGAAGAACATCGCAACAACTAAGCTTCAAAAATTGAAGTTTACACTTGGCAAAGAACAAGCTGAAGGGGATAGTATCGAACATGTTAAA       2 Nora_Median-Nor  96.8%       ACGAGCCAAACAGAAGAACATCGCAACAACTAAGCTTCAAAAATTGAAGTTTACACTTGGCAAAGAACAAGCTGAAGGGGATAGTATCGAACATGTTAAA       3 Nora_raw_reads   96.7%       ACGAGCCAAACAGAAGAACATCGCAACAACTAAGCTTCAAAAATTGAAGTTTACACTTGGCAAAGAACAAGCTGAAGGGGATAGTATCGAACATGTTAAA       4 JX220408.1       96.9%       ACGAGCCAAACAGAAGAACATCGCAACAACTAAGCTTCAAAAATTGAAGTTTACACTTGGCAAAGAACAAGCTGAAGGGGATAGTATCGAACATGTTAAA       5 Nora_MV          96.6%       ACGAGCCAAACAGAAGAACATCGCAACAACTAAGCTTCAAAAATTGAAGTTTACACTTGGCAAAGAACAAGCTGAAGGGGATAGTATCGAACATGTTAAA                                  5301          .         .         .         .         :         .         .         .         .         4 5400  1 NC_007919.3     100.0%       GAATTTGACGGTGATGTTAAGTTCGAAACAATCGAAAAACTGTTTGATCACATTGATGAACACCCCAACCTCAACATAGTGGGTTTGAACTTGATAGCAC       2 Nora_Median-Nor  96.8%       GAATTTGACGGCGATGTTAAGTTCGAAACAATTGAGAAACTATTTGATCACATTGATGAACACCCCAACCTCAACATAGTAGGCTTAAACTTGGTAGCAC       3 Nora_raw_reads   96.7%       GAATTTGACGGTGATGTTAAGTTCGAAACAATTGAGAAACTATTTGATCACATTGATGAACACCCCAACCTCAACATAGTAGGCTTAAACTTGGTAGCAC       4 JX220408.1       96.9%       GAATTTGACGGTGATGTTAAGTTCGAAACAATTGAGAAACTATTTGATCACATTGATGAACACCCCAACCTCAACATAGTAGGCTTAAACTTGGTAGCAC       5 Nora_MV          96.6%       GAATTTGACGGTGATGTTAAGTTCGAAACAATTGAGAAACTATTTGATCACATTGATGAACACCCCAACCTCAACATAGTAGGCTTAAACTTGGTAGCAC                                  5401          .         .         .         .         :         .         .         .         .         5 5500  1 NC_007919.3     100.0%       CAGAAAACCCAATTGCTATCTATGCCGCAAGAGAAGAATCGTACGACTTTTCATTCTCCGAACCGCGCCCGCCTCAATGGAAAAAGGTGGTAACGTTTAA       2 Nora_Median-Nor  96.8%       CAGAAAACCCAATTGCTATCTATGCCGCAAGAGAAGAATCGTACGACTTTTCATTTTCCGAACCGCGCCCGCCTCAATGGAAAAAGGTGGTAACGTTTAA       3 Nora_raw_reads   96.7%       CAGAAAACCCAATTGCTATCTATGCCGCAAGAGAAGAATCGTACGACTTTTCATTTTCCGAACCGCGCCCGCCTCAATGGAAAAAGGTGGTAACGTTTAA       4 JX220408.1       96.9%       CAGAAAACCCAATTGCTATCTATGCCGCAAGAGAAGAATCGTACGACTTTTCATTTTCCGAACCGCGCCCGCCTCAATGGAAAAAGGTGGTAACGTTTAA       5 Nora_MV          96.6%       CAGAAAACCCAATTGCTATCTATGCCGCAAGAGAAGAATCGTACGACTTTTCATTTTCCGAACCGCGCCCGCCTCAATGGAAAAAGGTGGTAACGTTTAA                                  5501          .         .         .         .         :         .         .         .         .         6 5600  1 NC_007919.3     100.0%       GGAAGACAGTAAACGAATAATCTCTCTACAGTTGCGAGGAGAAGATACTGAAGATAACATCCTTGATGAGATAGAACACGCTATAAAAGTCTCACATGGA       2 Nora_Median-Nor  96.8%       GGAAGACAGTAAACGAATAATCTCTCTACAGTTGCGAGGAGAAGATACTGAAGATAACATCCTTGATGAGATAGAACACGCTATAAAAGTCTCACATGGA       3 Nora_raw_reads   96.7%       GGAAGACAGTAAACGAATAATCTCTCTACAGTTGCGAGGAGAAGATACTGAAGATAACATCCTTGATGAGATAGAACACGCTATAAAAGTCTCACATGGA       4 JX220408.1       96.9%       GGAAGACAGTAAACGAATAATCTCTCTACAGTTGCGAGGAGAAGATACTGAAGATAACATCCTTGATGAGATAGAACACGCTATAAAAGTCTCACATGGA       5 Nora_MV          96.6%       GGAAGACAGTAAACGAATAATCTCTCTACAGTTGCGAGGAGAAGATACTGAAGATAACATCCTTGATGAGATAGAACACGCTATAAAAGTCTCACATGGA                                  5601          .         .         .         .         :         .         .         .         .         7 5700  1 NC_007919.3     100.0%       ATGCCGTATGCTGAATGGATATTCAATGGATGGTTTAAGAAAGAGAGCAACGACAATATATTATATTGTGTCGAGTTGGACCTGGTGACCGCAAAAACCC       2 Nora_Median-Nor  96.8%       ATGCCGTATGCTGAATGGATATTCAATGGATGGTTTAAGAAAGAGAGCAACGACAATATATTATATTGTGTCGAGTTGGACCTGGTGACCGCAAAAACCC       3 Nora_raw_reads   96.7%       ATGCCGTATGCTGAATGGATATTCAATGGATGGTTTAAGAAAGAGAGCAACGACAATATATTATATTGTGTCGAGTTGGACCTGGTGACCGCAAAAACCC       4 JX220408.1       96.9%       ATGCCGTATGCTGAATGGATATTCAATGGATGGTTTAAGAAAGAGAGCAACGACAATATATTATATTGTGTCGAGTTGGACCTGGTGACCGCAAAAACCC       5 Nora_MV          96.6%       ATGCCGTATGCTGAATGGATATTCAATGGATGGTTTAAGAAAGAGAGCAACGACAATATATTATATTGTGTCGAGTTGGACCTGGTGACCGCAAAAACCC                                  5701          .         .         .         .         :         .         .         .         .         8 5800  1 NC_007919.3     100.0%       AATCAGGCCCAGTTGGATGGACACGTGCTCAAACAAAGAACTTGAAAGATCTTGAAATTCAGCTAAATAAAGGCAAACCGATCGATGTTAAATCAGTGGT       2 Nora_Median-Nor  96.8%       AATCAGGCCCAGTTGGATGGACACGTGCTCAAACGAAAAACCTGAAAGATCTTGAAATTCAGCTAAATAAAGGCAAACCGATCGATGTTAAATCAGTGGT       3 Nora_raw_reads   96.7%       AATCAGGCCCAGTTGGATGGACACGTGCTCAAACGAAAAACCTGAAAGATCTTGAAATTCAGCTAAATAAAGGCAAACCGATCGATGTTAAATCAGTGGT       4 JX220408.1       96.9%       AATCAGGCCCAGTTGGATGGACACGTGCTCAAACGAAAAACCTGAAAGATCTTGAAATTCAGCTAAATAAAGGCAAACCGATCGATGTTAAATCAGTGGT       5 Nora_MV          96.6%       AATCAGGCCCAGTTGGATGGACACGTGCTCAAACGAAAAACCTGAAAGATCTTGAAATTCAGCTAAATAAAGGCAAACCGATCGATGTTAAATCAGTGGT                                  5801          .         .         .         .         :         .         .         .         .         9 5900  1 NC_007919.3     100.0%       TTTAGGTGCACCGCAAGCATCTACGCAGGCGACCGATACTATGGACGTGCTAGTGAATAAGCATTTAGTTAAAGTTCATTGTCTGAGTTGTGAAAATTTG       2 Nora_Median-Nor  96.8%       TTTAGGTGCACCGCAAGCATCTACGCAGGCGACCGATACTATGGACGTGCTAGTGAATAAGCATTTAGTTAAAGTTCATTGTCTGAGTTATGAAAATTTG       3 Nora_raw_reads   96.7%       TTTAGGTGCACCGCAAGCATCTACGCAGGCGACCGATACTATGGACGTGCTAGTGAATAAGCATTTAGTTAAAGTTCATTGTCTGAGTTATGAAAATTTG       4 JX220408.1       96.9%       TTTAGGTGCACCGCAAGCATCTACGCAGGCGACCGATACTATGGACGTGCTAGTGAATAAGCATTTAGTTAAAGTTCATTGTCTGAGTTATGAAAATTTG       5 Nora_MV          96.6%       TTTAGGTGCACCGCAAGCATCTACGCAGGCGACCGATACTATGGACGTGCTAGTGAATAAGCATTTAGTTAAAGTTCATTGTCTGAGTTATGAAAATTTG                                  5901          .         .         .         .         :         .         .         .         .         0 6000  1 NC_007919.3     100.0%       AACAACTTAGCTTTGAACGGGACACAAGTGTTTGCTTTAGCATCCGACAATATATTGATCGTGCCTGCACACGCAGCCAGACAGAATAAGTGGATCCGAT       2 Nora_Median-Nor  96.8%       AACAACTTAGCTTTGAACGGAACACAAGTGTTTGCTTTAGCATCCGACAATATATTGATCGTGCCTGCACATGCAGCCAGACAGAACAAGTGGATCCGAT       3 Nora_raw_reads   96.7%       AACAACTTAGCTTTGAACGGAACACAAGTGTTTGCTTTAGCATCCGACAATATATTGATCGTGCCTGCACATGCAGCCAGACAGAACAAGTGGATCCGAT       4 JX220408.1       96.9%       AACAACTTAGCTTTGAACGGAACACAAGTGTTTGCTTTAGCATCCGACAATATATTGATCGTGCCTGCACATGCAGCCAGACAGAACAAGTGGATCCGAT       5 Nora_MV          96.6%       AACAACTTAGCTTTGAACGGAACACAAGTGTTTGCTTTAGCATCCGACAATATATTGATCGTGCCTGCACATGCAGCCAGACAGAACAAGTGGATCCGAT                                  6001          .         .         .         .         :         .         .         .         .         1 6100  1 NC_007919.3     100.0%       TTAGTCGCGCAACACAAACTGGTCATTATGGAGTAGCGAAAGTTGATGAACGTAGAATTGATTTTACACGTGACATCGCTATAGCCATTATTTTGACTAG       2 Nora_Median-Nor  96.8%       TTAGTCGCGCAACACAAACTGGTCATTATGGAGTAGCGAAAGTTGATGAACGTAGAATTGATTTTACACGTGACATCGCTATAGCCGTTATTTTGACTAG       3 Nora_raw_reads   96.7%       TTAGTCGCGCAACACAAACTGGTCATTATGGAGTAGCGAAAGTTGATGAACGTAGAATTGATTTTACACGTGACATCGCTATAGCCGTTATTTTGACTAG       4 JX220408.1       96.9%       TTAGTCGCGCAACACAAACTGGTCATTATGGAGTAGCGAAAGTTGATGAACGTAGAATTGATTTTACACGTGACATCGCTATAGCCGTTATTTTGACTAG       5 Nora_MV          96.6%       TTAGTCGCGCAACACAAACTGGTCATTATGGAGTAGCGAAAGTTGATGAACGTAGAATTGATTTTACACGTGACATCGCTATAGCCGTTATTTTGACTAG                                  6101          .         .         .         .         :         .         .         .         .         2 6200  1 NC_007919.3     100.0%       AGCCGAAGCAGAACAAAAATTGTGCGAATTAGATTACTCGATCCAGTTGACGAATATTAGTAAAGAGAAATTCCACTTCCCTCTTATTACGAAATATTTG       2 Nora_Median-Nor  96.8%       AACCGAAGCAGAACAAAAATTGTGCGAATTAGATTATTCGATCCAGTTGACGAATATTAGTAAAGAGAAATTCCACTTCCCTCTTATTACGAAATATTTG       3 Nora_raw_reads   96.7%       AACCGAAGCAGAACAAAAATTGTGCGAATTAGATTATTCGATCCAGTTGACGAATATTAGTAAAGAGAAATTCCACTTCCCTCTTATTACGAAATATTTG       4 JX220408.1       96.9%       AACCGAAGCAGAACAAAAATTGTGCGAATTAGATTATTCGATCCAGTTGACGAATATTAGTAAAGAGAAATTCCACTTCCCTCTTATTACGAAATATTTG       5 Nora_MV          96.6%       AACCGAAGCAGAACAAAAATTGTGCGAATTAGATTATTCGATCCAGTTGACGAATATTAGTAAAGAGAAATTCCACTTCCCTCTTATTACGAAATATTTG                                  6201          .         .         .         .         :         .         .         .         .         3 6300  1 NC_007919.3     100.0%       TTAACCGCTGACCAGTCAGAAGTAGAATGGATGAATTGTACGACCCTACATTATTTTGCAAAAAATAGAACCGTGGGGTTAGGGAGAACAACATCATTCC       2 Nora_Median-Nor  96.8%       TTAACCGCTGACCAGTCAGAAGTAGAATGGATGAATTGTACGACCCTACATTATTTTGCAAAGAATAGAACCGTGGGGTTAGGAAGAACAACATCATTCC       3 Nora_raw_reads   96.7%       TTAACCGCTGACCAGTCAGAAGTAGAATGGATGAATTGTACGACCCTACATTATTTTGCAAAGAATAGAACCGTGGGGTTAGGAAGAACAACATCATTCC       4 JX220408.1       96.9%       TTAACCGCTGACCAGTCAGAAGTAGAATGGATGAATTGTACGACCCTACATTATTTTGCAAAGAATAGAACCGTGGGGTTAGGAAGAACAACATCATTCC       5 Nora_MV          96.6%       TTAACCGCTGACCAGTCAGAAGTAGAATGGATGAATTGTACGACCCTACATTATTTTGCAAAGAATAGAACCGTGGGGTTAGGAAGAACAACATCATTCC                                  6301          .         .         .         .         :         .         .         .         .         4 6400  1 NC_007919.3     100.0%       AAGTTTCCGAATTTTTATGTGGAAACGAGTACATATCTAAGAAGCTGGTCGCATGCGCGCAAGGACTCCAGTCAAGTGTCGAACTAAGCCGACTTGGTGA       2 Nora_Median-Nor  96.8%       AAGTTTCTGAATTTCTATGTGGAAACGAGTACATATCCAAGAAACTGGTCGCATGCGCGCAAGGACTCCAGTCAAGTGTCGAACTAAGCCGACTTGGTGA       3 Nora_raw_reads   96.7%       AAGTTTCTGAATTTCTATGTGGAAACGAGTACATATCCAAGAAACTGGTCGCATGCGCGCAAGGACTCCAGTCAAGTGTCGAACTAAGCCGACTTGGTGA       4 JX220408.1       96.9%       AAGTTTCTGAATTTCTATGTGGAAACGAGTACATATCCAAGAAACTGGTCGCATGCGCGCAAGGACTCCAGTCAAGTGTCGAACTAAGCCGACTTGGTGA       5 Nora_MV          96.6%       AAGTTTCTGAATTTCTATGTGGAAACGAGTACATATCCAAGAAACTGGTCGCATGCGCGCAAGGACTCCAGTCAAGTGTCGAACTAAGCCGACTTGGTGA                                  6401          .         .         .         .         :         .         .         .         .         5 6500  1 NC_007919.3     100.0%       TTGTGGAAGTCCAATTGTCTTGGCGTCGGGAAAGAAGGCAGGAAAACTGATAGGTTTTCACGGTTATCACTCTCCAAATCTACAAACGTGGTATGGAGCA       2 Nora_Median-Nor  96.8%       TTGTGGAAGTCCAATTGTCTTGGCGTCGGGAAAGAAGGCAGGAAAACTGATAGGTTTTCACGGTTATCACTCTCCAAATCTACAAACGTGGTATGGAGCA       3 Nora_raw_reads   96.7%       TTGTGGAAGTCCAATTGTCTTGGCGTCGGGAAAGAAGGCAGGAAAACTGATAGGTTTTCACGGTTATCACTCTCCAAATCTACAAACGTGGTATGGAGCA       4 JX220408.1       96.9%       TTGTGGAAGTCCAATTGTCTTGGCGTCGGGAAAGAAGGCAGGAAAACTGATAGGTTTTCACGGTTATCACTCTCCAAATCTACAAACGTGGTATGGAGCA       5 Nora_MV          96.6%       TTGTGGAAGTCCAATTGTCTTGGCGTCGGGAAAGAAGGCAGGAAAACTGATAGGTTTTCACGGTTATCACTCTCCAAATCTACAAACGTGGTATGGAGCA                                  6501          .         .         .         .         :         .         .         .         .         6 6600  1 NC_007919.3     100.0%       ATGTTGACTGTTGAGGACTTGGGAATCATCAACGGCGTAGAAGAACATTTTGATGACCCATGGGCCAAACTTATCACACAAGGATTACCTGTCGATTTGC       2 Nora_Median-Nor  96.8%       ATGTTGACTGTTGAGGACTTGGGAATCATCAACGGCGTAGAAGAACATTTTGATGACCCATGGGCCAAACTTATTACACAAGGATTACCTGTCGATTTGC       3 Nora_raw_reads   96.7%       ATGTTGACTGTTGAGGACTTGGGAATCATCAACGGCGTAGAAGAACATTTTGATGACCCATGGGCCAAACTTATTACACAAGGATTACCTGTCGATTTGC       4 JX220408.1       96.9%       ATGTTGACTGTTGAGGACTTGGGAATCATCAACGGCGTAGAAGAACATTTTGATGACCCATGGGCCAAACTTATTACACAAGGATTACCTGTCGATTTGC       5 Nora_MV          96.6%       ATGTTGACTGTTGAGGACTTGGGAATCATCAACGGCGTAGAAGAACATTTTGATGACCCATGGGCCAAACTTATTACACAAGGATTACCTGTCGATTTGC                                  6601          .         .         .         .         :         .         .         .         .         7 6700  1 NC_007919.3     100.0%       CAATTGGACCAGAAGTTGAGTATGTTGGTAATCTAATAAGACCTAGTTTACCTGTGACAAATGACTCATTGGACCATTGGCACAAATCACCATTTGCTGA       2 Nora_Median-Nor  96.8%       CAATTGGACCAGAAGTTGAGTATGTTGGTAATCTAATAAGACCTAGTTTACCTGTGACAAATGACTCATTGGACCATTGGCACAAATCACCATTTGCTGA       3 Nora_raw_reads   96.7%       CAATTGGACCAGAAGTTGAGTATGTTGGTAATCTAATAAGACCTAGTTTACCTGTGACAAATGACTCATTGGACCATTGGCACAAATCACCATTTGCTGA       4 JX220408.1       96.9%       CAATTGGACCAGAAGTTGAGTATGTTGGTAATCTAATAAGACCTAGTTTACCTGTGACAAATGACTCATTGGACCATTGGCACAAATCACCATTTGCTGA       5 Nora_MV          96.6%       CAATTGGACCAGAAGTTGAGTATGTTGGTAATCTAATAAGACCTAGTTTACCTGTGACAAATGACTCATTGGACCATTGGCACAAATCACCATTTGCTGA                                  6701          .         .         .         .         :         .         .         .         .         8 6800  1 NC_007919.3     100.0%       TCAATTTGAAGAACAATTAGCACCTGGTCGATTGAATCCATATGATTCATATATTGAAGGAGATTTGCCAACTAATCTTGAGGGCCGAAAAAGTTTAATC       2 Nora_Median-Nor  96.8%       TCAATTTGAAGAACAACTAGCACCTGGTCGATTGAATCCATATGATTCATATATTGAAGGAGATTTGCCAACTAATCTTGAGGGCCGAAAAAGTTTAATC       3 Nora_raw_reads   96.7%       TCAATTTGAAGAACAACTAGCACCTGGTCGATTGAATCCATATGATTCATATATTGAAGGAGATTTGCCAACTAATCTTGAGGGCCGAAAAAGTTTAATC       4 JX220408.1       96.9%       TCAATTTGAAGAACAACTAGCACCTGGTCGATTGAATCCATATGATTCATATATTGAAGGAGATTTGCCAACTAATCTTGAGGGCCGAAAAAGTTTAATC       5 Nora_MV          96.6%       TCAATTTGAAGAACAACTAGCACCTGGTCGATTGAATCCATATGATTCATATATTGAAGGAGATTTGCCAACTAATCTTGAGGGCCGAAAAAGTTTAATC                                  6801          .         .         .         .         :         .         .         .         .         9 6900  1 NC_007919.3     100.0%       TTAGGCCCGAATAGTGAAATGGCAAAAACTCTTCCAGAATTGGATCAAGGAATTATCGACTGGATTGTAGATCAGTTGGTGGTGGAACAAGTTGCAACTT       2 Nora_Median-Nor  96.8%       TTAGGCCCGAATAGTGAAATGGCAAAAACTCTTCCAGAATTGGATCAAGGAATTATCGACTGGATTGTAGATCAGTTGGTGGTGGAACAAGTTGCAACTT       3 Nora_raw_reads   96.7%       TTAGGCCCGAATAGTGAAATGGCAAAAACTCTTCCAGAATTGGATCAAGGAATTATCGACTGGATTGTAGATCAGTTGGTGGTGGAACAAGTTGCAACTT       4 JX220408.1       96.9%       TTAGGCCCGAATAGTGAAATGGCAAAAACTCTTCCAGAATTGGATCAAGGAATTATCGACTGGATTGTAGATCAGTTGGTGGTGGAACAAGTTGCAACTT       5 Nora_MV          96.6%       TTAGGCCCGAATAGTGAAATGGCAAAAACTCTTCCAGAATTGGATCAAGGAATTATCGACTGGATTGTAGATCAGTTGGTGGTGGAACAAGTTGCAACTT                                  6901          .         .         .         .         :         .         .         .         .         0 7000  1 NC_007919.3     100.0%       TCAAAGCAGAAAACCTTTTAACGAAAGTTAGTGACGATATTGACGAAATGCTTGATTATGCCCTGAATGGAAATGTAGATAACACATATGTTAGGGGTAT       2 Nora_Median-Nor  96.8%       TCAAAGCAGAAAACCTTTTAACGAAAGTTAGTGACGATATTGACGAAATGCTTGATTATGCCCTGAATGGAAATGTAGATAACACATATGTTAGGGGTAT       3 Nora_raw_reads   96.7%       TCAAAGCAGAAAACCTTTTAACGAAAGTTAGTGACGATATTGACGAAATGCTTGATTATGCCCTGAATGGAAATGTAGATAACACATATGTTAGGGGTAT       4 JX220408.1       96.9%       TCAAAGCAGAAAACCTTTTAACGAAAGTTAGTGACGATATTGACGAAATGCTTGATTATGCCCTGAATGGAAATGTAGATAACACATATGTTAGGGGTAT       5 Nora_MV          96.6%       TCAAAGCAGAAAACCTTTTAACGAAAGTTAGTGACGATATTGACGAAATGCTTGATTATGCCCTGAATGGAAATGTAGATAACACATATGTTAGGGGTAT                                  7001          .         .         .         .         :         .         .         .         .         1 7100  1 NC_007919.3     100.0%       GGAAGTCAACAAAGCATCAGGATTACCTTGGAGTCTTTCAGGTAGTCCGAAAAAGAGTGACTTTATCGATGTAGATGAAGCTACTGGAGTCAGATCGTTT       2 Nora_Median-Nor  96.8%       GGAAGTCAACAAAGCATCAGGATTACCTTGGAGTCTTTCAGGTAGTCCGAAAAAGAGTGACTTTATCGATGTAGATGAAGCTACTGGAGTCAGATCGTTT       3 Nora_raw_reads   96.7%       GGAAGTCAACAAAGCATCAGGATTACCTTGGAGTCTTTCAGGTAGTCCGAAAAAGAGTGACTTTATCGATGTAGATGAAGCTACTGGAGTCAGATCGTTT       4 JX220408.1       96.9%       GGAAGTCAACAAAGCATCAGGATTACCTTGGAGTCTTTCAGGTAGTCCGAAAAAGAGTGACTTTATCGATGTAGATGAAGCTACTGGAGTCAGATCGTTT       5 Nora_MV          96.6%       GGAAGTCAACAAAGCATCAGGATTACCTTGGAGTCTTTCAGGTAGTCCGAAAAAGAGTGACTTTATCGATGTAGATGAAGCTACTGGAGTCAGATCGTTT                                  7101          .         .         .         .         :         .         .         .         .         2 7200  1 NC_007919.3     100.0%       AAGGTTAATGCTAATGGAGACGCTCTTAAAAATAGAGTTATCCTGAAGTTGCAACAGGCGAAGATGGGAAATAGAATCTTGAGTTTTTCAAGTTCGAAAT       2 Nora_Median-Nor  96.8%       AAGGTTAATGCTAATGGAGACGCTCTTAAAAATAGAGTTATCCTGAAGTTGCAACAGGCGAAGATGGGAAATAGAATCTTGAGTTTTTCAAGTTCGAAAC       3 Nora_raw_reads   96.7%       AAGGTTAATGCTAATGGAGACGCTCTTAAAAATAGAGTTATCCTGAAGTTGCAACAGGCGAAGATGGGAAATAGAATCTTGAGTTTTTCAAGTTCGAAAC       4 JX220408.1       96.9%       AAGGTTAATGCTAATGGAGACGCTCTTAAAAATAGAGTTATCCTGAAGTTGCAACAGGCGAAGATGGGAAATAGAATCTTGAGTTTTTCAAGTTCGAAAC       5 Nora_MV          96.6%       AAGGTTAATGCTAATGGAGACGCTCTTAAAAATAGAGTTATCCTGAAGTTGCAACAGGCGAAGATGGGAAATAGAATCTTGAGTTTTTCAAGTTCGAAAC                                  7201          .         .         .         .         :         .         .         .         .         3 7300  1 NC_007919.3     100.0%       TAAAAGACCAACCCATCAAAATAGCGCAAGCGAAGAGTGGAAGGACGAGAGTATTCCATTGTATCCCAGTGGATTTAATCTTGTTTTCGGGAGCGCTGTA       2 Nora_Median-Nor  96.8%       TAAAGGACCAACCCATCAAAATAGCGCAAGCGAAGAGTGGAAGGACGAGAGTATTCCATTGTATCCCAGTGGACTTAATCTTGTTTTCGGGAGCGCTGTA       3 Nora_raw_reads   96.7%       TAAAGGACCAACCCATCAAAATAGCGCAAGCGAAGAGTGGAAGGACGAGAGTATTCCATTGTATCCCAGTGGACTTAATCTTGTTTTCGGGAGCGCTGTA       4 JX220408.1       96.9%       TAAAGGACCAACCCATCAAAATAGCGCAAGCGAAGAGTGGAAGGACGAGAGTATTCCATTGTATCCCAGTGGACTTAATCTTGTTTTCGGGAGCGCTGTA       5 Nora_MV          96.6%       TAAAGGACCAACCCATCAAAATAGCGCAAGCGAAGAGTGGAAGGACGAGAGTATTCCATTGTATCCCAGTGGACTTAATCTTGTTTTCGGGAGCGCTGTA                                  7301          .         .         .         .         :         .         .         .         .         4 7400  1 NC_007919.3     100.0%       CGGCCCGTACAAAGAAGCATACACAAAGGCTGGATTGAAATGCTATCATGCTGTAGGAATAGATCCGAAATCAGTTGGTTGGCAACAGTTGGCTACGTAT       2 Nora_Median-Nor  96.8%       CGGTCCGTACAAAGAAGCATACACAAAGGCTGGACTGAAATGCTATCATGCTGTAGGAATAGATCCGAAATCAGTTGGTTGGCAACAGTTGGCTACGTAT       3 Nora_raw_reads   96.7%       CGGCCCGTACAAAGAAGCATACACAAAGGCTGGACTGAAATGCTATCATGCTGTAGGAATAGATCCGAAATCAGTTGGTTGGCAACAGTTGGCTACGTAT       4 JX220408.1       96.9%       CGGCCCGTACAAAGAAGCATACACAAAGGCTGGACTGAAATGCTATCATGCTGTAGGAATAGATCCGAAATCAGTTGGTTGGCAACAGTTGGCTACGTAT       5 Nora_MV          96.6%       CGGCCCGTACAAAGAAGCATACACAAAGGCTGGACTGAAATGCTATCATGCTGTAGGAATAGATCCGAAATCAGTTGGTTGGCAACAGTTGGCTACGTAT                                  7401          .         .         .         .         :         .         .         .         .         5 7500  1 NC_007919.3     100.0%       ATGACGAAGCATCCAAATTATTTTGATGCTGATTATAAGAATTACGATAAGTATTTGCATAGGCAGGTATTTAAAGCAGTTCGAAAAATTCAGCGATCAG       2 Nora_Median-Nor  96.8%       ATGACGAAGCATCCTAATTATTTTGATGCTGATTATAAGAATTACGATAAGTATTTGCATAGGCAGGTATTTAAAGCAGTTCGAAAAATTCAGCGATCAG       3 Nora_raw_reads   96.7%       ATGACGAAGCATCCTAATTATTTTGATGCTGATTATAAGAATTACGATAAGTATTTGCATAGGCAGGTATTTAAAGCAGTTCGAAAAATTCAGCGATCAG       4 JX220408.1       96.9%       ATGACGAAGCATCCTAATTATTTTGATGCTGATTATAAGAATTACGATAAGTATTTGCATAGGCAGGTATTTAAAGCAGTTCGAAAAATTCAGCGATCAG       5 Nora_MV          96.6%       ATGACGAAGCATCCTAATTATTTTGATGCTGATTATAAGAATTACGATAAGTATTTGCATAGGCAGGTATTTAAAGCAGTTCGAAAAATTCAGCGATCAG                                  7501          .         .         .         .         :         .         .         .         .         6 7600  1 NC_007919.3     100.0%       TGATTCAACAGATGTGCCCAGATAAATGGGATAAAGCAAGAGCTGTTGAAGAATTAGATGCTATTGACACGTATGTAGTCGACTATCAAACAGTCTACAA       2 Nora_Median-Nor  96.8%       TGATTCAACAGATGTGCCCAGATAAATGGGATAAAGCAAGAGCTGTTGAAGAATTAGATGCTATTGACACGTATGTAGTTGACTATCAAACAGTCTACAA       3 Nora_raw_reads   96.7%       TGATTCAACAGATGTGCCCAGATAAATGGGATAAAGCAAGAGCTGTTGAAGAATTAGATGCTATTGACACGTATGTAGTTGACTATCAAACAGTCTACAA       4 JX220408.1       96.9%       TGATTCAACAGATGTGCCCAGATAAATGGGATAAAGCAAGAGCTGTTGAAGAATTAGATGCTATTGACACGTATGTAGTTGACTATCAAACAGTCTACAA       5 Nora_MV          96.6%       TGATTCAACAGATGTGCCCAGATAAATGGGATAAAGCAAGAGCTGTTGAAGAATTAGATGCTATTGACACGTATGTAGTTGACTATCAAACAGTCTACAA                                  7601          .         .         .         .         :         .         .         .         .         7 7700  1 NC_007919.3     100.0%       AACCAATCGTGGCAACAAAAGTGGTAGTTATACTACTACAATTGATAATTGCCTGGCGAACGATATTTATGGTTTGTATGCGTGGGTGAAGACAACCGGC       2 Nora_Median-Nor  96.8%       AACCAATCGTGGCAACAAAAGTGGTAGTTATACTACTACAATTGATAATTGCCTGGCGAACGATATTTATGGTTTGTATGCGTGGGTGAAGACAACCGGC       3 Nora_raw_reads   96.7%       AACCAATCGTGGCAACAAAAGTGGTAGTTATACTACTACAATTGATAATTGCCTGGCGAACGATATTTATGGTTTGTATGCGTGGGTGAAGACAACCGGC       4 JX220408.1       96.9%       AACCAATCGTGGCAACAAAAGTGGTAGTTATACTACTACAATTGATAATTGCCTGGCGAACGATATTTATGGTTTGTATGCGTGGGTGAAGACAACCGGC       5 Nora_MV          96.6%       AACCAATCGTGGCAACAAAAGTGGTAGTTATACTACTACAATTGATAATTGCCTGGCGAACGATATTTATGGTTTGTATGCGTGGGTGAAGACAACCGGC                                  7701          .         .         .         .         :         .         .         .         .         8 7800  1 NC_007919.3     100.0%       CTAAGATCGCTGTGGGATTACCGACAGAATGTCTCGAGCGTTGCGTTTGGTGATGATATTATAAAAAGCGTTAGCGATGAGTATAAAGATAAGTATAATT       2 Nora_Median-Nor  96.8%       CTAAGATCACTGTGGGATTACCGACAGAATGTCTCGAGCGTCGCGTTTGGTGATGATATTATAAAAAGCGTTAGCGATGAGTATAAGGATAAGTATAATT       3 Nora_raw_reads   96.7%       CTAAGATCACTGTGGGATTACCGACAGAATGTCTCGAGCGTCGCGTTTGGTGATGATATTATAAAAAGCGTTAGCGATGAGTATAAGGATAAGTATAATT       4 JX220408.1       96.9%       CTAAGATCACTGTGGGATTACCGACAGAATGTCTCGAGCGTCGCGTTTGGTGATGATATTATAAAAAGCGTTAGCGATGAGTATAAGGATAAGTATAATT       5 Nora_MV          96.6%       CTAAGATCACTGTGGGATTACCGACAGAATGTCTCGAGCGTCGCGTTTGGTGATGATATTATAAAAAGCGTTAGCGATGAGTATAAGGATAAGTATAATT                                  7801          .         .         .         .         :         .         .         .         .         9 7900  1 NC_007919.3     100.0%       ATTGTACTTACCGAGATGTATTAAATGCTACAGGACATATCATGACACCAGGTTCAAAGGATGGAGAAGAGAAGCCATTTACTTCTTTCGAAAACTTACA       2 Nora_Median-Nor  96.8%       ATTGTACTTATCGAGATGTACTAAATGCTACAGGACATATCATGACACCAGGTTCAAAGGATGGAGAAGAGAAGCCATTTACTTCTTTTGAAAACCTACA       3 Nora_raw_reads   96.7%       ATTGTACTTATCGAGATGTACTAAATGCTACAGGACATATCATGACACCAGGTTCAAAGGATGGAGAAGAGAAGCCATTTACTTCTTTTGAAAACCTACA       4 JX220408.1       96.9%       ATTGTACTTATCGAGATGTACTAAATGCTACAGGACATATCATGACACCAGGTTCAAAGGATGGAGAAGAGAAGCCATTTACTTCTTTTGAAAACCTACA       5 Nora_MV          96.6%       ATTGTACTTATCGAGATGTACTAAATGCTACAGGACATATCATGACACCAGGTTCAAAGGATGGAGAAGAGAAGCCATTTACTTCTTTTGAAAACCTACA                                  7901          .         .         .         .         :         .         .         .         .         0 8000  1 NC_007919.3     100.0%       ATTCTTGAAAAGAGGGTTTAAGTTGGAAAACGGTATGGTTTTAGCTCCATTGCTACAACGATCTATTGAAGGACCGTTTGTATGGACTGATATCCGCGAA       2 Nora_Median-Nor  96.8%       ATTCTTGAAAAGAGGATTTAAGTTAGAAAGCGGTATGGTTTTAGCTCCATTACTACAACGATCTATTGAAGGACCGTTTGTATGGACTGATATCCGCGAA       3 Nora_raw_reads   96.7%       ATTCTTGAAAAGAGGATTTAAGTTAGAAAACGGTATGGTTTTAGCTCCATTACTACAACGATCTATTGAAGGACCGTTTGTATGGACTGATATCCGCGAA       4 JX220408.1       96.9%       ATTCTTGAAAAGAGGATTTAAGTTAGAAAACGGTATGGTTTTAGCTCCATTACTACAACGATCTATTGAAGGACCGTTTGTATGGACTGATATCCGCGAA       5 Nora_MV          96.6%       ATTCTTGAAAAGAGGATTTAAGTTAGAAAACGGTATGGTTTTAGCTCCATTACTACAACGATCTATTGAAGGACCGTTTGTATGGACTGATATCCGCGAA                                  8001          .         .         .         .         :         .         .         .         .         1 8100  1 NC_007919.3     100.0%       GATCAGATAACTGTGTGGGTAAATCTCGTGCAAGAACAGTTGATCGAAGCCGCTCTTTGGGGTGAGGAATATTACAATGAGCTTTGTCAAAAGCTAAAAT       2 Nora_Median-Nor  96.8%       GATCAGATAACTGTGTGGGTAAATCTCGTGCAAGAACAGTTGATCGAAGCCGCTCTTTGGGGTGAGGAGTATTACAATGAGCTTTGTCAAAAGCTAAAAT       3 Nora_raw_reads   96.7%       GATCAGATAACTGTGTGGGTAAATCTCGTGCAAGAACAGTTGATCGAAGCCGCTCTTTGGGGTGAGGAGTATTACAATGAGCTTTGTCAAAAGCTAAAAT       4 JX220408.1       96.9%       GATCAGATAACTGTGTGGGTAAATCTCGTGCAAGAACAGTTGATCGAAGCCGCTCTTTGGGGTGAGGAGTATTACAATGAGCTTTGTCAAAAGCTAAAAT       5 Nora_MV          96.6%       GATCAGATAACTGTGTGGGTAAATCTCGTGCAAGAACAGTTGATCGAAGCCGCTCTTTGGGGTGAGGAGTATTACAATGAGCTTTGTCAAAAGCTAAAAT                                  8101          .         .         .         .         :         .         .         .         .         2 8200  1 NC_007919.3     100.0%       GTGGTACAAATAGAACCTTGAATGGAGCATTAGCAGTATTGTTGAATACGAGCTGGGAAGTTACTTTCCAAAAATTCTGTAATCGTTATTATGGCATTAA       2 Nora_Median-Nor  96.8%       GTGGTACAAATAGAACCTTGAATGGAGCATTAGCAGTATTGTTGAATACGAGCTGGGAAGTTACTTTCCAAAAATTCTGTAATCGTTATTATGGCATTAA       3 Nora_raw_reads   96.7%       GTGGTACAAATAGAACCTTGAATGGAGCATTAGCAGTATTGTTGAATACGAGCTGGGAAGTTACTTTCCAAAAATTCTGTAATCGTTATTATGGCATTAA       4 JX220408.1       96.9%       GTGGTACAAATAGAACCTTGAATGGAGCATTAGCAGTATTGTTGAATACGAGCTGGGAAGTTACTTTCCAAAAATTCTGTAATCGTTATTATGGCATTAA       5 Nora_MV          96.6%       GTGGTACAAATAGAACCTTGAATGGAGCATTAGCAGTATTGTTGAATACGAGCTGGGAAGTTACTTTCCAAAAATTCTGTAATCGTTATTATGGCATTAA                                  8201          .         .         .         .         :         .         .         .         .         3 8300  1 NC_007919.3     100.0%       AAGAGGAGATTTTTGATCAAAACACCACTCTTTTCGCCGTTTTAGACGAGAACGAGGTTACTGAGATTAAGTCGATTCAAGCCTCAGTAACAGCAGTAAA       2 Nora_Median-Nor  96.8%       AAGAGGAGATCTTTGATCAGAACACCACTCTTTTCACCGTTTTAGACGAGAACGAGGTTACTGAGATTAAGTCAATTCAATCTTCAGTAACAGCAGTAAA       3 Nora_raw_reads   96.7%       AAGAGGAGATCTTTGATCAGAACACCACTCTTTTCACCGTTTTAGACGAGAACGAGGTTACTGAGATTAAGTCAATTCAATCTTCAGTAACAGCAGTAAA       4 JX220408.1       96.9%       AAGAGGAGATCTTTGATCAGAACACCACTCTTTTCACCGTTTTAGACGAGAACGAGGTTACTGAGATTAAGTCAATTCAATCTTCAGTAACAGCAGTAAA       5 Nora_MV          96.6%       AAGAGGAGATCTTTGATCAGAACACCACTCTTTTCACCGTTTTAGACGAGAACGAGGTTACTGAGATTAAGTCAATTCAATCTTCAGTAACAGCAGTAAA                                  8301          .         .         .         .         :         .         .         .         .         4 8400  1 NC_007919.3     100.0%       GACCCAGCTCGATCAACAAAAACTGCAACTTGATGGTTTAGCTAAAGTAGTTGATAACAATCAAGCTCGAAATGAAGAACAATTCGTTAATATCAATACA       2 Nora_Median-Nor  96.8%       GACCCAGCTCGATCAACAAAAACTGCAACTTGACGGTTTAGCTAAAGTAGTTGACAACAATCAAGCTCGAAATGAAGAACAATTCGTTAATATCAATACA       3 Nora_raw_reads   96.7%       GACCCAGCTCGATCAACAAAAACTGCAACTTGACGGTTTAGCTAAAGTAGTTGACAACAATCAAGCTCGAAATGAAGAACAATTCGTTAATATCAATACA       4 JX220408.1       96.9%       GACCCAGCTCGATCAACAAAAACTGCAACTTGACGGTTTAGCTAAAGTAGTTGACAACAATCAAGCTCGAAATGAAGAACAATTCGTTAATATCAATACA       5 Nora_MV          96.6%       GACCCAGCTCGATCAACAAAAACTGCAACTTGACGGTTTAGCTAAAGTAGTTGACAACAATCAAGCTCGAAATGAAGAACAATTCGTTAATATCAATACA                                  8401          .         .         .         .         :         .         .         .         .         5 8500  1 NC_007919.3     100.0%       ACATTGGTAGAAATGAGTTTAGAAGTTGATAAATTAACCACAACAACGAGTCAACAGGCAAAGCAGATTAACACCTTTGCCACAGCATTAAACGAGCTTG       2 Nora_Median-Nor  96.8%       ACATTGGTAGAAATGAATTCAGAAGTTGACAAATTAACCACAACAACAAGTCAACAGGCAAAACAGATTAACACCCTTGCCACAACATTAAACGAGCTTG       3 Nora_raw_reads   96.7%       GCATTGGTAGAAATGAATTCAGAAGTTGACAAATTAACCACAACAACAAGTCAACAGGCAAAACAGATTAACACCCTTGCCACAACATTAAACGAGCTTG       4 JX220408.1       96.9%       GCATTGGTAGAAATGAATTCAGAAGTTGACAAATTAACCACAACAACAAGTCAACAGGCAAAACAGATTAACACCCTTGCCACAACATTAAACGAGCTTG       5 Nora_MV          96.6%       GCATTGGTAGAAATGAATTCAGAAGTTGACAAATTAACCACAACAACAAGTCAACAGGCAAAACAGATTAACACCCTTGCCACAACATTAAACGAGCTTG                                  8501          .         .         .         .         :         .         .         .         .         6 8600  1 NC_007919.3     100.0%       ATCAAACTACGAGAGACTCCTTAGACACGTTGAACACAACAACAGAATCTCTTACAAAACAAGTATTATTTAATACTGATGAGATTACCGTGTTAAAGGT       2 Nora_Median-Nor  96.8%       ATCAAACTACGAAAGACTCCTTAGACACGTTGAACACAACAACAGAATCTCTTAGTAAACAAGTATTATTTAATACTGATGAGATTACCGTGTTAAAGGT       3 Nora_raw_reads   96.7%       ATCAAACTACGAAAGACTCCTTAGACACGTTGAACACAACAACAGAATCTCTTAGTAAACAAGTATTATTTAATACTGATGAGATTACCGTGTTAAAGGT       4 JX220408.1       96.9%       ATCAAACTACGAAAGACTCCTTAGACACGTTGAACACAACAACAGAATCTCTTAGTAAACAAGTATTATTTAATACTGATGAGATTACCGTGTTAAAGGT       5 Nora_MV          96.6%       ATCAAACTACGAAAGACTCCTTAGACACGTTGAACACAACAACAGAATCTCTTAGTAAACAAGTATTATTTAATACTGATGAGATTACCGTGTTAAAGGT                                  8601          .         .         .         .         :         .         .         .         .         7 8700  1 NC_007919.3     100.0%       AGATGTAGCAACCGTCACACAAAAACAACAAGACGTAGAACATTCACTTGAGACGATGAAAGATGAGATAGGAGAATTGCACGTATCAGTGAACGCCAAT       2 Nora_Median-Nor  96.8%       AGACGTAGCAACCGTCACACAAAAACAACAGGACGTAGAACATTCACTTGTGACAATGAAAGATGAGATAGGAGAATTGCACATATCAGTGAATGCCAAT       3 Nora_raw_reads   96.7%       AGACGTAGCAACCGTCACACAAAAACAACAGGACGTAGAACATTCACTTGTGACAATGAAAGATGAGATAGGAGAATTGCACATATCAGTGAATGCCAAT       4 JX220408.1       96.9%       AGACGTAGCAACCGTCACACAAAAACAACAGGACGTAGAACATTCACTTGTGACAATGAAAGATGAGATAGGAGAATTGCACATATCAGTGAATGCCAAT       5 Nora_MV          96.6%       AGACGTAGCAACCGTCACACAAAAACAACAGGACGTAGAACATTCACTTGTGACAATGAAAGATGAGATAGGAGAATTGCACATATCAGTGAATGCCAAT                                  8701          .         .         .         .         :         .         .         .         .         8 8800  1 NC_007919.3     100.0%       GTTAACTCCATTGAGGCATTACGCACCAGAATTGCCGCGCTAGAGATTAGAGATGTAGGACCGTGGGTTTTGAAAGATAGAATTTACAAATTCGTGATCA       2 Nora_Median-Nor  96.8%       GCTAACTCCATTGAGGCATTACGCACCAGAATTGCCGCGCTAGAGGTTAGAGATGTAGGACCGTGGGTTTTGAAAAACAGAATTTACAAATTCGTGATCA       3 Nora_raw_reads   96.7%       GCTAACTCCATTGAGGCATTACGCACCAGAATTGCCGCGCTAGAGGTTAGAGATGTAGGACCGTGGGTTTTGAAAAACAGAATTTACAAATTCGTGATCA       4 JX220408.1       96.9%       GCTAACTCCATTGAGGCATTACGCACCAGAATTGCCGCGCTAGAGGTTAGAGATGTAGGACCGTGGGTTTTGAAAAACAGAATTTACAAATTCGTGATCA       5 Nora_MV          96.6%       GCTAACTCCATTGAGGCATTACGCACCAGAATTGCCGCGCTAGAGGTTAGAGATGTAGGACCGTGGGTTTTGAAAAACAGAATTTACAAATTCGTGATCA                                  8801          .         .         .         .         :         .         .         .         .         9 8900  1 NC_007919.3     100.0%       ACAAGCCAAATGGAACTACTCGCTATACCACAATATACTTTTTCGCCGACGTATATTATAGCACTGGAGTAAGAGCAGCACCTACAAATTCGGGAACAGC       2 Nora_Median-Nor  96.8%       ATATGCCAAATGGAACTACTCGCTATACTACAATATACTTTTTCGCCGATGTATATTATAGCACTGGAGTAAGAGCAGCACCTACAAATGCGGGAACAAC       3 Nora_raw_reads   96.7%       ATATGCCAAATGGAACTACTCGCTATACTACAATATACTTTTTCGCCGATGTATATTATAGCACTGGAGTAAGAGCAGCACCTACAAATGCGGGAACAAC       4 JX220408.1       96.9%       ATATGCCAAATGGAACTACTCGCTATACTACAATATACTTTTTCGCCGATGTATATTATAGCACTGGAGTAAGAGCAGCACCTACAAATGCGGGAACAAC       5 Nora_MV          96.6%       ATATGCCAAATGGAACTACTCGCTATACTACAATATACTTTTTCGCCGATGTATATTATAGCACTGGAGTAAGAGCAGCACCTACAAATGCGGGAACAAC                                  8901          .         .         .         .         :         .         .         .         .         0 9000  1 NC_007919.3     100.0%       TACAAGTATATTAACGATTACTTCGTTGACAACATCATATAGTTTGGCTAACGTTCCCGTTTTAAAAGGTGTTCCTTATAGAGTTAATGGTTACTTTGCT       2 Nora_Median-Nor  96.8%       TACAAGCATATTGACGATTACTTCGTTGACAACATCATATAGTTTGGCTAACGTCCCCGTTTTAAAAGGTGTACCTTATAGAGTCAATGGTTACTTTGCT       3 Nora_raw_reads   96.7%       TACAAGCATATTGACGATTACTTCGTTGACAACATCATATAGTTTGGCTAACGTCCCCGTTTTAAAAGGTGTACCTTATAGAGTCAATGGTTACTTTGCT       4 JX220408.1       96.9%       TACAAGCATATTGACGATTACTTCGTTGACAACATCATATAGTTTGGCTAACGTCCCCGTTTTAAAAGGTGTACCTTATAGAGTCAATGGTTACTTTGCT       5 Nora_MV          96.6%       TACAAGCATATTGACGATTACTTCGTTGACAACATCATATAGTTTGGCTAACGTCCCCGTTTTAAAAGGTGTACCTTATAGAGTCAATGGTTACTTTGCT                                  9001          .         .         .         .         :         .         .         .         .         1 9100  1 NC_007919.3     100.0%       AACGGAAACAATATCGAAGATATAACTGGAAGCACATCAGTAATTTATGACTCTATGTAAAAACCGGACATGACATGTCTATGACACGACATTAAACTGT       2 Nora_Median-Nor  96.8%       AACGGAAATAGTATCGAAGATATAACCGGAAGCACGTCAGTGATTTACGACTCTATGT--AAACCGGACATGACATGTCTATGACACGACATTAAACTGT       3 Nora_raw_reads   96.7%       AACGGAAATAGTATCGAAGATATAACCGGAAGCACGTCAGTGATTTACGACTCTATGT--AAACCGGACATGACATGTCTATGACACGACATTAAACTGT       4 JX220408.1       96.9%       AACGGAAATAGTATCGAAGATATAACCGGAAGCACGTCAGTGATTTACGACTCTATGTAAAAACCGGACATGACATGTCTATGACACGACATTAAACTGT       5 Nora_MV          96.6%       AACGGAAATAGTATCGAAGATATAACCGGAAGCACGTCAGTGATTTACGACTCTATGT--AAACCGGACATGACATGTCTATGACACGACATTAAACTGT                                  9101          .         .         .         .         :         .         .         .         .         2 9200  1 NC_007919.3     100.0%       CAGAACCTGTTTGGTTAACACAGAGAGATTAACCGCAAGACGAATATGCAGAATCCAACACAAACCATGCATATATATGACATGCCTCTACGCGTCATCG       2 Nora_Median-Nor  96.8%       CAGAACCTGTTTGGTTAACACAGAGAGATTAACCGCAAGACGAGTATGCAGAATCCAACACAAACCATGCATATATACGACATGCCCCTACGCGTCATCG       3 Nora_raw_reads   96.7%       CAGAACCTGTTTGGTTAACACAGAGAGATTAACCGCAAGACGAGTATGCAGAATCCAACACAAACCATGCATATATACGACATGCCCCTACGCGTCATCG       4 JX220408.1       96.9%       CAGAACCTGTTTGGTTAACACAGAGAGATTAACCGCAAGACGAGTATGCAGAATCCAACACAAACCATGCATATATACGACATGCCCCTACGCGTCATCG       5 Nora_MV          96.6%       CAGAACCTGTTTGGTTAACACAGAGAGATTAACCGCAAGACGAGTATGCAGAATCCAACACAAACCATGCATATATACGACATGCCCCTACGCGTCATCG                                  9201          .         .         .         .         :         .         .         .         .         3 9300  1 NC_007919.3     100.0%       CTGGCCTGTCAACCCTTGCCAAAACAACCGAAGAAGACGACAACACCTCAACTGGAATAGTAGTTAGTGAAGTAGGAGAGCCACAAGTGGTTAACCATCC       2 Nora_Median-Nor  96.8%       CTGGCCTGTCAACCCTTGCCAAAACAACTGAAGAAGACGACAACACCTCAACTGGAATAGTAGTTAGTGAAGTAGGAGAGCCACAAGTGGTCGACCATCC       3 Nora_raw_reads   96.7%       CTGGCCTGTCAACCCTTGCCAAAACAACTGAAGAAGACGACAACACCTCAACTGGAATAGTAGTTAGTGAAGTAGGAGAGCCACAAGTGGTCGACCATCC       4 JX220408.1       96.9%       CTGGCCTGTCAACCCTTGCCAAAACAACTGAAGAAGACGACAACACCTCAACTGGAATAGTAGTTAGTGAAGTAGGAGAGCCACAAGTGGTCGACCATCC       5 Nora_MV          96.6%       CTGGCCTGTCAACCCTTGCCAAAACAACTGAAGAAGACGACAACACCTCAACTGGAATAGTAGTTAGTGAAGTAGGAGAGCCACAAGTGGTCGACCATCC                                  9301          .         .         .         .         :         .         .         .         .         4 9400  1 NC_007919.3     100.0%       AGCATGGATTGATCCCTTTGTTGCTTACCAATTGCGAGCTCCACGTAAAAATATCACACCAGATTTTATATTTGGTCGAGCCGATATTGGTAATGCGTTT       2 Nora_Median-Nor  96.8%       AGCATGGATTGATCCCTTTGTTGCTTATCAATTGCGAGCTCCACGTAAAAACATCACACCAGATTTTATATTTGGTCGAGCCGATATTGGTAATGCGTTT       3 Nora_raw_reads   96.7%       AGCATGGATTGATCCCTTTGTTGCTTATCAATTGCGAGCTCCACGTAAAAACATCACACCAGATTTTATATTTGGTCGAGCCGATATTGGTAATGCGTTT       4 JX220408.1       96.9%       AGCATGGATTGATCCCTTTGTTGCTTATCAATTGCGAGCTCCACGTAAAAACATCACACCAGATTTTATATTTGGTCGAGCCGATATTGGTAATGCGTTT       5 Nora_MV          96.6%       AGCATGGATTGATCCCTTTGTTGCTTATCAATTGCGAGCTCCACGTAAAAACATCACACCAGATTTTATATTTGGTCGAGCCGATATTGGTAATGCGTTT                                  9401          .         .         .         .         :         .         .         .         .         5 9500  1 NC_007919.3     100.0%       AGTGCTTTCTTGCCACGCCGTTTTTCTGCTCCAGCAGTAGGAACCCGACTTGTAGTAGATCCTGTTTTCACTTACCAACAGAGAACGGTGTTAGGACTAT       2 Nora_Median-Nor  96.8%       AGTGCTTTCTTACCGCGCCGCTTTTCTGCTCCAGCAGTAGGAACCCGACTTGTGATAGACCCTGTTTTCACTTACCAACAGAAAACGGTGCTAGGACTAT       3 Nora_raw_reads   96.7%       AGTGCTTTCTTACCGCGCCGCTTTTCTGCTCCAGCAGTAGGAACCCGACTTGTGATAGACCCTGTTTTCACTTACCAACAGAAAACGGTGCTAGGACTAT       4 JX220408.1       96.9%       AGTGCTTTCTTACCGCGCCGCTTTTCTGCTCCAGCAGTAGGAACCCGACTTGTGATAGACCCTGTTTTCACTTACCAACAGAAAACGGTGCTAGGACTAT       5 Nora_MV          96.6%       AGTGCTTTCTTACCGCGCCGCTTTTCTGCTCCAGCAGTAGGAACCCGACTTGTGATAGACCCTGTTTTCACTTACCAACAGAAAACGGTGCTAGGACTAT                                  9501          .         .         .         .         :         .         .         .         .         6 9600  1 NC_007919.3     100.0%       ATAATTATTTCCATGCGGATTTTTATTATATAGTGCACGTTCCAGCACCCTTGGGAACAGGTATCTATCTGAAGATCTACGCTCCTGAATTTGACACTAC       2 Nora_Median-Nor  96.8%       ATAATTATTTCCATGCGGATTTTTATTATATAGTGCATGTTCCAGCACCCTTGGGAACAGGTATCTATCTGAAGATCTATGCTCCTGAATTTGACACTAC       3 Nora_raw_reads   96.7%       ATAATTATTTCCATGCGGATTTTTATTATATAGTGCATGTTCCAGCACCCTTGGGAACAGGTATCTATCTGAAGATCTATGCTCCTGAATTTGACACTAC       4 JX220408.1       96.9%       ATAATTATTTCCATGCGGATTTTTATTATATAGTGCATGTTCCAGCACCCTTGGGAACAGGTATCTATCTGAAGATCTATGCTCCTGAATTTGACACTAC       5 Nora_MV          96.6%       ATAATTATTTCCATGCGGATTTTTATTATATAGTGCATGTTCCAGCACCCTTGGGAACAGGTATCTATCTGAAGATCTATGCTCCTGAATTTGACACTAC                                  9601          .         .         .         .         :         .         .         .         .         7 9700  1 NC_007919.3     100.0%       AACCGTGACACGAGGAATTCGGTTCAAACCAAGTGCATCTCCAACTATTGCACTTTCAGTCCCTTGGAGCAATGATCTGTCGACCGTAGAAACATCCGTA       2 Nora_Median-Nor  96.8%       AACCGTAACACGAGGAATTCGGTTTAAGCCAAGTGCATCTCCAACAATTGCACTTTCAGTCCCTTGGAGCAACGATCTATCGACCGTAGAAACATCCGTA       3 Nora_raw_reads   96.7%       AACCGTAACACGAGGAATTCGGTTTAAGCCAAGTGCATCTCCAACAATTGCACTTTCAGTCCCTTGGAGCAACGATCTATCGACCGTAGAAACATCCGTA       4 JX220408.1       96.9%       AACCGTAACACGAGGAATTCGGTTTAAGCCAAGTGCATCTCCAACAATTGCACTTTCAGTCCCTTGGAGCAACGATCTATCGACCGTAGAAACATCCGTA       5 Nora_MV          96.6%       AACCGTAACACGAGGAATTCGGTTTAAGCCAAGTGCATCTCCAACAATTGCACTTTCAGTCCCTTGGAGCAACGATCTATCGACCGTAGAAACATCCGTA                                  9701          .         .         .         .         :         .         .         .         .         8 9800  1 NC_007919.3     100.0%       GGTCGAGTTGGACAGAGTGGAGGAAGTATTGTTATTGAAACCATCGAAGATAACAGTAACGAGACGGTCAACACCCCACTCAGCATCACCGTTTGGTGTT       2 Nora_Median-Nor  96.8%       GGTCGAGTCGGACAGAGTGGAGGGAGTATTGTTATCGAAACTATCGAAGATAACAGTAACGAGACGGTCAACACCCCACTCAGCATCACCGTTTGGTGTT       3 Nora_raw_reads   96.7%       GGTCGAGTCGGACAGAGTGGAGGGAGTATTGTTATCGAAACTATCGAAGATAACAGTAACGAGACGGTCAACACCCCACTCAGCATCACCGTTTGGTGTT       4 JX220408.1       96.9%       GGTCGAGTCGGACAGAGTGGAGGGAGTATTGTTATCGAAACTATCGAAGATAACAGTAACGAGACGGTCAACACCCCACTCAGCATCACCGTTTGGTGTT       5 Nora_MV          96.6%       GGTCGAGTCGGACAGAGTGGAGGGAGTATTGTTATCGAAACTATCGAAGATAACAGTAACGAGACGGTCAACACCCCACTCAGCATCACCGTTTGGTGTT                                  9801          .         .         .         .         :         .         .         .         .         9 9900  1 NC_007919.3     100.0%       GCATGGCGAACATTAAAGCCACAGGCTATAGGCATGCGGACACGTCAGCTTATAATGAAAAAGGCATGAACTTTATTCCAGTTCCAGTGCCAAAGCCGCC       2 Nora_Median-Nor  96.8%       GCATGGCGAATATTAAAGCCACAGGCTACAAAAATGCGGATACGTCAGCTTACAACGAAAAAGGCATGAACTTTGTCCCAGTTCCAGTGCCAAAGCCGCC       3 Nora_raw_reads   96.7%       GCATGGCGAATATTAAAGCCACAGGCTACAAAAATGCGGATACGTCAGCTTACAACGAAAAAGGCATGAACTTTGTCCCAGTTCCAGTGCCAAAGCCGCC       4 JX220408.1       96.9%       GCATGGCGAACATTAAAGCCACAGGCTACAAAAATGCGGATACGTCAGCTTACAACGAAAAAGGCATGAACTTTGTCCCAGTTCCAGTGCCAAAGCCGCC       5 Nora_MV          96.6%       GCATGGCGAACATTAAAGCCACAGGCTACAAAAATGCGGATACGTCAGCTTACAACGAAAAAGGCATGAACTTTGTCCCAGTTCCAGTGCCAAAGCCGCC                                  9901          .         .         .         .         :         .         .         .         .         0 10000 1 NC_007919.3     100.0%       CGTTCCCCCAACTAAACCAATTACGGGCGAAGAACAAGCTGACAATGAAGTTACAGCCGAAGGTGGTAAACTTGTACAGGAATTGGTCTATGACCATTCT       2 Nora_Median-Nor  96.8%       CGTTCCCCCAACAAAACCAATTATGGGCGAGGAACAAGCTGACAATGAAGTTACAGCCGAAGGTGGTAAACTTGTACAGGAATTGGTTTACGACCATTCT       3 Nora_raw_reads   96.7%       CGTTCCCCCAACAAAACCAATTATGGGCGAGGAACAAGCTGACAATGAAGTTACAGCCGAAGGTGGTAAACTTGTACAGGAATTGGTTTACGACCATTCT       4 JX220408.1       96.9%       CGTTCCCCCAACAAAACCAATTATGGGCGAGGAACAAGCTGACAATGAAGTTACAGCCGAAGGTGGTAAACTTGTACAGGAATTGGTTTACGACCATTCT       5 Nora_MV          96.6%       CGTTCCCCCAACAAAACCAATTATGGGCGAGGAACAAGCTGACAATGAAGTTACAGCCGAAGGTGGTAAACTTGTACAGGAATTGGTTTACGACCATTCT                                 10001          .         .         .         .         :         .         .         .         .         1 10100 1 NC_007919.3     100.0%       GCGATTCCTGTAGCGCCAGTTGTCGAAACACAAGCAGAACAGCCAGAAGTCCCGGTGTCATTAGTGGCAACACGAAAGAACGACACGGGACATCTGGCAA       2 Nora_Median-Nor  96.8%       GCGATTCCTGTAGCGCCAGTCGTCGAAACACAAGCAGAACAGCCGGAAGTCCCAGTTTCATCAGTGGCAACGCGAAAGAACGATACGGGACATTTGGCAA       3 Nora_raw_reads   96.7%       GCGATTCCTGTAGCGCCAGTCGTCGAAACACAAGCAGAACAGCCGGAAGTCCCAGTTTCATCAGTGGCAACGCGAAAGAACGATACGGGACATTTGGCAA       4 JX220408.1       96.9%       GCGATTCCTGTAGCGCCAGTCGTCGAAACACAAGCAGAACAGCCGGAAGTCCCAGTTTCATCAGTGGCAACGCGAAAGAACGATACGGGACATTTGGCAA       5 Nora_MV          96.6%       GCGATTCCTGTAGCGCCAGTCGTCGAAACACAAGCAGAACAGCCGGAAGTCCCAGTTTCATCAGTGGCAACGCGAAAGAACGATACGGGACATTTGGCAA                                 10101          .         .         .         .         :         .         .         .         .         2 10200 1 NC_007919.3     100.0%       CAAAGTGGTATGATTTTGCCAAAATCAGTCTGTCAAATCCAGCCAACATGAATTGGACCACACTAACCATAGACCCGTACAACAATGTTACATTGTCTAG       2 Nora_Median-Nor  96.8%       CAAAGTGGTATGATTTCGCCAAAATCAGTCTGTCAAACCCAGCTAACATGAACTGGACCACGCTAACCATAGACCCGTACAACAATGTTACATTGTCTAG       3 Nora_raw_reads   96.7%       CAAAGTGGTATGATTTCGCCAAAATCAGTCTGTCAAACCCAGCTAACATGAACTGGACCACGCTAACCATAGACCCGTACAACAATGTTACATTGTCTAG       4 JX220408.1       96.9%       CAAAGTGGTATGATTTCGCCAAAATCAGTCTGTCAAACCCAGCTAACATGAACTGGACCACGCTAACCATAGACCCGTACAACAATGTTACATTGTCTAG       5 Nora_MV          96.6%       CAAAGTGGTATGATTTCGCCAAAATCAGTCTGTCAAACCCAGCTAACATGAACTGGACCACGCTAACCATAGACCCGTACAACAATGTTACATTGTCTAG                                 10201          .         .         .         .         :         .         .         .         .         3 10300 1 NC_007919.3     100.0%       GGATGGTGAGTCGATGGTTCTACCATGGAGGCGAAATGTTTGGACAACCGGATCGAAAAGTATTGGATATATCCGAACAATGGTTGCACAGATTAACATA       2 Nora_Median-Nor  96.8%       AGATGGTGAGTCGATGGTCCTACCATGGAGGCGAAATGTTTGGACAACCGGATCGAAAAGTATTGGATATATCCGAACGATGGTTGCACAAATTAACATA       3 Nora_raw_reads   96.7%       AGATGGTGAGTCGATGGTCCTACCATGGAGGCGAAATGTTTGGACAACCGGATCGAAAAGTATTGGATATATCCGAACGATGGTTGCACAAATTAACATA       4 JX220408.1       96.9%       AGATGGTGAGTCGATGGTCCTACCATGGAGGCGAAATGTTTGGACAACCGGATCGAAAAGTATTGGATATATCCGAACGATGGTTGCACAAATTAACATA       5 Nora_MV          96.6%       AGATGGTGAGTCGATGGTCCTACCATGGAGGCGAAATGTTTGGACAACCGGATCGAAAAGTATTGGATATATCCGAACGATGGTTGCACAAATTAACATA                                 10301          .         .         .         .         :         .         .         .         .         4 10400 1 NC_007919.3     100.0%       CCACGTCCGCCGCAAATCAGTGGAGTGCTCGAAGTTAAAGATTCAATCAATAACTCAAGTATTTCACTGGTAGAGTTTGGAGGAAAAGTAGAAATCCCAA       2 Nora_Median-Nor  96.8%       CCACGCCCGCCGCAGATCAGTGGAGTGCTCGAAGTTAAAGATTCAATCAATAACTCAAGTATTTCACTGGTAGAATTTGGAGGAAAAGTAGAGATTCCAA       3 Nora_raw_reads   96.7%       CCACGCCCGCCGCAGATCAGTGGAGTGCTCGAAGTTAAAGATTCAATCAATAACTCAAGTATTTCACTGGTAGAATTTGGAGGAAAAGTAGAGATTCCAA       4 JX220408.1       96.9%       CCACGCCCGCCGCAGATCAGTGGAGTGCTCGAAGTTAAAGATTCAATCAATAACTCAAGTATTTCACTGGTAGAATTTGGAGGAAAAGTAGAGATTCCAA       5 Nora_MV          96.6%       CCACGCCCGCCGCAGATCAGTGGAGTGCTCGAAGTTAAAGATTCAATCAATAACTCAAGTATTTCACTGGTAGAATTTGGAGGAAAAGTAGAGATTCCAA                                 10401          .         .         .         .         :         .         .         .         .         5 10500 1 NC_007919.3     100.0%       TTATTCCAAAGGTTATGAACGGACTAGCAACAACTGCTAGTTTGCCAAGGCATAGACTAAACCCGTGGATGAGAACCGCCGAAAGTAAGGTTGAATTGCA       2 Nora_Median-Nor  96.8%       TTATTCCGAAGGTTATGAACGGACTAGTAACAAGTGCTAGTTTGCCAAGGCATAGACTAAACCCATGGATGAGAACCGCCGAAAGTAAGGTTGAATTGCA       3 Nora_raw_reads   96.7%       TTATTCCGAAGGTTATGAACGGACTAGTAACAAGTGCTAGTTTGCCAAGGCATAGACTAAACCCATGGATGAGAACCGCCGAAAGTAAGGTTGAATTGCA       4 JX220408.1       96.9%       TTATTCCGAAGGTTATGAACGGACTAGTAACAAGTGCTAGTTTGCCAAGGCATAGACTAAACCCATGGATGAGAACCGCCGAAAGTAAGGTTGAATTGCA       5 Nora_MV          96.6%       TTATTCCGAAGGTTATGAACGGACTAGTAACAAGTGCTAGTTTGCCAAGGCATAGACTAAACCCATGGATGAGAACCGCCGAAAGTAAGGTTGAATTGCA                                 10501          .         .         .         .         :         .         .         .         .         6 10600 1 NC_007919.3     100.0%       ATATCGCATTATTGCTTTTAATCGAACTAGCGACATTGCTGACCTTAACGTTAGCGTTTTGTTGCGACCTGGCGATTCGCAATTCCAATTGCCGATGAAA       2 Nora_Median-Nor  96.8%       ATATCGAATTATTGCTTTTAATCGAACTAGCGACATTGCTGACCTTAACGTTAGCGTTTTGTTGCGACCTGGCGATTCGCAATTCCAATTGCCGATGAAA       3 Nora_raw_reads   96.7%       ATATCGAATTATTGCTTTTAATCGAACTAGCGACATTGCTGACCTTAACGTTAGCGTTTTGTTGCGACCTGGCGATTCGCAATTCCAATTGCCGATGAAA       4 JX220408.1       96.9%       ATATCGAATTATTGCTTTTAATCGAACTAGCGACATTGCTGACCTTAACGTTAGCGTTTTGTTGCGACCTGGCGATTCGCAATTCCAATTGCCGATGAAA       5 Nora_MV          96.6%       ATATCGAATTATTGCTTTTAATCGAACTAGCGACATTGCTGACCTTAACGTTAGCGTTTTGTTGCGACCTGGCGATTCGCAATTCCAATTGCCGATGAAA                                 10601          .         .         .         .         :         .         .         .         .         7 10700 1 NC_007919.3     100.0%       CCTGACAATAATGTGGATACACGTCATTTTGAGCTCGTTGAAGCTTTGATGTATCACTACGATAGCCTCCGAATTCGAGGAGAAGAACAGAGTCTACCAG       2 Nora_Median-Nor  96.8%       CCTGACAATAGTGTGGATACACGTCATTTTGAGCTTGTTGAAGCTTTAATGTACCACTACGATAGCCTCCGAATTCGAGGAGAAGAACAGAGTCTGCCAG       3 Nora_raw_reads   96.7%       CCTGACAATAGTGTGGATACACGTCATTTTGAGCTTGTTGAAGCTTTAATGTACCACTACGATAGCCTCCGAATTCGAGGAGAAGAACAGAGTCTGCCAG       4 JX220408.1       96.9%       CCTGACAATAGTGTGGATACACGTCATTTTGAGCTTGTTGAAGCTTTAATGTACCACTACGATAGCCTCCGAATTCGAGGAGAAGAACAGAGTCTGCCAG       5 Nora_MV          96.6%       CCTGACAATAGTGTGGATACACGTCATTTTGAGCTTGTTGAAGCTTTAATGTACCACTACGATAGCCTCCGAATTCGAGGAGAAGAACAGAGTCTGCCAG                                 10701          .         .         .         .         :         .         .         .         .         8 10800 1 NC_007919.3     100.0%       AAAATGCACCTAATGCAGTTTCAAACCCTCAGCAGTTTATCACACCCGCAACCGCTCTAAGTGCCGAAGAATATAATGTGCACGAGGCGTTGGGTGAGAC       2 Nora_Median-Nor  96.8%       AAAATGCACCTAATGCAGTTTCAAACCCTCAGCAGTTCATTACACCCGCAACCGCTCTAAGTGCCGAAGAATATAATGTGCACGAGGCGTTGGGTGAAAC       3 Nora_raw_reads   96.7%       AAAATGCACCTAATGCAGTTTCAAACCCTCAGCAGTTCATTACACCCGCAACCGCTCTAAGTGCCGAAGAATATAATGTGCACGAGGCGTTGGGTGAAAC       4 JX220408.1       96.9%       AAAATGCACCTAATGCAGTTTCAAACCCTCAGCAGTTCATTACACCCGCAACCGCTCTAAGTGCCGAAGAATATAATGTGCACGAGGCGTTGGGTGAAAC       5 Nora_MV          96.6%       AAAATGTACCTAATGCAGTTTCAAACCCTCAGCAGTTCATTACACCCGCAACCGCTCTAAGTGCCGAAGAATATAATGTGCACGAGGCGTTGGGTGAAAC                                 10801          .         .         .         .         :         .         .         .         .         9 10900 1 NC_007919.3     100.0%       TGAGGAGTTGGAGCTGGATGAATTTCCAGTTCTGGTGTTCAAGGGAAATGTTCCCGTTGACTCAGTGACGTCCATTCCTTTGGACCTCGCAACTATATAC       2 Nora_Median-Nor  96.8%       TGAGGAGTTGGAGCTGGATGAATTTCCGGTTCTGGTGTTCAAGGGAAATGTTCCCGTTGACTCAGTGACGTCCATTCCTTTGGACCTCGCAACTATATAC       3 Nora_raw_reads   96.7%       TGAGGAGTTGGAGCTGGATGAATTTCCGGTTCTGGTGTTCAAGGGAAATGTTCCCGTTGACTCAGTGACGTCCATTCCTTTGGACCTCGCAACTATATAC       4 JX220408.1       96.9%       TGAGGAGTTGGAGCTGGATGAATTTCCGGTTCTGGTGTTCAAGGGAAATGTTCCCGTTGACTCAGTGACGTCCATTCCTTTGGACCTCGCAACTATATAC       5 Nora_MV          96.6%       TGAGGAGTTGGAGCTGGATGAATTTCCGGTTCTGGTGTTCAAGGGAAATGTTCCCGTTGACTCAGTGACGTCCATTCCTTTGGACCTCGCAACTATATAC                                 10901          .         .         .         .         :         .         .         .         .         0 11000 1 NC_007919.3     100.0%       GATTTCGCTTGGGACGGAGAGCAGAATGCAATTTCTCAGAAATTTCAGCGTTTTGCTCATCTGATACCGAAAAGCGCAGGTGGTTTTGGCCCAGTGATTG       2 Nora_Median-Nor  96.8%       GACTTTGCTTGGGACGGAGAGCAGAATGCAATTTCTCAGAAATTTCAGCGTTTTGCTCATCTGATACCGAAAAGCGCAGGTGGTTTTGGCCCAGTGATTG       3 Nora_raw_reads   96.7%       GACTTTGCTTGGGACGGAGAGCAGAATGCAATTTCTCAGAAATTTCAGCGTTTTGCTCATCTGATACCGAAAAGCGCAGGTGGTTTTGGCCCAGTGATTG       4 JX220408.1       96.9%       GACTTTGCTTGGGACGGAGAGCAGAATGCAATTTCTCAGAAATTTCAGCGTTTTGCTCATCTGATACCGAAAAGCGCAGGTGGTTTTGGCCCAGTGATTG       5 Nora_MV          96.6%       GACTTTGCTTGGGACGGAGAGCAGAATGCAATTTCTCAGAAATTTCAGCGTTTTGCTCATCTGATACCGAAAAGCGCAGGTGGTTTTGGCCCAGTGATTG                                 11001          .         .         .         .         :         .         .         .         .         1 11100 1 NC_007919.3     100.0%       GTAATTATACTATCACGGCTAACCTCCCTACCGGTGTAGCAGGTCGTATTCTGCACAATTGTCTCCCAGGAGATTGTGTAGATCTAGCAGTGTCGAGAAT       2 Nora_Median-Nor  96.8%       GTAATTATACTATCACGGCTAACCTCCCCACCGGTGTAGCAGGTCGTATTCTGCACAATTGTCTCCCAGGAGATTGTGTAGATCTAGCAGTATCGAGAAT       3 Nora_raw_reads   96.7%       GTAATTATACTATCACGGCTAACCTCCCCACCGGTGTAGCAGGTCGTATTCTGCACAATTGTCTCCCAGGAGATTGTGTAGATCTAGCAGTATCGAGAAT       4 JX220408.1       96.9%       GTAATTATACTATCACGGCTAACCTCCCCACCGGTGTAGCAGGTCGTATTCTGCACAATTGTCTCCCAGGAGATTGTGTAGATCTAGCAGTATCGAGAAT       5 Nora_MV          96.6%       GTAATTATACTATCACGGCTAACCTCCCCACCGGTGTAGCAGGTCGTATTCTGCACAATTGTCTCCCAGGAGATTGTGTAGATCTAGCAGTATCGAGAAT                                 11101          .         .         .         .         :         .         .         .         .         2 11200 1 NC_007919.3     100.0%       TTTTGGCTTGAAGAGCCTTCTTGGTGTTGCAGGAACAGCAGTTTCAGCCATTGGTGGTCCACTCCTTAACGGTTTGGTTAACACCGCAGCGCCTATCCTT       2 Nora_Median-Nor  96.8%       TTTTGGCTTGAAGAGCCTTCTCGGTGTTGCAGGAACAGCAGTTTCAGCCATTGGTGGCCCACTCCTTAACGGTTTGGTTAACACCGCAGCGCCTATCCTT       3 Nora_raw_reads   96.7%       TTTTGGCTTGAAGAGCCTTCTCGGTGTTGCAGGAACAGCAGTTTCAGCCATTGGTGGCCCACTCCTTAACGGTTTGGTTAACACCGCAGCGCCTATCCTT       4 JX220408.1       96.9%       TTTTGGCTTGAAGAGCCTTCTCGGTGTTGCAGGAACAGCAGTTTCAGCCATTGGTGGCCCACTCCTTAACGGTTTGGTTAACACCGCAGCGCCTATCCTT       5 Nora_MV          96.6%       TTTTGGCTTGAAGAGCCTTCTCGGTGTTGCAGGAACAGCAGTTTCAGCCATTGGTGGCCCACTCCTTAACGGTTTGGTTAACACCGCAGCGCCTATCCTT                                 11201          .         .         .         .         :         .         .         .         .         3 11300 1 NC_007919.3     100.0%       AGTGGAGCAGCACACGCCATTGGTGGAAATGTTGTAGGAGGACTGGCTGATGCAGTGATCGACATTGGATCTAATTTGCTGACGCCAAAAGAAAAAGAAC       2 Nora_Median-Nor  96.8%       AGTGGAGCAGCGCACGCCATTGGTGGAAATGTTGTAGGAGGACTGGCTGATGCAGTGATCGACATTGGATCTAATTTGCTGACGCCAAAAGAAAAAGAAC       3 Nora_raw_reads   96.7%       AGTGGAGCAGCGCACGCCATTGGTGGAAATGTTGTAGGAGGACTGGCTGATGCAGTGATCGACATTGGATCTAATTTGCTGACGCCAAAAGAAAAAGAAC       4 JX220408.1       96.9%       AGTGGAGCAGCGCACGCCATTGGTGGAAATGTTGTAGGAGGACTGGCTGATGCAGTGATCGACATTGGATCTAATTTGCTGACGCCAAAAGAAAAAGAAC       5 Nora_MV          96.6%       AGTGGAGCAGCGCACGCCATTGGTGGAAATGTTGTAGGAGGACTGGCTGATGCAGTGATCGACATTGGATCTAATTTGCTGACGCCAAAAGAAAAAGAAC                                 11301          .         .         .         .         :         .         .         .         .         4 11400 1 NC_007919.3     100.0%       AACCAAGCGCAAATTCAAGCGCGATTTCTGGAGATATTCCGATTTCGCGCTTTGTAGAGATGCTCAAGTATGTCAAGGAGAATTACCAGGATAATCCTGT       2 Nora_Median-Nor  96.8%       AACCAAGCGCAAATTCAAGCGCGATTTCTGGAGATATTCCGATTTCGCGTTTTGTAGAGATGCTCAAGTATGTCAAGGAGAATTACCAGGATAATCCTGT       3 Nora_raw_reads   96.7%       AACCAAGCGCAAATTCAAGCGCGATTTCTGGAGATATTCCGATTTCGCGTTTTGTAGAGATGCTCAAGTATGTCAAGGAGAATTACCAGGATAATCCTGT       4 JX220408.1       96.9%       AACCAAGCGCAAATTCAAGCGCGATTTCTGGAGATATTCCGATTTCGCGTTTTGTAGAGATGCTCAAGTATGTCAAGGAGAATTACCAGGATAATCCTGT       5 Nora_MV          96.6%       AACCAAGCGCAAATTCAAGCGCGATTTCTGGAGATATTCCGATTTCGCGTTTTGTAGAGATGCTCAAGTATGTCAAGGAGAATTACCAGGATAATCCTGT                                 11401          .         .         .         .         :         .         .         .         .         5 11500 1 NC_007919.3     100.0%       ATTCCCGACTTTGCTTGTTGAACCACAAAACTTCATCTCAAACGCAATGACCGCACTCAAAACAATTCCAATTGAGGTCTTTGCCAATATGCGCAATGTG       2 Nora_Median-Nor  96.8%       GTTCCCGACTTTACTTGTTGAACCACAAAACTTCATCTCAAACGCAATGACCGCACTCAAAACAATTCCAATTGAGGTCTTTGCCAACATGCGCAATGTG       3 Nora_raw_reads   96.7%       GTTCCCGACTTTACTTGTTGAACCACAAAACTTCATCTCAAACGCAATGACCGCACTCAAAACAATTCCAATTGAGGTCTTTGCCAACATGCGCAATGTG       4 JX220408.1       96.9%       GTTCCCGACTTTACTTGTTGAACCACAAAACTTCATCTCAAACGCAATGACCGCACTCAAAACAATTCCAATTGAGGTCTTTGCCAACATGCGCAATGTG       5 Nora_MV          96.6%       GTTCCCGACTTTACTTGTTGAACCACAAAACTTCATCTCAAACGCAATGACCGCACTCAAAACAATTCCAATTGAGGTCTTTGCCAACATGCGCAATGTG                                 11501          .         .         .         .         :         .         .         .         .         6 11600 1 NC_007919.3     100.0%       AAAGTAGAACGAAATTTGTTCGACCGAACAGTTGTTCCAACTGTGAAGGAAGCTACTCTAGCTGATATTGTCATACCTAATCATATGTATGGTTATATTC       2 Nora_Median-Nor  96.8%       AAAGTTGAACGAAATTTGTTCGACCGAACGGTTGTTCCAACTGTGAAGGAAGCTACTCTAGCTGATATTGTCATACCTAATCATATATATGGCTATATTC       3 Nora_raw_reads   96.7%       AAAGTTGAACGAAATTTGTTCGACCGAACGGTTGTTCCAACTGTGAAGGAAGCTACTCTAGCTGATATTGTCATACCTAATCATATGTATGGCTATATTC       4 JX220408.1       96.9%       AAAGTTGAACGAAATTTGTTCGACCGAACGGTTGTTCCAACTGTGAAGGAAGCTACTCTAGCTGATATTGTCATACCTAATCATATGTATGGCTATATTC       5 Nora_MV          96.6%       AAAGTTGAACGAAATTTGTTCGACCGAACGGTTGTTCCAACTGTGAAGGAAGCTACTCTAGCTGATATTGTCATACCTAATCATATGTATGGCTATATTC                                 11601          .         .         .         .         :         .         .         .         .         7 11700 1 NC_007919.3     100.0%       TTCGAGACTTTCTTCAAAACAAACGAGCGTTTCAATCTGGGACCAAACAAAATGTGTATTTTCAACAGTTTCTAACTGTTCTATCTCAACGCAATATCCG       2 Nora_Median-Nor  96.8%       TCCGAGACTTCCTCCAAAACAAACGAGCGTTTCAATCTGGGACCAAACAAAATGTGTACTTTCAGCAGTTTCTAACTGTTCTATCTCAACGTAATACCCG       3 Nora_raw_reads   96.7%       TCCGAGACTTCCTCCAAAACAAACGAGCGTTTCAATCTGGGACCAAACAAAATGTGTACTTTCAGCAGTTTCTAACTGTTCTATCTCAACGTAATACCCG       4 JX220408.1       96.9%       TCCGAGACTTCCTCCAAAACAAACGAGCGTTTCAATCTGGGACCAAACAAAATGTGTACTTTCAGCAGTTTCTAACTGTTCTATCTCAACGTAATACCCG       5 Nora_MV          96.6%       TCCGAGACTTCCTCCAAAACAAACGAGCGTTTCAATCTGGGACCAAACAAAATGTGTACTTTCAGCAGTTTCTAACTGTTCTATCTCAACGTAATACCCG                                 11701          .         .         .         .         :         .         .         .         .         8 11800 1 NC_007919.3     100.0%       TACACATATTACGCTCAACGACATCACAAGTTGCTCTATTGATAGTGAGTCGATTGCAAACAAAATAGAGAGAGTAAAACACTACTTGAGTACAAACTCG       2 Nora_Median-Nor  96.8%       TACACATATTACGCTCAACGACATCACAAGTTGCTCTATTGATAGTGAGTCGATTGCAAACAAAATAGAGAGAGTAAAACGCTACTTGAGTGCAAACTCG       3 Nora_raw_reads   96.7%       TACACATATTACGCTCAACGACATCACAAGTTGCTCTATTGATAGTGAGTCGATTGCAAACAAAATAGAGAGAGTAAAACGCTACTTGAGTGCAAACTCG       4 JX220408.1       96.9%       TACACATATTACGCTCAACGACATCACAAGTTGCTCTATTGATAGTGAGTCGATTGCAAACAAAATAGAGAGAGTAAAACGCTACTTGAGTGCAAACTCG       5 Nora_MV          96.6%       TACACATATTACGCTCAACGACATCACAAGTTGCTCTATTGATAGTGAGTCGATTGCAAACAAAATAGAGAGAGTAAAACGCTACTTGAGTGCAAACTCG                                 11801          .         .         .         .         :         .         .         .         .         9 11900 1 NC_007919.3     100.0%       A--GTGGGGAGACCACGGAAGAATTTTCGCGAACAGACACCGGTCTGCTACCTATTACGACCCGCAAAATCGTATTAGGTGAAAGTAAACGCAGGACGGA       2 Nora_Median-Nor  96.8%       A--GTGGGGAGACCACGGAAGAATTTTCGCGAACAGACACCGGTCTGCTACCTACTACGACCCGCAAAATCGTATTAGGTGAAAGTAAACGCAGGACGGA       3 Nora_raw_reads   96.7%       AGTGTGGGGAGACCACGGAAGAATTTTCGCAAAGAAACAGCG--CTGCTACCTACTACGACCCGCAAAATCGTATTAGGTGAAAGTAAACGCAGGACGGA       4 JX220408.1       96.9%       A--GTGGGGAGACCACGGAAGAATTTTCGCGAACAGACACCGGTCTGCTACCTACTACGACCCGCAAAATCGTATTAGGTGAAAGTAAACGCAGGACGGA       5 Nora_MV          96.6%       A--GTGGGGAGACCACGGAAGAATTTTCGCGAACAGACACCGGTCTGCTACCTACTACGACCCGCAAAATCGTATTAGGTGAAAGTAAACGCAGGACGGA                                 11901          .         .         .         .         :         .         .         .         .         0 12000 1 NC_007919.3     100.0%       ACGATACGTAGCAGAAACTGTTTTCCCTTCCGTGAGGCAGTAGATTAAATCCAAAACTCGCCATGTGCGCGTCTCAGAGACTGATTTTTAAAATTGGTTT       2 Nora_Median-Nor  96.8%       ACGAAACGTAGCAGAAACTGTTTTCCCTTCCGTGAGGCAGTAGATTAAATCCAAAACTCGCCATGTGCGCGTCTCAGAAACTAATTTTTAAAATTGGTTT       3 Nora_raw_reads   96.7%       ACGAAACGTAGCAGAAACTGTTTTCCCTTCCGTGAGGCAGTAGATTAAATCCAAAACTCGCCATGTGCGCGTCTCAGAAACTAATTTTTAAAATTGGTTT       4 JX220408.1       96.9%       ACGAAACGTAGCAGAAACTGTTTTCCCTTCCGTGAGGCAGTAGATTAAATCCAAAACTCGCCATGTGCGCGTCTCAGAAACTAATTTTTAAAATTGGTTT       5 Nora_MV          96.6%       ACGAAACGTAGCAGAAACTGTTTTCCCTTCCGTGAGGCAGTAGATTAAATCCAAAACTCGCCATGTGCGCGTCTCAGAAACTAATTTTTAAAATTGGTTT                                 12001          .         .         .         .         :         .         .         .         .         1 12100 1 NC_007919.3     100.0%       GCGCGTGTGGTTAAAAAAAATCGCATTAAATATGAGGATGGTCGCTATAATTTTCTAGCAGAAAGTTATAAAGGACGGTGTGATGATGTTTTGGAGCCCG       2 Nora_Median-Nor  96.8%       GCGCGTATGGTTAAAA--AGTCACATTAAATATGAGGATGGTCGCTATAATTTTCTAGCAGAAGATTATAAAGGACGGTGTGATGATGTTTTGGAGCCCG       3 Nora_raw_reads   96.7%       GCGCGTATGGTTAAAA--AGTCACATTAAATATGAGGATGGTCGCTATAATTTTCTAGCAGAAGATTATAAAGGACGGTGTGATGATGTTTTGGAGCCCG       4 JX220408.1       96.9%       GCGCGTATGGTTAAAAAGAGTCACATTAAATATGAGGATGGTCGCTATAATTTTCTAGCAGAAGATTATAAAGGACGGTGTGATGATGTTTTGGAGCCCG       5 Nora_MV          96.6%       GCGCGTATGGTTAAAA--AGTCACATTAAATATGAGGATGGTCGCTATAATTTTCTAGCAGAAGATTATAAAGGACGGTGTGATGATGTTTTGGAGCCCG                                 12101          .         .         .         .         :         .         .         .         .         2 12200 1 NC_007919.3     100.0%       CACTGGGTGTGGGTTCGTAGTGCTAGCAATTGCAACTTTTCCTGAAAGTTAATACCTATAGTCTATAGGAGAAAAATTAACATTTATTGAGAATATCCGT       2 Nora_Median-Nor  96.8%       CACTGGGTGTGGGTTCGTAGTGCTAGCAATTGCAACTTTTCCTGAAAGTTAATACCTATAGTTTATAGGAGAAAAATTAACATTTATTGAGAATATCTGT       3 Nora_raw_reads   96.7%       CACTGGGTGTGGGTTCGTAGTGCTAGCAATTGCAACTTTTCCTGAAAGTTAATACCTATAGTTTATAGGAGAAAAATTAACATTTATTGAGAATATCTGT       4 JX220408.1       96.9%       CACTGGGTGTGGGTTCGTAGTGCTAGCAATTGCAACTTTTCCTGAAAGTTAATACCTATAGTTTATAGGAGAAAAATTAACATTTATTGAGAATATCTGT       5 Nora_MV          96.6%       CACTGGGTGTGGGTTCGTAGTGCTAGCAATTGCAACTTTTCCTGAAAGTTAATACCTATAGTTTATAGGAGAAAAATTAACATTTATTGAGAATATCTGT                                 12201          .         .         .         .         :         .         .         .         .         3 12300 1 NC_007919.3     100.0%       GCAAACGACTCCCCACGCGGGGAGTAGTGGAATCTCGATAAACAGGTTTTTTAAGCTTTTGCGTTTCCGAAAGGGTTACTCTGGCTGAACCAGCAGTTTT       2 Nora_Median-Nor  96.8%       GCAAACGACTCCCCACGCGGGGAGTAGTGGAATCTTGATAAACAGGTTTTTTAAGCTTTTGCGTTTCCGAAAGGGTTACTCTGGCTGAACCAGCAGTTTT       3 Nora_raw_reads   96.7%       GCAAACGACTCCCCACGCGGGGAGTAGTGGAATCTTGATAAACAGGTTTTTTAAGCTTTTGCGTTTCCGAAAGGGTTACTCTGGCTGAACCAGCAGTTTT       4 JX220408.1       96.9%       GCAAACGACTCCCCACGCGGGGAGTAGTGGAATCTTGATAAACAGGTTTTTTAAGCTTTTGCGTTTCCGAAAGGGTTACTCTGGCTGAACCAGCAGTTTT       5 Nora_MV          96.6%       GCAAACGACTCCCCACGCGGGGAGTAGTGGAATCTTGATAAACAGGTTTTTTAAGCTTTTGCGTTTCCGAAAGGGTTACTCTGGCTGAACCAGCAGTTTT                                 12301          .         .         .      ] 12337 1 NC_007919.3     100.0%       TATAACTGTGGGTAGCCCCTCGGAAAATCAGGTTTGC       2 Nora_Median-Nor  96.8%       TATAACTGTGGGTAGCCCCTCGGAAAATCAGGTTTGC       3 Nora_raw_reads   96.7%       TATAACTGTGGGTAGCCCCTCGGAAAATCAGGTTTGC       4 JX220408.1       96.9%       TATAACTGTGGGTAGCCCCTCGGAAAATCAGGTTTGC       5 Nora_MV          96.6%       TATAACTGTGGGTAGCCCCTCGGAAAATCAGGTTTGC ``` |
