# Supplementary material for: Metavisitor, a Suite of Galaxy Tools for Simple and Rapid Detection and Discovery of Viruses in Deep Sequence Data
Source: PLoS One. 2017 Jan 3;12(1):e0168397. doi: 10.1371/journal.pone.0168397 (PMC5207757; doi:10.1371/journal.pone.0168397)
Supplement: S10 File — Small RNA sequences reads were aligned to the contigs and size distribution and read maps were generated using the “Generate readmap and histograms from alignment files” tool. Plots show the map and abundance of 18–30 nt small RNA reads for indicated contigs and histograms show length distributions of these reads. Positive and negative values correspond to sense and antisense reads, respectively. (PDF) [file pone.0168397.s027.pdf]

Readmaps and size distributions

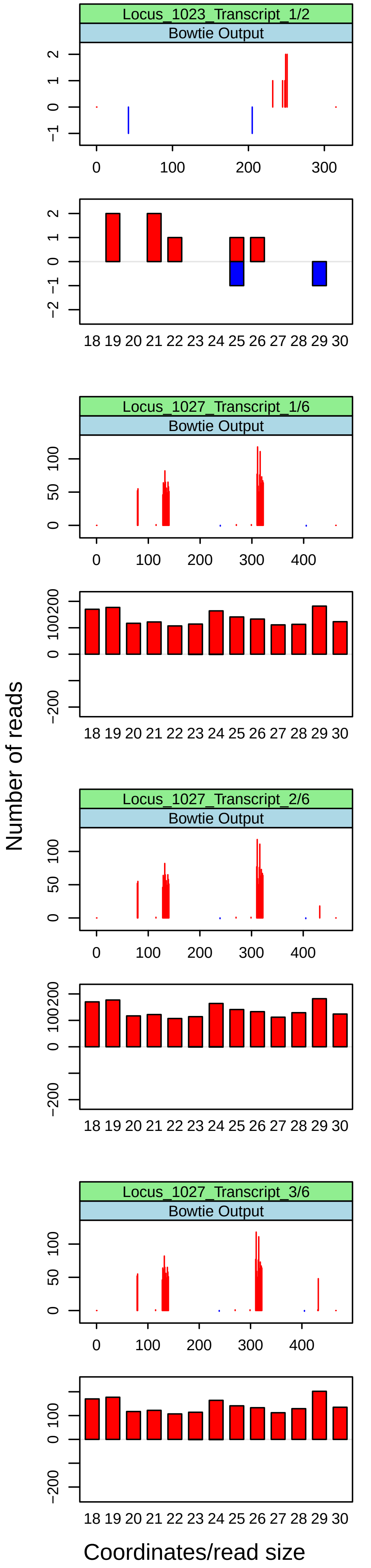

Readmaps and size distributions

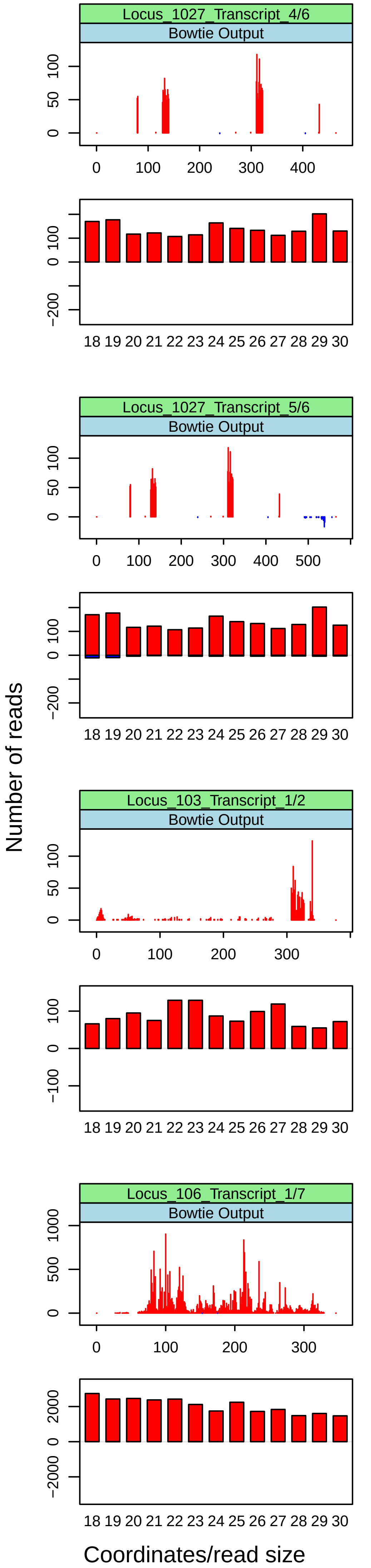

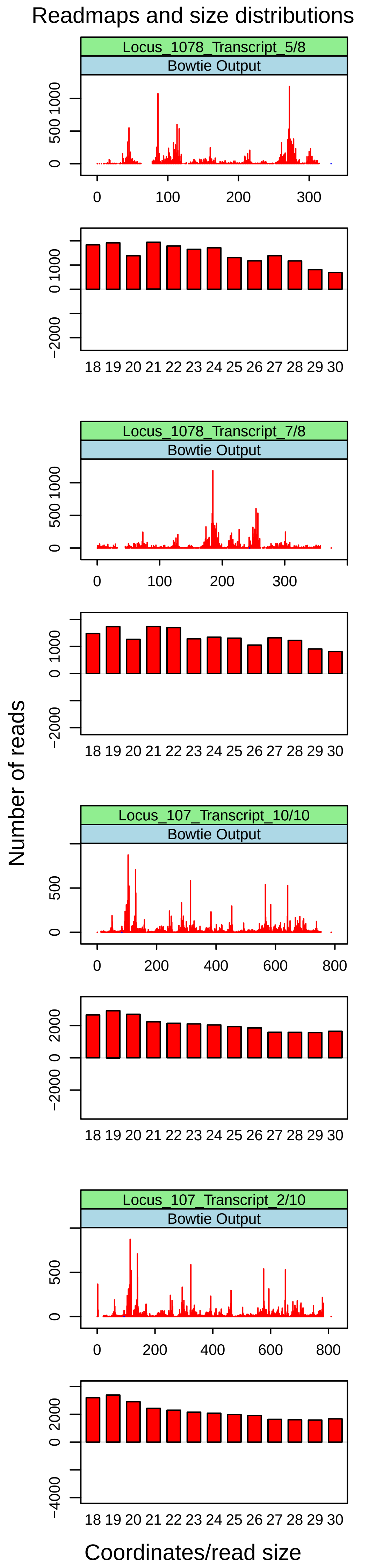
Readmaps and size distributions
Locus_1078_Transcript_5/8
Bowtie Output
Locus_1078_Transcript_7/8
Bowtie Output
Locus_107_Transcript_10/10
Bowtie Output
Locus_107_Transcript_2/10
Bowtie Output
Number of reads
Coordinates/read size

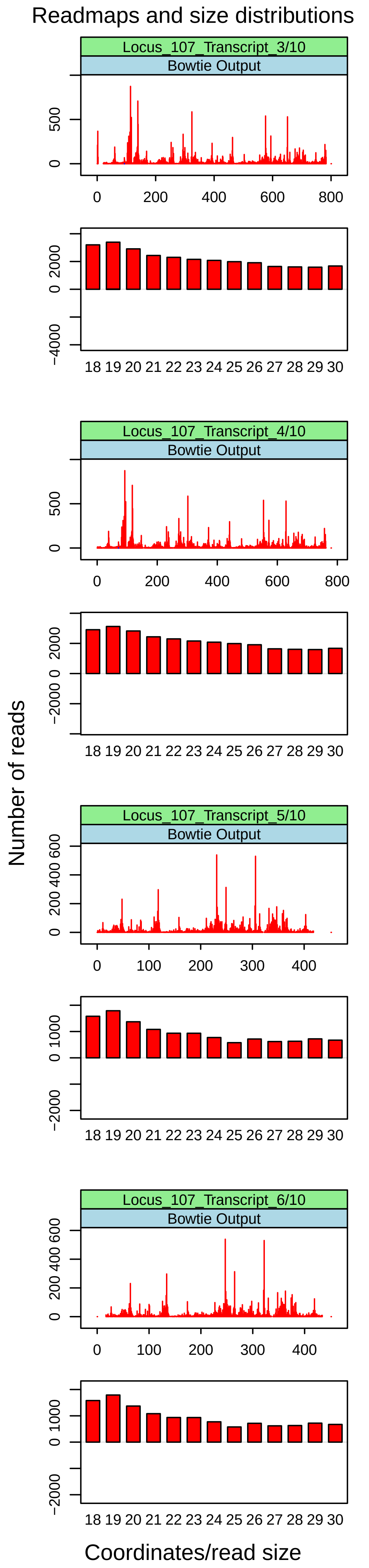
Readmaps and size distributions
Locus_107_Transcript_3/10
Bowtie Output
0
500
0
200
400
600
800
−4000
0
2000
18 19 20 21 22 23 24 25 26 27 28 29 30
Locus_107_Transcript_4/10
Bowtie Output
0
500
0
200
400
600
800
−2000 0
2000
18 19 20 21 22 23 24 25 26 27 28 29 30
Locus_107_Transcript_5/10
Bowtie Output
0
200 400 600
0
100
200
300
400
−2000
0
1000
18 19 20 21 22 23 24 25 26 27 28 29 30
Locus_107_Transcript_6/10
Bowtie Output
0
200 400 600
0
100
200
300
400
−2000
0
1000
18 19 20 21 22 23 24 25 26 27 28 29 30
Number of reads
Coordinates/read size

Readmaps and size distributions

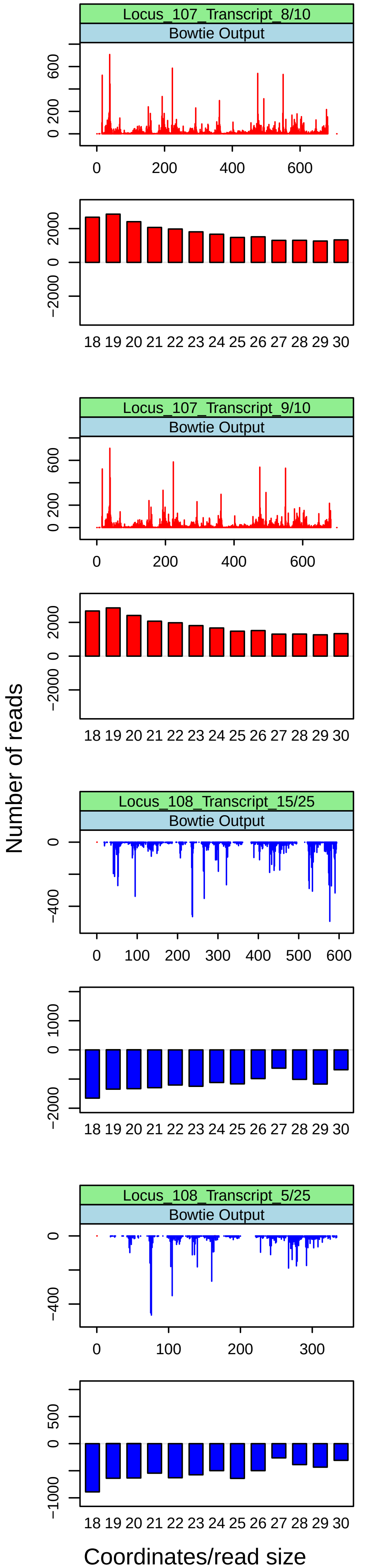

Readmaps and size distributions

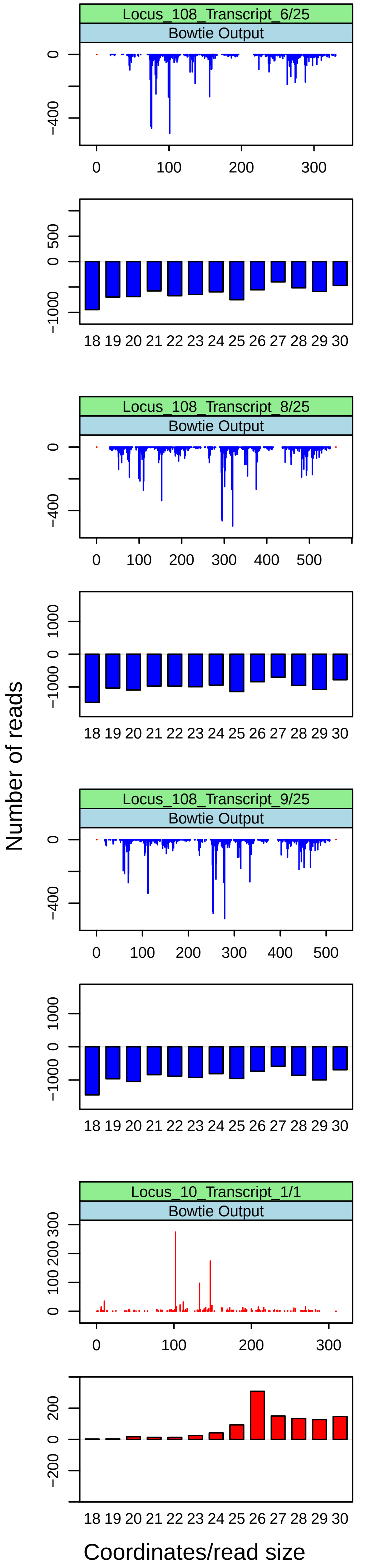

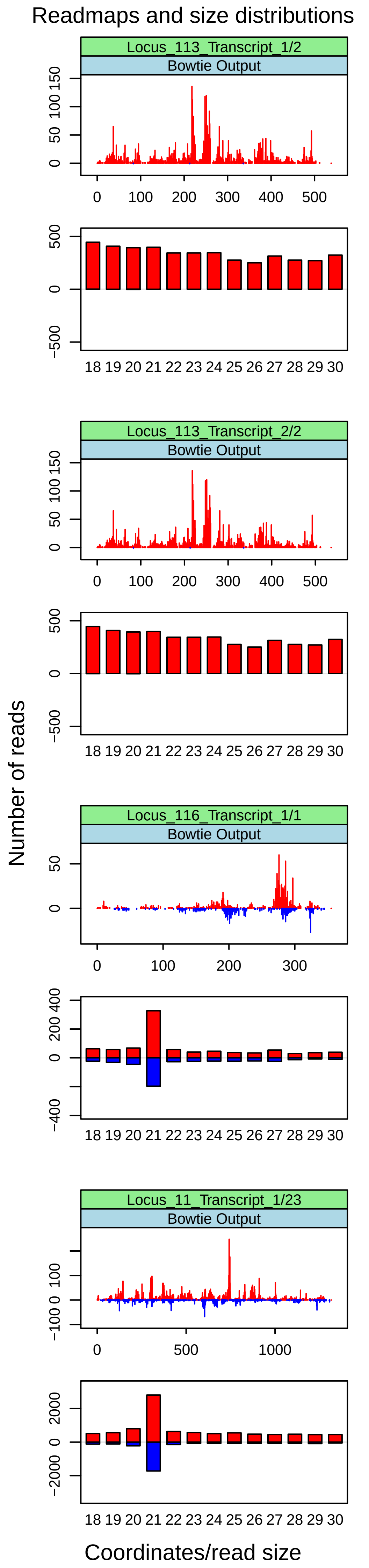
Readmaps and size distributions
Locus_113_Transcript_1/2
Bowtie Output
Locus_113_Transcript_2/2
Bowtie Output
Locus_116_Transcript_1/1
Bowtie Output
Locus_11_Transcript_1/23
Bowtie Output
18 19 20 21 22 23 24 25 26 27 28 29 30
Number of reads
Coordinates/read size

Readmaps and size distributions

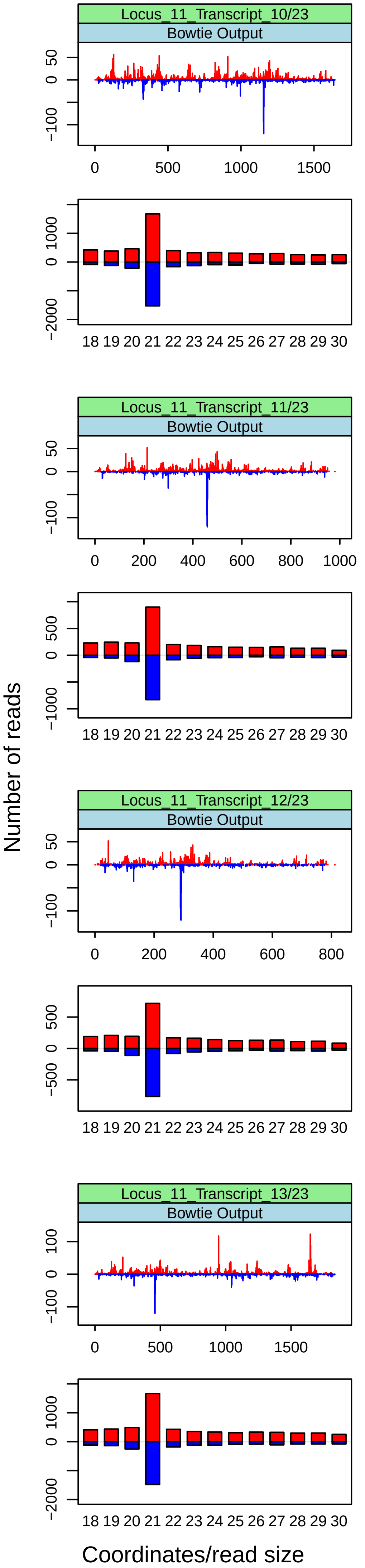

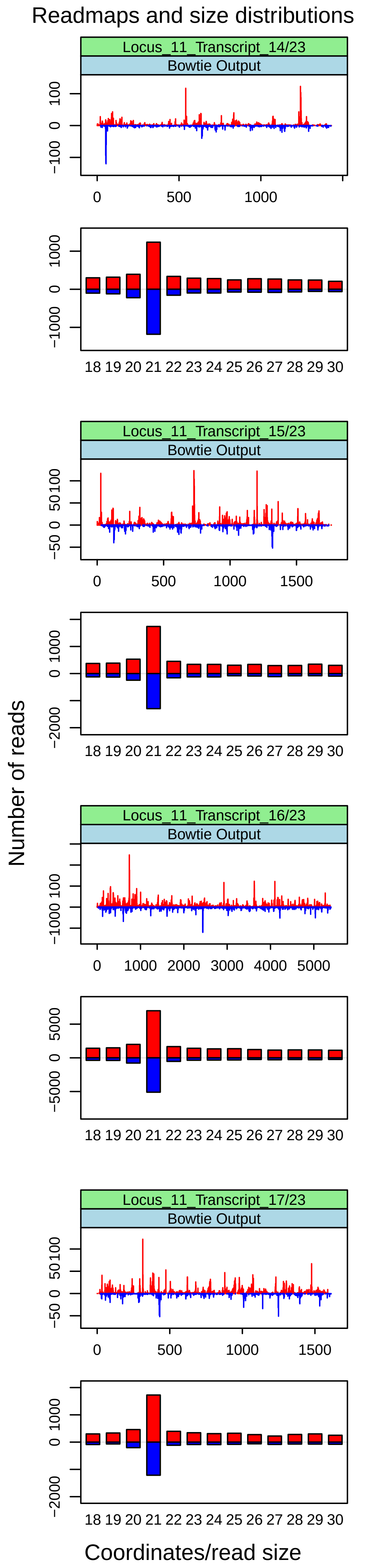
Readmaps and size distributions
Locus_11_Transcript_14/23
Bowtie Output
Locus_11_Transcript_15/23
Bowtie Output
Locus_11_Transcript_16/23
Bowtie Output
Locus_11_Transcript_17/23
Bowtie Output
18 19 20 21 22 23 24 25 26 27 28 29 30
Number of reads
Coordinates/read size

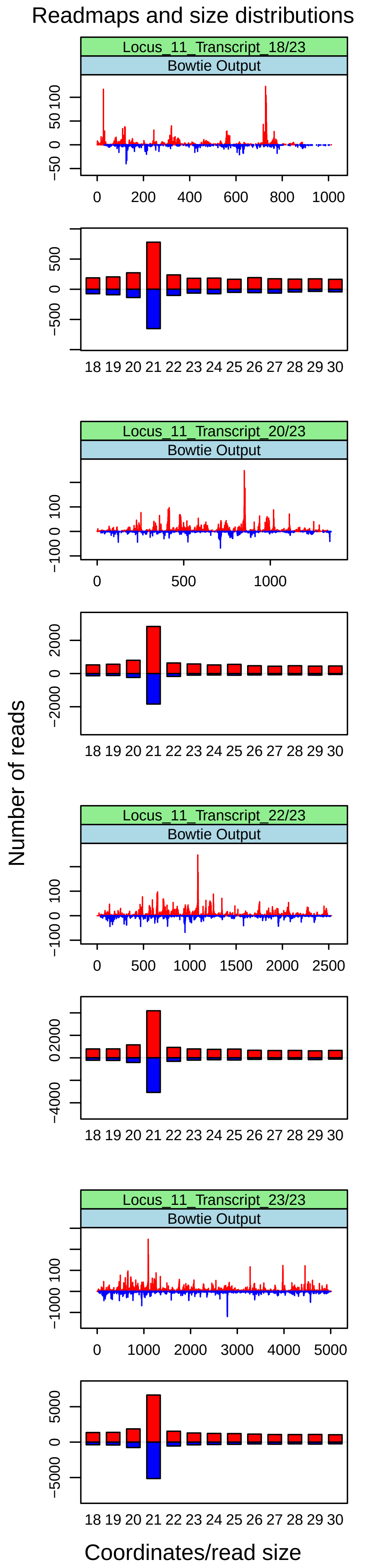
Readmaps and size distributions
Locus_11_Transcript_18/23
Bowtie Output
Locus_11_Transcript_20/23
Bowtie Output
Locus_11_Transcript_22/23
Bowtie Output
Locus_11_Transcript_23/23
Bowtie Output
18 19 20 21 22 23 24 25 26 27 28 29 30
Number of reads
Coordinates/read size

Readmaps and size distributions

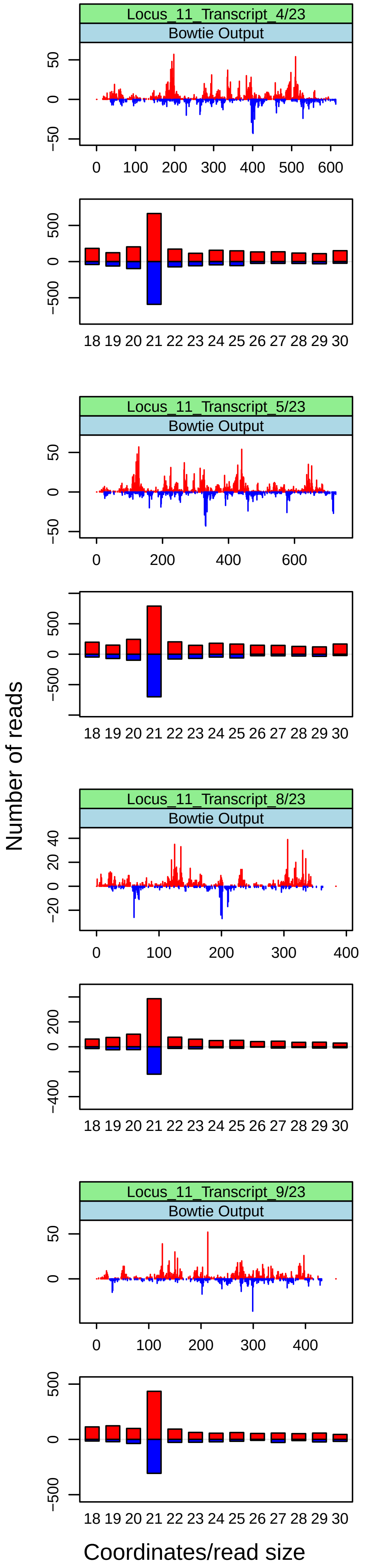

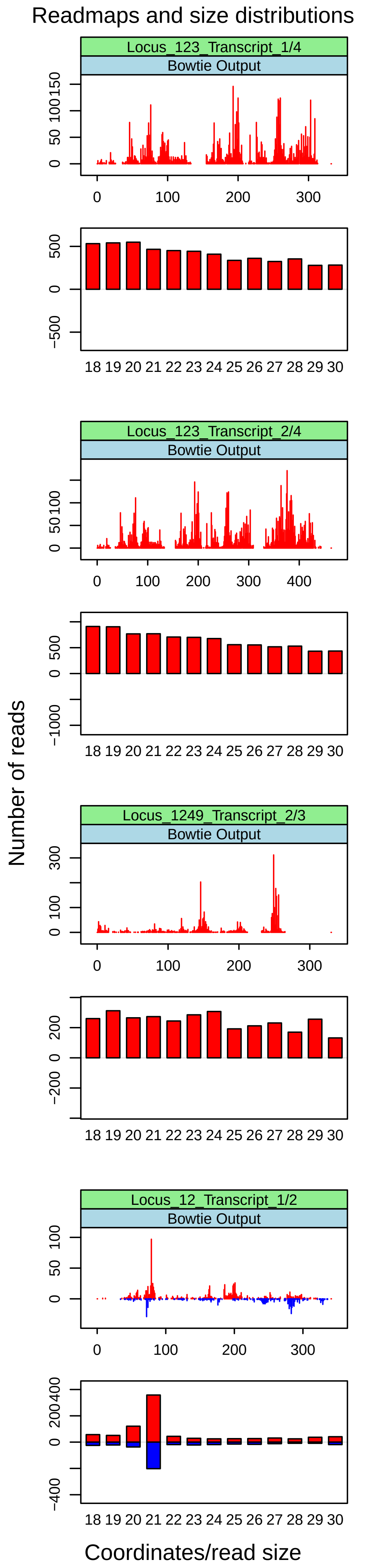
Readmaps and size distributions
Locus_123_Transcript_1/4
Bowtie Output
Locus_123_Transcript_2/4
Bowtie Output
Locus_1249_Transcript_2/3
Bowtie Output
Locus_12_Transcript_1/2
Bowtie Output
Number of reads
Coordinates/read size
18 19 20 21 22 23 24 25 26 27 28 29 30

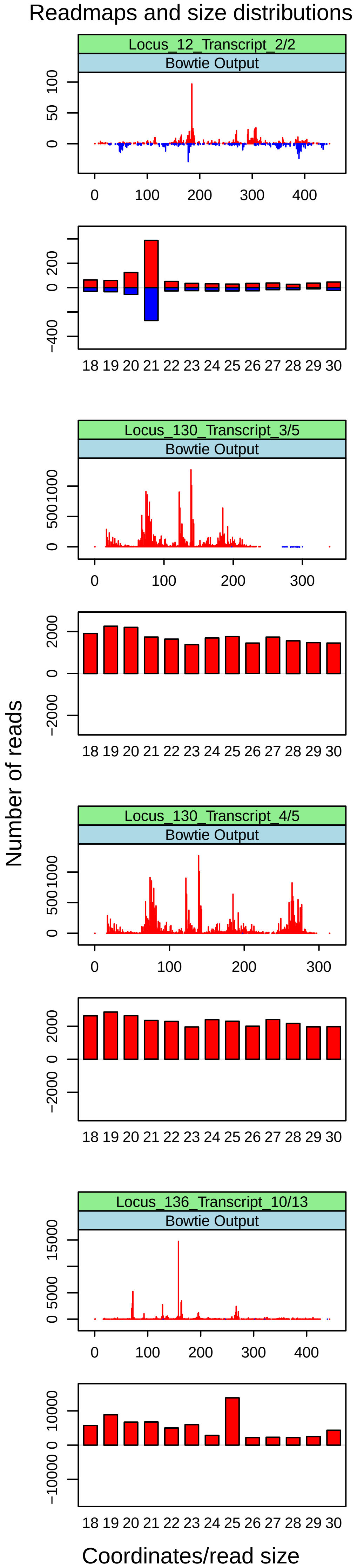
Readmaps and size distributions
Locus_12_Transcript_2/2
Bowtie Output
Locus_130_Transcript_3/5
Bowtie Output
Locus_130_Transcript_4/5
Bowtie Output
Locus_136_Transcript_10/13
Bowtie Output
Number of reads
Coordinates/read size

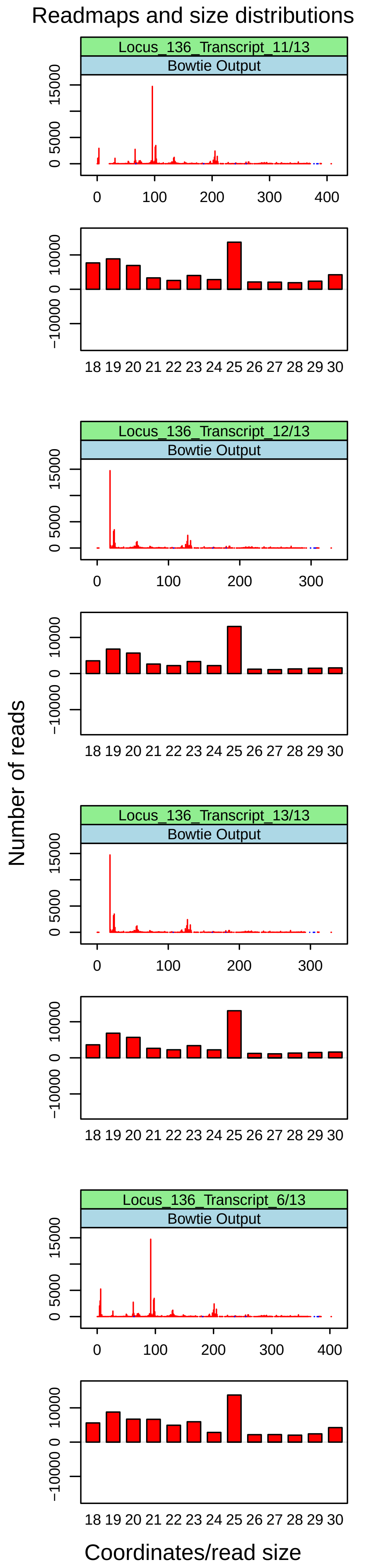

Readmaps and size distributions
Locus_136_Transcript_11/13
Bowtie Output
Locus_136_Transcript_12/13
Bowtie Output
Locus_136_Transcript_13/13
Bowtie Output
Locus_136_Transcript_6/13
Bowtie Output
Number of reads
Coordinates/read size

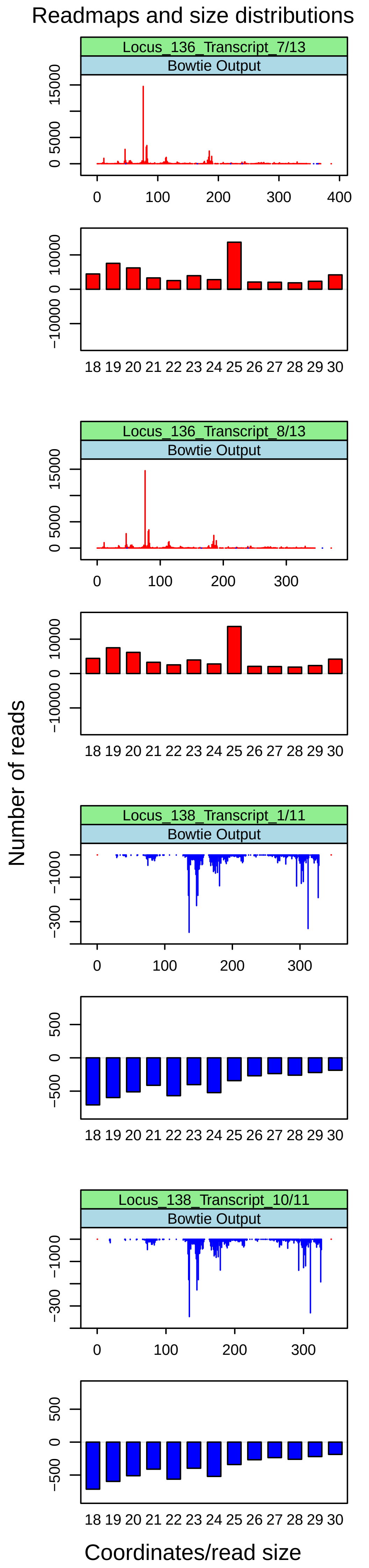

Readmaps and size distributions
Locus_136_Transcript_7/13
Bowtie Output
Locus_136_Transcript_8/13
Bowtie Output
Locus_138_Transcript_1/11
Bowtie Output
Locus_138_Transcript_10/11
Bowtie Output
Number of reads
Coordinates/read size

Readmaps and size distributions

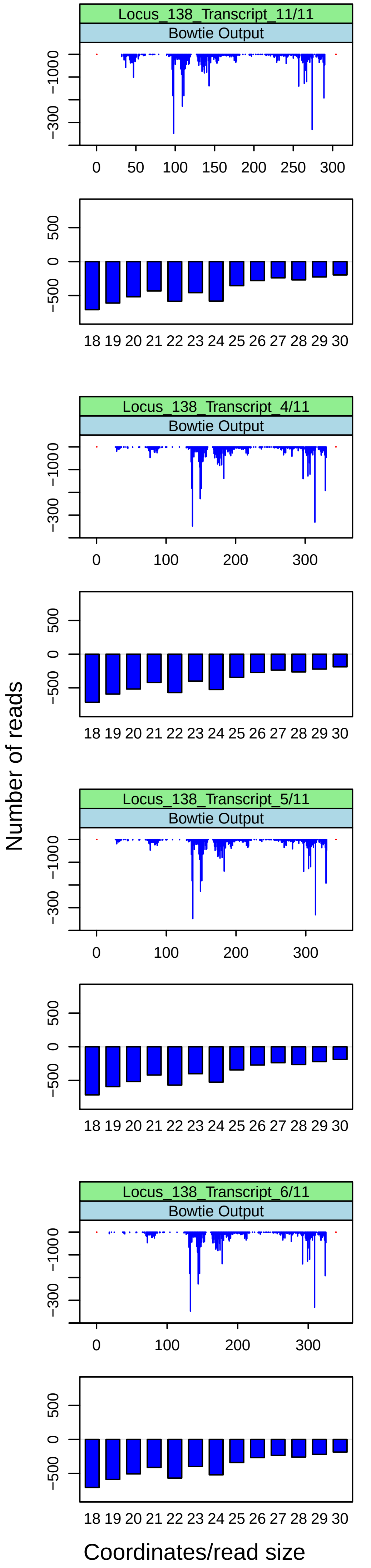

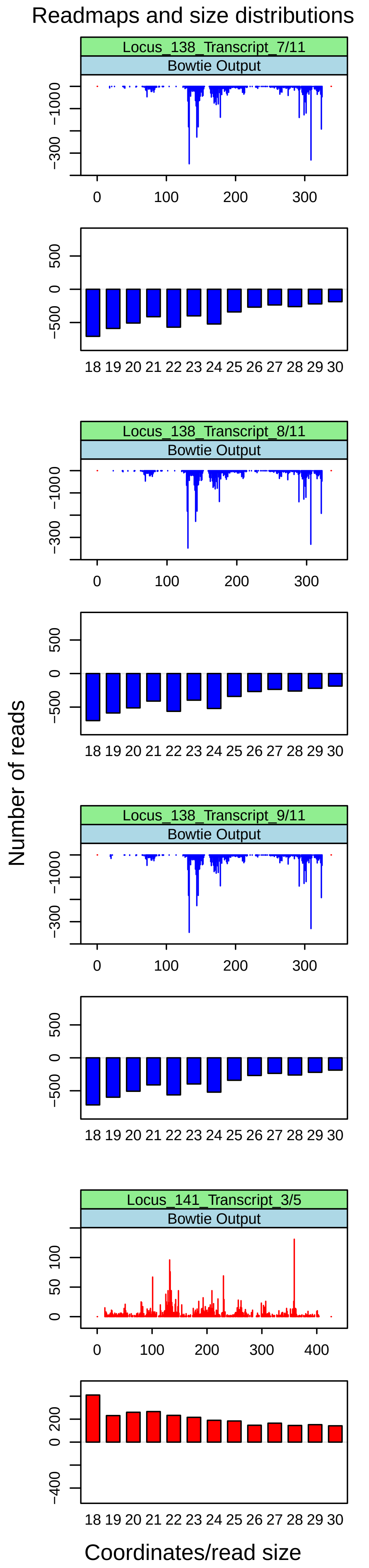

Readmaps and size distributions
Locus_138_Transcript_7/11
Bowtie Output
Locus_138_Transcript_8/11
Bowtie Output
Locus_138_Transcript_9/11
Bowtie Output
Locus_141_Transcript_3/5
Bowtie Output
Number of reads
Coordinates/read size

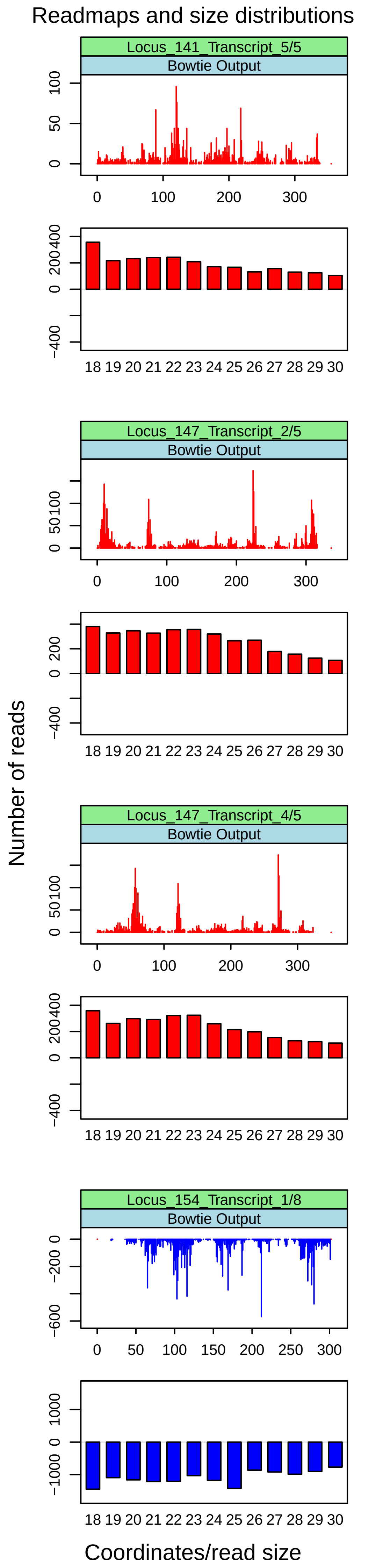

Readmaps and size distributions
Locus_141_Transcript_5/5
Bowtie Output
Locus_147_Transcript_2/5
Bowtie Output
Locus_147_Transcript_4/5
Bowtie Output
Locus_154_Transcript_1/8
Bowtie Output
Number of reads
Coordinates/read size
18 19 20 21 22 23 24 25 26 27 28 29 30

Readmaps and size distributions

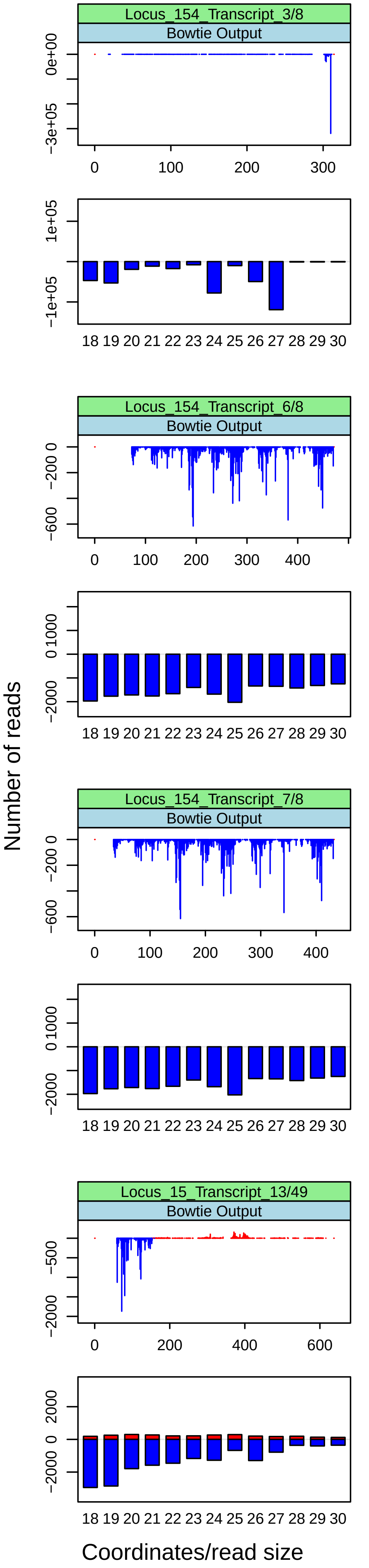

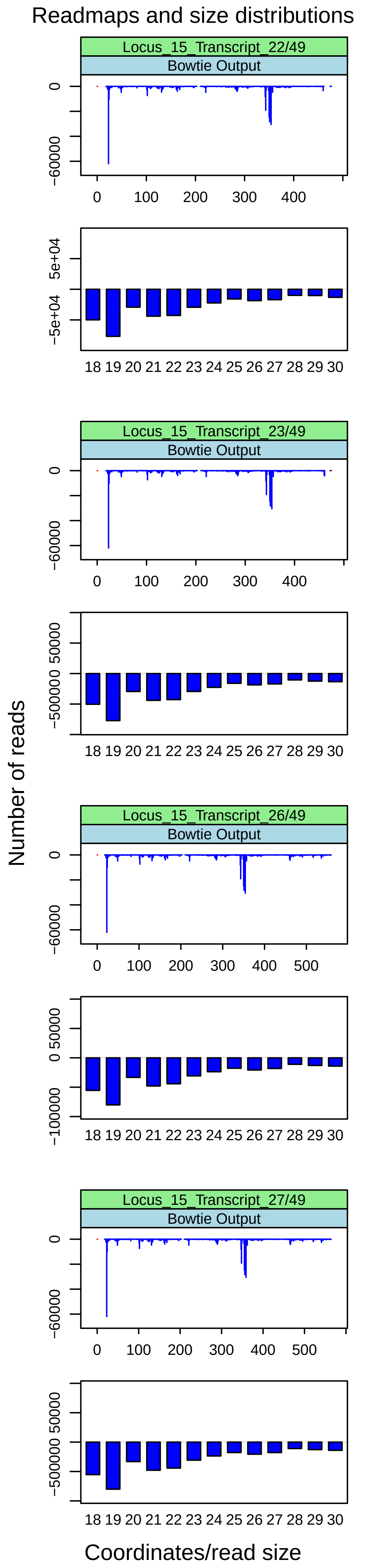

Readmaps and size distributions
Locus_15_Transcript_22/49
Bowtie Output
0
-60000
0 100 200 300 400
5e+04
-5e+04
18 19 20 21 22 23 24 25 26 27 28 29 30
Locus_15_Transcript_23/49
Bowtie Output
0
-60000
0 100 200 300 400
-500000 50000
18 19 20 21 22 23 24 25 26 27 28 29 30
Locus_15_Transcript_26/49
Bowtie Output
0
-60000
0 100 200 300 400 500
-100000 0 50000
18 19 20 21 22 23 24 25 26 27 28 29 30
Locus_15_Transcript_27/49
Bowtie Output
0
-60000
0 100 200 300 400 500
-500000 50000
18 19 20 21 22 23 24 25 26 27 28 29 30
Number of reads
Coordinates/read size

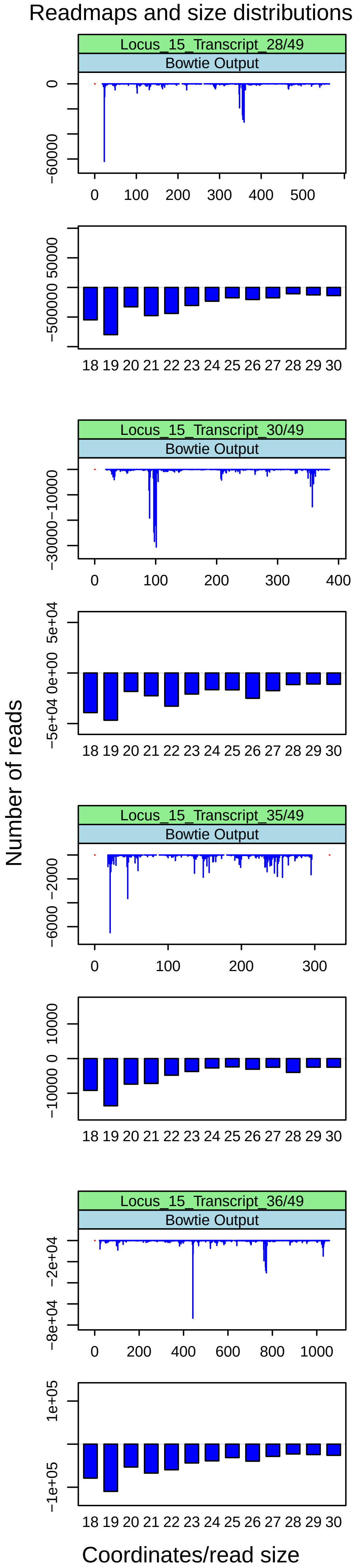

Readmaps and size distributions
Locus_15_Transcript_28/49
Bowtie Output
0
-60000
0 100 200 300 400 500
-500000 50000
18 19 20 21 22 23 24 25 26 27 28 29 30
Locus_15_Transcript_30/49
Bowtie Output
-30000 -10000
0 100 200 300 400
-5e+04 0e+00 5e+04
18 19 20 21 22 23 24 25 26 27 28 29 30
Locus_15_Transcript_35/49
Bowtie Output
-6000 -2000
0 100 200 300
-10000 0 10000
18 19 20 21 22 23 24 25 26 27 28 29 30
Locus_15_Transcript_36/49
Bowtie Output
-8e+04 -2e+04
0 200 400 600 800 1000
-1e+05 1e+05
18 19 20 21 22 23 24 25 26 27 28 29 30
Number of reads
Coordinates/read size

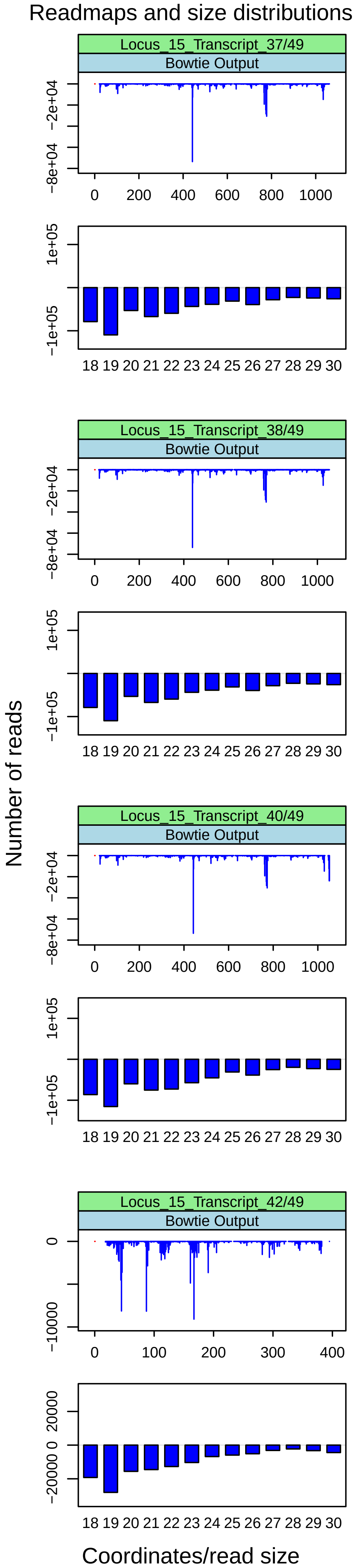
Readmaps and size distributions
Locus_15_Transcript_37/49
Bowtie Output
Locus_15_Transcript_38/49
Bowtie Output
Locus_15_Transcript_40/49
Bowtie Output
Locus_15_Transcript_42/49
Bowtie Output
Number of reads
Coordinates/read size

Readmaps and size distributions

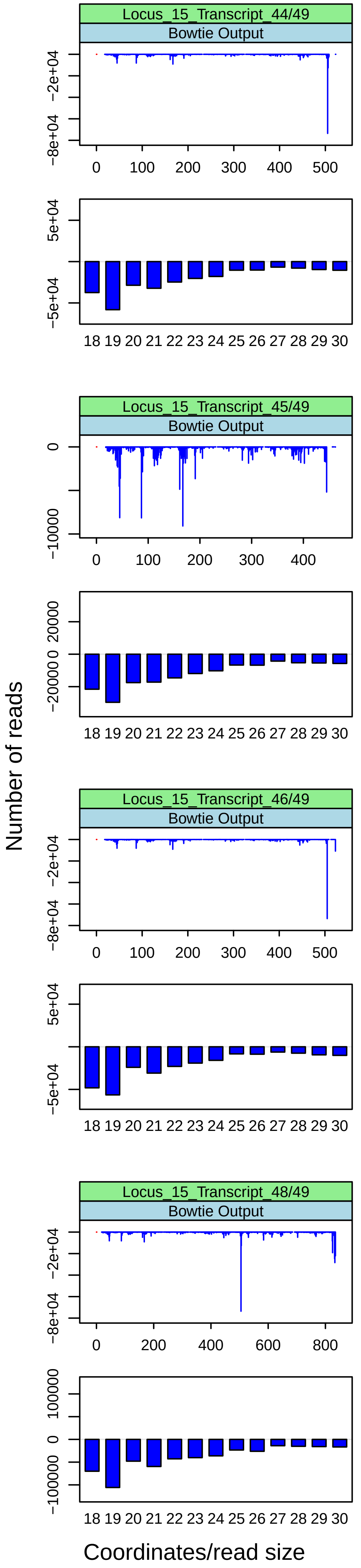

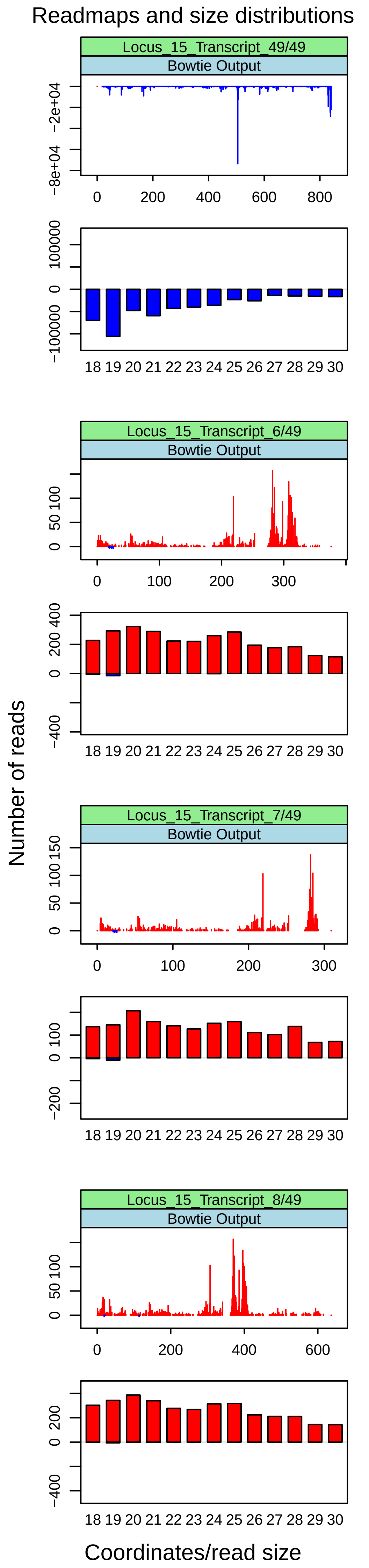
Readmaps and size distributions
Locus_15_Transcript_49/49
Bowtie Output
Locus_15_Transcript_6/49
Bowtie Output
Locus_15_Transcript_7/49
Bowtie Output
Locus_15_Transcript_8/49
Bowtie Output
Number of reads
Coordinates/read size

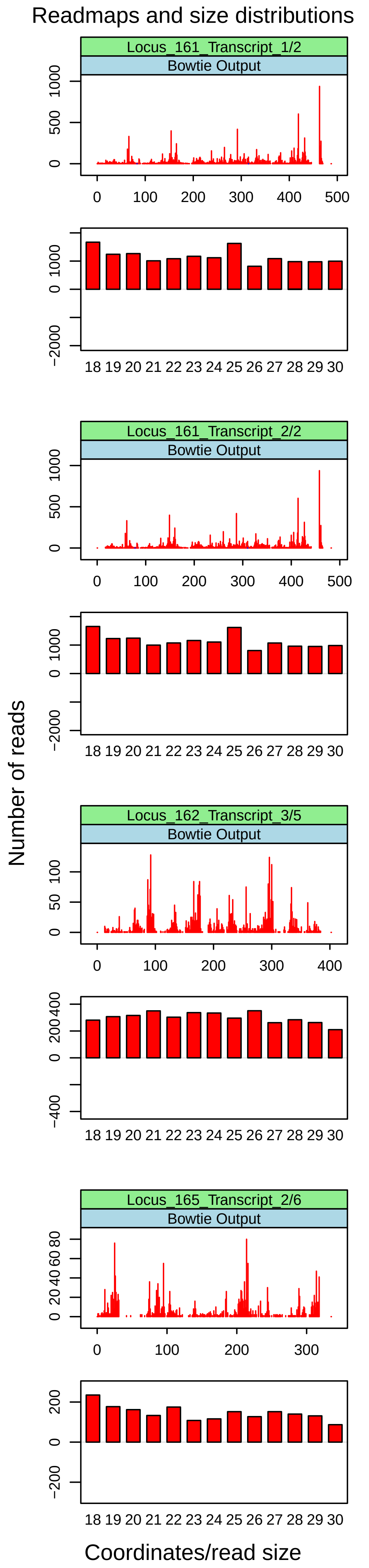

Readmaps and size distributions
Locus_161_Transcript_1/2
Bowtie Output
Locus_161_Transcript_2/2
Bowtie Output
Locus_162_Transcript_3/5
Bowtie Output
Locus_165_Transcript_2/6
Bowtie Output
Number of reads
Coordinates/read size

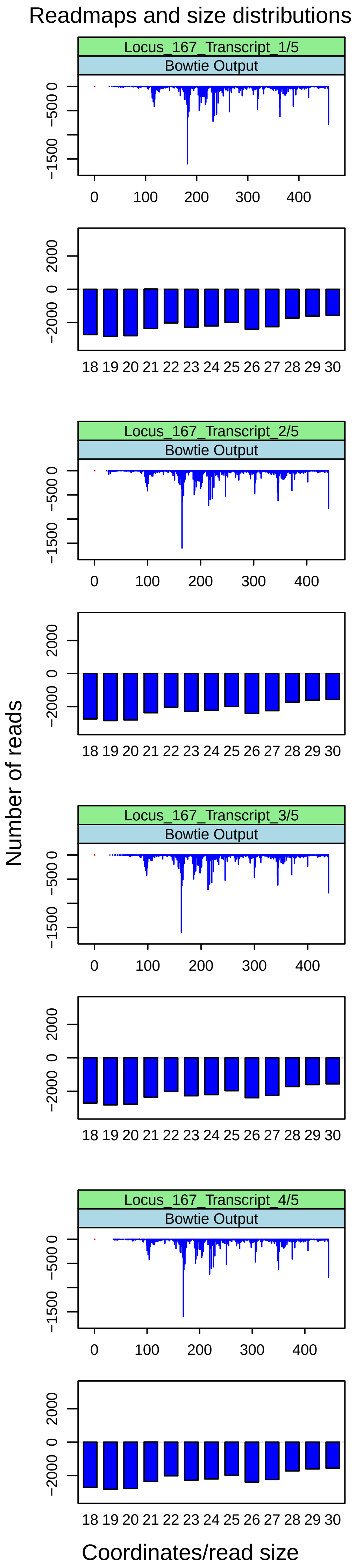

Readmaps and size distributions
Locus_167_Transcript_1/5
Bowtie Output
Locus_167_Transcript_2/5
Bowtie Output
Locus_167_Transcript_3/5
Bowtie Output
Locus_167_Transcript_4/5
Bowtie Output
Number of reads
Coordinates/read size

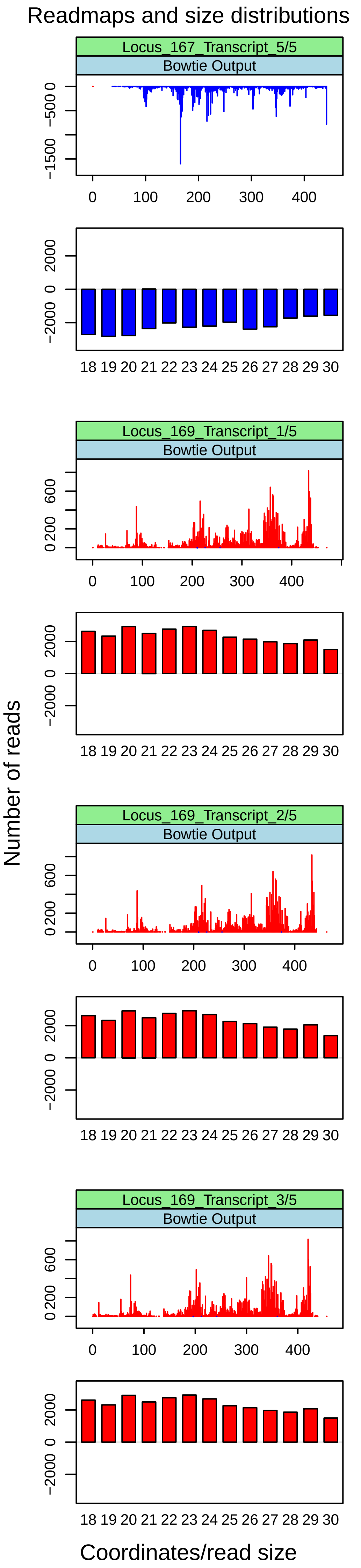

Readmaps and size distributions
Locus_167_Transcript_5/5
Bowtie Output
Locus_169_Transcript_1/5
Bowtie Output
Locus_169_Transcript_2/5
Bowtie Output
Locus_169_Transcript_3/5
Bowtie Output
Number of reads
Coordinates/read size

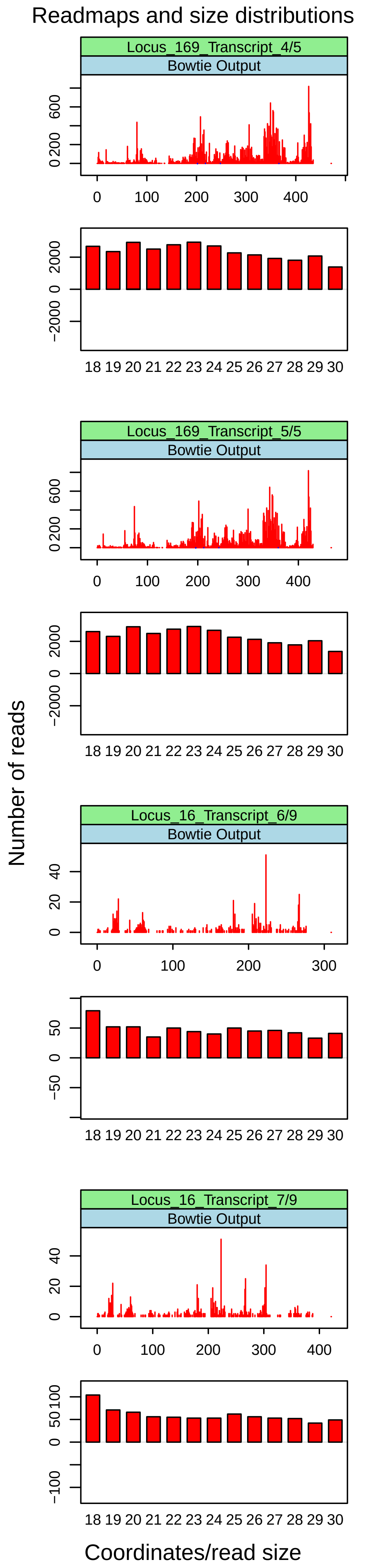

Readmaps and size distributions
Locus_169_Transcript_4/5
Bowtie Output
Locus_169_Transcript_5/5
Bowtie Output
Locus_16_Transcript_6/9
Bowtie Output
Locus_16_Transcript_7/9
Bowtie Output
Number of reads
Coordinates/read size

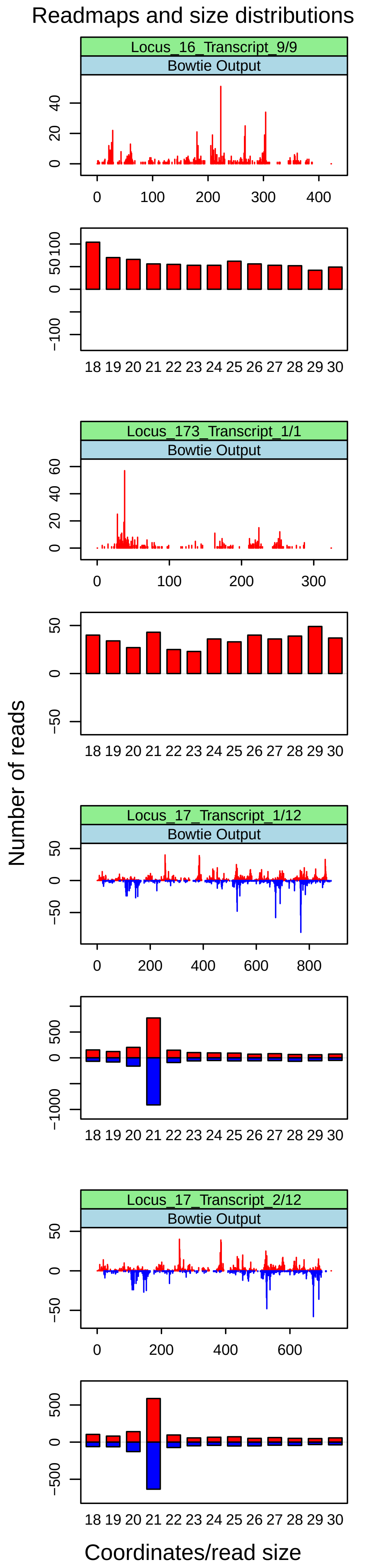

Readmaps and size distributions
Locus_16_Transcript_9/9
Bowtie Output
Locus_173_Transcript_1/1
Bowtie Output
Locus_17_Transcript_1/12
Bowtie Output
Locus_17_Transcript_2/12
Bowtie Output
Number of reads
Coordinates/read size

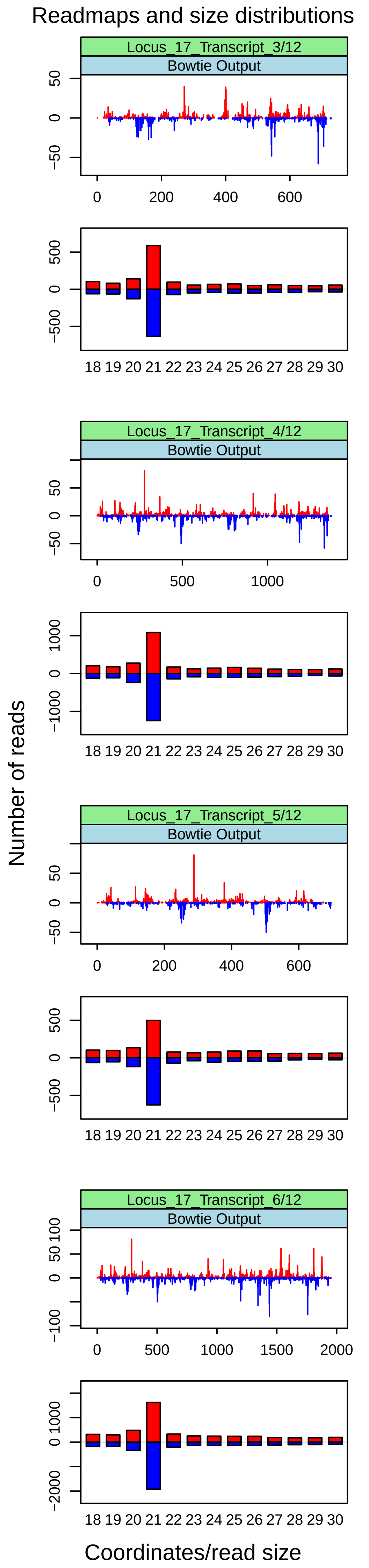
Readmaps and size distributions
Locus_17_Transcript_3/12
Bowtie Output
50
0
−50
0
200
400
600
500
0
−500
18 19 20 21 22 23 24 25 26 27 28 29 30
Locus_17_Transcript_4/12
Bowtie Output
50
0
−50
0
500
1000
1000
0
−1000
18 19 20 21 22 23 24 25 26 27 28 29 30
Locus_17_Transcript_5/12
Bowtie Output
50
0
−50
0
200
400
600
500
0
−500
18 19 20 21 22 23 24 25 26 27 28 29 30
Locus_17_Transcript_6/12
Bowtie Output
100
50
0
−100
0
500
1000
1500
2000
1000
0
−2000
18 19 20 21 22 23 24 25 26 27 28 29 30
Number of reads
Coordinates/read size

Readmaps and size distributions

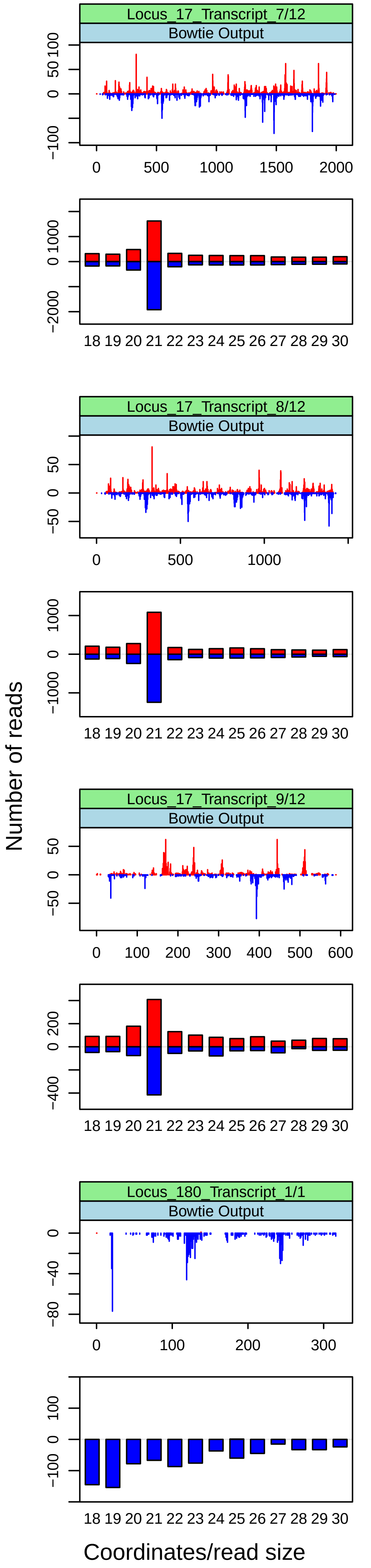

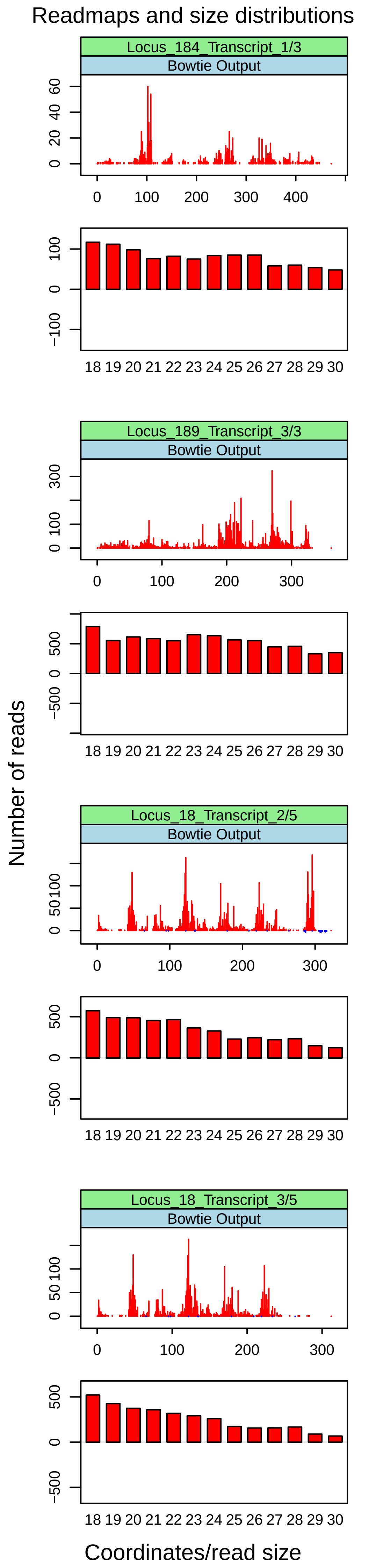

Readmaps and size distributions
Locus_184_Transcript_1/3
Bowtie Output
0 20 40 60
0 100 200 300 400
-100 0 100
18 19 20 21 22 23 24 25 26 27 28 29 30
Locus_189_Transcript_3/3
Bowtie Output
0 100 300
0 100 200 300
-500 0 500
18 19 20 21 22 23 24 25 26 27 28 29 30
Locus_18_Transcript_2/5
Bowtie Output
0 50 100
0 100 200 300
-500 0 500
18 19 20 21 22 23 24 25 26 27 28 29 30
Locus_18_Transcript_3/5
Bowtie Output
0 50 100
0 100 200 300
-500 0 500
18 19 20 21 22 23 24 25 26 27 28 29 30
Number of reads
Coordinates/read size

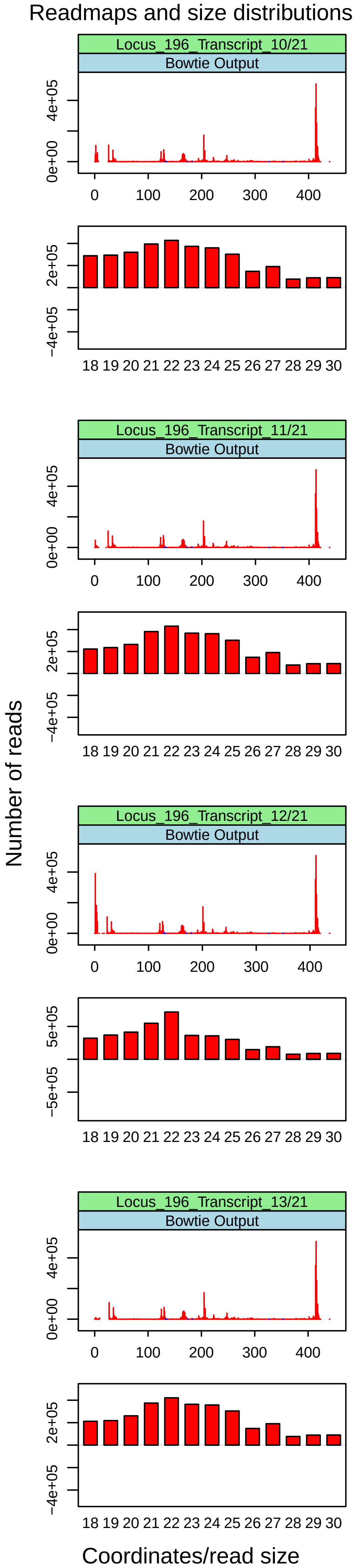
Readmaps and size distributions
Locus_196_Transcript_10/21
Bowtie Output
0e+00
4e+05
0
100
200
300
400
−4e+05
2e+05
18 19 20 21 22 23 24 25 26 27 28 29 30
Locus_196_Transcript_11/21
Bowtie Output
0e+00
4e+05
0
100
200
300
400
−4e+05
2e+05
18 19 20 21 22 23 24 25 26 27 28 29 30
Locus_196_Transcript_12/21
Bowtie Output
0e+00
4e+05
0
100
200
300
400
−5e+05
5e+05
18 19 20 21 22 23 24 25 26 27 28 29 30
Locus_196_Transcript_13/21
Bowtie Output
0e+00
4e+05
0
100
200
300
400
−4e+05
2e+05
18 19 20 21 22 23 24 25 26 27 28 29 30
Number of reads
Coordinates/read size

Readmaps and size distributions

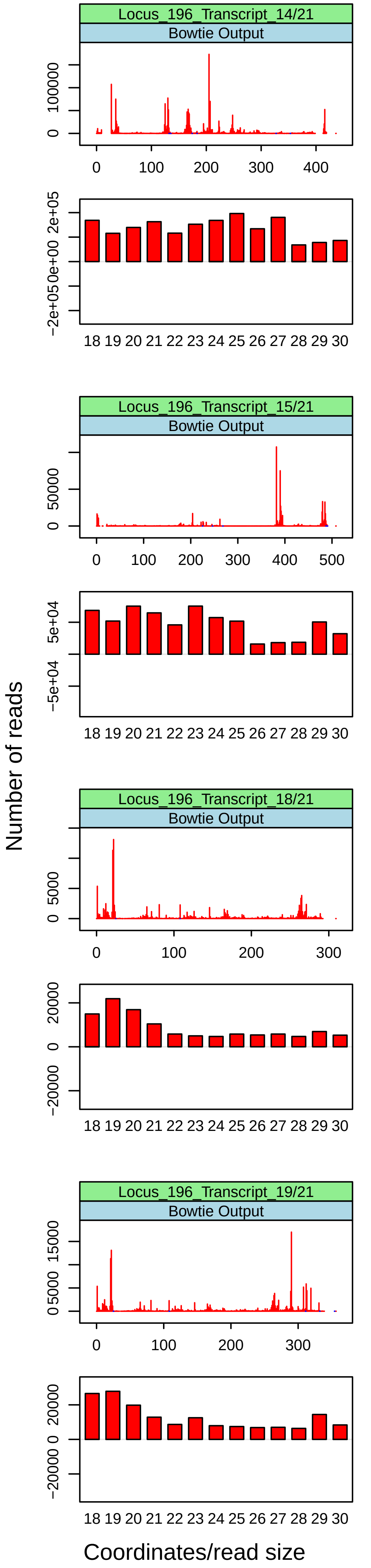

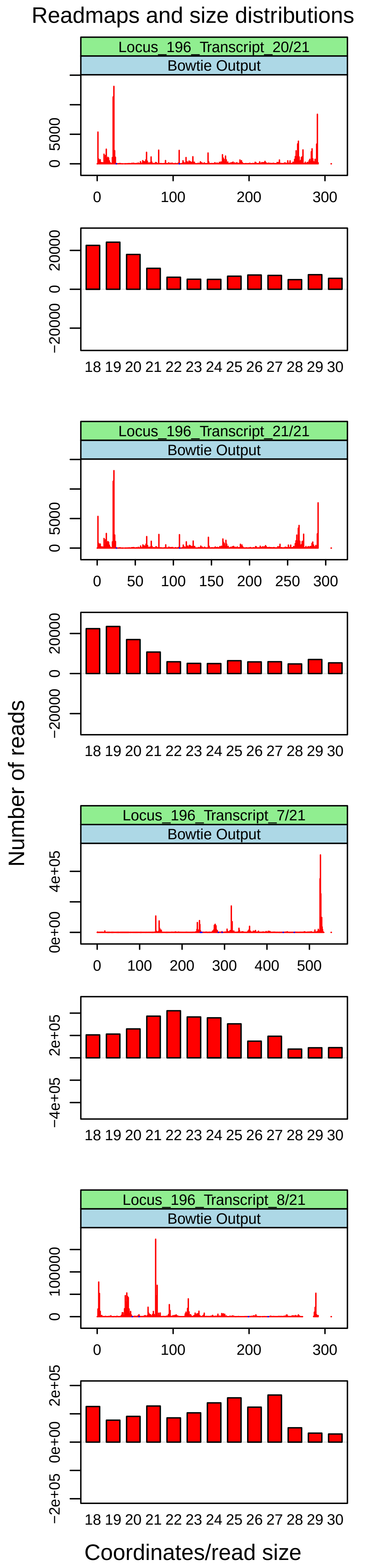

Readmaps and size distributions
Locus_196_Transcript_20/21
Bowtie Output
Locus_196_Transcript_21/21
Bowtie Output
Locus_196_Transcript_7/21
Bowtie Output
Locus_196_Transcript_8/21
Bowtie Output
Number of reads
Coordinates/read size

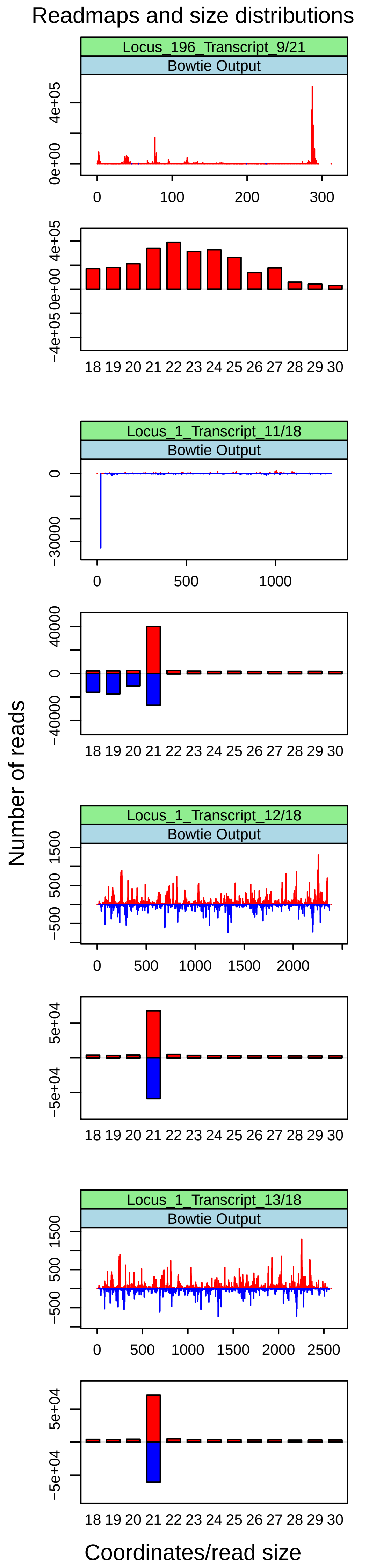
Readmaps and size distributions
Locus_196_Transcript_9/21
Bowtie Output
Locus_1_Transcript_11/18
Bowtie Output
Locus_1_Transcript_12/18
Bowtie Output
Locus_1_Transcript_13/18
Bowtie Output
Number of reads
Coordinates/read size
18 19 20 21 22 23 24 25 26 27 28 29 30

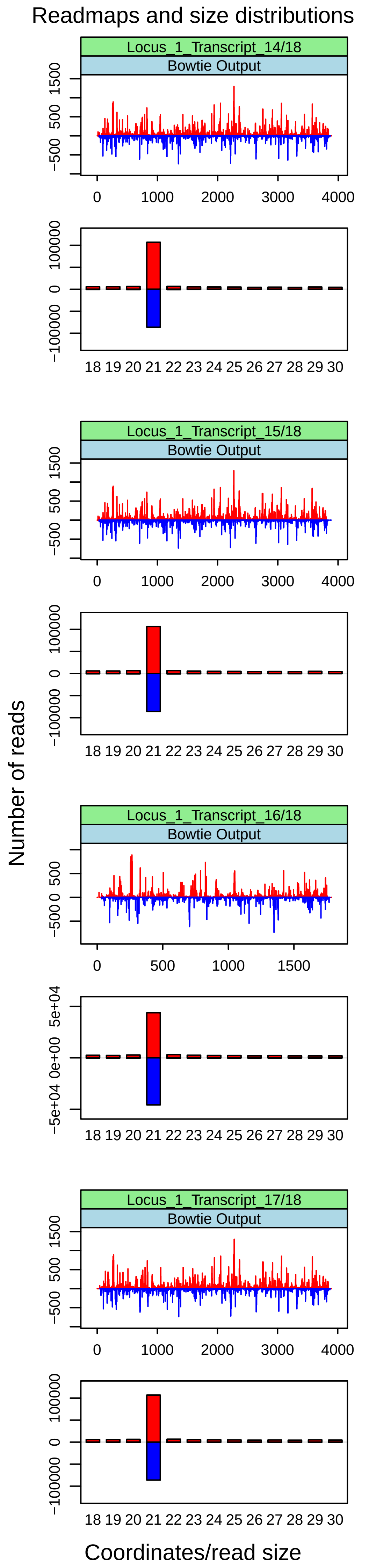
Readmaps and size distributions
Locus_1_Transcript_14/18
Bowtie Output
Locus_1_Transcript_15/18
Bowtie Output
Locus_1_Transcript_16/18
Bowtie Output
Locus_1_Transcript_17/18
Bowtie Output
Number of reads
Coordinates/read size

Readmaps and size distributions

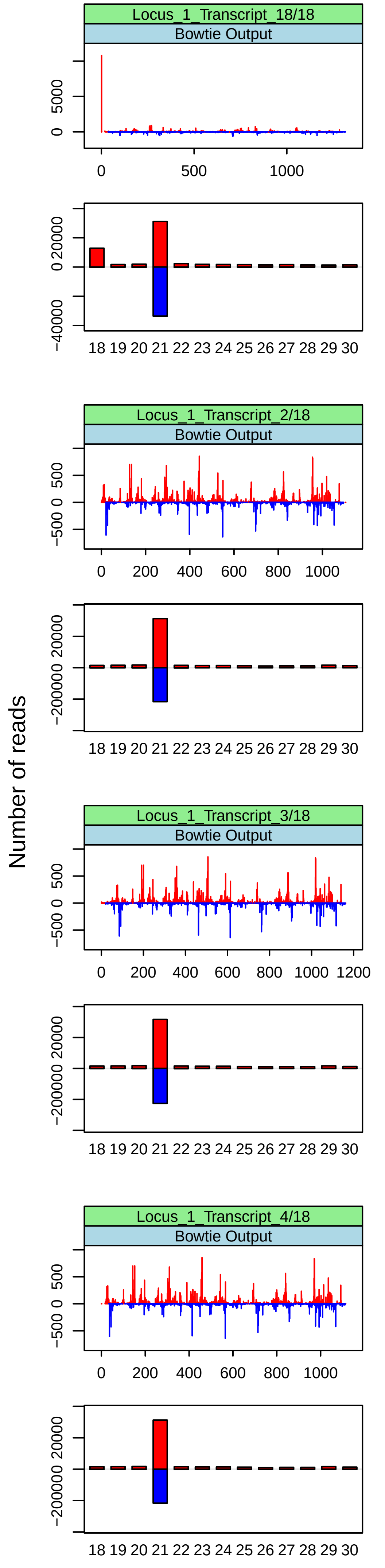

Coordinates/read size

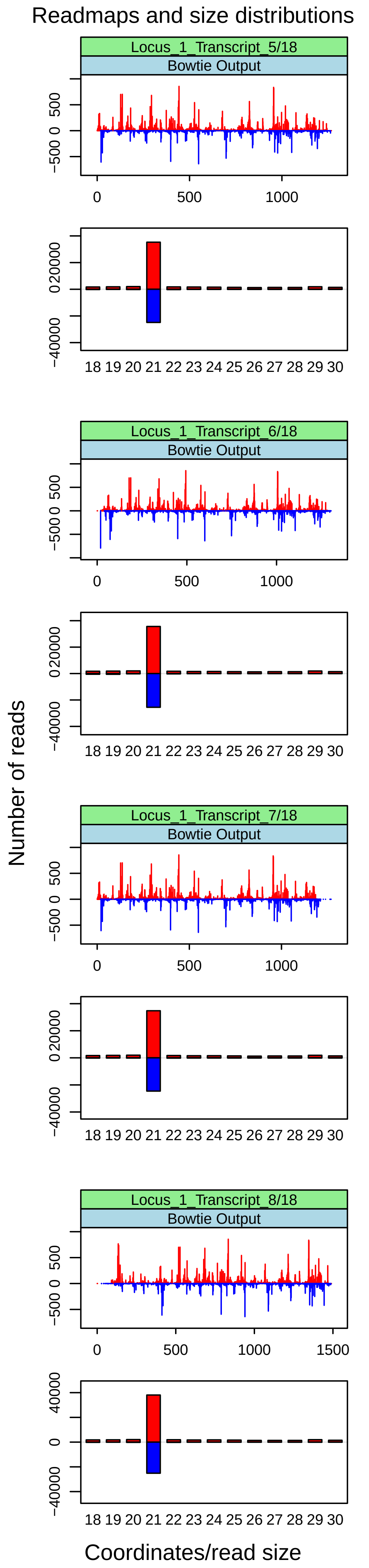
Readmaps and size distributions
Locus_1_Transcript_5/18
Bowtie Output
Locus_1_Transcript_6/18
Bowtie Output
Locus_1_Transcript_7/18
Bowtie Output
Locus_1_Transcript_8/18
Bowtie Output
Number of reads
Coordinates/read size
18 19 20 21 22 23 24 25 26 27 28 29 30

Readmaps and size distributions

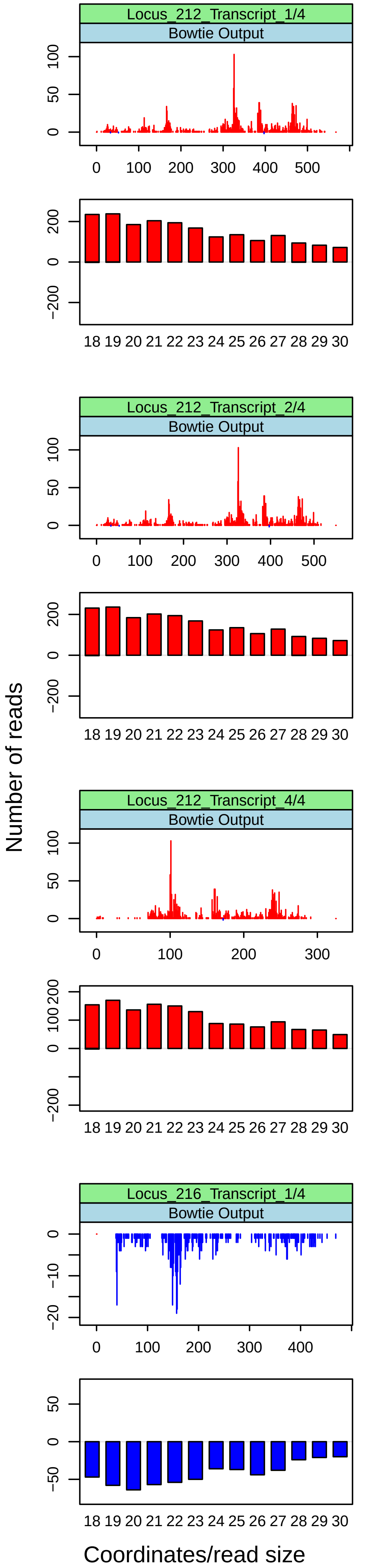

Readmaps and size distributions

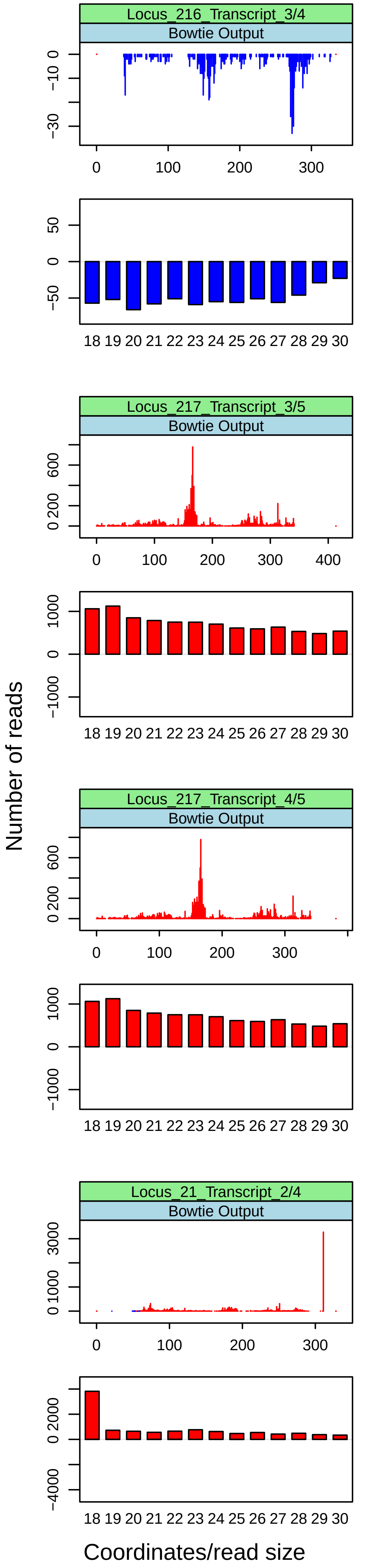

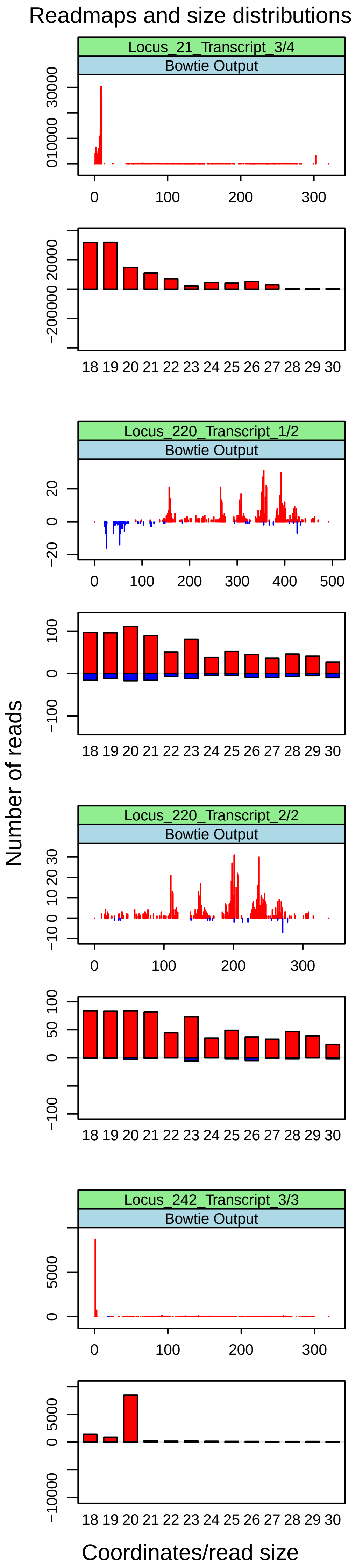

Readmaps and size distributions
Locus_21_Transcript_3/4
Bowtie Output
Locus_220_Transcript_1/2
Bowtie Output
Locus_220_Transcript_2/2
Bowtie Output
Locus_242_Transcript_3/3
Bowtie Output
Number of reads
Coordinates/read size
18 19 20 21 22 23 24 25 26 27 28 29 30

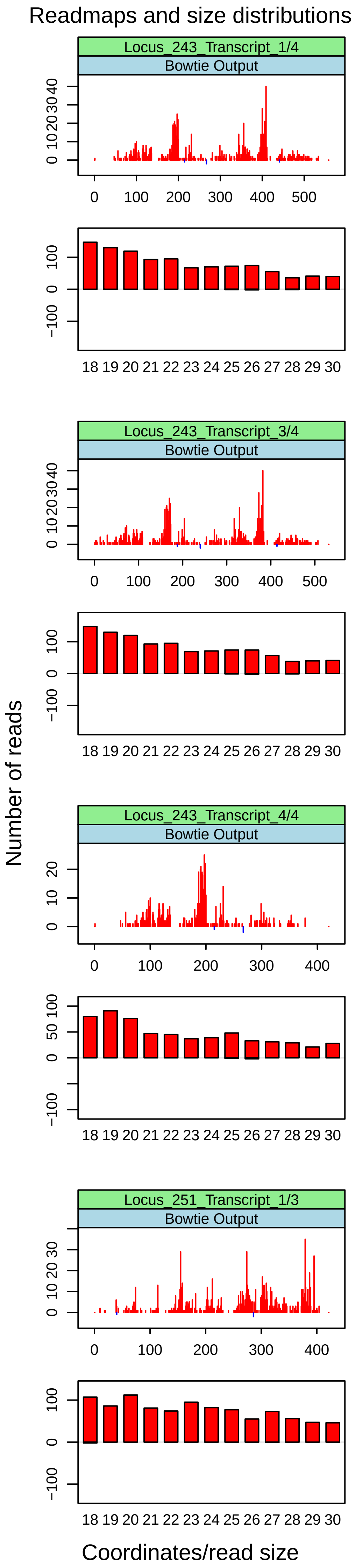
Readmaps and size distributions
Locus_243_Transcript_1/4
Bowtie Output
Locus_243_Transcript_3/4
Bowtie Output
Locus_243_Transcript_4/4
Bowtie Output
Locus_251_Transcript_1/3
Bowtie Output
18 19 20 21 22 23 24 25 26 27 28 29 30
Number of reads
Coordinates/read size

Readmaps and size distributions

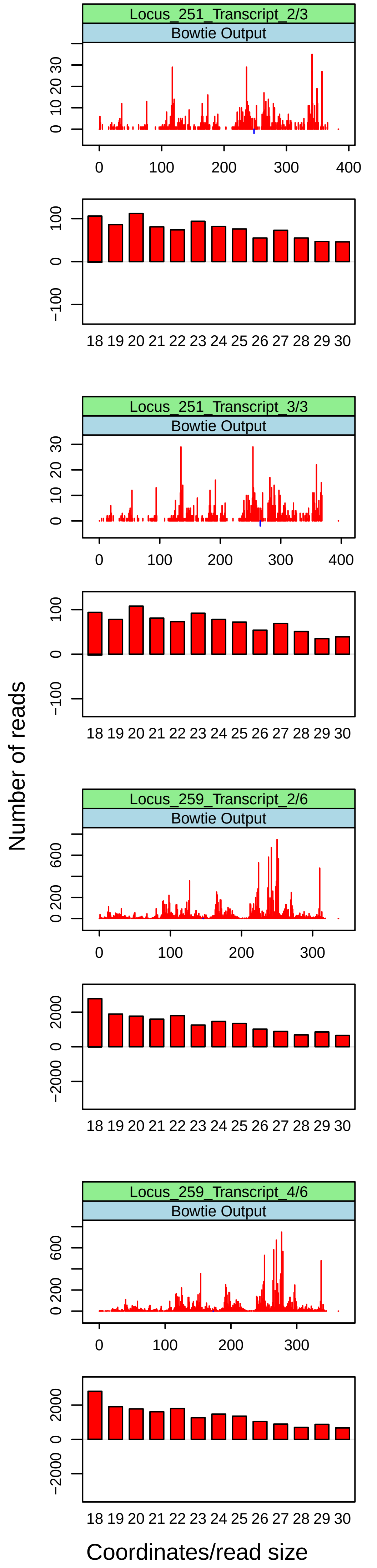

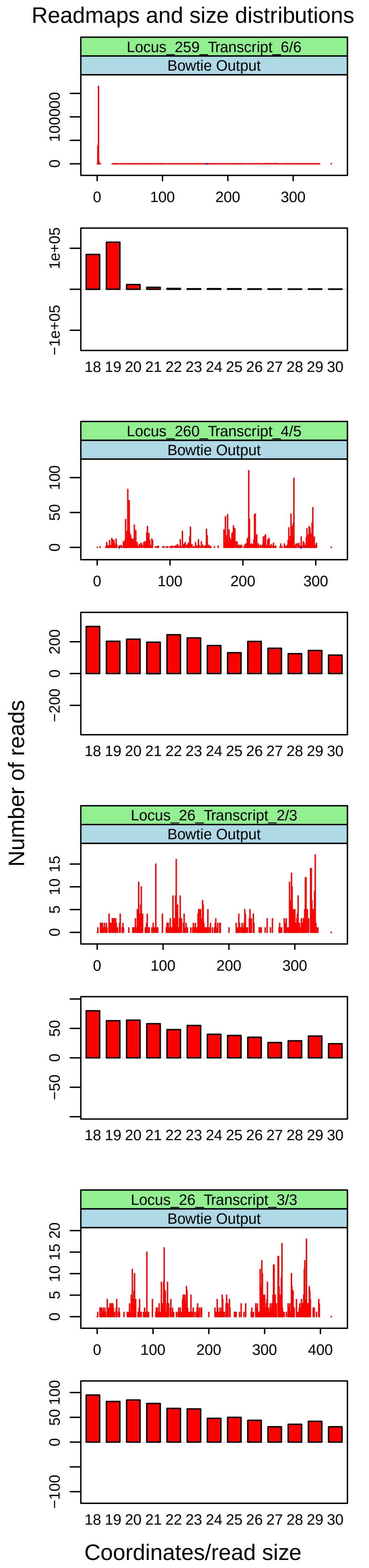
Readmaps and size distributions
Locus_259_Transcript_6/6
Bowtie Output
Locus_260_Transcript_4/5
Bowtie Output
Locus_26_Transcript_2/3
Bowtie Output
Locus_26_Transcript_3/3
Bowtie Output
Number of reads
Coordinates/read size

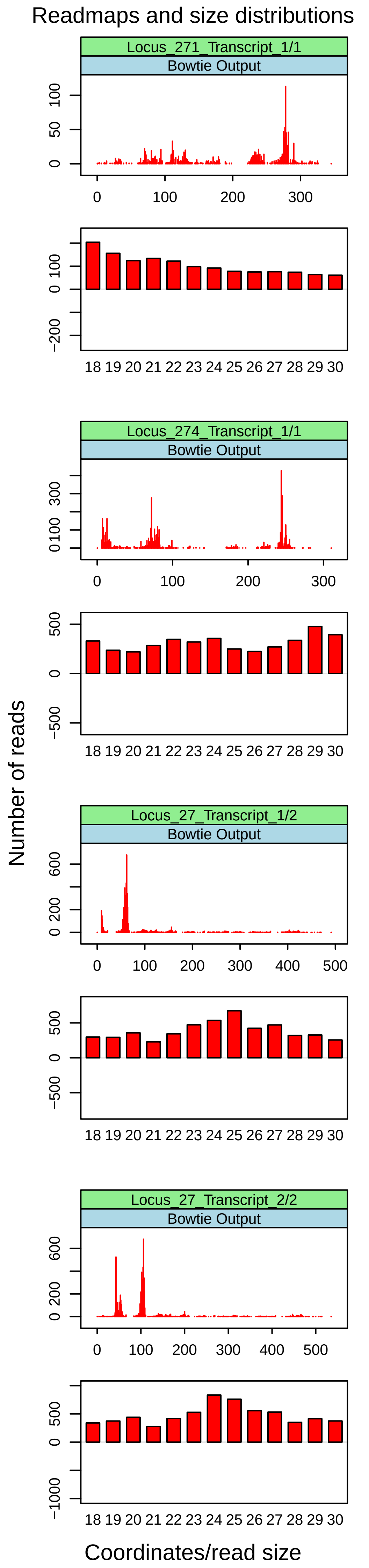

Readmaps and size distributions
Locus_271_Transcript_1/1
Bowtie Output
Locus_274_Transcript_1/1
Bowtie Output
Locus_27_Transcript_1/2
Bowtie Output
Locus_27_Transcript_2/2
Bowtie Output
Number of reads
Coordinates/read size
18 19 20 21 22 23 24 25 26 27 28 29 30

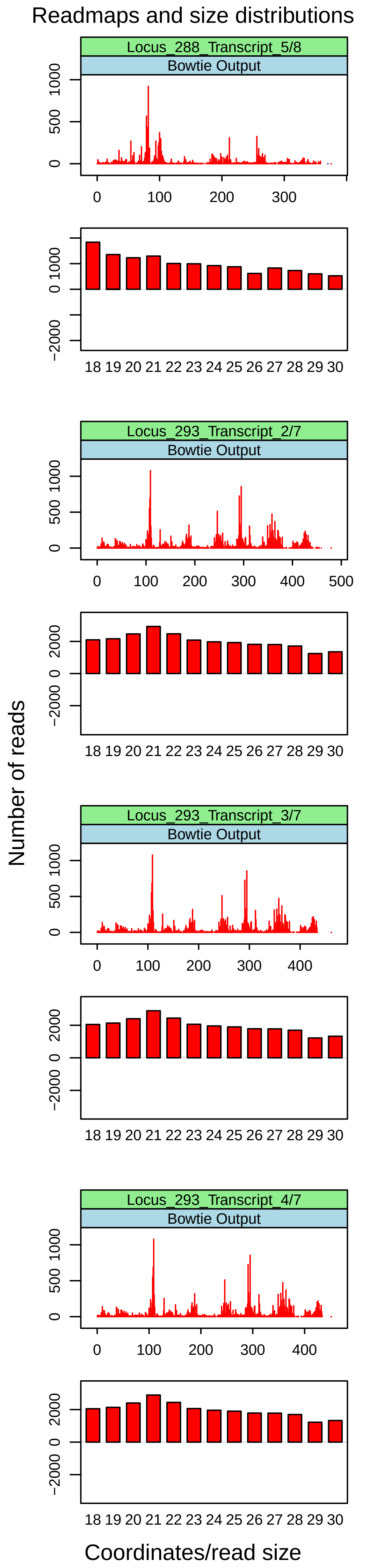
Readmaps and size distributions
Locus_288_Transcript_5/8
Bowtie Output
Locus_293_Transcript_2/7
Bowtie Output
Locus_293_Transcript_3/7
Bowtie Output
Locus_293_Transcript_4/7
Bowtie Output
Number of reads
Coordinates/read size

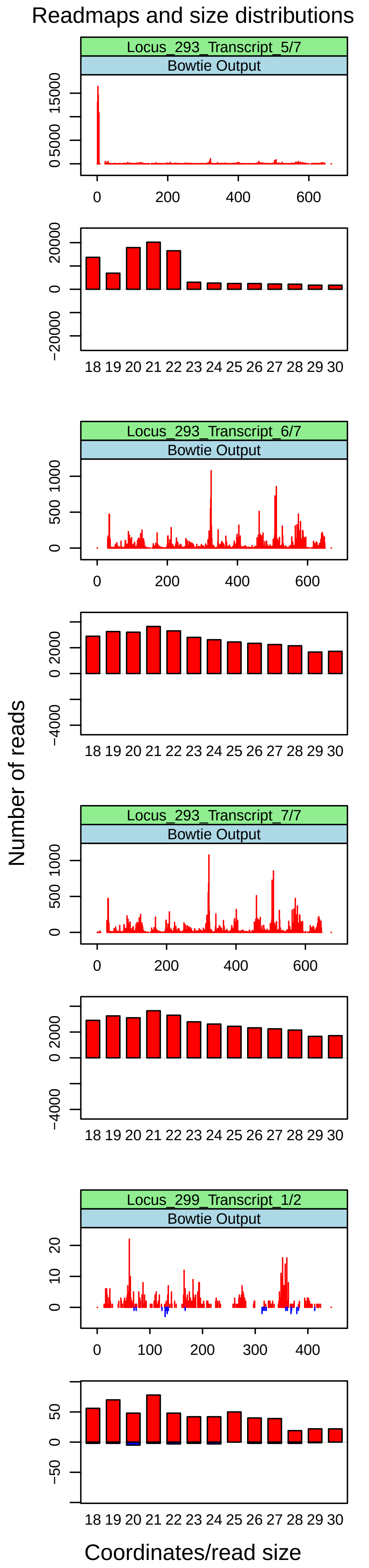

Readmaps and size distributions
Locus_293_Transcript_5/7
Bowtie Output
Locus_293_Transcript_6/7
Bowtie Output
Locus_293_Transcript_7/7
Bowtie Output
Locus_299_Transcript_1/2
Bowtie Output
Number of reads
Coordinates/read size

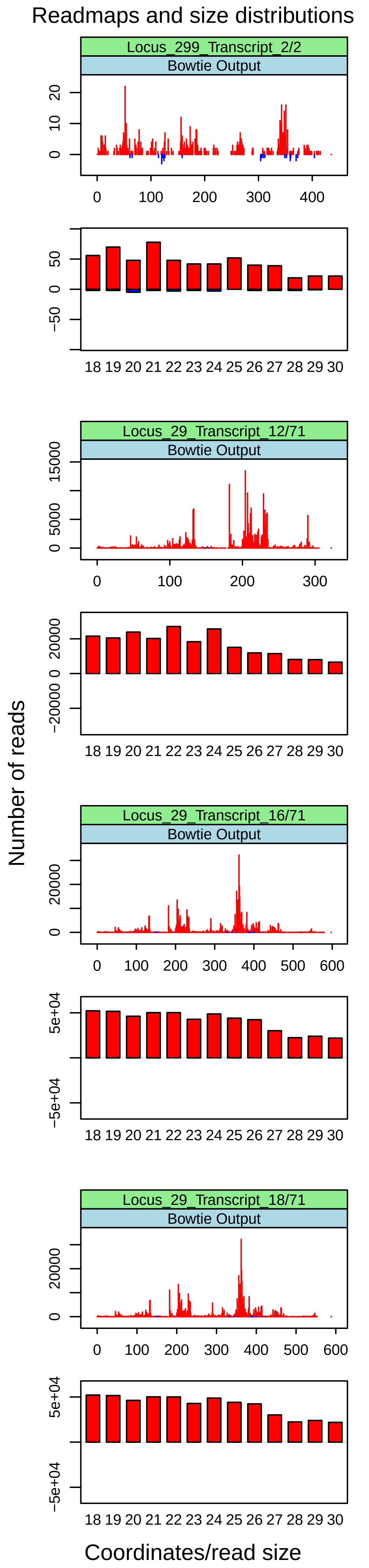
Readmaps and size distributions
Locus_299_Transcript_2/2
Bowtie Output
Locus_29_Transcript_12/71
Bowtie Output
Locus_29_Transcript_16/71
Bowtie Output
Locus_29_Transcript_18/71
Bowtie Output
Number of reads
Coordinates/read size

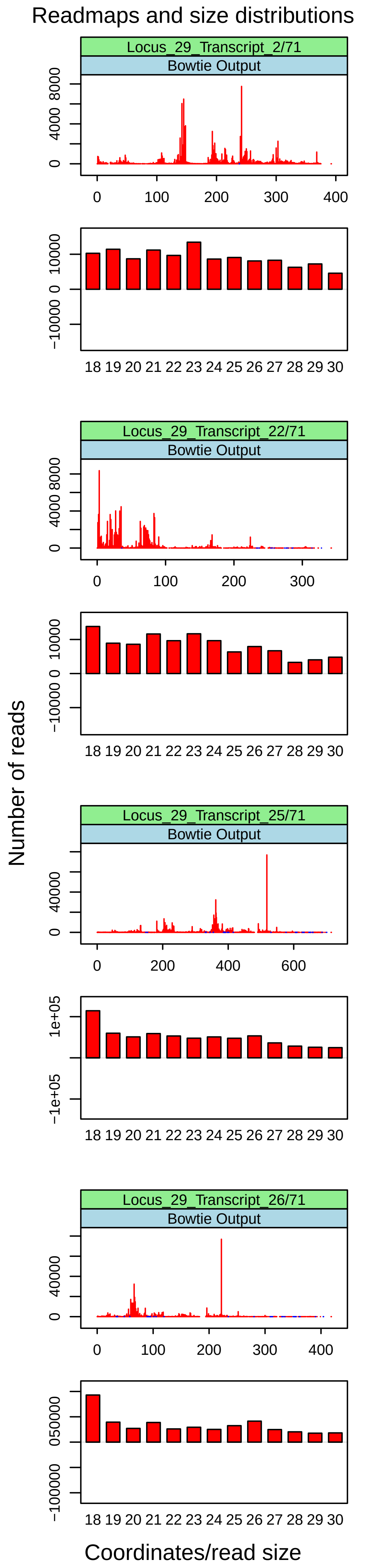

Readmaps and size distributions
Locus_29_Transcript_2/71
Bowtie Output
Locus_29_Transcript_22/71
Bowtie Output
Locus_29_Transcript_25/71
Bowtie Output
Locus_29_Transcript_26/71
Bowtie Output
18 19 20 21 22 23 24 25 26 27 28 29 30
Number of reads
Coordinates/read size

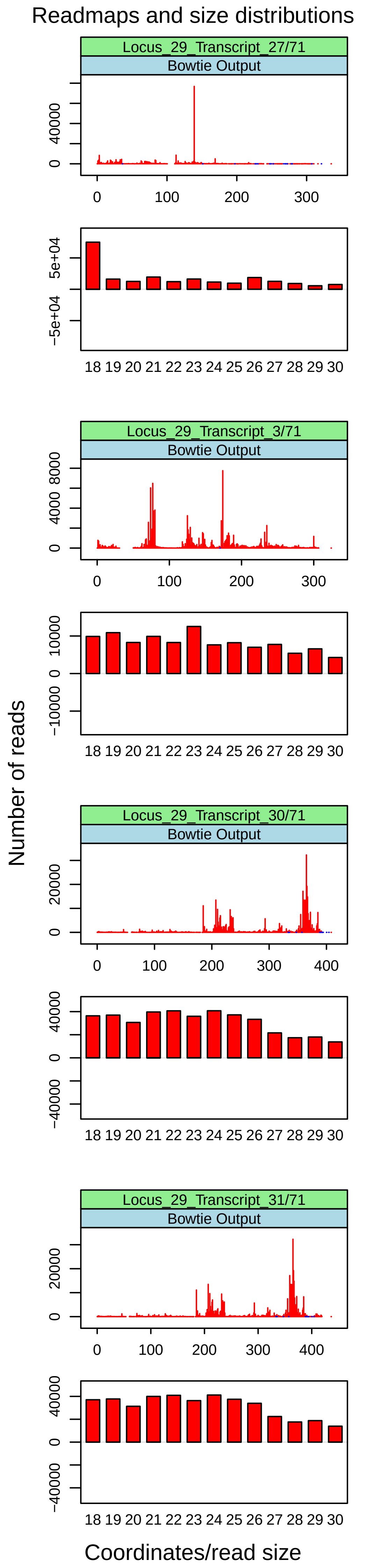

Readmaps and size distributions
Locus_29_Transcript_27/71
Bowtie Output
Locus_29_Transcript_3/71
Bowtie Output
Locus_29_Transcript_30/71
Bowtie Output
Locus_29_Transcript_31/71
Bowtie Output
Number of reads
Coordinates/read size

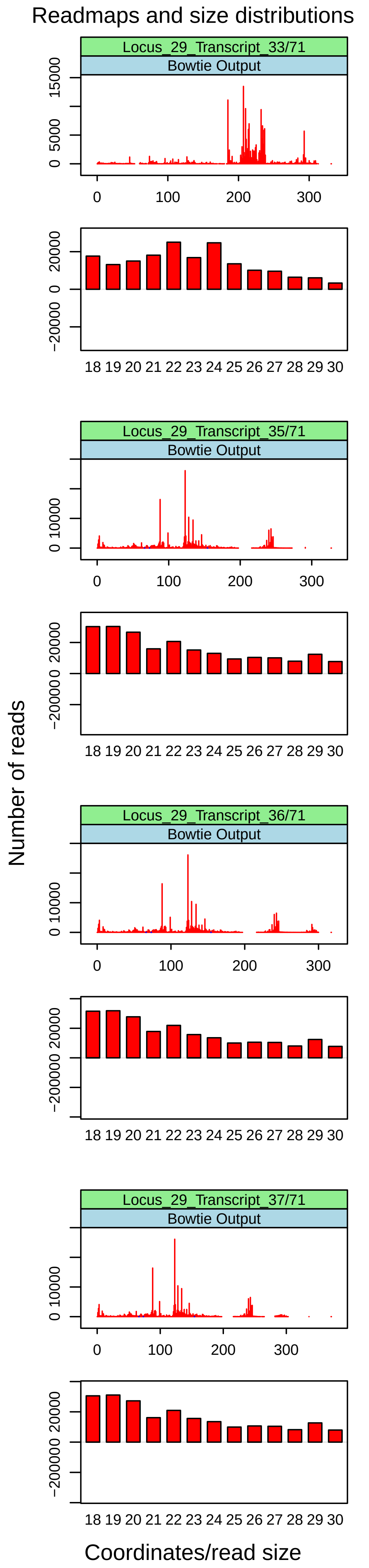
Readmaps and size distributions
Locus_29_Transcript_33/71
Bowtie Output
0 5000 15000
0 100 200 300
−20000 0 20000
18 19 20 21 22 23 24 25 26 27 28 29 30
Locus_29_Transcript_35/71
Bowtie Output
0 10000
0 100 200 300
−200000 20000
18 19 20 21 22 23 24 25 26 27 28 29 30
Locus_29_Transcript_36/71
Bowtie Output
0 10000
0 100 200 300
−200000 20000
18 19 20 21 22 23 24 25 26 27 28 29 30
Locus_29_Transcript_37/71
Bowtie Output
0 10000
0 100 200 300
−200000 20000
18 19 20 21 22 23 24 25 26 27 28 29 30
Number of reads
Coordinates/read size

Readmaps and size distributions

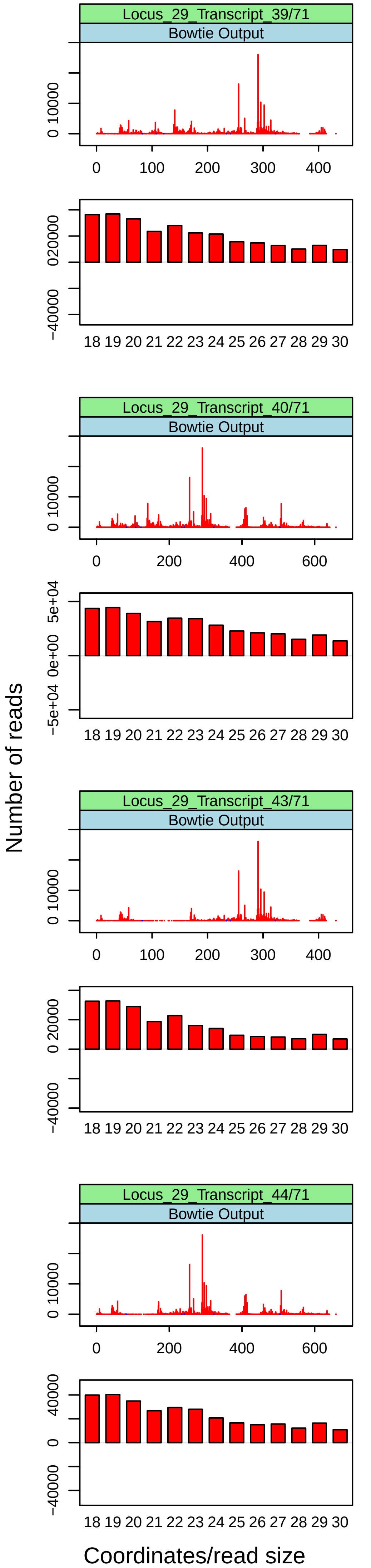

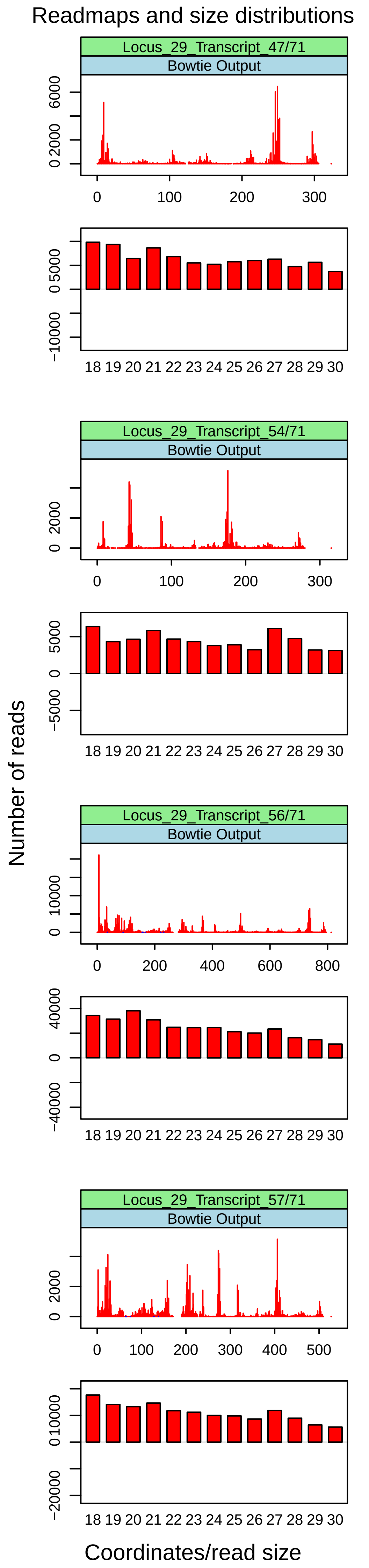

Readmaps and size distributions
Locus_29_Transcript_47/71
Bowtie Output
Locus_29_Transcript_54/71
Bowtie Output
Locus_29_Transcript_56/71
Bowtie Output
Locus_29_Transcript_57/71
Bowtie Output
Number of reads
Coordinates/read size

Readmaps and size distributions

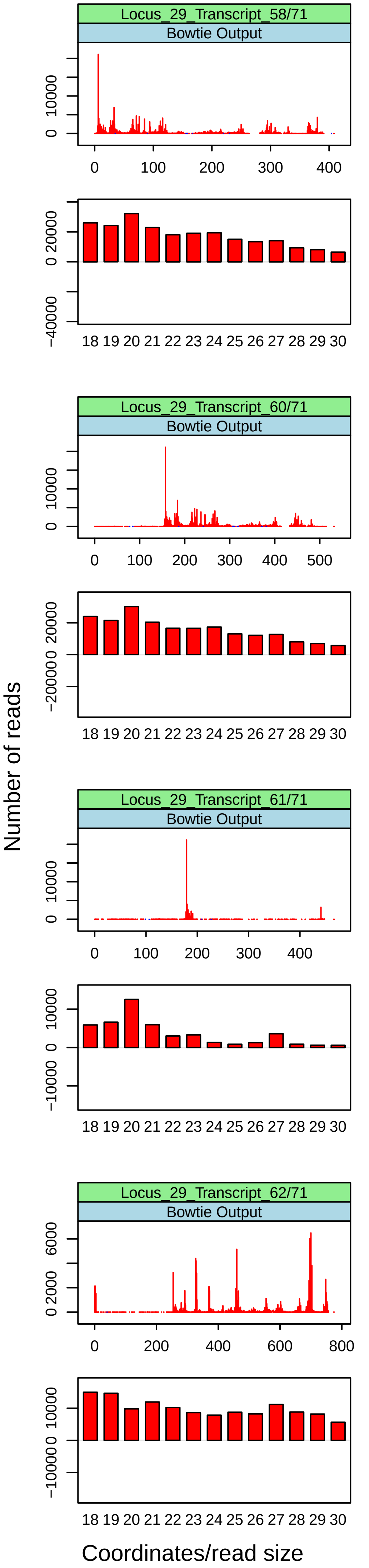

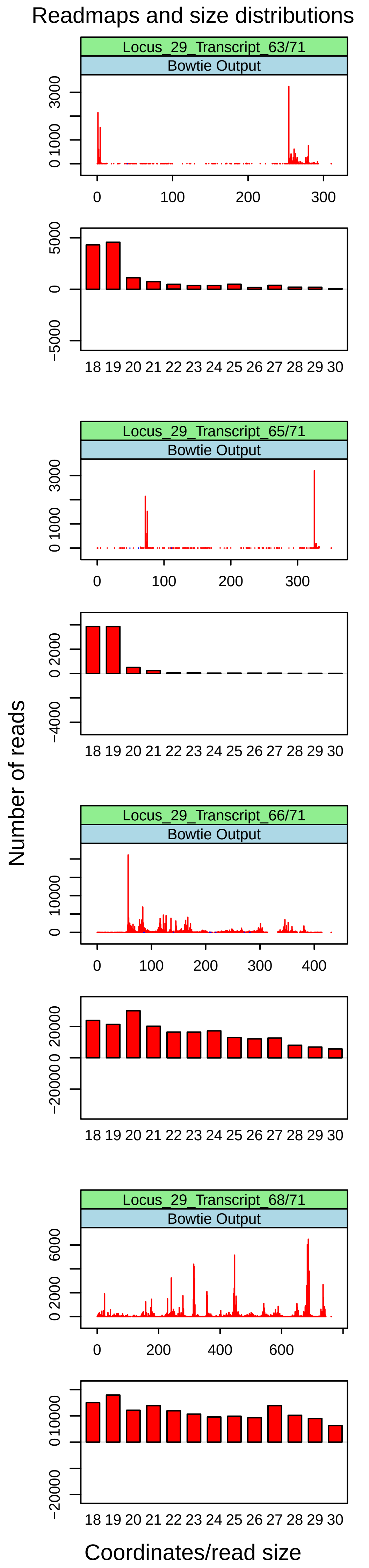
Readmaps and size distributions
Locus_29_Transcript_63/71
Bowtie Output
Locus_29_Transcript_65/71
Bowtie Output
Locus_29_Transcript_66/71
Bowtie Output
Locus_29_Transcript_68/71
Bowtie Output
Number of reads
Coordinates/read size

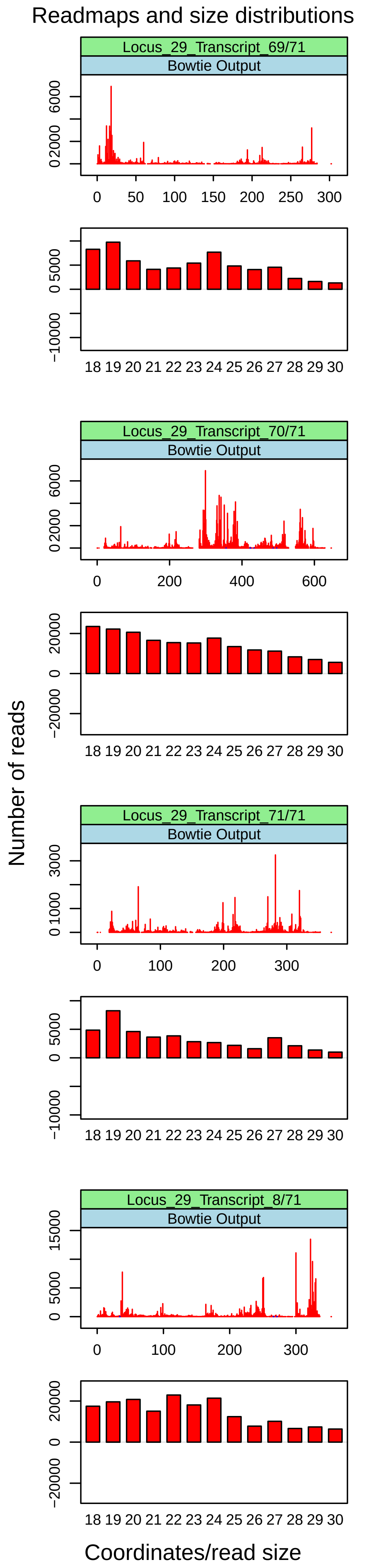
Readmaps and size distributions
Locus_29_Transcript_69/71
Bowtie Output
Locus_29_Transcript_70/71
Bowtie Output
Locus_29_Transcript_71/71
Bowtie Output
Locus_29_Transcript_8/71
Bowtie Output
Number of reads
Coordinates/read size

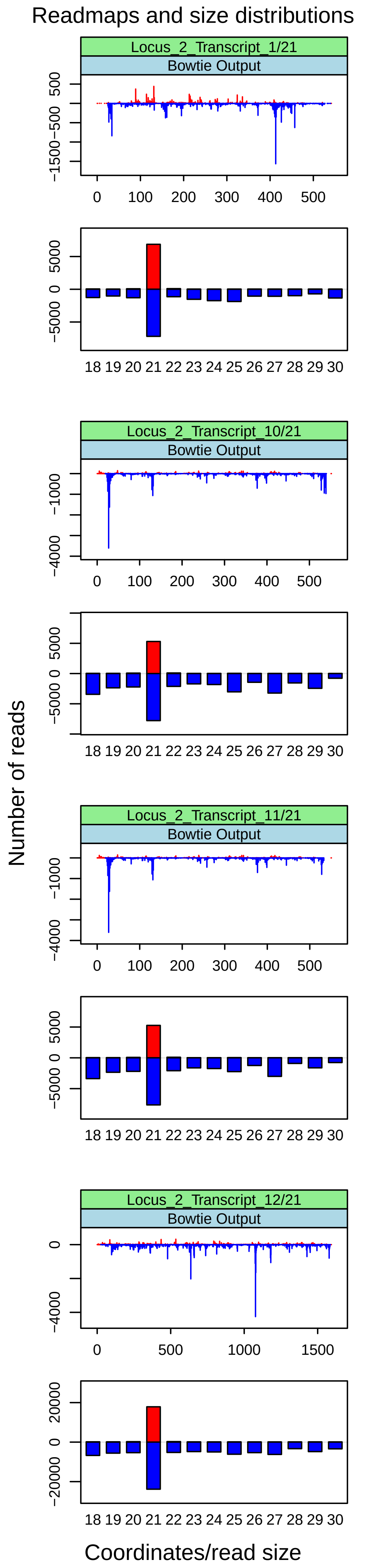

Readmaps and size distributions
Locus_2_Transcript_1/21
Bowtie Output
Locus_2_Transcript_10/21
Bowtie Output
Locus_2_Transcript_11/21
Bowtie Output
Locus_2_Transcript_12/21
Bowtie Output
Number of reads
Coordinates/read size
18 19 20 21 22 23 24 25 26 27 28 29 30

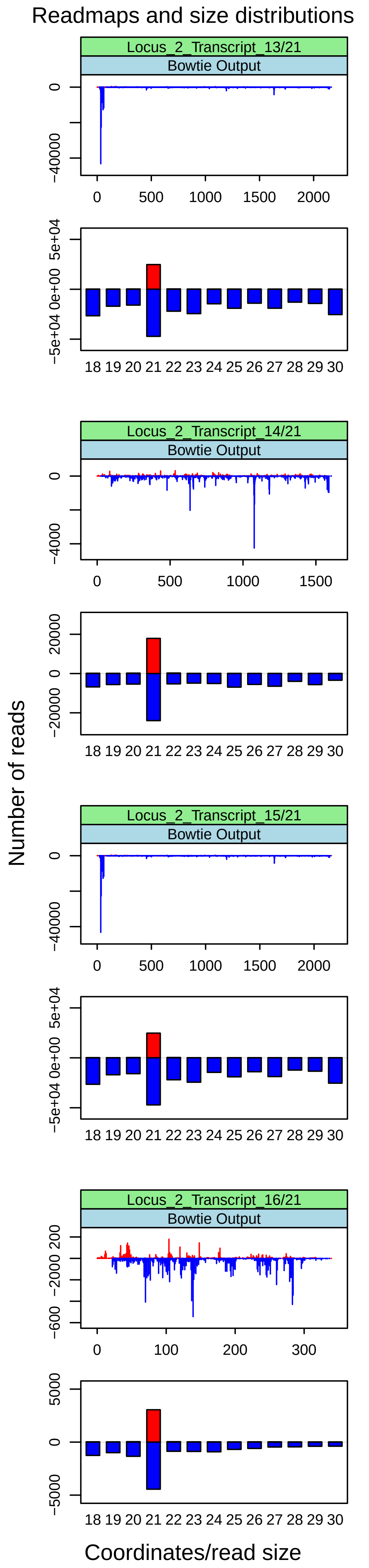

Readmaps and size distributions
Locus_2_Transcript_13/21
Bowtie Output
Locus_2_Transcript_14/21
Bowtie Output
Locus_2_Transcript_15/21
Bowtie Output
Locus_2_Transcript_16/21
Bowtie Output
Number of reads
Coordinates/read size

Readmaps and size distributions

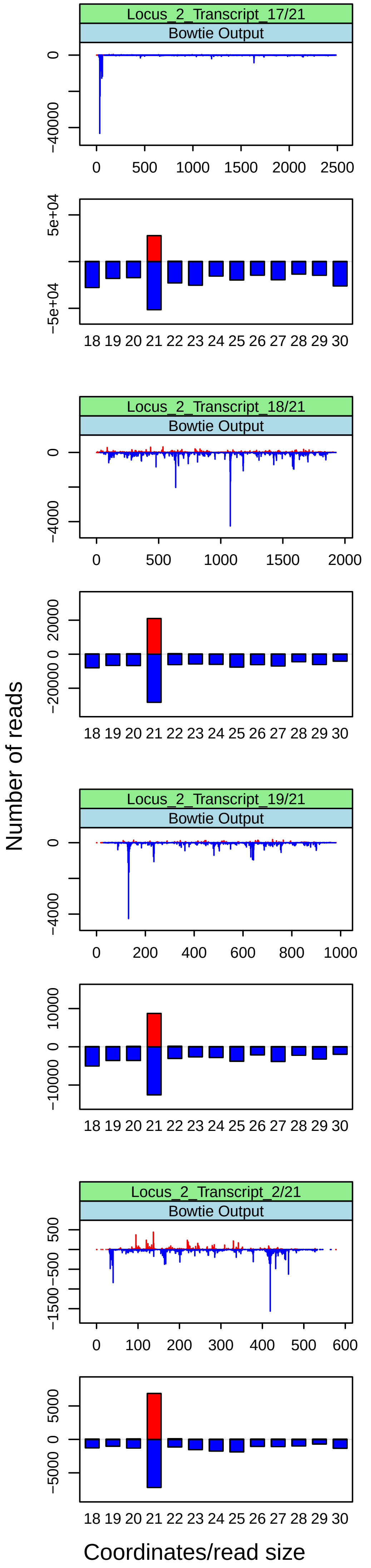

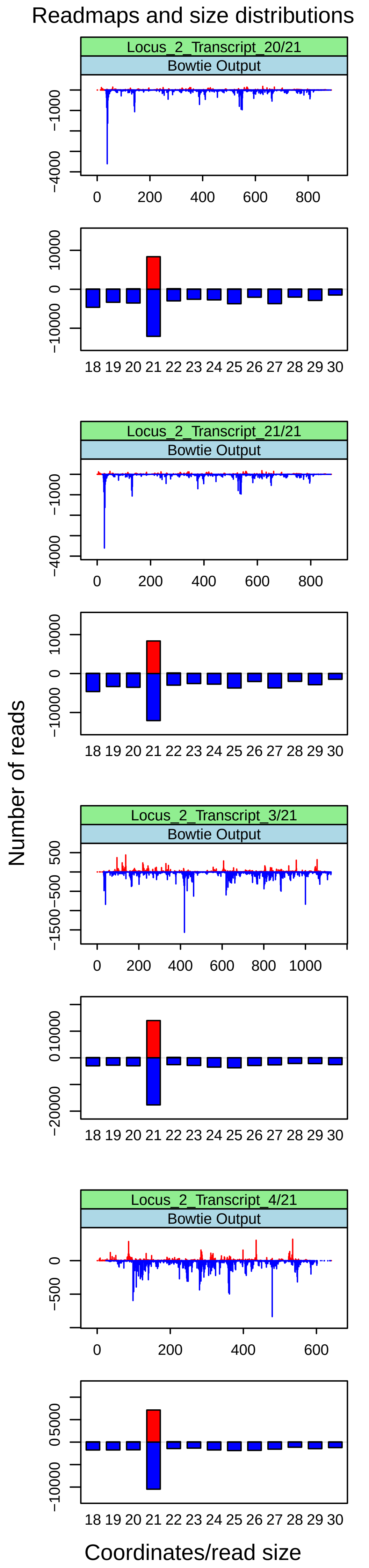
Readmaps and size distributions
Locus_2_Transcript_20/21
Bowtie Output
Locus_2_Transcript_21/21
Bowtie Output
Locus_2_Transcript_3/21
Bowtie Output
Locus_2_Transcript_4/21
Bowtie Output
Number of reads
Coordinates/read size

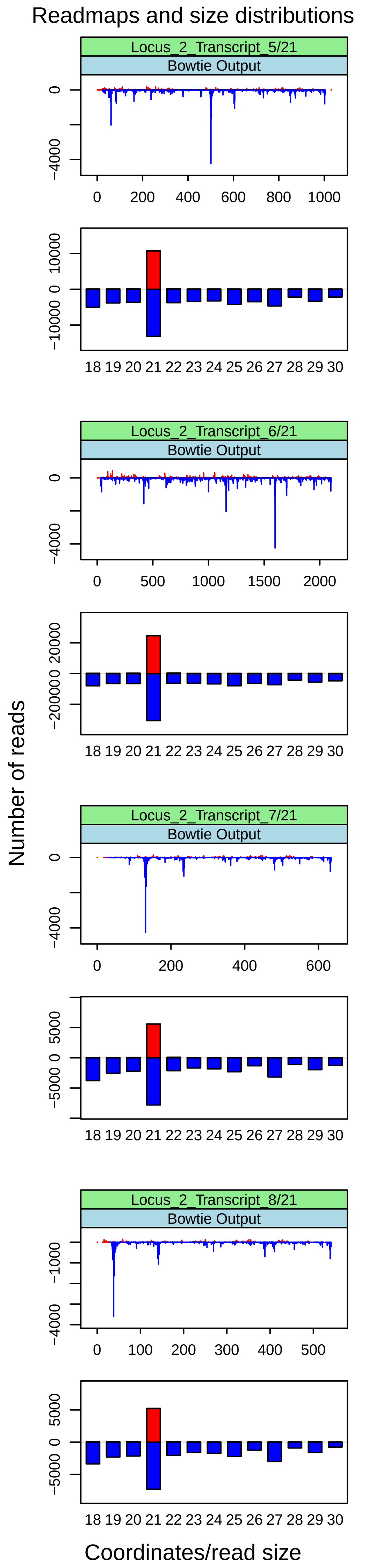

Readmaps and size distributions
Locus_2_Transcript_5/21
Bowtie Output
Locus_2_Transcript_6/21
Bowtie Output
Locus_2_Transcript_7/21
Bowtie Output
Locus_2_Transcript_8/21
Bowtie Output
Number of reads
Coordinates/read size
18 19 20 21 22 23 24 25 26 27 28 29 30

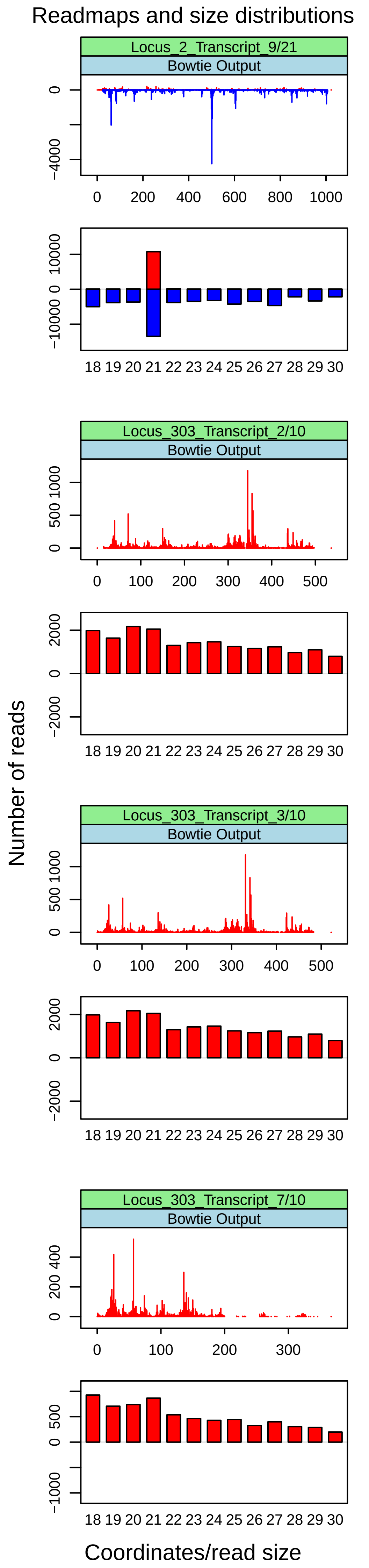

Readmaps and size distributions
Locus_2_Transcript_9/21
Bowtie Output
Locus_303_Transcript_2/10
Bowtie Output
Locus_303_Transcript_3/10
Bowtie Output
Locus_303_Transcript_7/10
Bowtie Output
Number of reads
Coordinates/read size

Readmaps and size distributions

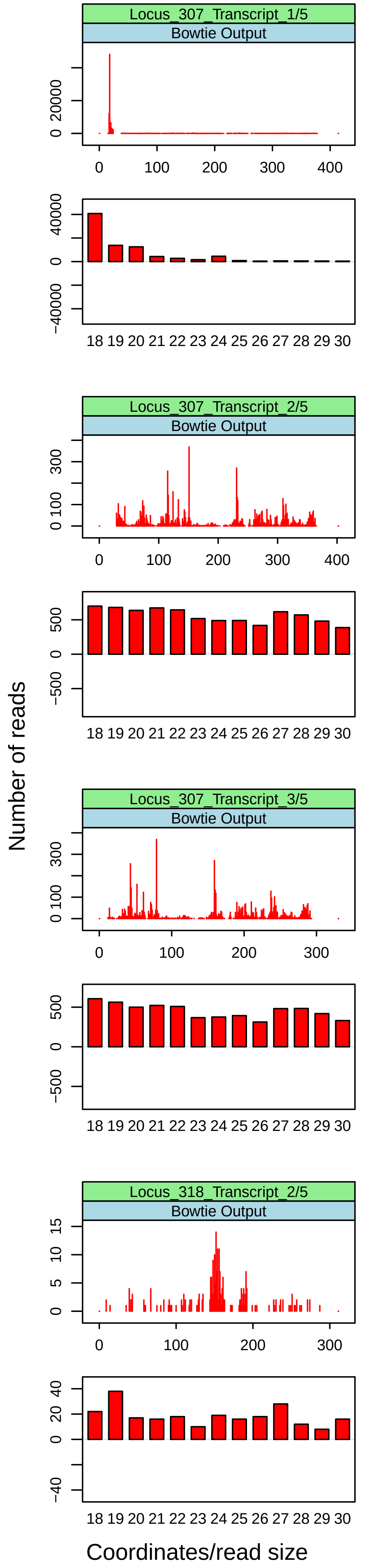

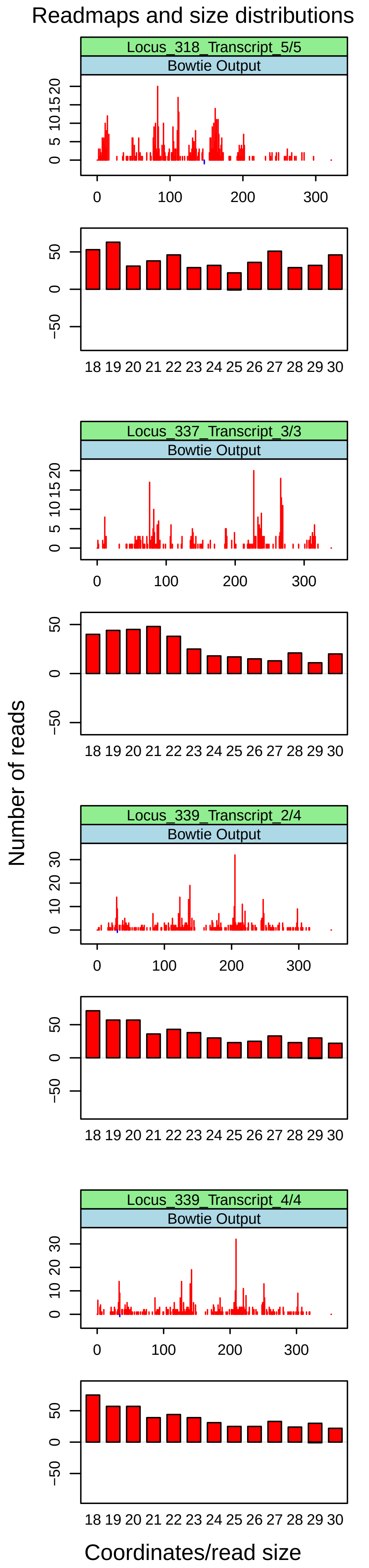

Readmaps and size distributions
Locus_318_Transcript_5/5
Bowtie Output
Locus_337_Transcript_3/3
Bowtie Output
Locus_339_Transcript_2/4
Bowtie Output
Locus_339_Transcript_4/4
Bowtie Output
Number of reads
Coordinates/read size

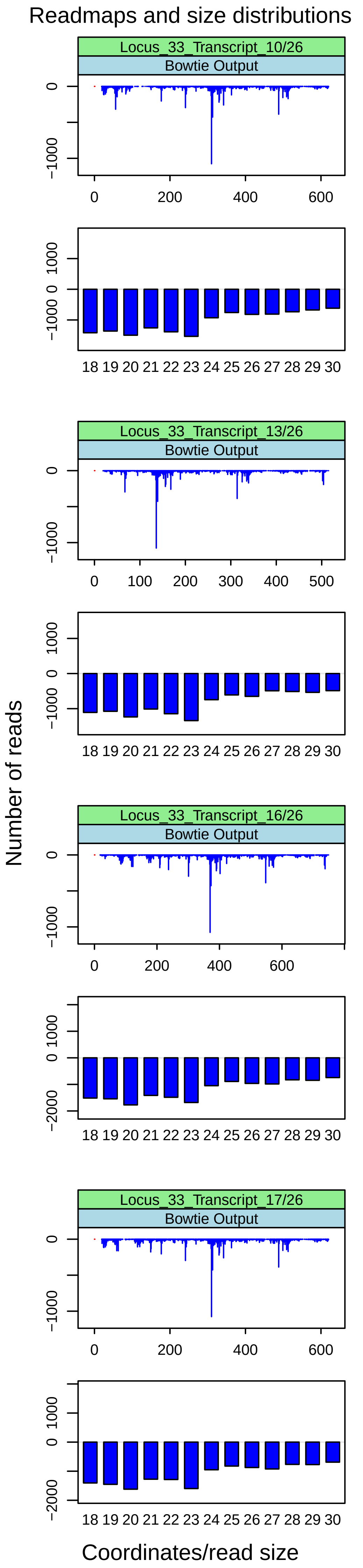
Readmaps and size distributions
Locus_33_Transcript_10/26
Bowtie Output
Locus_33_Transcript_13/26
Bowtie Output
Locus_33_Transcript_16/26
Bowtie Output
Locus_33_Transcript_17/26
Bowtie Output
Number of reads
18 19 20 21 22 23 24 25 26 27 28 29 30
Coordinates/read size

Readmaps and size distributions

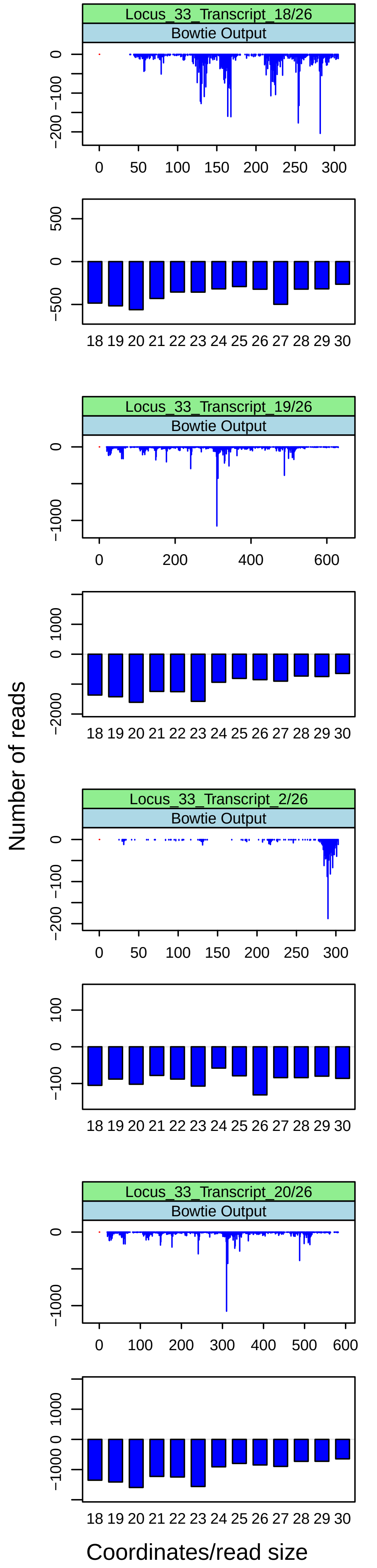

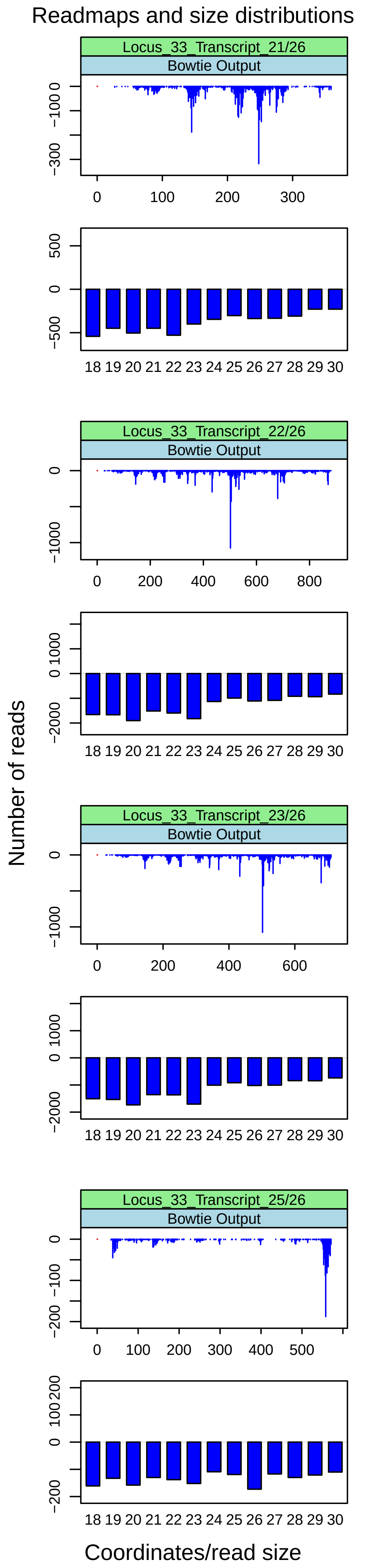

Readmaps and size distributions
Locus_33_Transcript_21/26
Bowtie Output
Locus_33_Transcript_22/26
Bowtie Output
Locus_33_Transcript_23/26
Bowtie Output
Locus_33_Transcript_25/26
Bowtie Output
Number of reads
Coordinates/read size
18 19 20 21 22 23 24 25 26 27 28 29 30

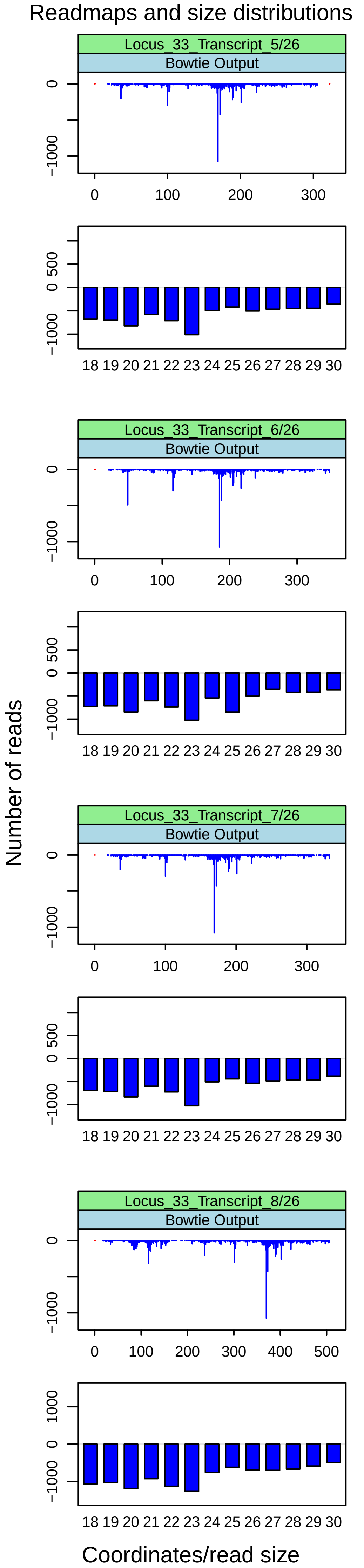
Readmaps and size distributions
Locus_33_Transcript_5/26
Bowtie Output
0
−1000
0 100 200 300
0 500
−1000
18 19 20 21 22 23 24 25 26 27 28 29 30
Locus_33_Transcript_6/26
Bowtie Output
0
−1000
0 100 200 300
0 500
−1000
18 19 20 21 22 23 24 25 26 27 28 29 30
Locus_33_Transcript_7/26
Bowtie Output
0
−1000
0 100 200 300
0 500
−1000
18 19 20 21 22 23 24 25 26 27 28 29 30
Locus_33_Transcript_8/26
Bowtie Output
0
−1000
0 100 200 300 400 500
1000
0
−1000
18 19 20 21 22 23 24 25 26 27 28 29 30
Number of reads
Coordinates/read size

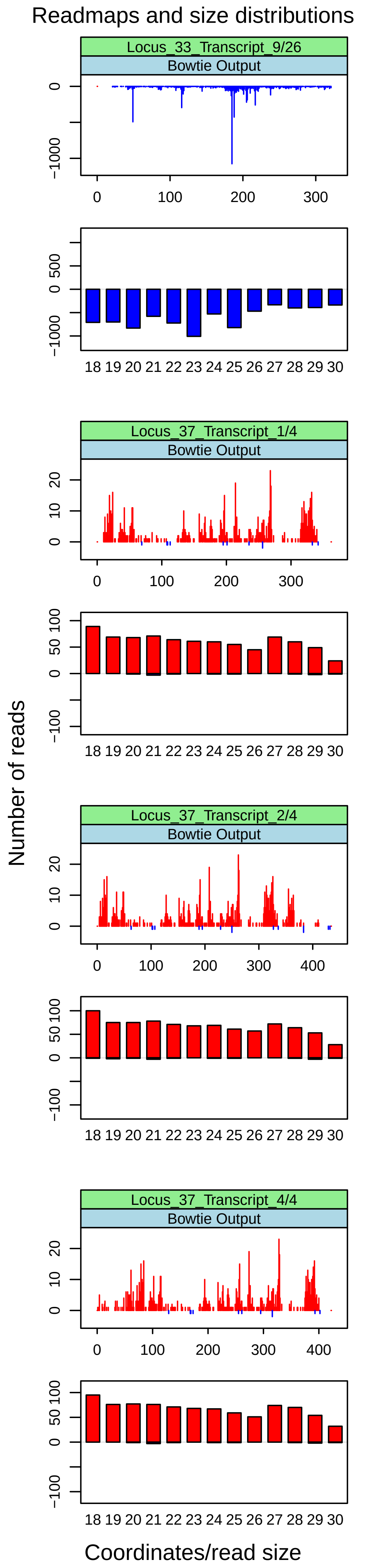
Readmaps and size distributions
Locus_33_Transcript_9/26
Bowtie Output
Locus_37_Transcript_1/4
Bowtie Output
Locus_37_Transcript_2/4
Bowtie Output
Locus_37_Transcript_4/4
Bowtie Output
Number of reads
Coordinates/read size
18 19 20 21 22 23 24 25 26 27 28 29 30

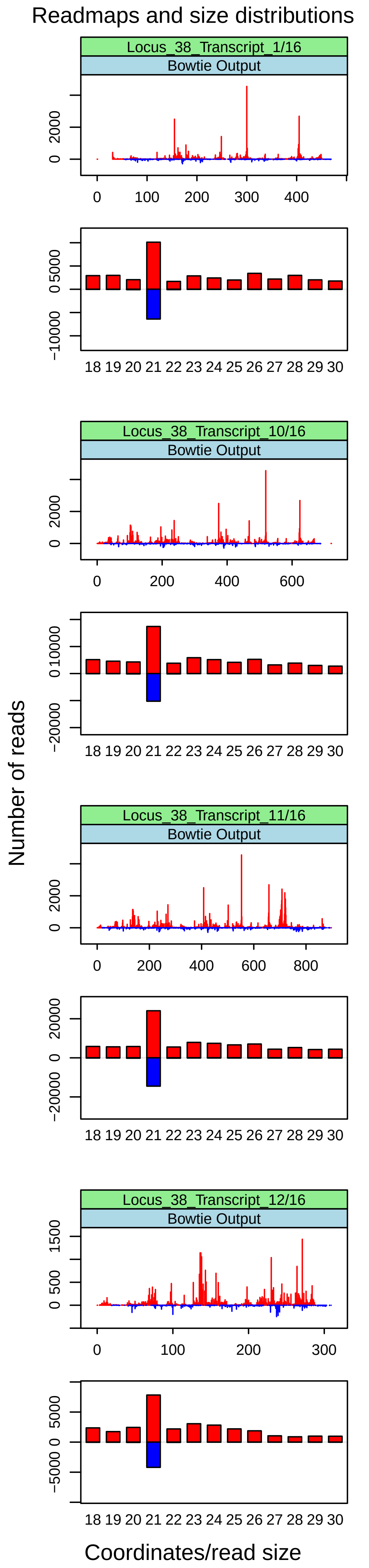
Readmaps and size distributions
Locus_38_Transcript_1/16
Bowtie Output
Locus_38_Transcript_10/16
Bowtie Output
Locus_38_Transcript_11/16
Bowtie Output
Locus_38_Transcript_12/16
Bowtie Output
18 19 20 21 22 23 24 25 26 27 28 29 30
Number of reads
Coordinates/read size

Readmaps and size distributions

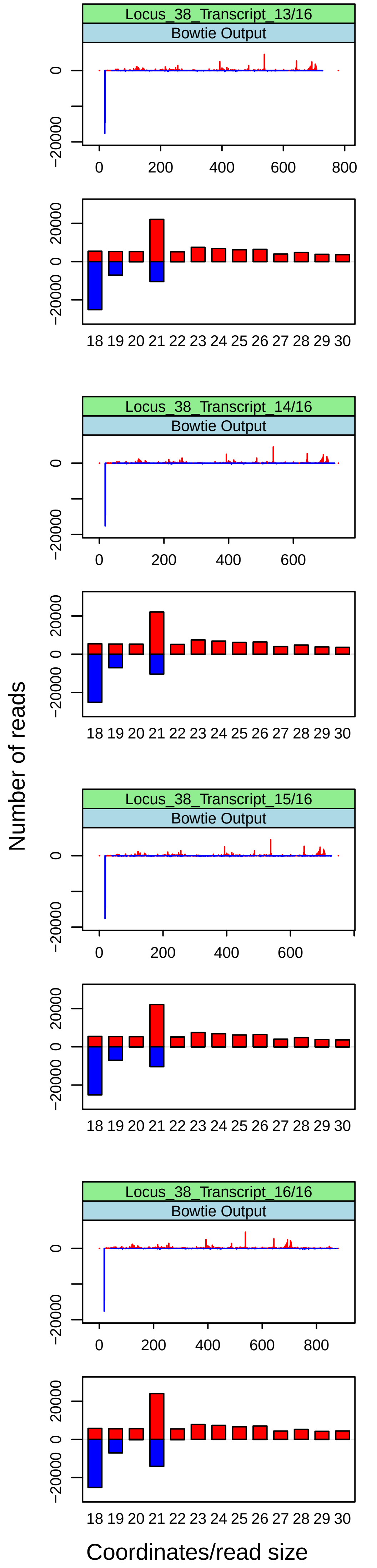

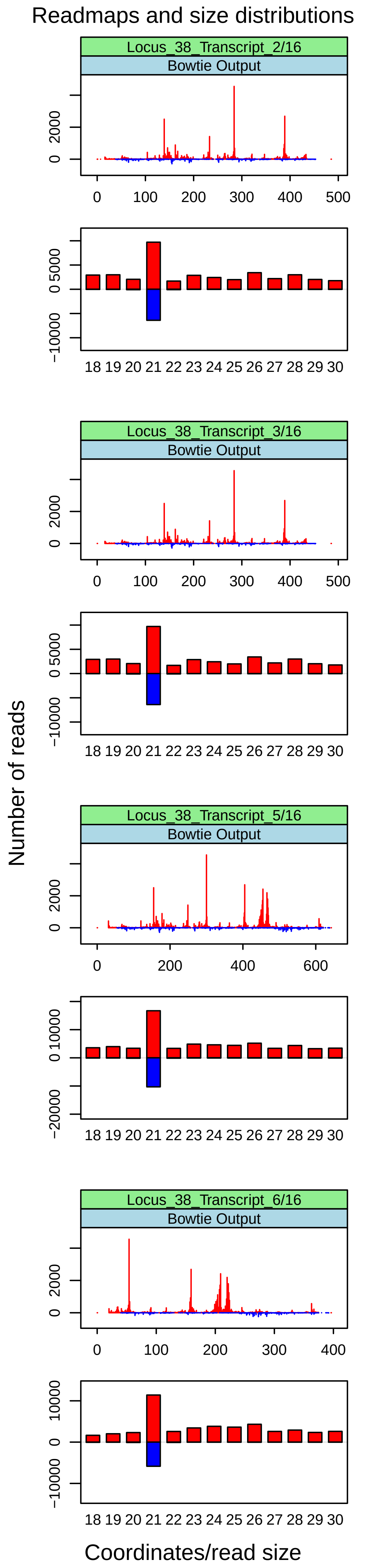

Readmaps and size distributions
Locus_38_Transcript_2/16
Bowtie Output
Locus_38_Transcript_3/16
Bowtie Output
Locus_38_Transcript_5/16
Bowtie Output
Locus_38_Transcript_6/16
Bowtie Output
Number of reads
Coordinates/read size

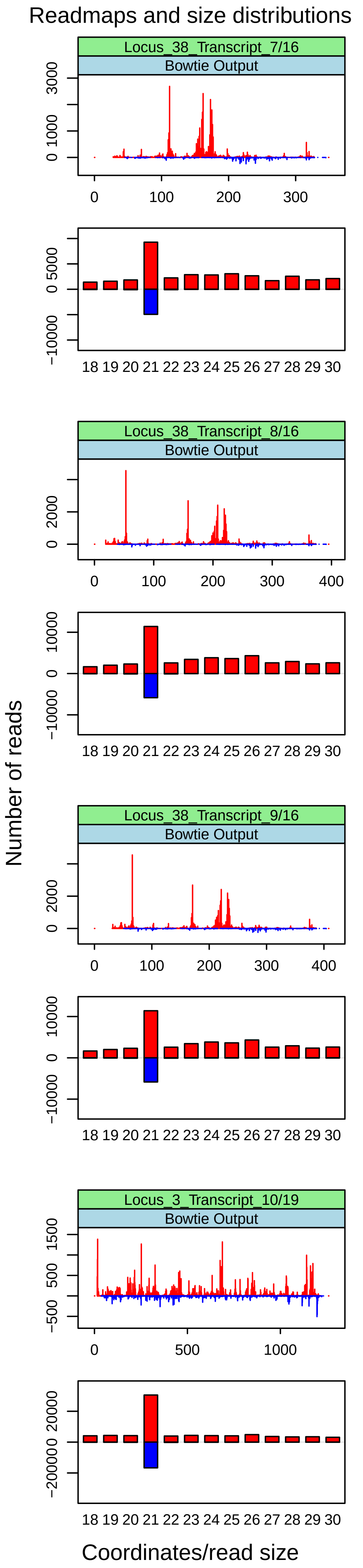
Readmaps and size distributions
Locus_38_Transcript_7/16
Bowtie Output
Locus_38_Transcript_8/16
Bowtie Output
Locus_38_Transcript_9/16
Bowtie Output
Locus_3_Transcript_10/19
Bowtie Output
Number of reads
Coordinates/read size

Readmaps and size distributions

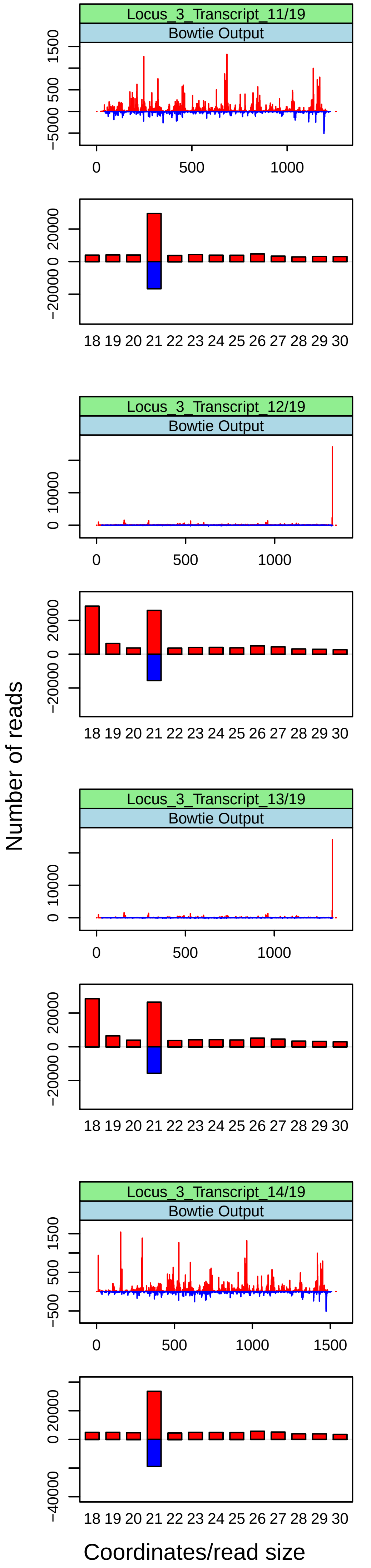

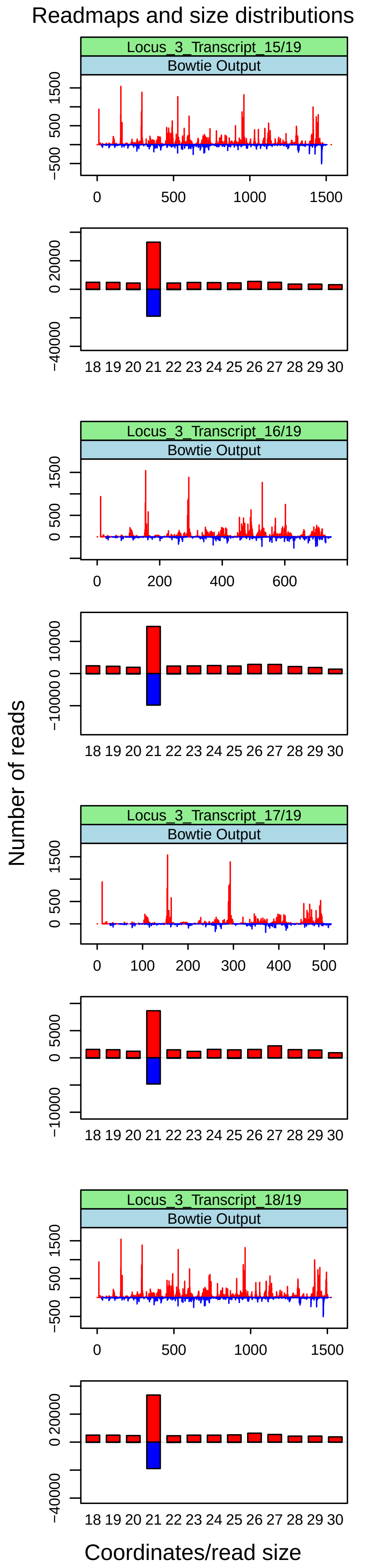
Readmaps and size distributions
Locus_3_Transcript_15/19
Bowtie Output
Locus_3_Transcript_16/19
Bowtie Output
Locus_3_Transcript_17/19
Bowtie Output
Locus_3_Transcript_18/19
Bowtie Output
18 19 20 21 22 23 24 25 26 27 28 29 30
Number of reads
Coordinates/read size

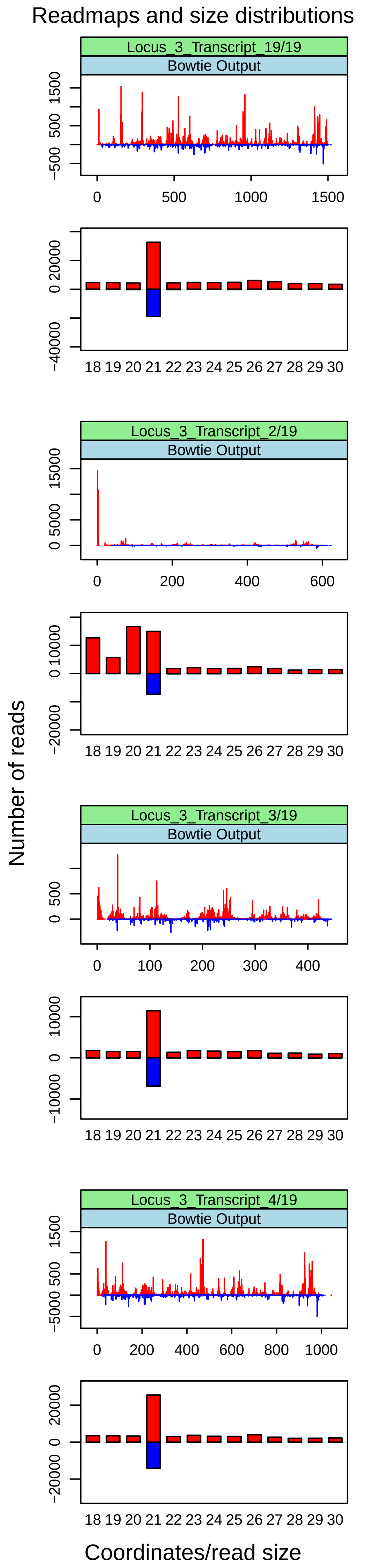

Readmaps and size distributions
Locus_3_Transcript_19/19
Bowtie Output
Locus_3_Transcript_2/19
Bowtie Output
Locus_3_Transcript_3/19
Bowtie Output
Locus_3_Transcript_4/19
Bowtie Output
18 19 20 21 22 23 24 25 26 27 28 29 30
Number of reads
Coordinates/read size

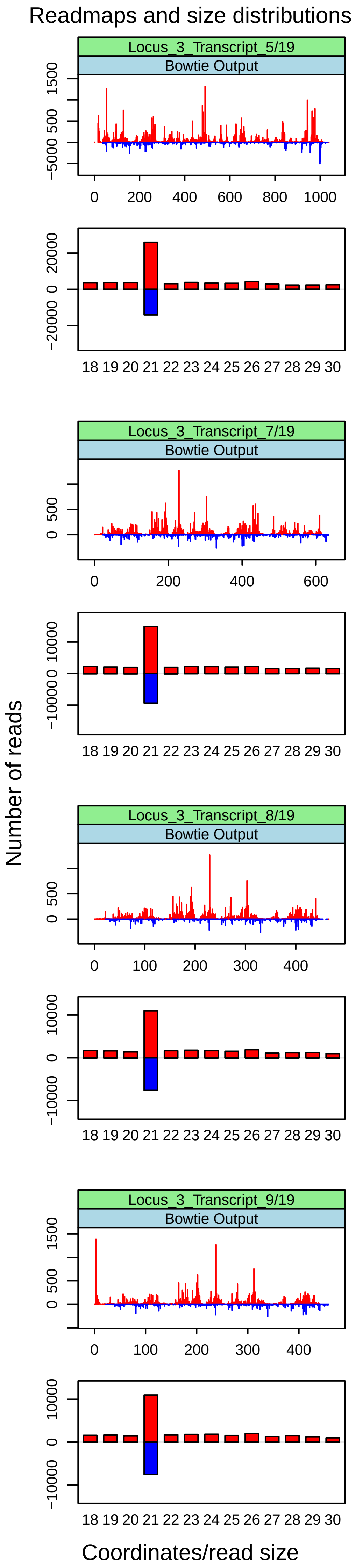

Readmaps and size distributions
Locus_3_Transcript_5/19
Bowtie Output
Locus_3_Transcript_7/19
Bowtie Output
Locus_3_Transcript_8/19
Bowtie Output
Locus_3_Transcript_9/19
Bowtie Output
Number of reads
Coordinates/read size

Readmaps and size distributions

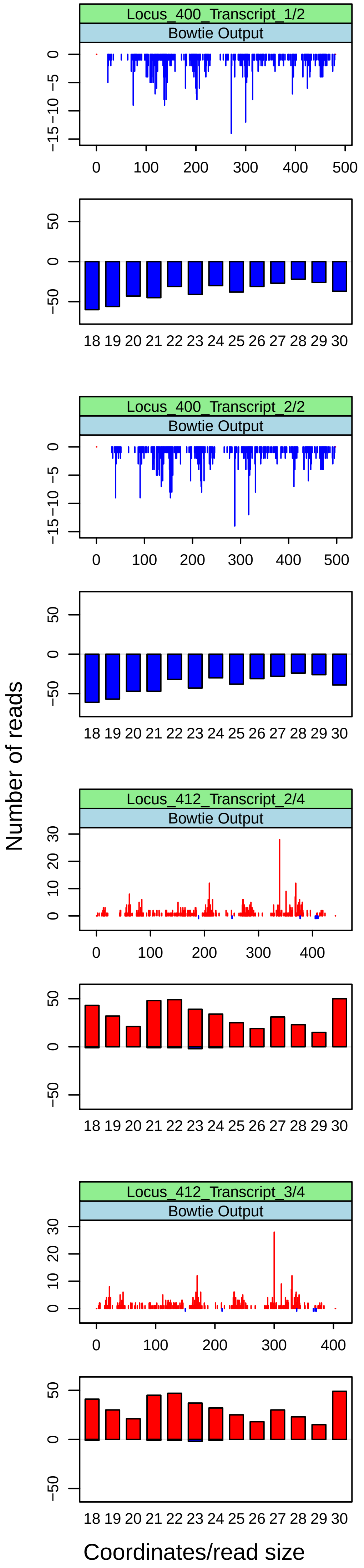

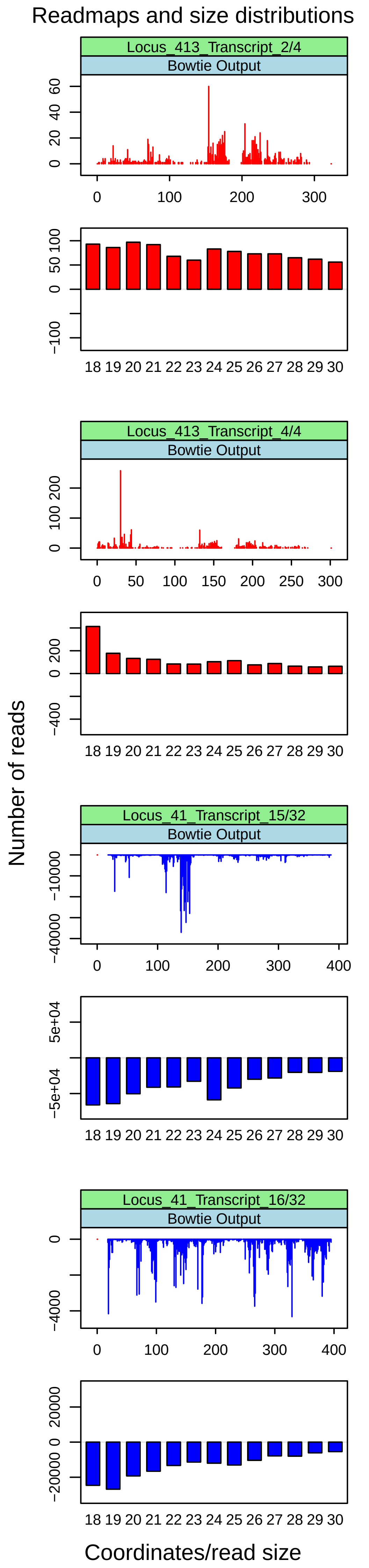
Readmaps and size distributions
Locus_413_Transcript_2/4
Bowtie Output
Locus_413_Transcript_4/4
Bowtie Output
Locus_41_Transcript_15/32
Bowtie Output
Locus_41_Transcript_16/32
Bowtie Output
Number of reads
Coordinates/read size

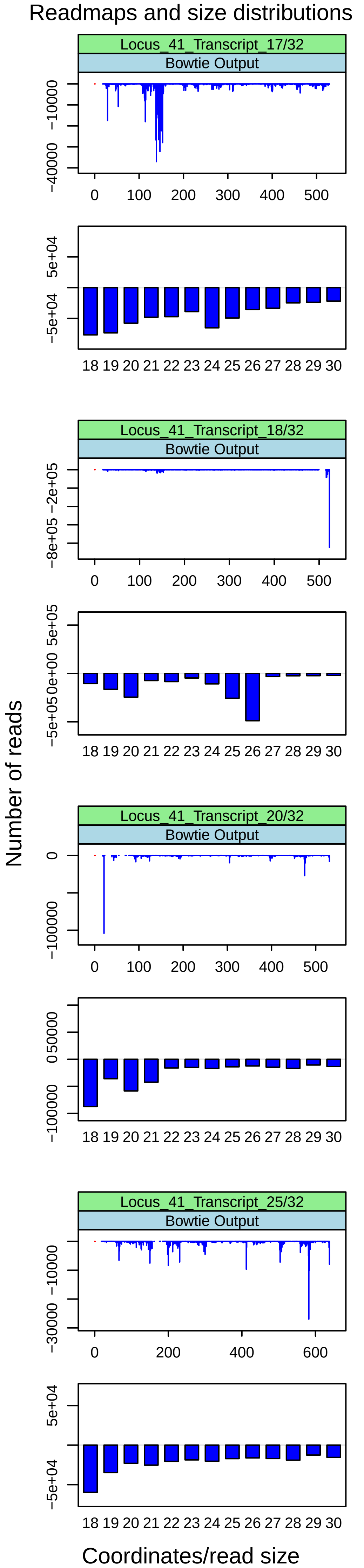

Readmaps and size distributions
Locus_41_Transcript_17/32
Bowtie Output
Locus_41_Transcript_18/32
Bowtie Output
Locus_41_Transcript_20/32
Bowtie Output
Locus_41_Transcript_25/32
Bowtie Output
Number of reads
Coordinates/read size

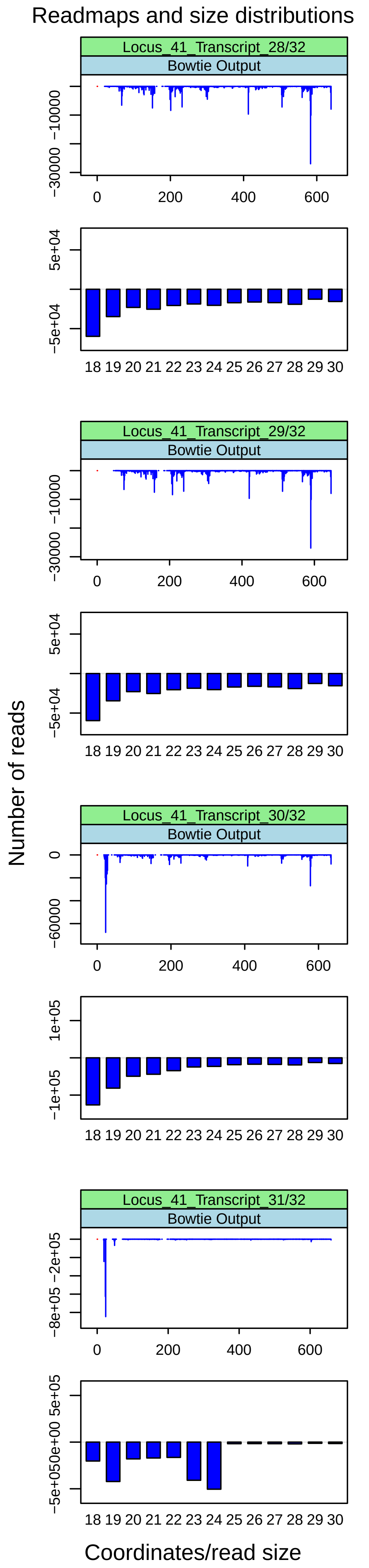

Readmaps and size distributions
Locus_41_Transcript_28/32
Bowtie Output
Locus_41_Transcript_29/32
Bowtie Output
Locus_41_Transcript_30/32
Bowtie Output
Locus_41_Transcript_31/32
Bowtie Output
Number of reads
Coordinates/read size

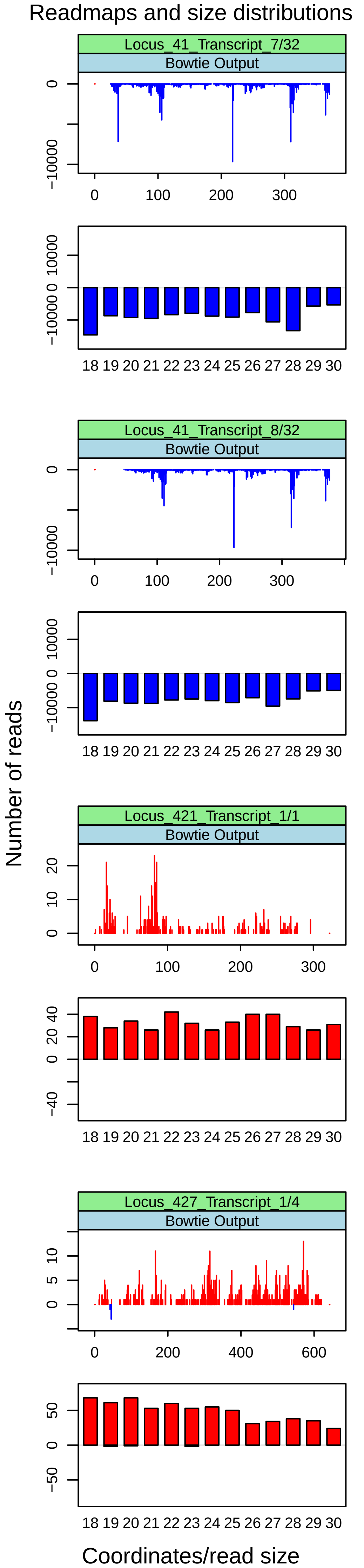
Readmaps and size distributions
Locus_41_Transcript_7/32
Bowtie Output
Locus_41_Transcript_8/32
Bowtie Output
Locus_421_Transcript_1/1
Bowtie Output
Locus_427_Transcript_1/4
Bowtie Output
Number of reads
Coordinates/read size
18 19 20 21 22 23 24 25 26 27 28 29 30

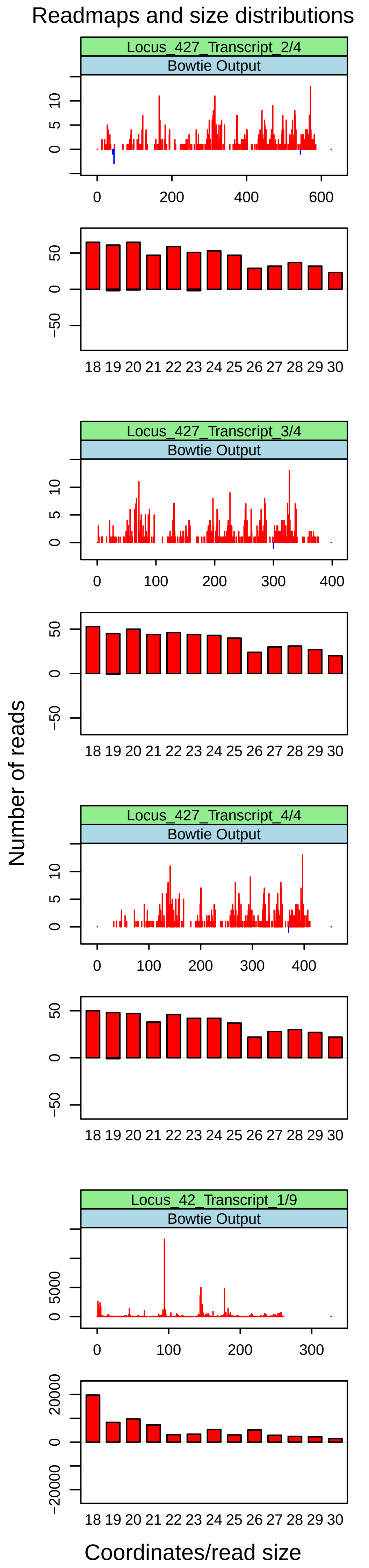
Readmaps and size distributions
Locus_427_Transcript_2/4
Bowtie Output
Locus_427_Transcript_3/4
Bowtie Output
Locus_427_Transcript_4/4
Bowtie Output
Locus_42_Transcript_1/9
Bowtie Output
Number of reads
Coordinates/read size
18 19 20 21 22 23 24 25 26 27 28 29 30

Readmaps and size distributions

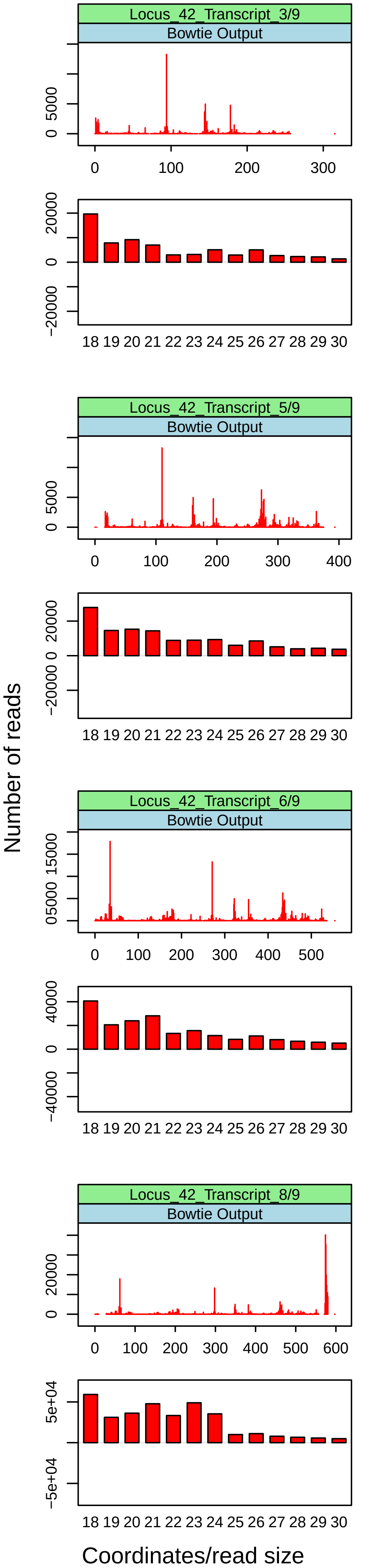

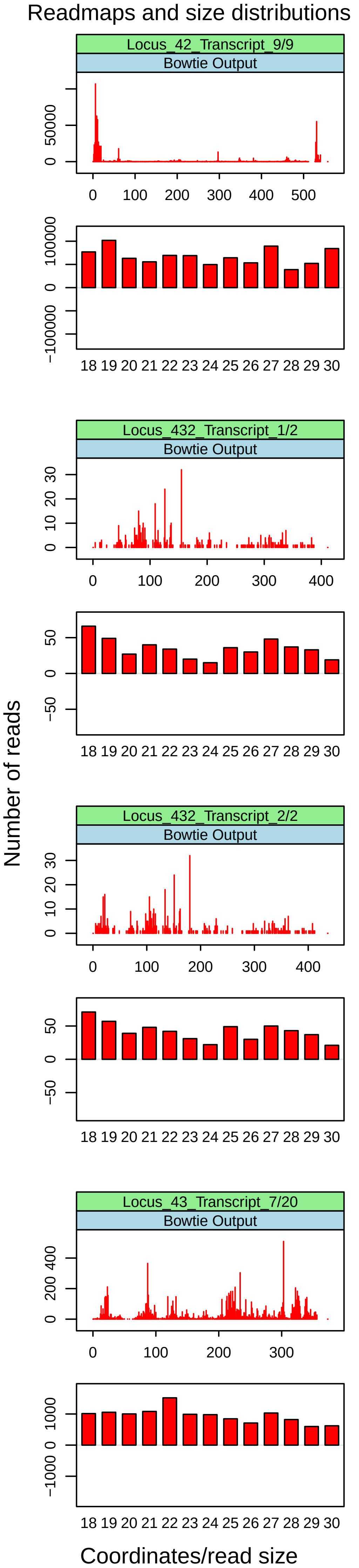
Readmaps and size distributions
Locus_42_Transcript_9/9
Bowtie Output
Locus_432_Transcript_1/2
Bowtie Output
Locus_432_Transcript_2/2
Bowtie Output
Locus_43_Transcript_7/20
Bowtie Output
Number of reads
Coordinates/read size

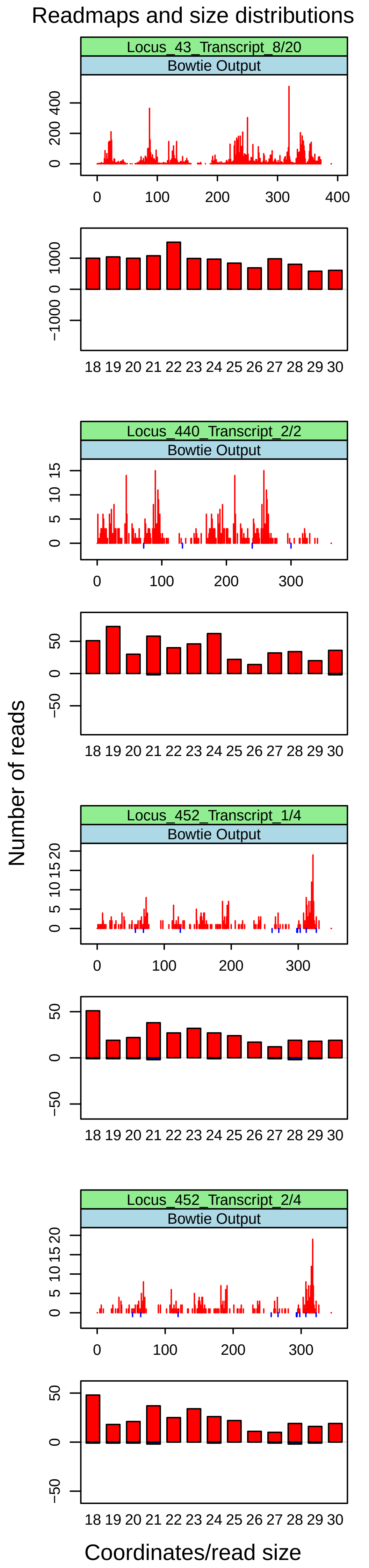
Readmaps and size distributions
Locus_43_Transcript_8/20
Bowtie Output
Locus_440_Transcript_2/2
Bowtie Output
Locus_452_Transcript_1/4
Bowtie Output
Locus_452_Transcript_2/4
Bowtie Output
18 19 20 21 22 23 24 25 26 27 28 29 30
Number of reads
Coordinates/read size

Readmaps and size distributions

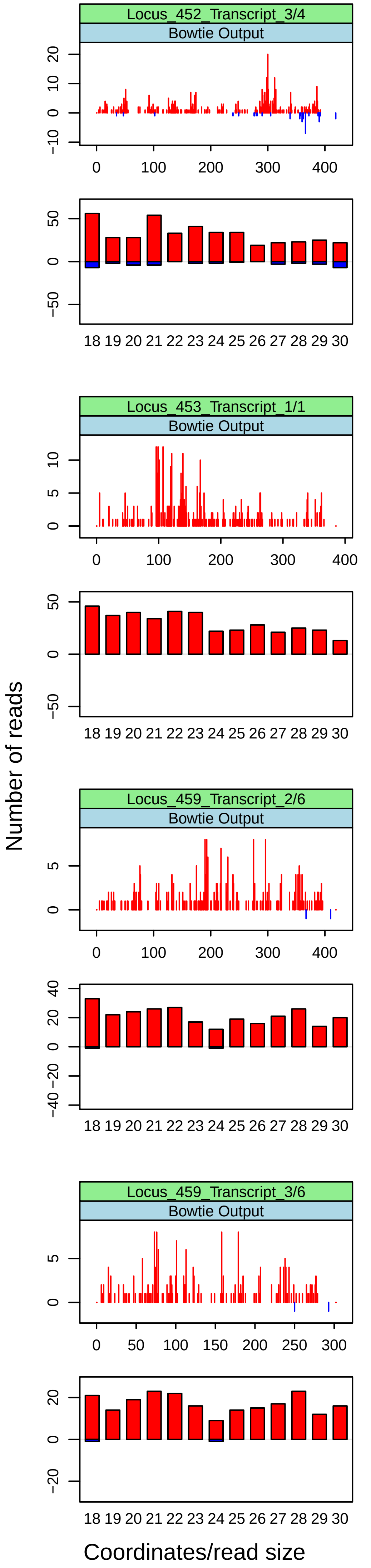

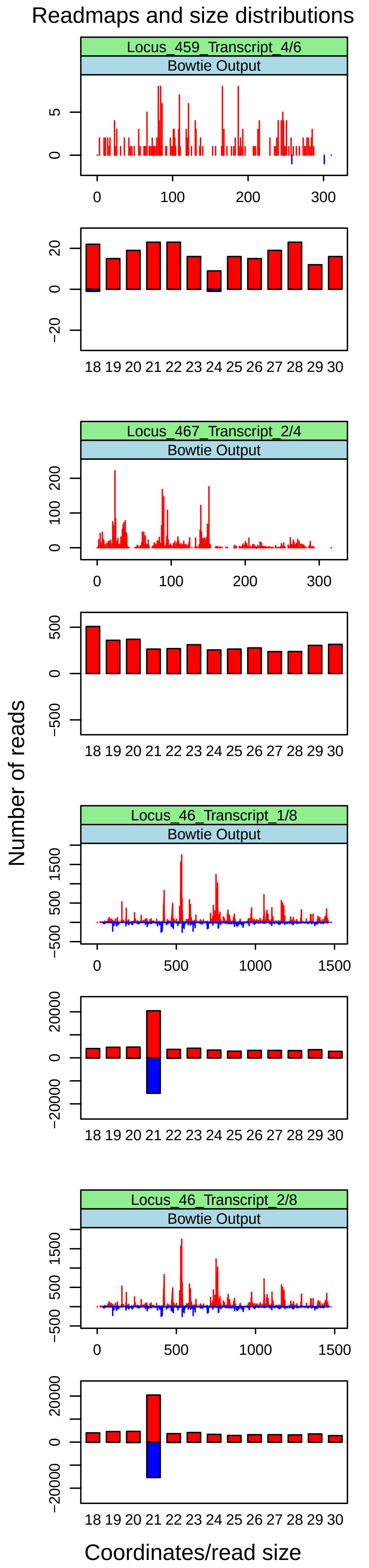

Readmaps and size distributions
Locus_459_Transcript_4/6
Bowtie Output
Locus_467_Transcript_2/4
Bowtie Output
Locus_46_Transcript_1/8
Bowtie Output
Locus_46_Transcript_2/8
Bowtie Output
Number of reads
Coordinates/read size

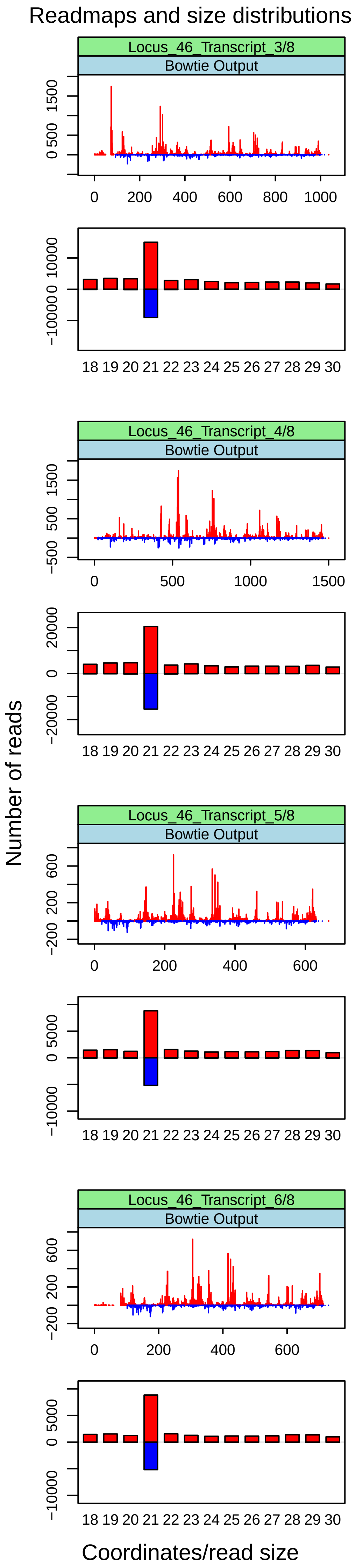
Readmaps and size distributions
Locus_46_Transcript_3/8
Bowtie Output
Locus_46_Transcript_4/8
Bowtie Output
Locus_46_Transcript_5/8
Bowtie Output
Locus_46_Transcript_6/8
Bowtie Output
18 19 20 21 22 23 24 25 26 27 28 29 30
Number of reads
Coordinates/read size

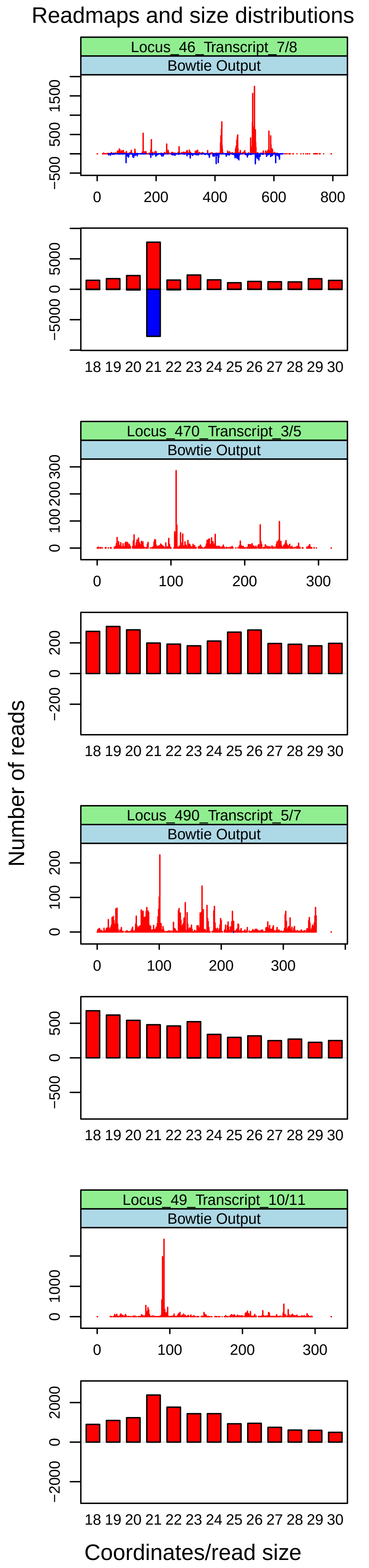

Readmaps and size distributions
Locus_46_Transcript_7/8
Bowtie Output
Locus_470_Transcript_3/5
Bowtie Output
Locus_490_Transcript_5/7
Bowtie Output
Locus_49_Transcript_10/11
Bowtie Output
Number of reads
Coordinates/read size

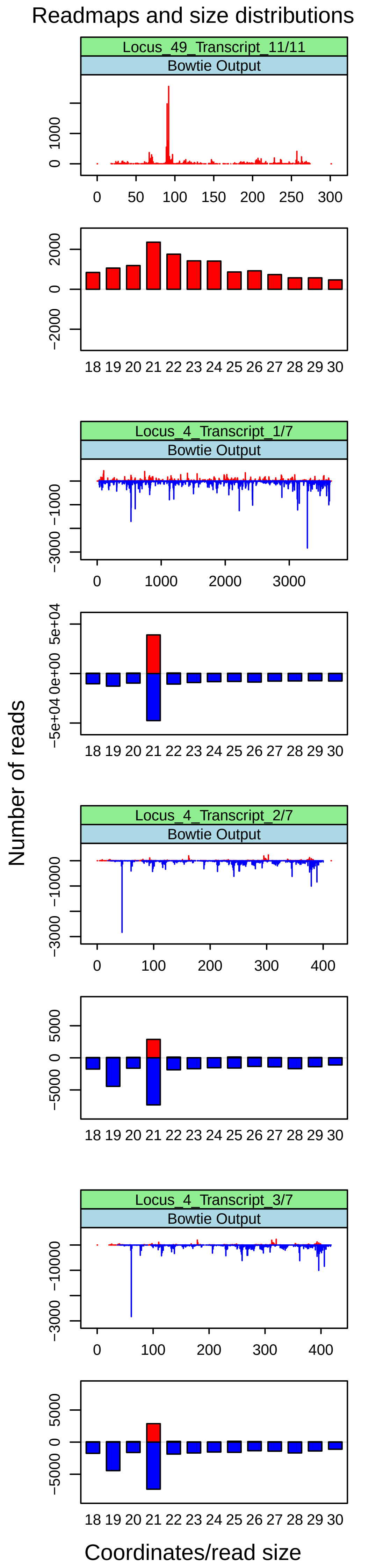

Readmaps and size distributions
Locus_49_Transcript_11/11
Bowtie Output
Locus_4_Transcript_1/7
Bowtie Output
Locus_4_Transcript_2/7
Bowtie Output
Locus_4_Transcript_3/7
Bowtie Output
Number of reads
Coordinates/read size

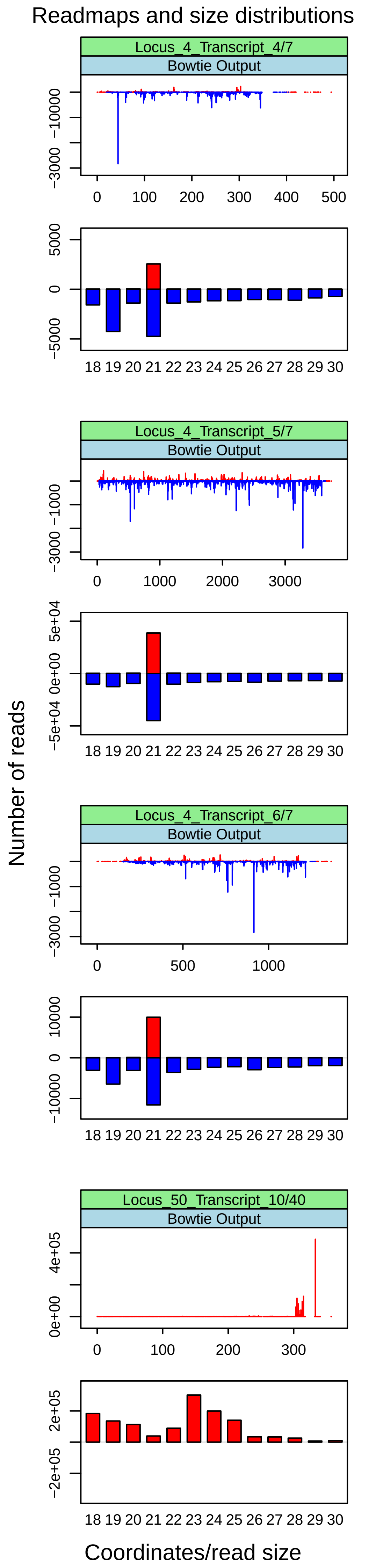
Readmaps and size distributions
Locus_4_Transcript_4/7
Bowtie Output
Locus_4_Transcript_5/7
Bowtie Output
Locus_4_Transcript_6/7
Bowtie Output
Locus_50_Transcript_10/40
Bowtie Output
Number of reads
Coordinates/read size
18 19 20 21 22 23 24 25 26 27 28 29 30

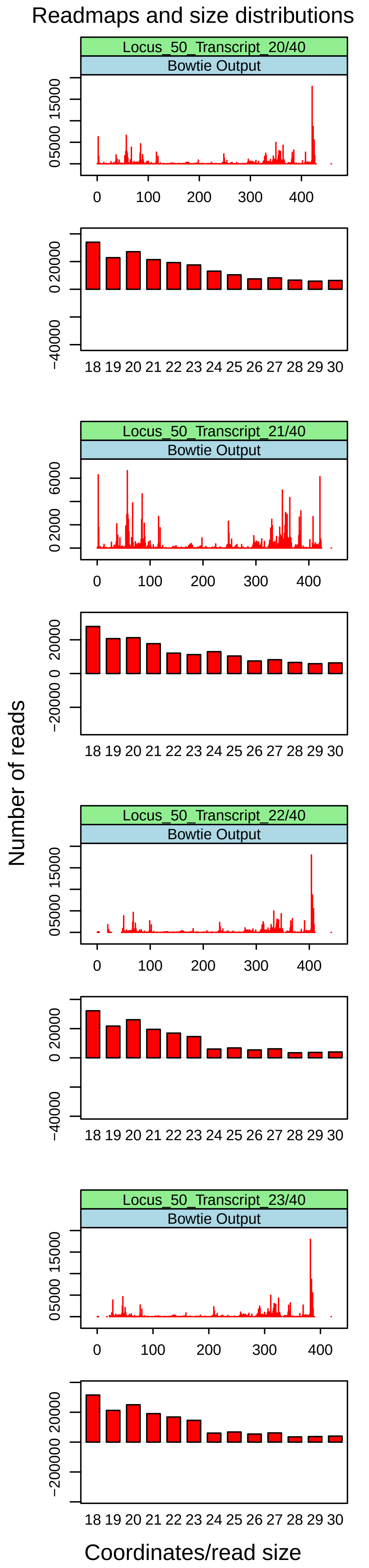

Readmaps and size distributions
Locus_50_Transcript_20/40
Bowtie Output
05000 15000
0 100 200 300 400
−40000 0 20000
18 19 20 21 22 23 24 25 26 27 28 29 30
Locus_50_Transcript_21/40
Bowtie Output
0 2000 6000
0 100 200 300 400
−20000 0 20000
18 19 20 21 22 23 24 25 26 27 28 29 30
Locus_50_Transcript_22/40
Bowtie Output
05000 15000
0 100 200 300 400
−40000 0 20000
18 19 20 21 22 23 24 25 26 27 28 29 30
Locus_50_Transcript_23/40
Bowtie Output
05000 15000
0 100 200 300 400
−200000 20000
18 19 20 21 22 23 24 25 26 27 28 29 30
Number of reads
Coordinates/read size

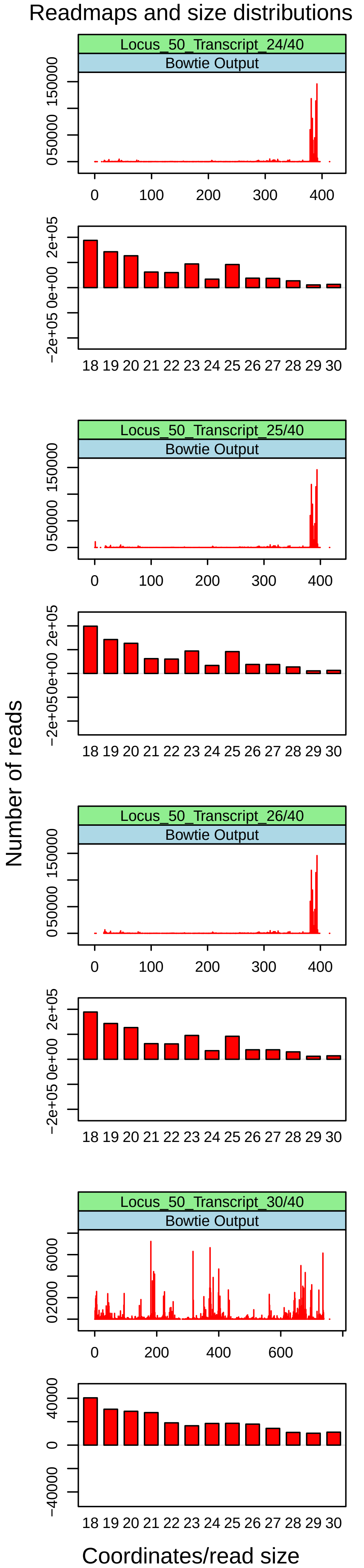
Readmaps and size distributions
Locus_50_Transcript_24/40
Bowtie Output
Locus_50_Transcript_25/40
Bowtie Output
Locus_50_Transcript_26/40
Bowtie Output
Locus_50_Transcript_30/40
Bowtie Output
Number of reads
Coordinates/read size
18 19 20 21 22 23 24 25 26 27 28 29 30

Readmaps and size distributions

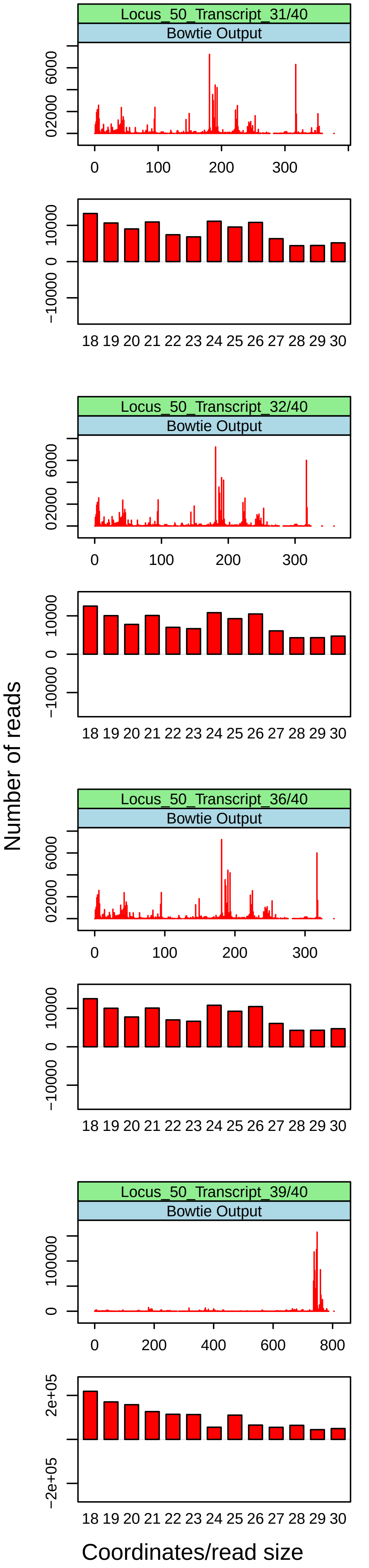

Readmaps and size distributions

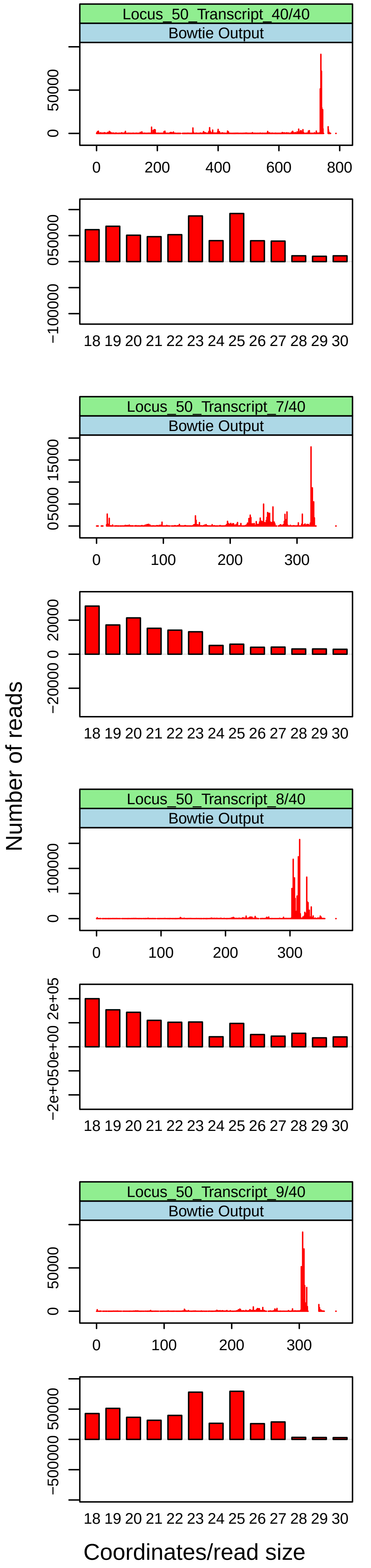

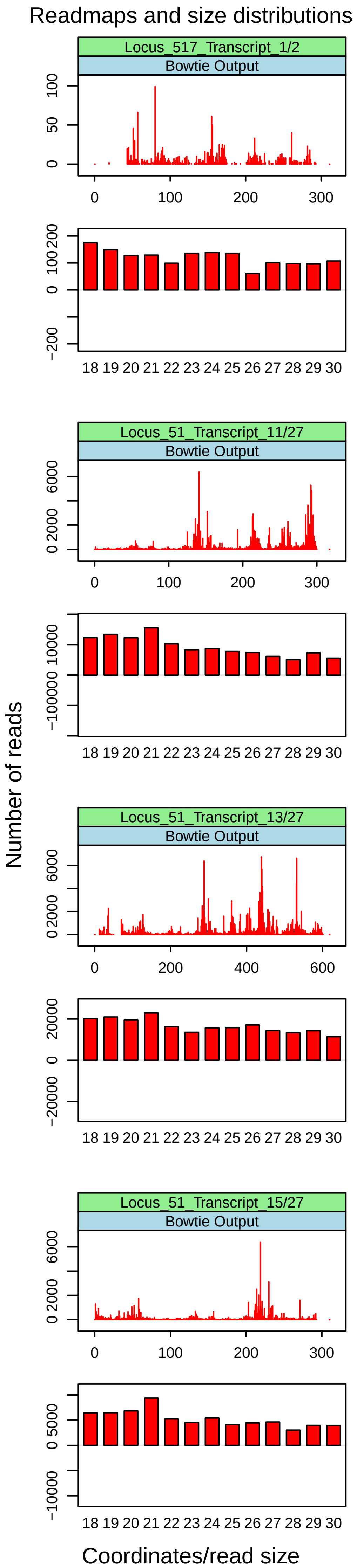
Readmaps and size distributions
Locus_517_Transcript_1/2
Bowtie Output
Locus_51_Transcript_11/27
Bowtie Output
Locus_51_Transcript_13/27
Bowtie Output
Locus_51_Transcript_15/27
Bowtie Output
Number of reads
Coordinates/read size

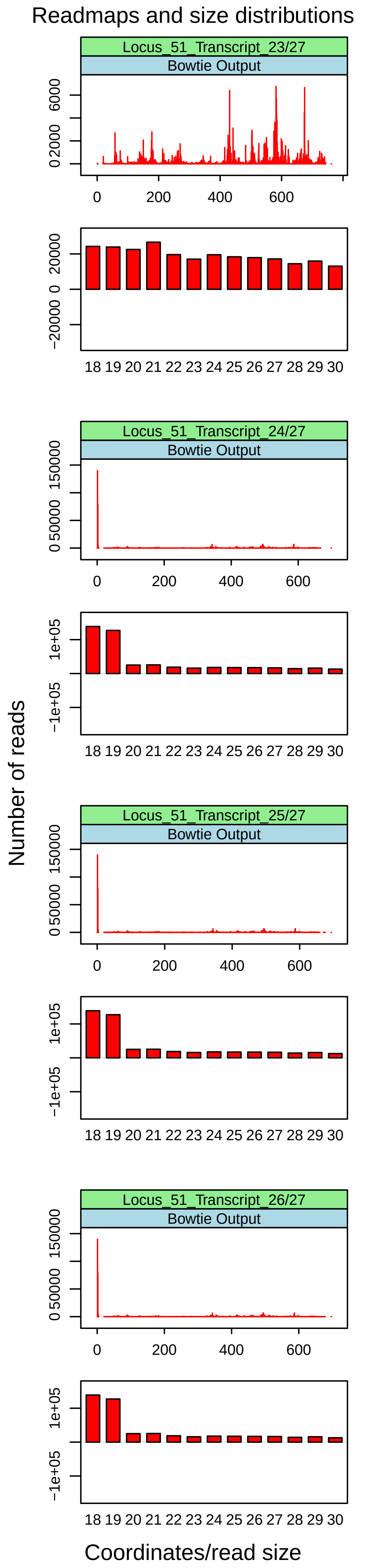

Readmaps and size distributions
Locus_51_Transcript_23/27
Bowtie Output
Locus_51_Transcript_24/27
Bowtie Output
Locus_51_Transcript_25/27
Bowtie Output
Locus_51_Transcript_26/27
Bowtie Output
Number of reads
Coordinates/read size
18 19 20 21 22 23 24 25 26 27 28 29 30

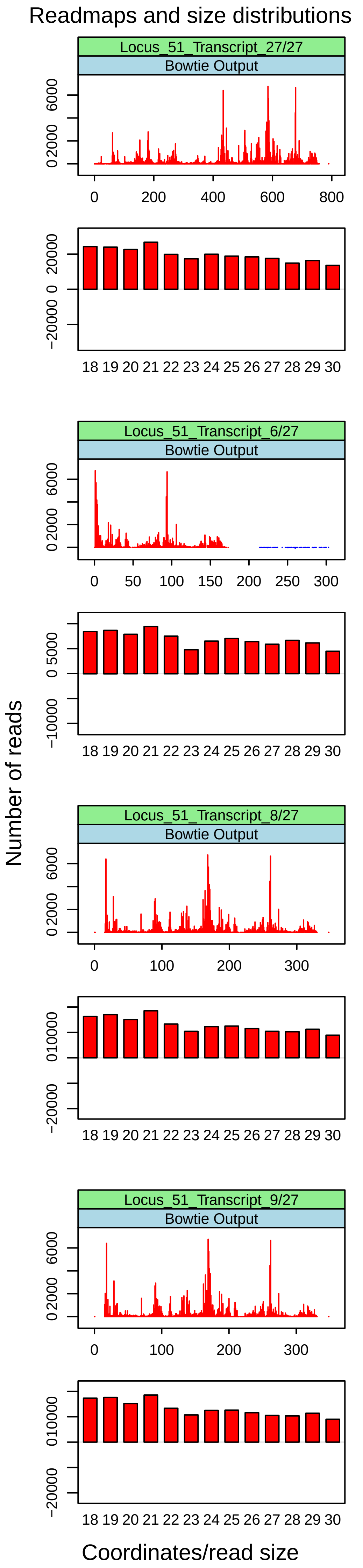
Readmaps and size distributions
Locus_51_Transcript_27/27
Bowtie Output
0 2000 6000
0 200 400 600 800
−20000 0 20000
18 19 20 21 22 23 24 25 26 27 28 29 30
Locus_51_Transcript_6/27
Bowtie Output
0 2000 6000
0 50 100 150 200 250 300
−10000 0 5000
18 19 20 21 22 23 24 25 26 27 28 29 30
Locus_51_Transcript_8/27
Bowtie Output
0 2000 6000
0 100 200 300
−20000 010000
18 19 20 21 22 23 24 25 26 27 28 29 30
Locus_51_Transcript_9/27
Bowtie Output
0 2000 6000
0 100 200 300
−20000 010000
18 19 20 21 22 23 24 25 26 27 28 29 30
Number of reads
Coordinates/read size

Readmaps and size distributions

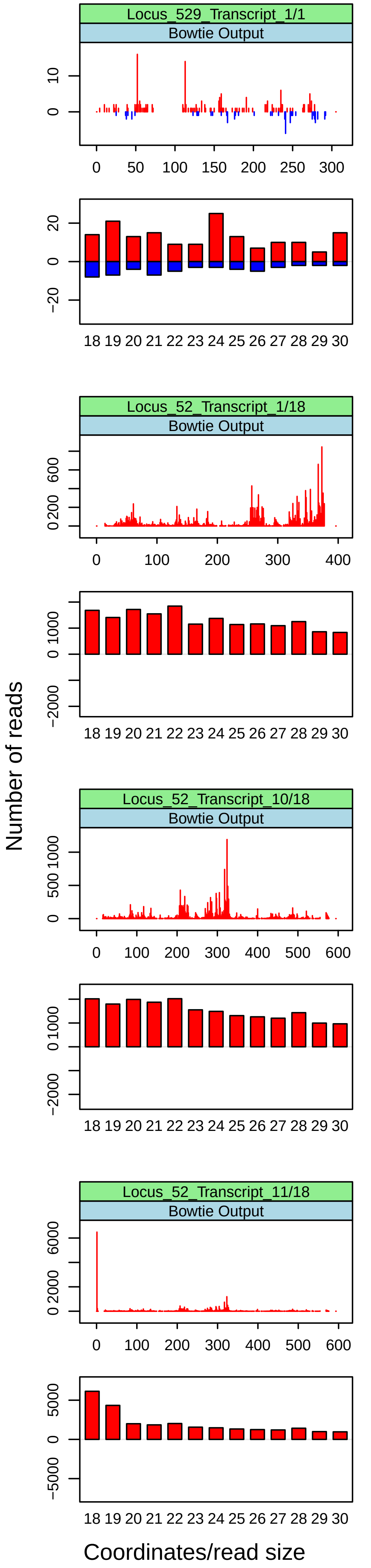

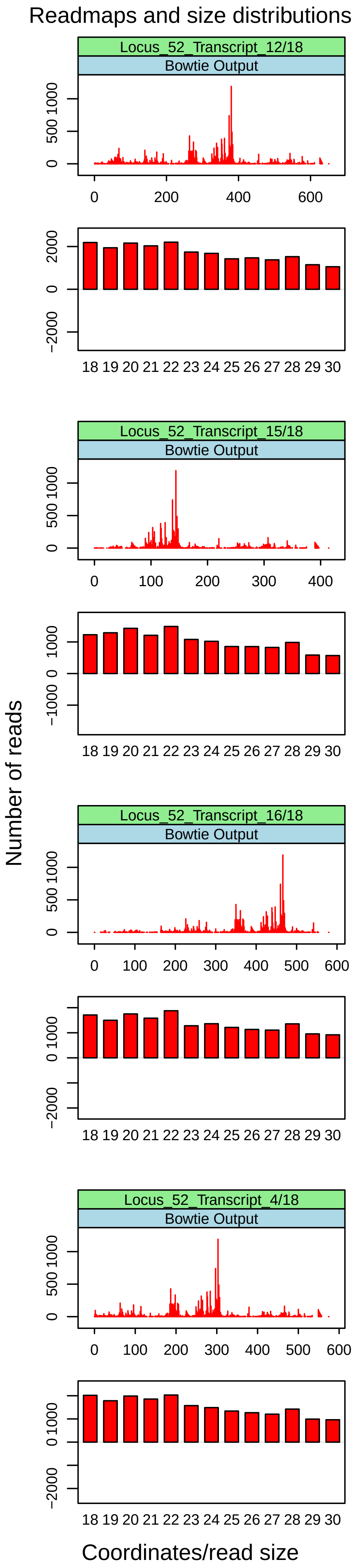
Readmaps and size distributions
Locus_52_Transcript_12/18
Bowtie Output
Locus_52_Transcript_15/18
Bowtie Output
Locus_52_Transcript_16/18
Bowtie Output
Locus_52_Transcript_4/18
Bowtie Output
Number of reads
Coordinates/read size

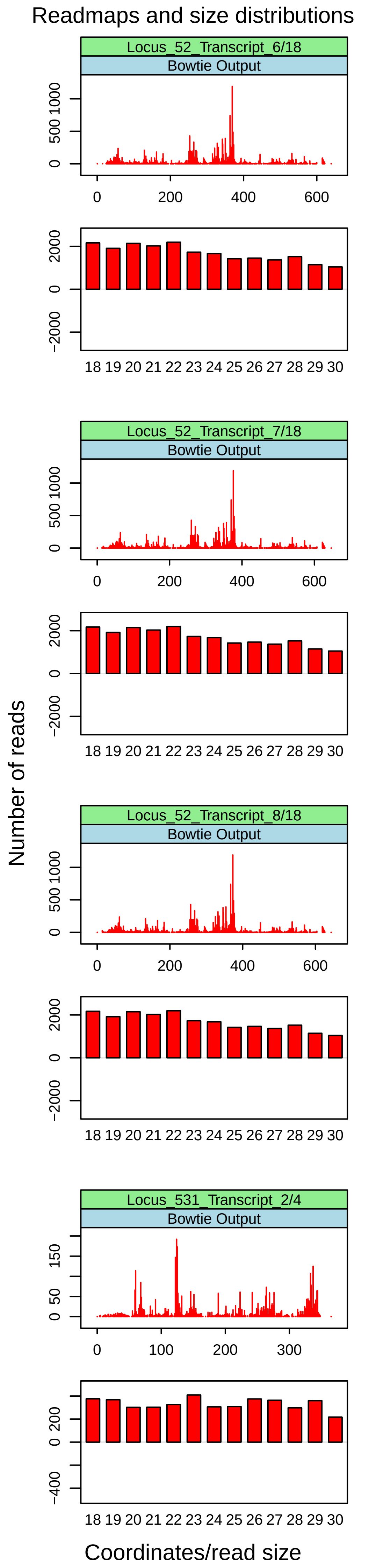

Readmaps and size distributions
Locus_52_Transcript_6/18
Bowtie Output
Locus_52_Transcript_7/18
Bowtie Output
Locus_52_Transcript_8/18
Bowtie Output
Locus_531_Transcript_2/4
Bowtie Output
Number of reads
Coordinates/read size
18 19 20 21 22 23 24 25 26 27 28 29 30

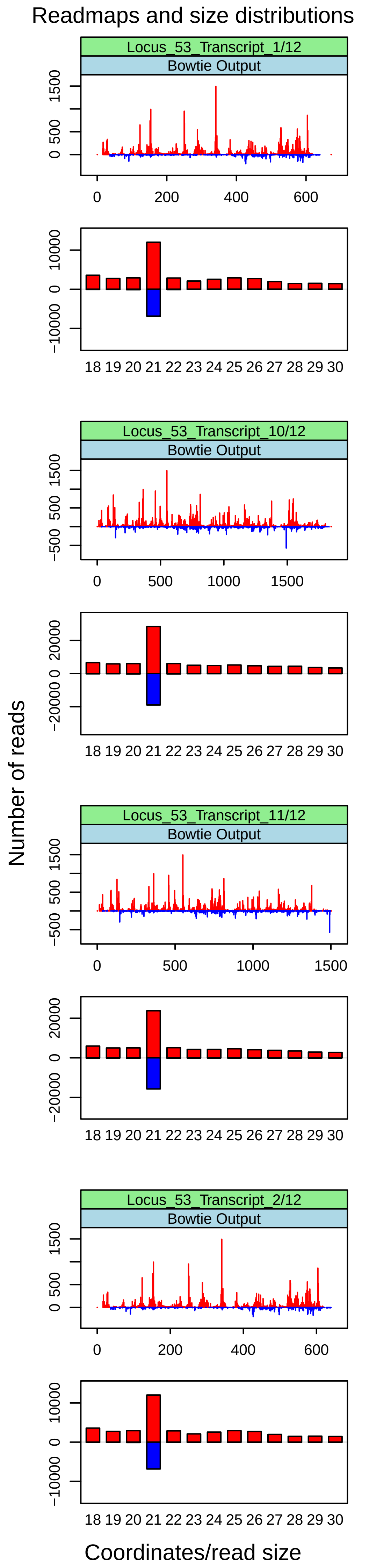
Readmaps and size distributions
Locus_53_Transcript_1/12
Bowtie Output
Locus_53_Transcript_10/12
Bowtie Output
Locus_53_Transcript_11/12
Bowtie Output
Locus_53_Transcript_2/12
Bowtie Output
Number of reads
Coordinates/read size
18 19 20 21 22 23 24 25 26 27 28 29 30

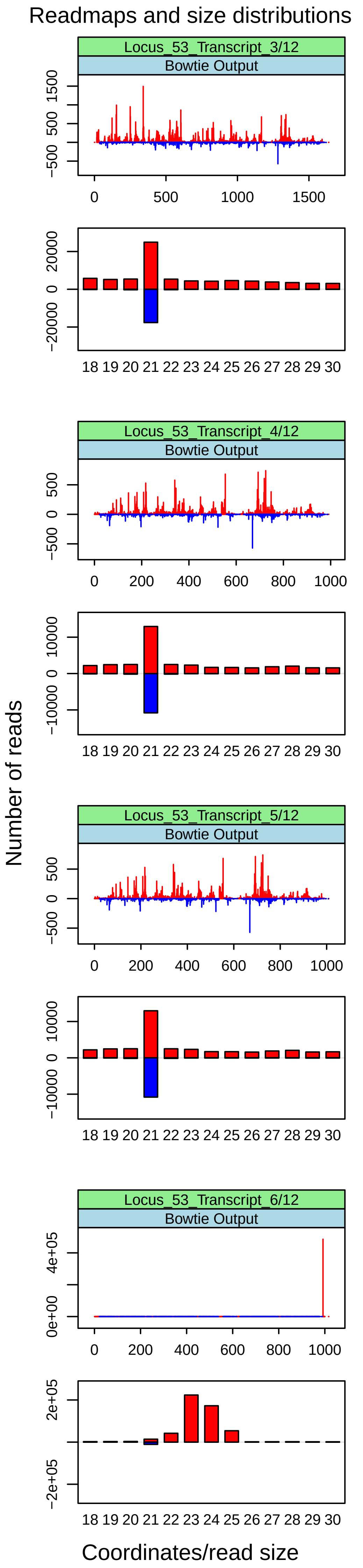

Readmaps and size distributions
Locus_53_Transcript_3/12
Bowtie Output
Locus_53_Transcript_4/12
Bowtie Output
Locus_53_Transcript_5/12
Bowtie Output
Locus_53_Transcript_6/12
Bowtie Output
Number of reads
Coordinates/read size

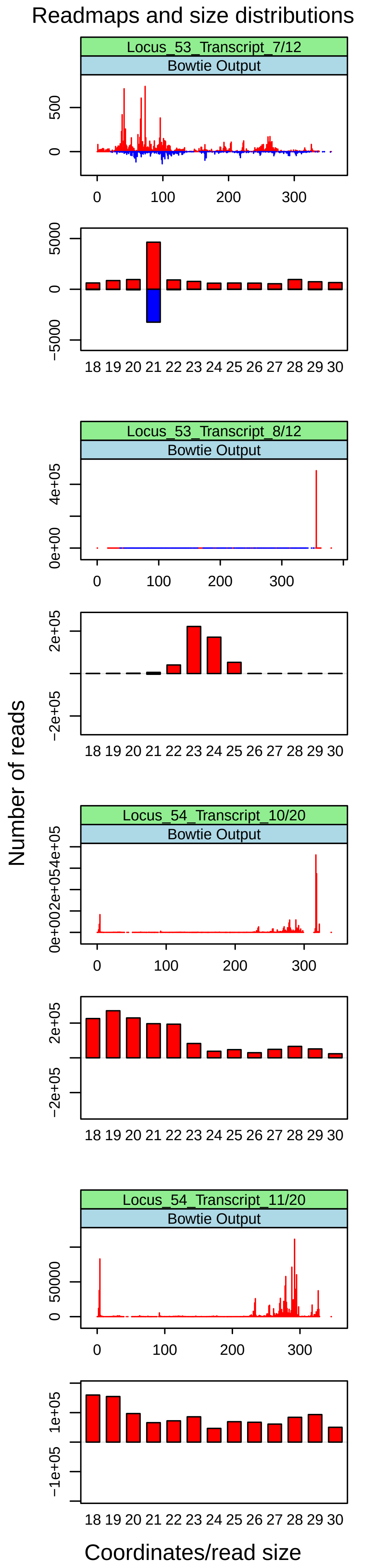

Readmaps and size distributions
Locus_53_Transcript_7/12
Bowtie Output
Locus_53_Transcript_8/12
Bowtie Output
Locus_54_Transcript_10/20
Bowtie Output
Locus_54_Transcript_11/20
Bowtie Output
Number of reads
Coordinates/read size

Readmaps and size distributions

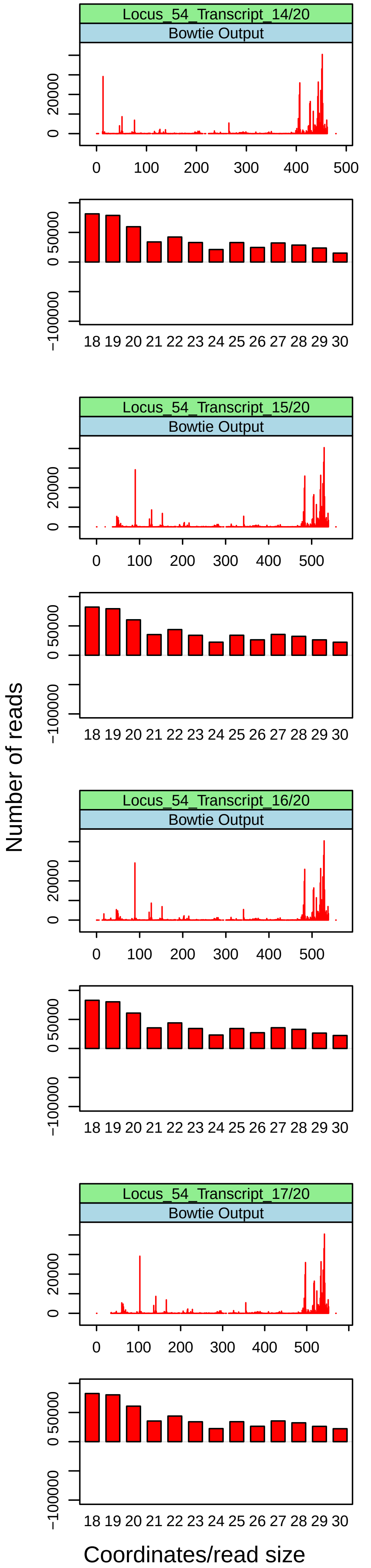

Readmaps and size distributions

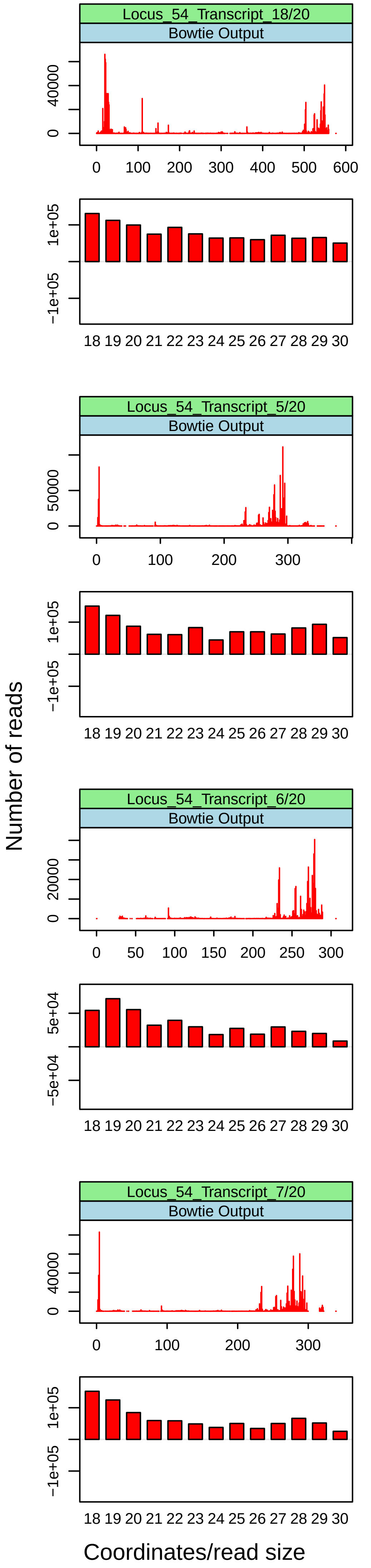

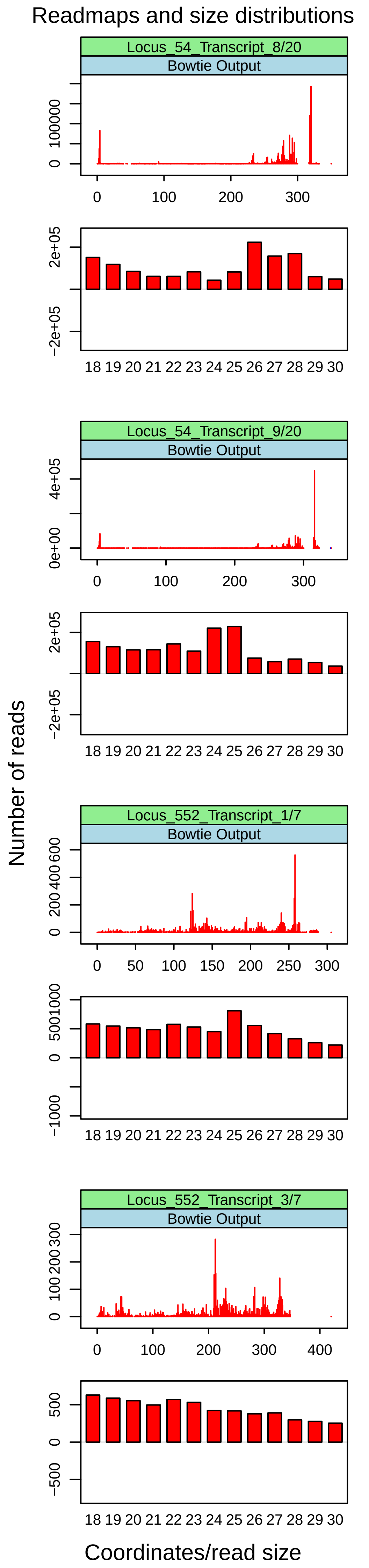
Readmaps and size distributions
Locus_54_Transcript_8/20
Bowtie Output
0
100000
0
100
200
300
−2e+05
2e+05
18 19 20 21 22 23 24 25 26 27 28 29 30
Locus_54_Transcript_9/20
Bowtie Output
0e+00
4e+05
0
100
200
300
−2e+05
2e+05
18 19 20 21 22 23 24 25 26 27 28 29 30
Locus_552_Transcript_1/7
Bowtie Output
0
200
400
600
0
50
100
150
200
250
300
−1000
0
500
1000
18 19 20 21 22 23 24 25 26 27 28 29 30
Locus_552_Transcript_3/7
Bowtie Output
0
100
200
300
0
100
200
300
400
−500
0
500
18 19 20 21 22 23 24 25 26 27 28 29 30
Number of reads
Coordinates/read size

Readmaps and size distributions

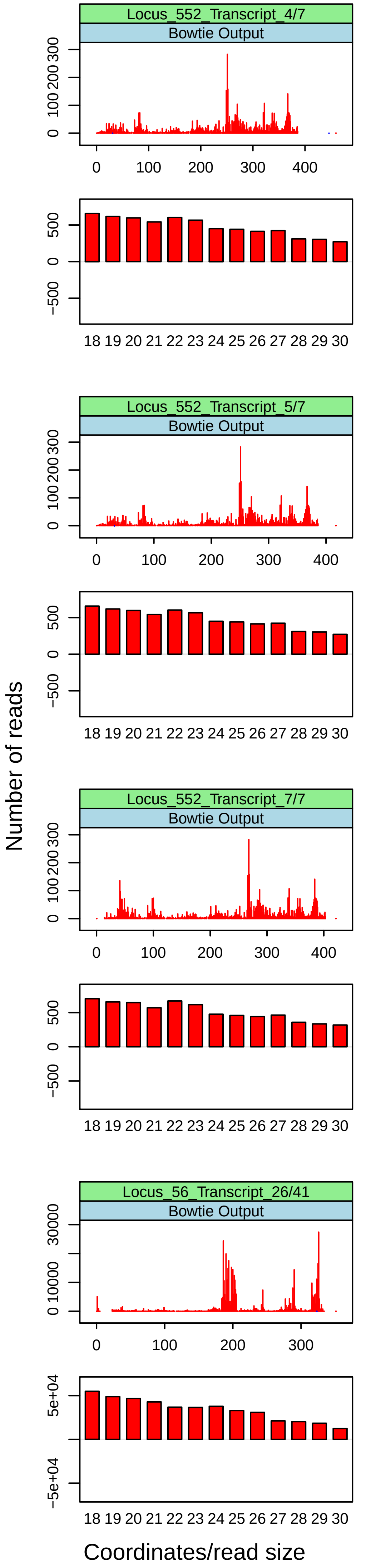

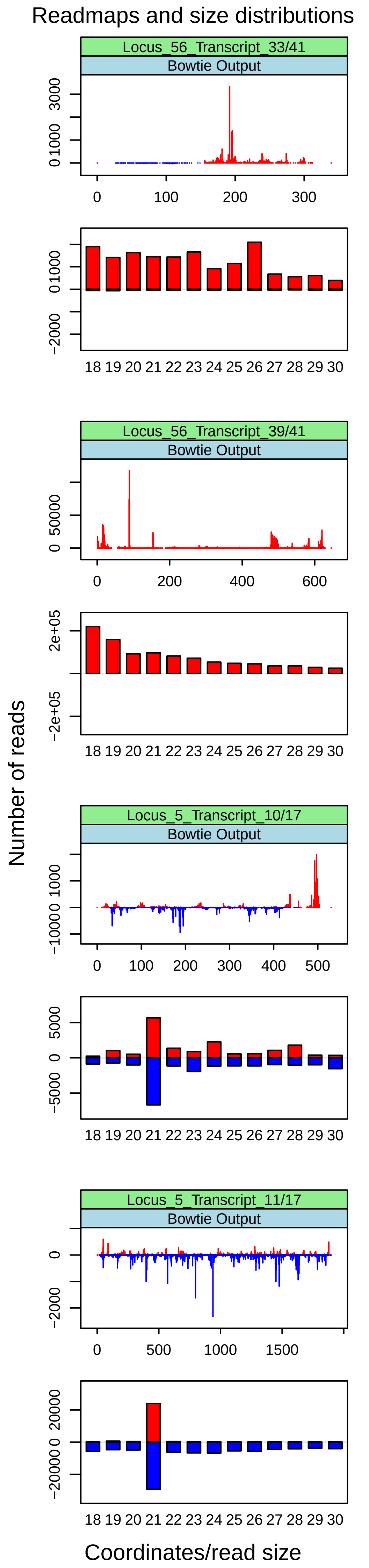
Readmaps and size distributions
Locus_56_Transcript_33/41
Bowtie Output
Locus_56_Transcript_39/41
Bowtie Output
Locus_5_Transcript_10/17
Bowtie Output
Locus_5_Transcript_11/17
Bowtie Output
Number of reads
Coordinates/read size

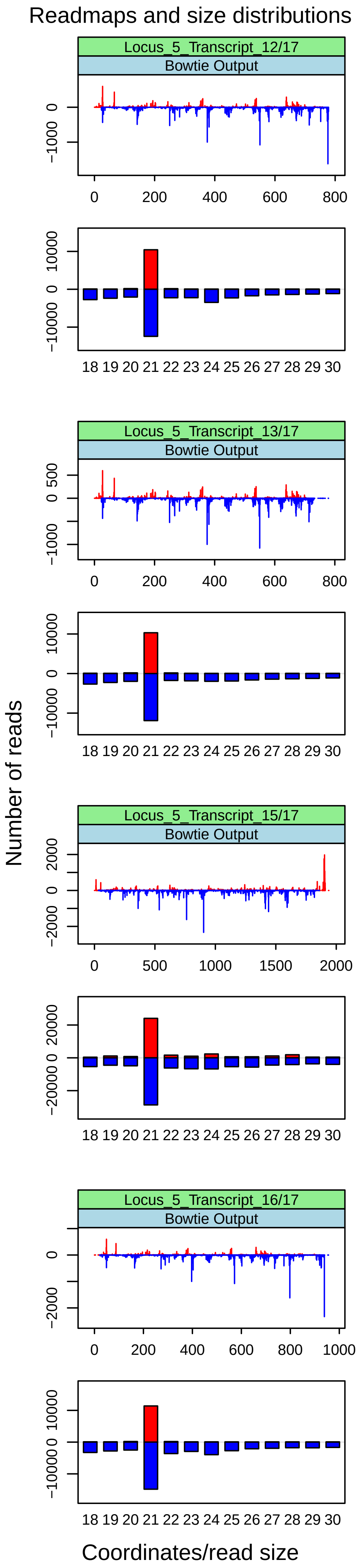
Readmaps and size distributions
Locus_5_Transcript_12/17
Bowtie Output
Locus_5_Transcript_13/17
Bowtie Output
Locus_5_Transcript_15/17
Bowtie Output
Locus_5_Transcript_16/17
Bowtie Output
Number of reads
Coordinates/read size

Readmaps and size distributions

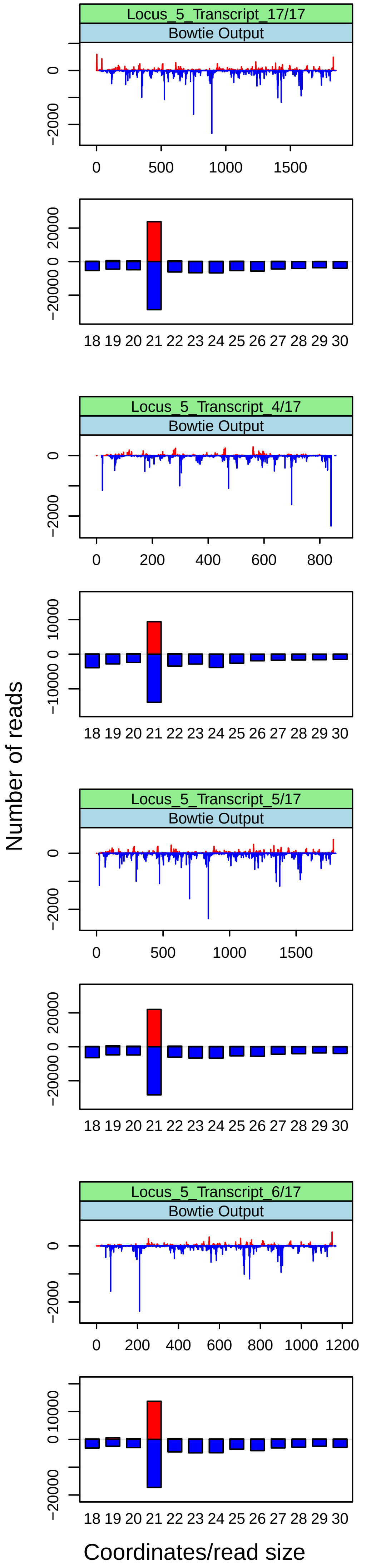

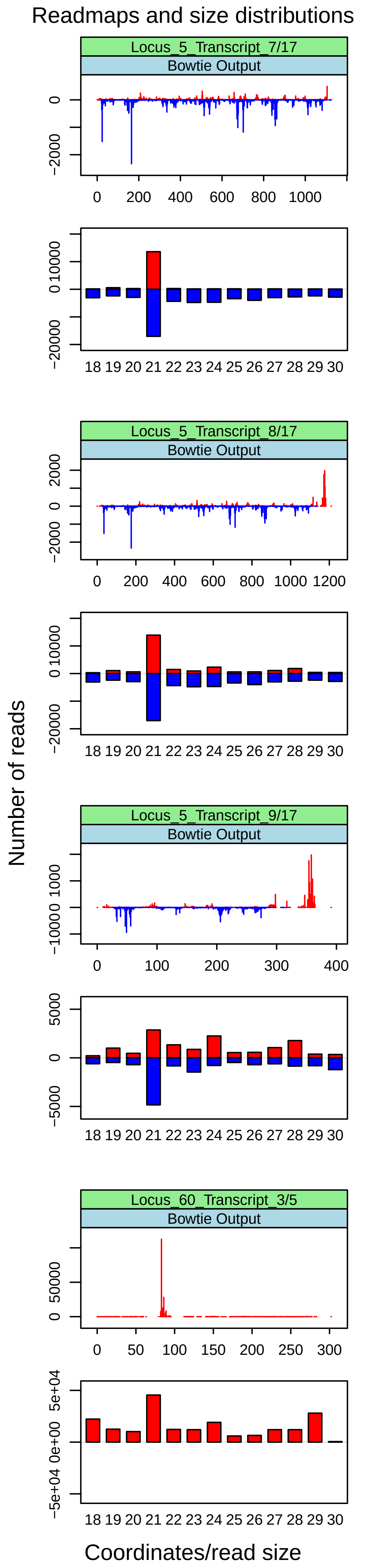
Readmaps and size distributions
Locus_5_Transcript_7/17
Bowtie Output
Locus_5_Transcript_8/17
Bowtie Output
Locus_5_Transcript_9/17
Bowtie Output
Locus_60_Transcript_3/5
Bowtie Output
Number of reads
Coordinates/read size

Readmaps and size distributions

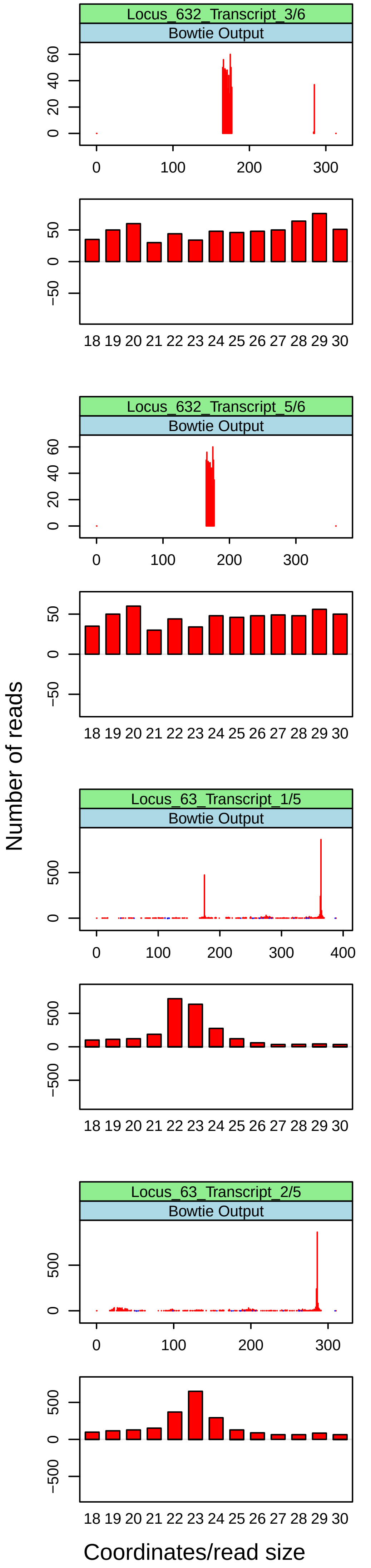

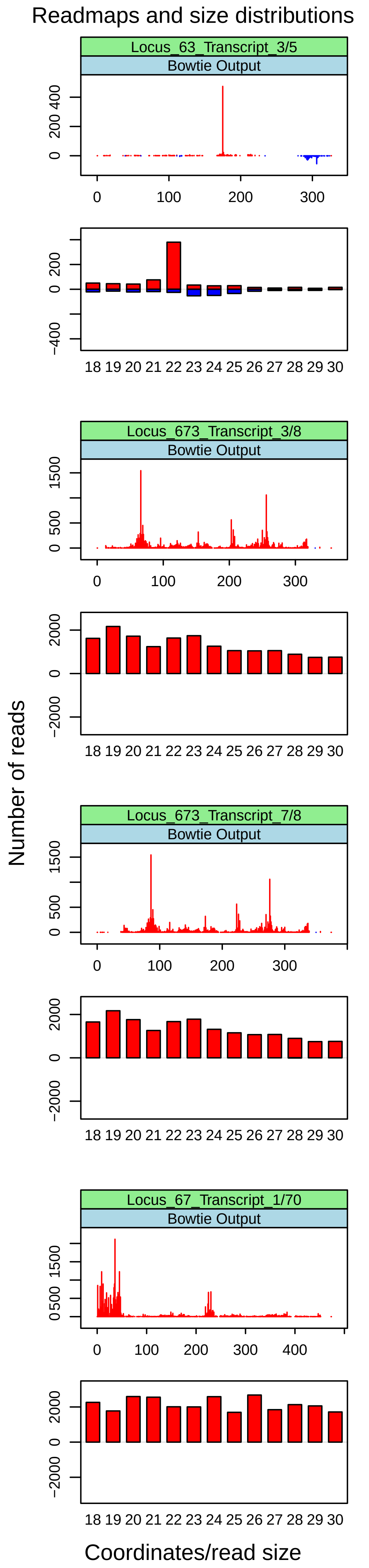
Readmaps and size distributions
Locus_63_Transcript_3/5
Bowtie Output
0
200
400
0
100
200
300
−400
200
18 19 20 21 22 23 24 25 26 27 28 29 30
Locus_673_Transcript_3/8
Bowtie Output
0
500
1500
0
100
200
300
−2000
0
2000
18 19 20 21 22 23 24 25 26 27 28 29 30
Locus_673_Transcript_7/8
Bowtie Output
0
500
1500
0
100
200
300
−2000
0
2000
18 19 20 21 22 23 24 25 26 27 28 29 30
Locus_67_Transcript_1/70
Bowtie Output
0
500
1500
0
100
200
300
400
−2000
0
2000
18 19 20 21 22 23 24 25 26 27 28 29 30
Number of reads
Coordinates/read size

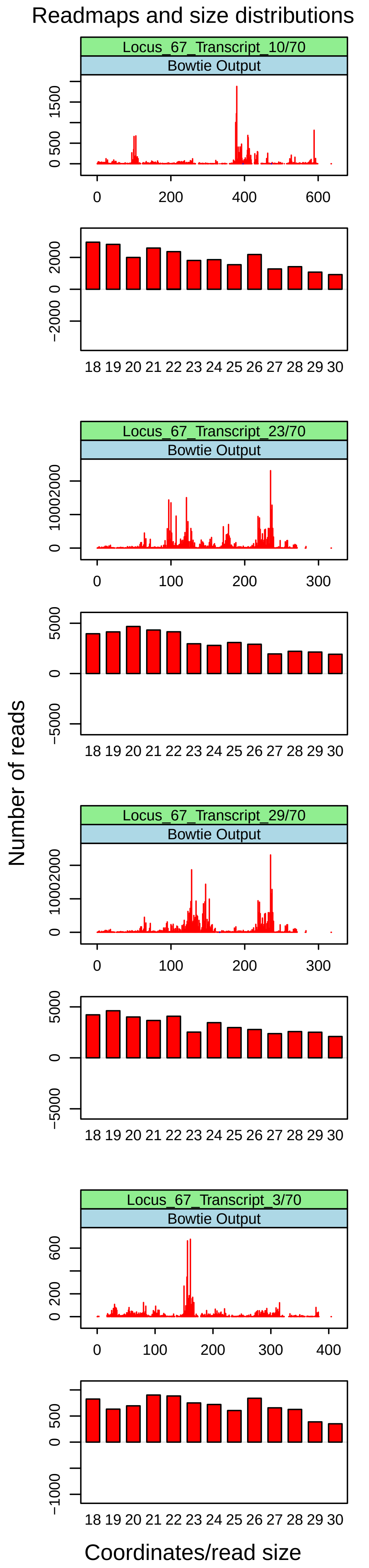

Readmaps and size distributions
Locus_67_Transcript_10/70
Bowtie Output
Locus_67_Transcript_23/70
Bowtie Output
Locus_67_Transcript_29/70
Bowtie Output
Locus_67_Transcript_3/70
Bowtie Output
Number of reads
Coordinates/read size

Readmaps and size distributions

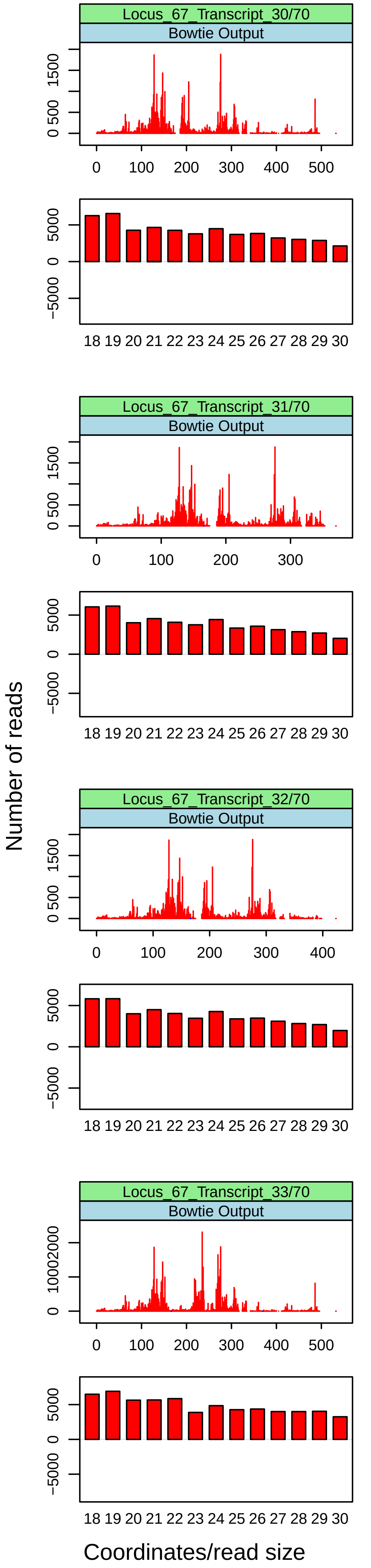

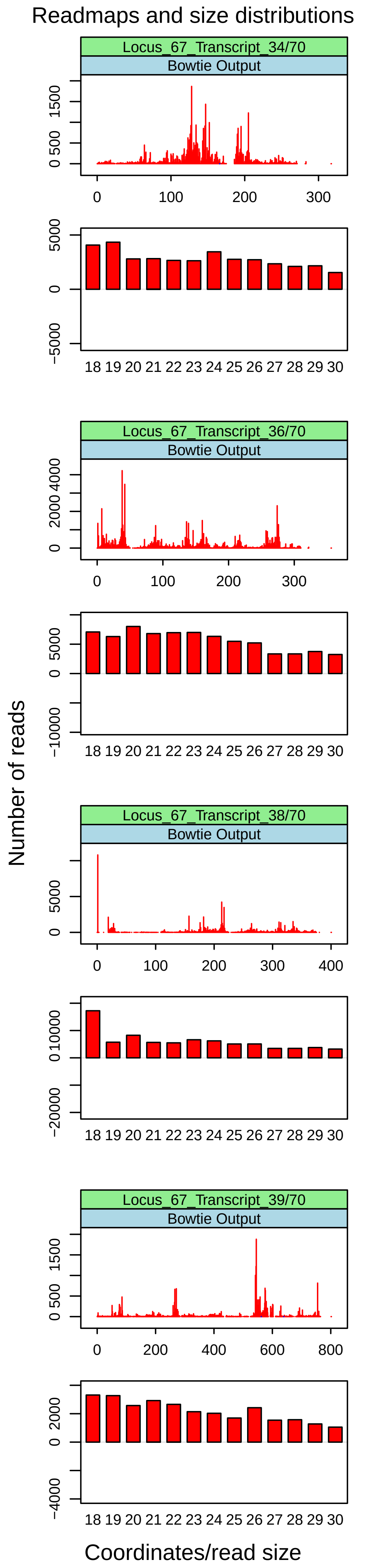
Readmaps and size distributions
Locus_67_Transcript_34/70
Bowtie Output
Locus_67_Transcript_36/70
Bowtie Output
Locus_67_Transcript_38/70
Bowtie Output
Locus_67_Transcript_39/70
Bowtie Output
Number of reads
Coordinates/read size

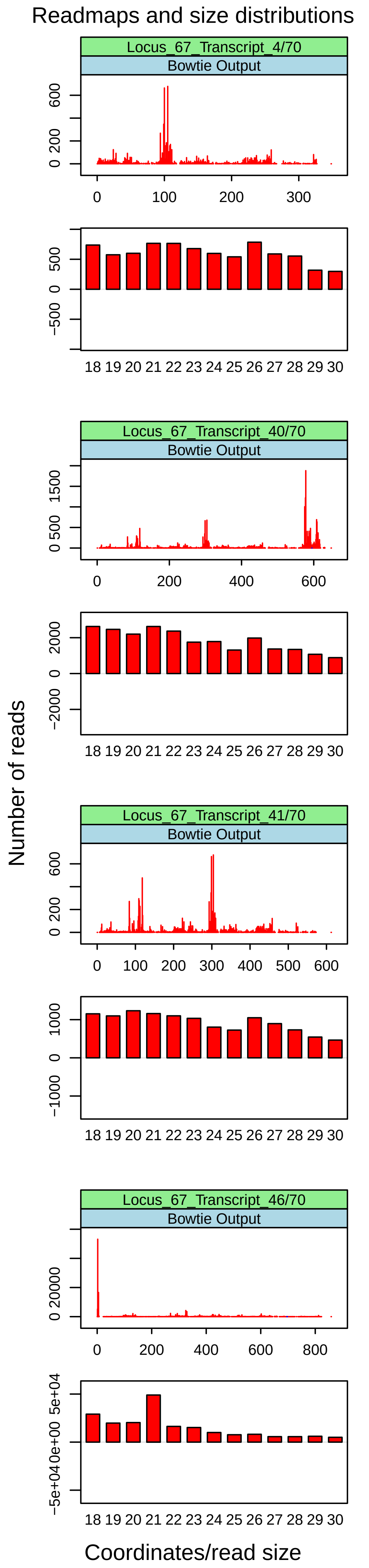
Readmaps and size distributions
Locus_67_Transcript_4/70
Bowtie Output
Locus_67_Transcript_40/70
Bowtie Output
Locus_67_Transcript_41/70
Bowtie Output
Locus_67_Transcript_46/70
Bowtie Output
18 19 20 21 22 23 24 25 26 27 28 29 30
Number of reads
Coordinates/read size

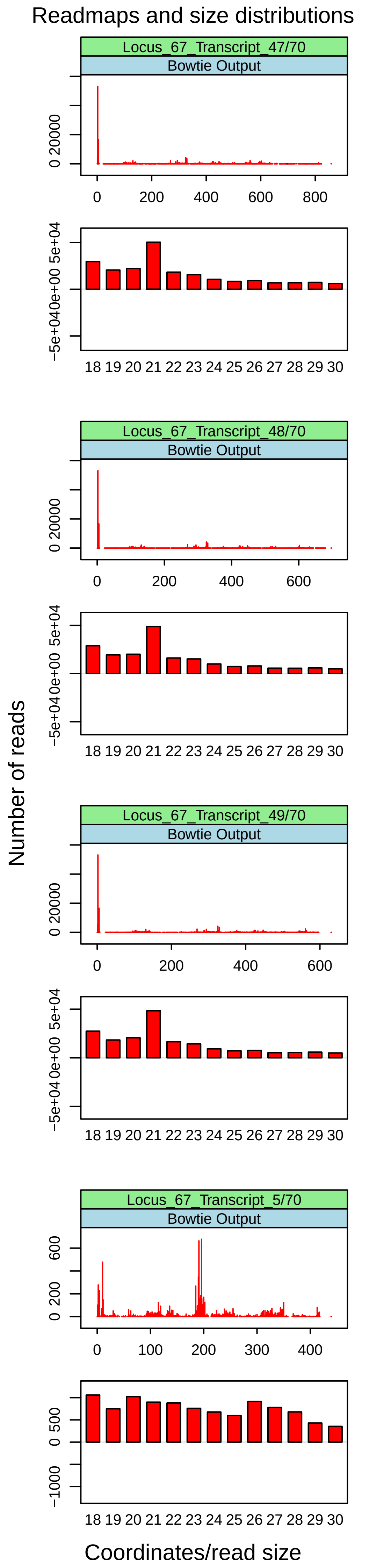

Readmaps and size distributions
Locus_67_Transcript_47/70
Bowtie Output
0 20000
0 200 400 600 800
−5e+04 0e+00 5e+04
18 19 20 21 22 23 24 25 26 27 28 29 30
Locus_67_Transcript_48/70
Bowtie Output
0 20000
0 200 400 600
−5e+04 0e+00 5e+04
18 19 20 21 22 23 24 25 26 27 28 29 30
Locus_67_Transcript_49/70
Bowtie Output
0 20000
0 200 400 600
−5e+04 0e+00 5e+04
18 19 20 21 22 23 24 25 26 27 28 29 30
Locus_67_Transcript_5/70
Bowtie Output
0 200 600
0 100 200 300 400
−1000 0 500
18 19 20 21 22 23 24 25 26 27 28 29 30
Number of reads
Coordinates/read size

Readmaps and size distributions

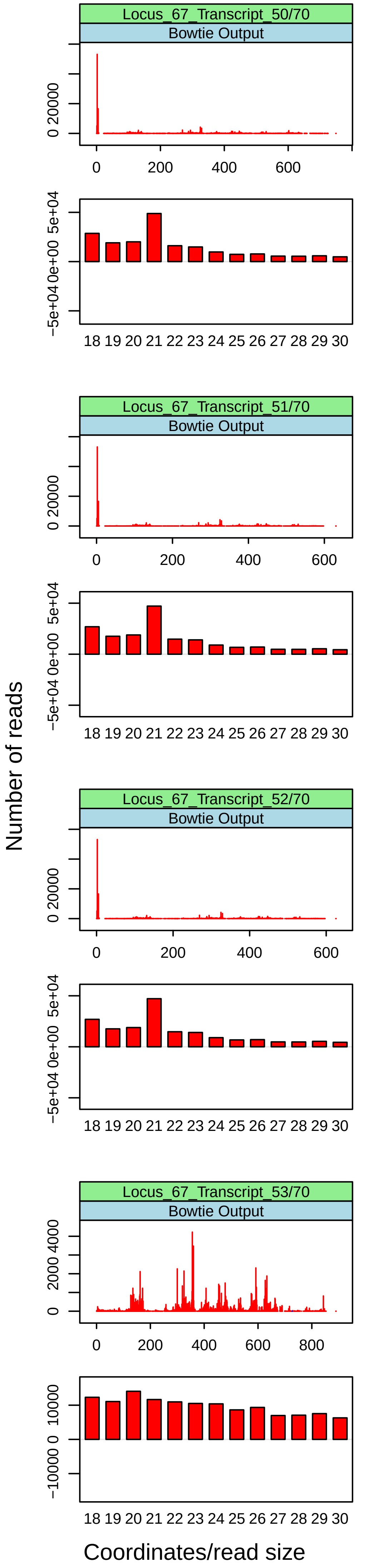

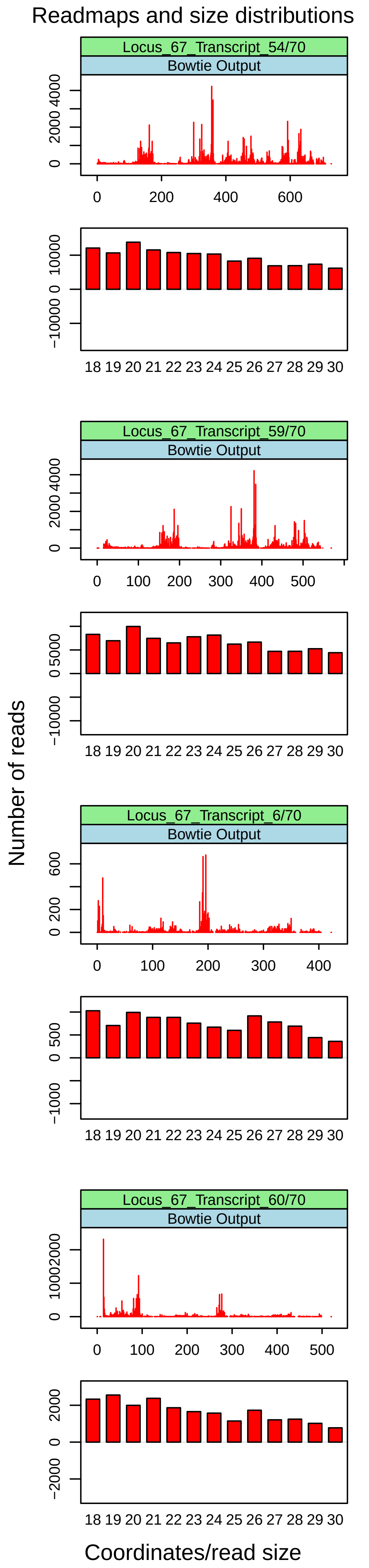

Readmaps and size distributions
Locus_67_Transcript_54/70
Bowtie Output
0 2000 4000
0 200 400 600
−10000 0 10000
18 19 20 21 22 23 24 25 26 27 28 29 30
Locus_67_Transcript_59/70
Bowtie Output
0 2000 4000
0 100 200 300 400 500
−10000 0 5000
18 19 20 21 22 23 24 25 26 27 28 29 30
Locus_67_Transcript_6/70
Bowtie Output
0 200 600
0 100 200 300 400
−1000 0 500
18 19 20 21 22 23 24 25 26 27 28 29 30
Locus_67_Transcript_60/70
Bowtie Output
0 1000 2000
0 100 200 300 400 500
−2000 0 2000
18 19 20 21 22 23 24 25 26 27 28 29 30
Number of reads
Coordinates/read size

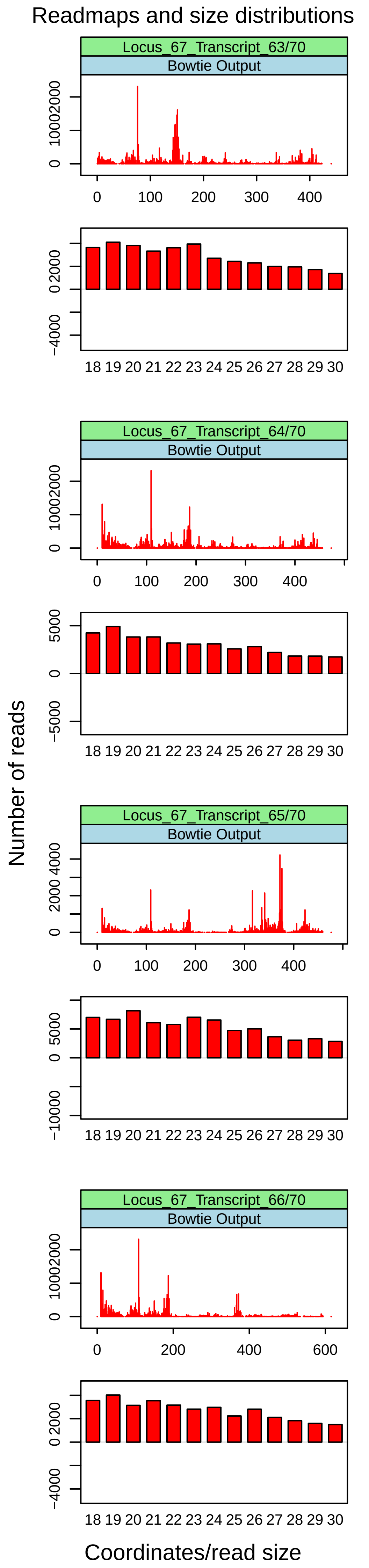

Readmaps and size distributions
Locus_67_Transcript_63/70
Bowtie Output
0
1000
2000
0
100
200
300
400
−4000
0
2000
18 19 20 21 22 23 24 25 26 27 28 29 30
Locus_67_Transcript_64/70
Bowtie Output
0
1000
2000
0
100
200
300
400
−5000
0
5000
18 19 20 21 22 23 24 25 26 27 28 29 30
Locus_67_Transcript_65/70
Bowtie Output
0
2000
4000
0
100
200
300
400
−10000
0
5000
18 19 20 21 22 23 24 25 26 27 28 29 30
Locus_67_Transcript_66/70
Bowtie Output
0
1000
2000
0
200
400
600
−4000
0
2000
18 19 20 21 22 23 24 25 26 27 28 29 30
Number of reads
Coordinates/read size

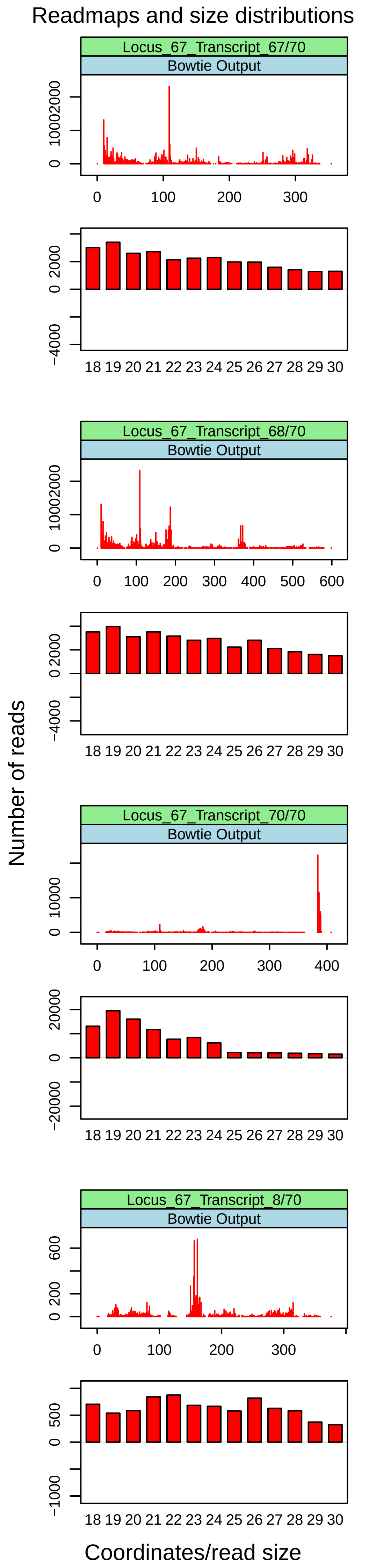
Readmaps and size distributions
Locus_67_Transcript_67/70
Bowtie Output
0
10002000
0 100 200 300
−4000
0 2000
18 19 20 21 22 23 24 25 26 27 28 29 30
Locus_67_Transcript_68/70
Bowtie Output
0
10002000
0 100 200 300 400 500 600
−4000
0 2000
18 19 20 21 22 23 24 25 26 27 28 29 30
Locus_67_Transcript_70/70
Bowtie Output
0
10000
0 100 200 300 400
−20000
0
20000
18 19 20 21 22 23 24 25 26 27 28 29 30
Locus_67_Transcript_8/70
Bowtie Output
0
200
600
0 100 200 300
−1000
0
500
18 19 20 21 22 23 24 25 26 27 28 29 30
Number of reads
Coordinates/read size

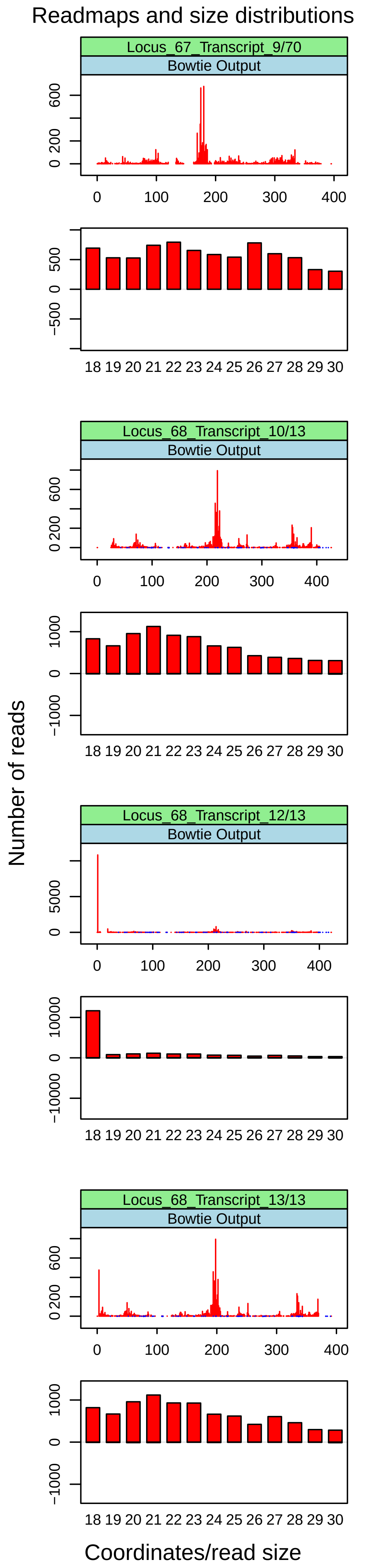
Readmaps and size distributions
Locus_67_Transcript_9/70
Bowtie Output
Locus_68_Transcript_10/13
Bowtie Output
Locus_68_Transcript_12/13
Bowtie Output
Locus_68_Transcript_13/13
Bowtie Output
18 19 20 21 22 23 24 25 26 27 28 29 30
Number of reads
Coordinates/read size

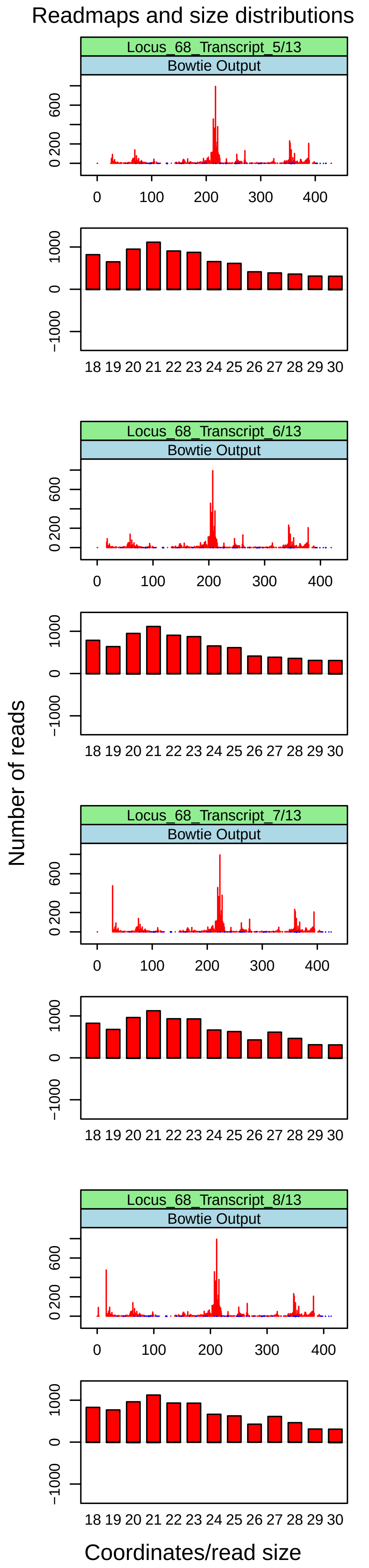

Readmaps and size distributions
Locus_68_Transcript_5/13
Bowtie Output
Locus_68_Transcript_6/13
Bowtie Output
Locus_68_Transcript_7/13
Bowtie Output
Locus_68_Transcript_8/13
Bowtie Output
Number of reads
Coordinates/read size

Readmaps and size distributions

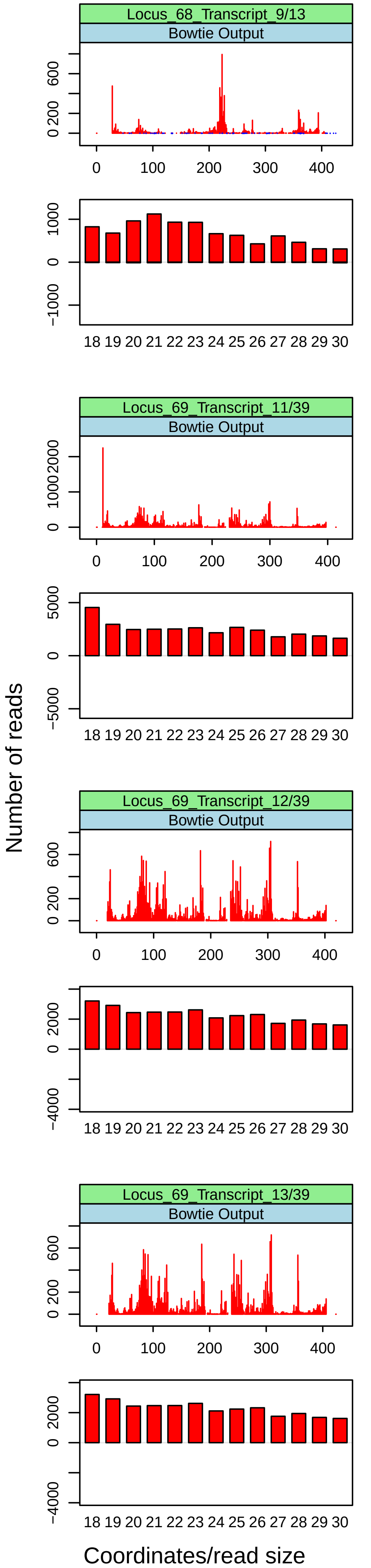

Readmaps and size distributions

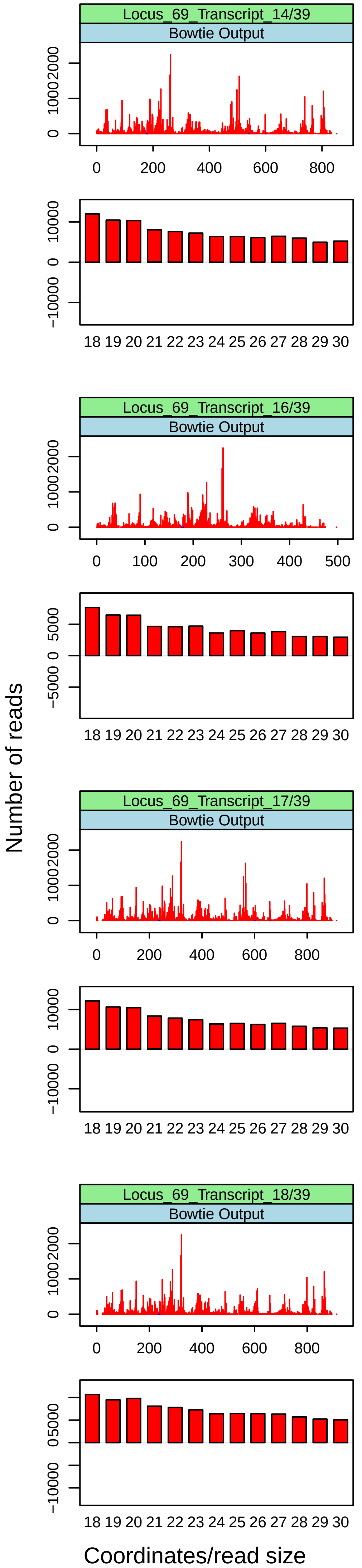

Readmaps and size distributions

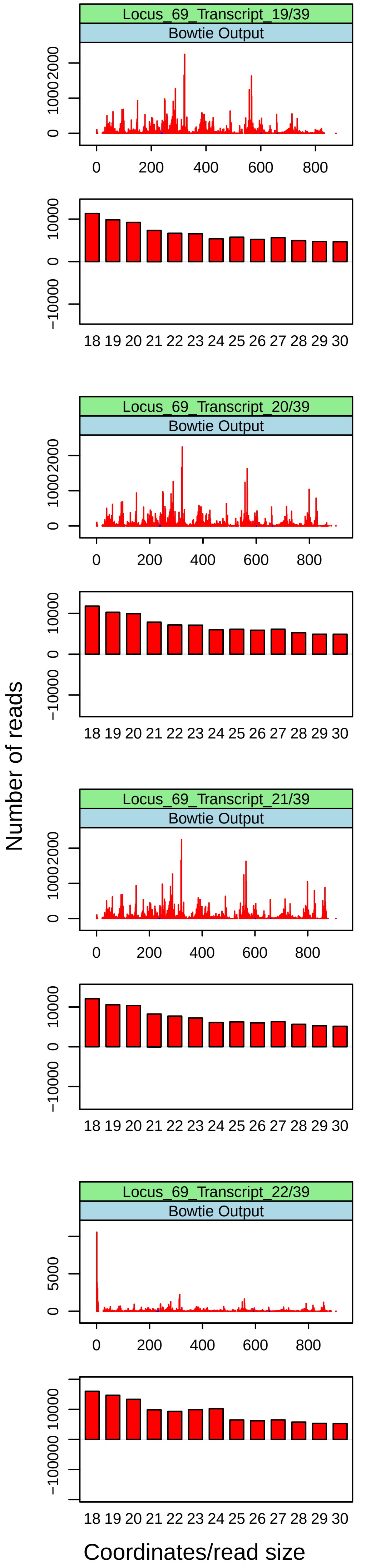

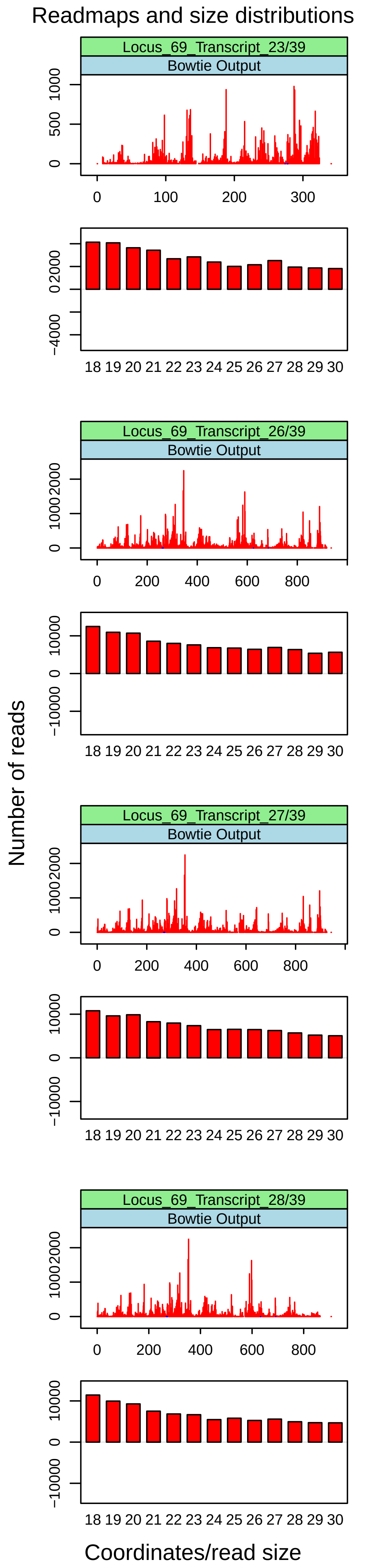
Readmaps and size distributions
Locus_69_Transcript_23/39
Bowtie Output
Locus_69_Transcript_26/39
Bowtie Output
Locus_69_Transcript_27/39
Bowtie Output
Locus_69_Transcript_28/39
Bowtie Output
Number of reads
Coordinates/read size

Readmaps and size distributions

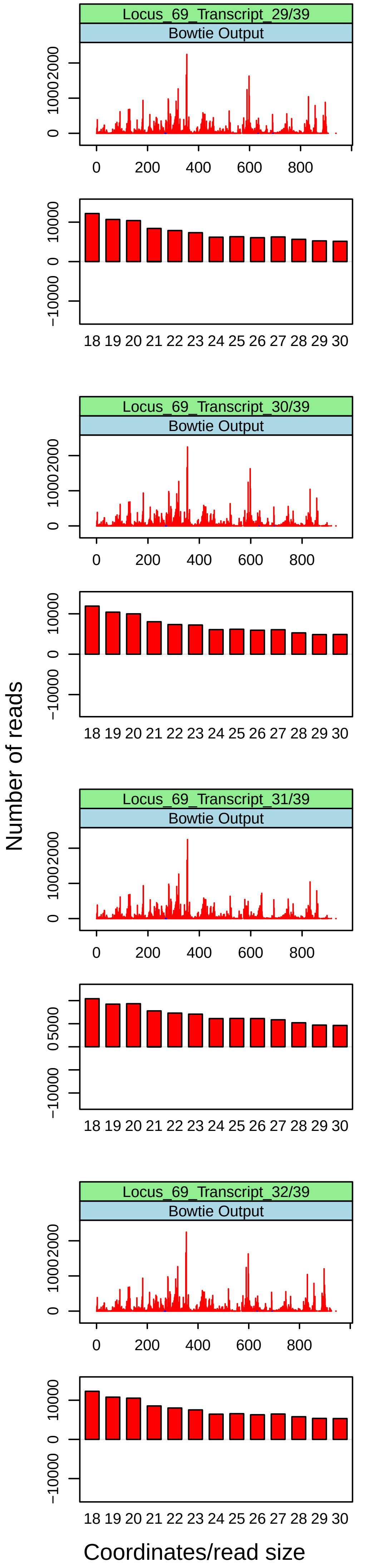

Readmaps and size distributions

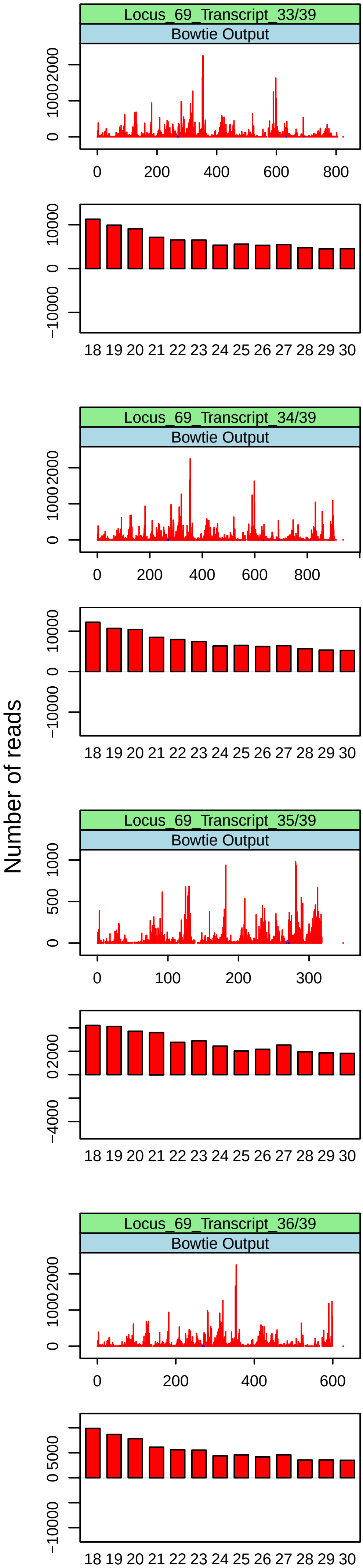

Coordinates/read size

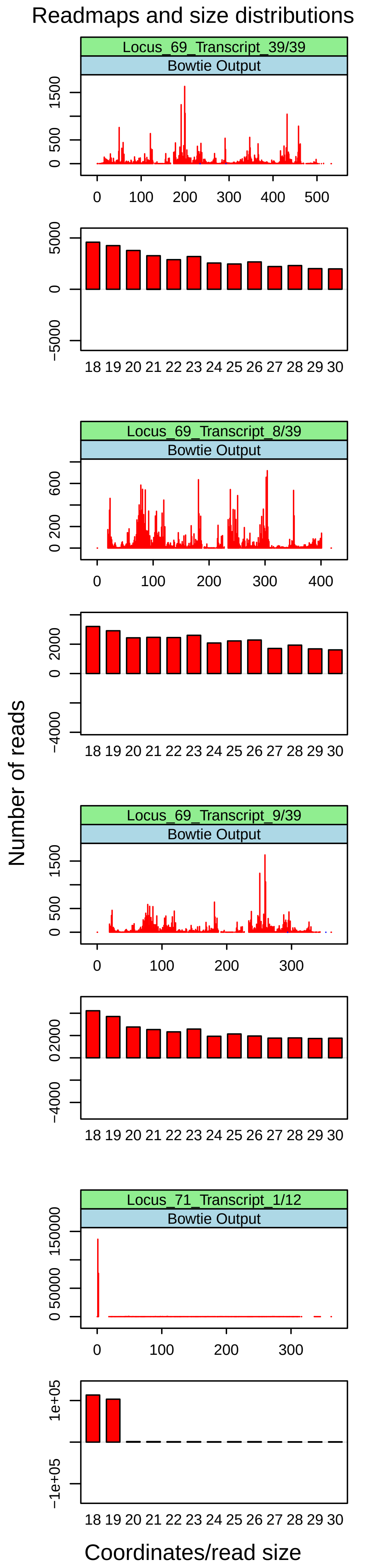

Readmaps and size distributions
Locus_69_Transcript_39/39
Bowtie Output
Locus_69_Transcript_8/39
Bowtie Output
Locus_69_Transcript_9/39
Bowtie Output
Locus_71_Transcript_1/12
Bowtie Output
Number of reads
Coordinates/read size
18 19 20 21 22 23 24 25 26 27 28 29 30

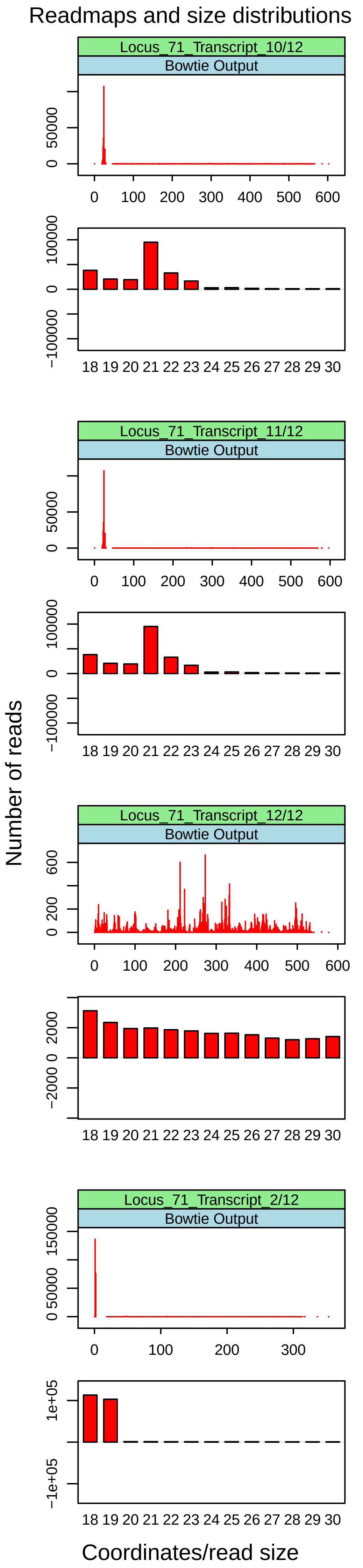
Readmaps and size distributions
Locus_71_Transcript_10/12
Bowtie Output
0
50000
0 100 200 300 400 500 600
−100000
0
100000
18 19 20 21 22 23 24 25 26 27 28 29 30
Locus_71_Transcript_11/12
Bowtie Output
0
50000
0 100 200 300 400 500 600
−100000
0
100000
18 19 20 21 22 23 24 25 26 27 28 29 30
Locus_71_Transcript_12/12
Bowtie Output
0
200
600
0 100 200 300 400 500 600
−2000 0
2000
18 19 20 21 22 23 24 25 26 27 28 29 30
Locus_71_Transcript_2/12
Bowtie Output
0 50000
150000
0 100 200 300
−1e+05
1e+05
18 19 20 21 22 23 24 25 26 27 28 29 30
Number of reads
Coordinates/read size

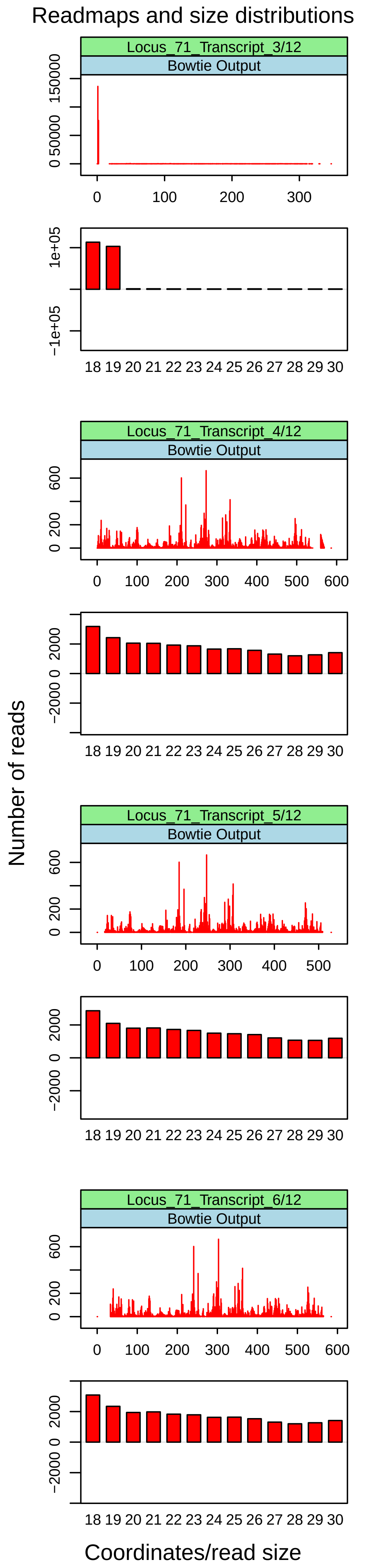
Readmaps and size distributions
Locus_71_Transcript_3/12
Bowtie Output
Locus_71_Transcript_4/12
Bowtie Output
Locus_71_Transcript_5/12
Bowtie Output
Locus_71_Transcript_6/12
Bowtie Output
Number of reads
Coordinates/read size
18 19 20 21 22 23 24 25 26 27 28 29 30

Readmaps and size distributions

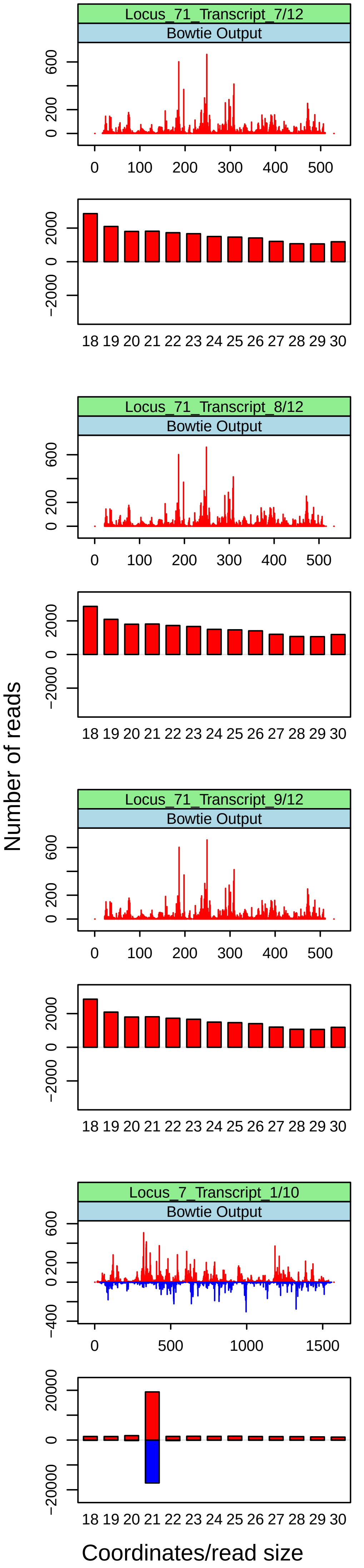

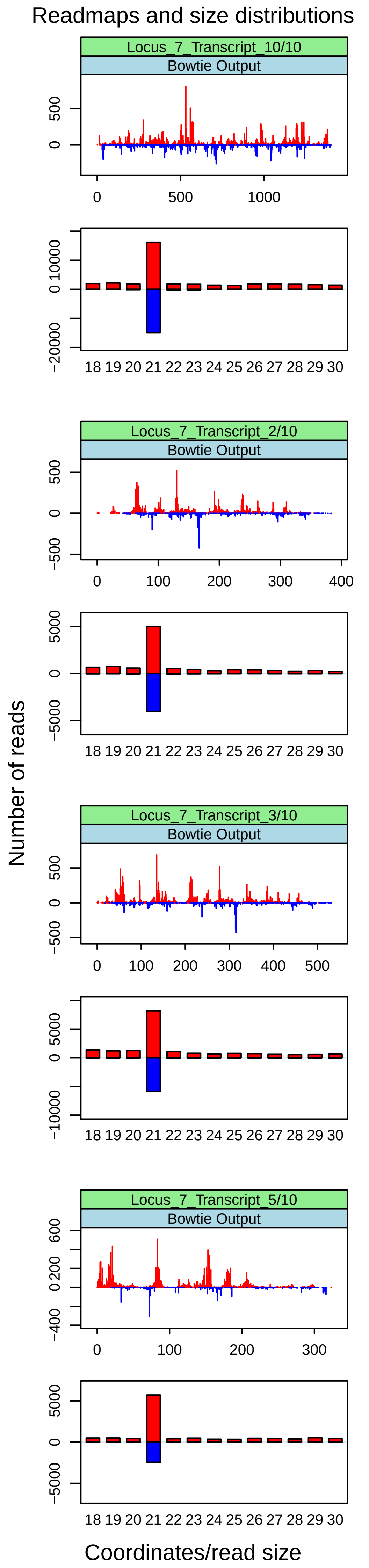
Readmaps and size distributions
Locus_7_Transcript_10/10
Bowtie Output
0
500
1000
Locus_7_Transcript_2/10
Bowtie Output
Locus_7_Transcript_3/10
Bowtie Output
Locus_7_Transcript_5/10
Bowtie Output
18 19 20 21 22 23 24 25 26 27 28 29 30
Number of reads
Coordinates/read size

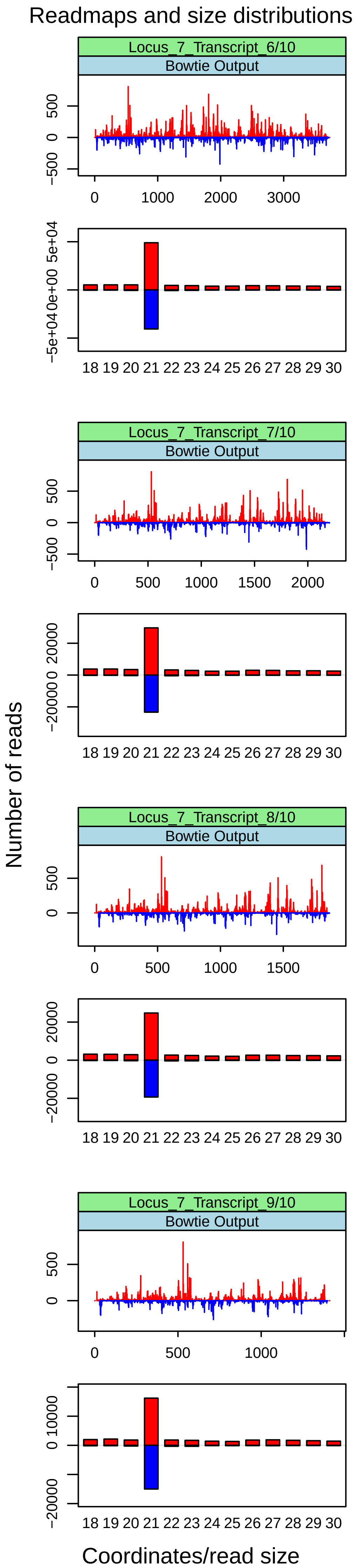
Readmaps and size distributions
Locus_7_Transcript_6/10
Bowtie Output
Locus_7_Transcript_7/10
Bowtie Output
Locus_7_Transcript_8/10
Bowtie Output
Locus_7_Transcript_9/10
Bowtie Output
Number of reads
Coordinates/read size

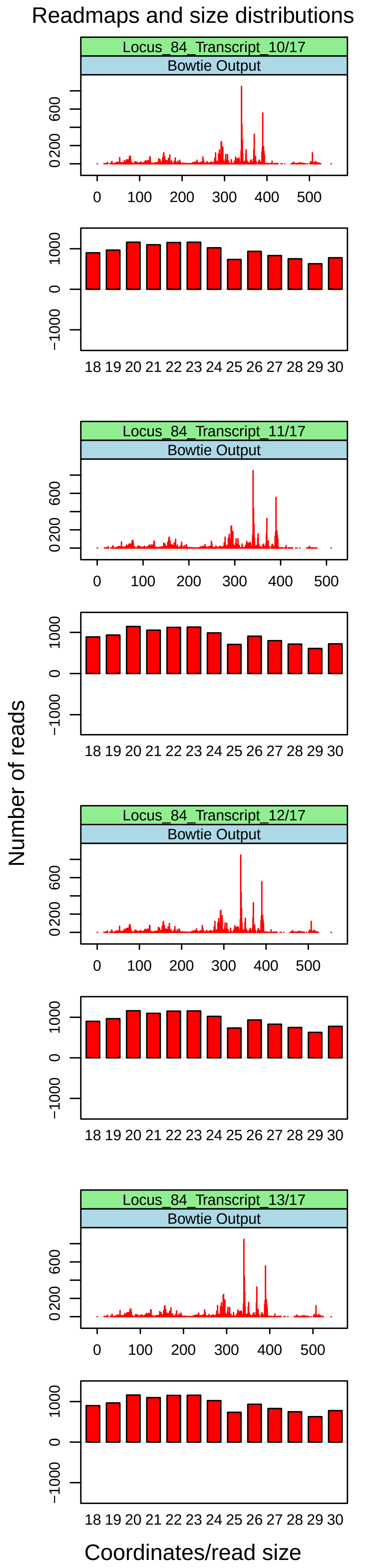
Readmaps and size distributions
Locus_84_Transcript_10/17
Bowtie Output
Locus_84_Transcript_11/17
Bowtie Output
Locus_84_Transcript_12/17
Bowtie Output
Locus_84_Transcript_13/17
Bowtie Output
Number of reads
Coordinates/read size

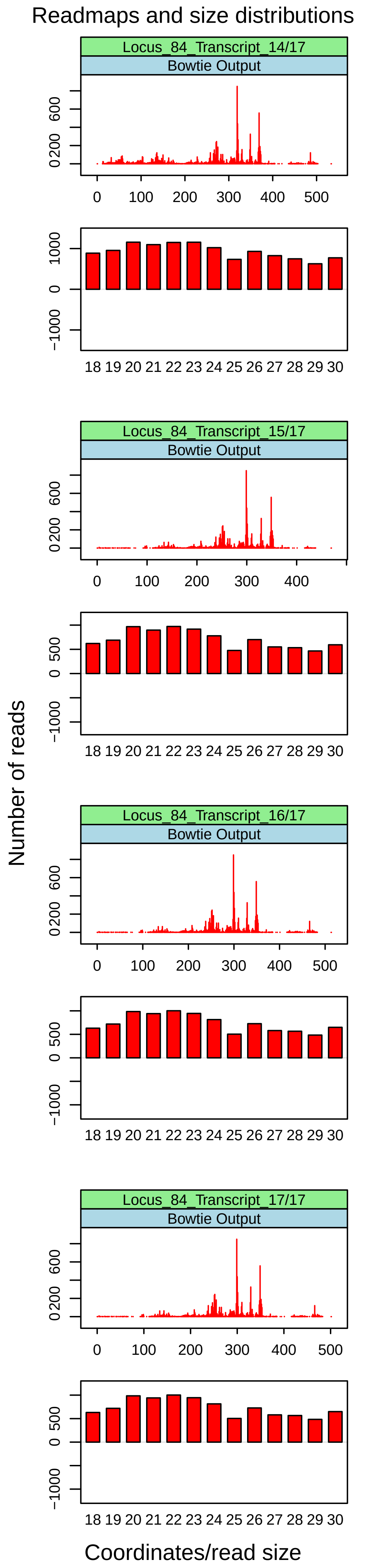
Readmaps and size distributions
Locus_84_Transcript_14/17
Bowtie Output
Locus_84_Transcript_15/17
Bowtie Output
Locus_84_Transcript_16/17
Bowtie Output
Locus_84_Transcript_17/17
Bowtie Output
Number of reads
Coordinates/read size

Readmaps and size distributions

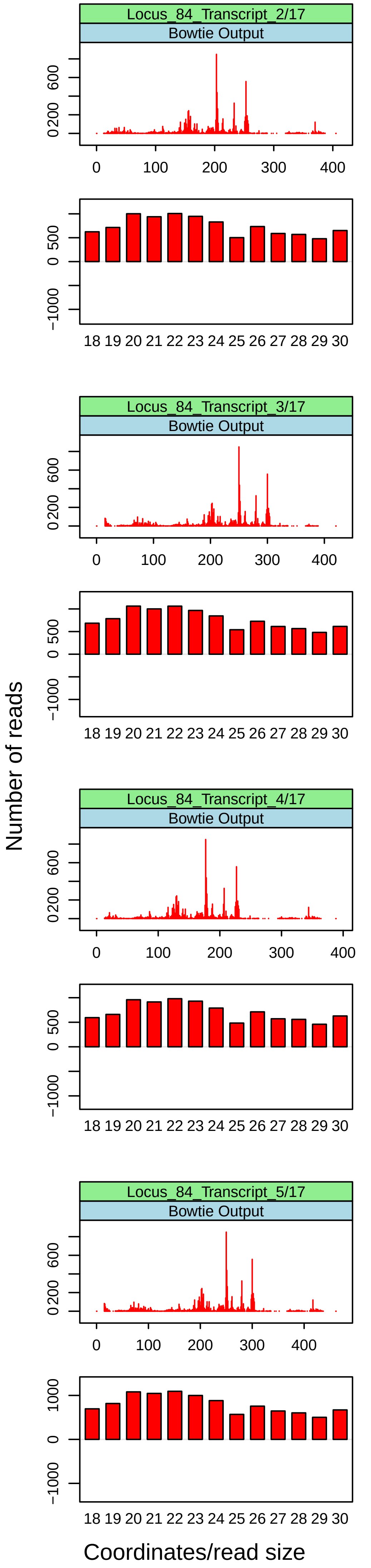

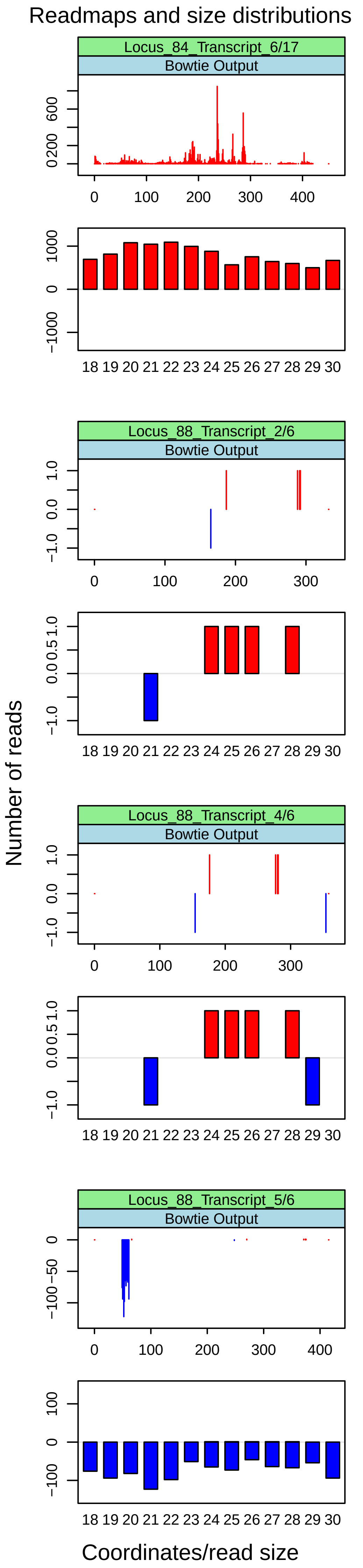
Readmaps and size distributions
Locus_84_Transcript_6/17
Bowtie Output
Locus_88_Transcript_2/6
Bowtie Output
Locus_88_Transcript_4/6
Bowtie Output
Locus_88_Transcript_5/6
Bowtie Output
Number of reads
Coordinates/read size

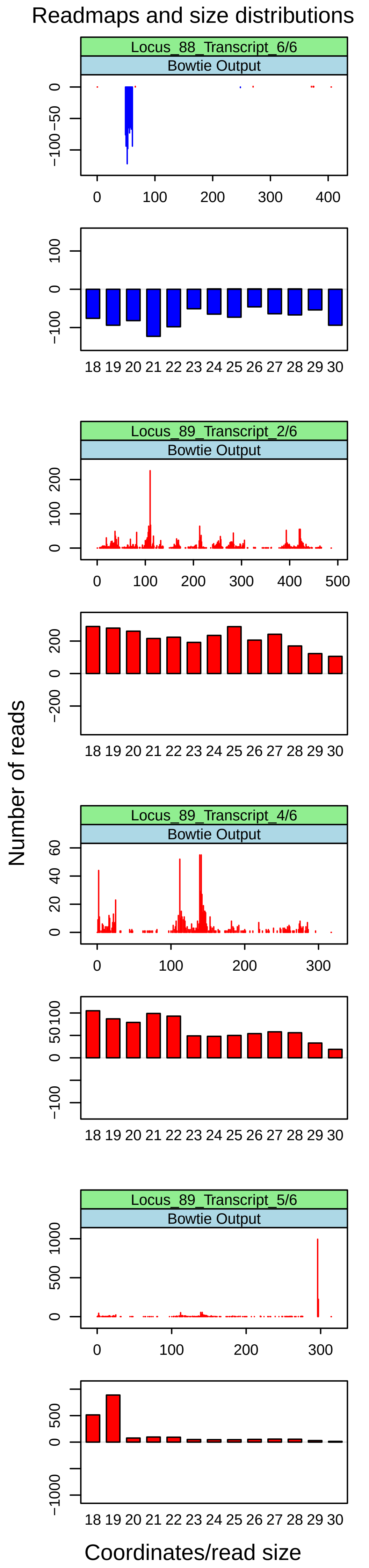

Readmaps and size distributions
Locus_88_Transcript_6/6
Bowtie Output
Locus_89_Transcript_2/6
Bowtie Output
Locus_89_Transcript_4/6
Bowtie Output
Locus_89_Transcript_5/6
Bowtie Output
Number of reads
Coordinates/read size
18 19 20 21 22 23 24 25 26 27 28 29 30

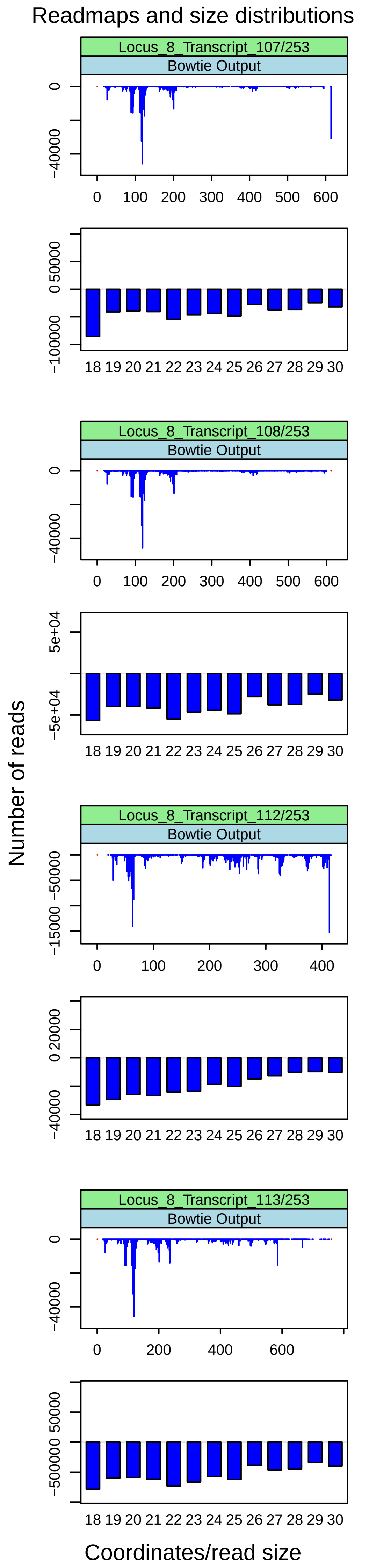

Readmaps and size distributions
Locus_8_Transcript_107/253
Bowtie Output
0
-40000
0 100 200 300 400 500 600
-100000 0 50000
18 19 20 21 22 23 24 25 26 27 28 29 30
Locus_8_Transcript_108/253
Bowtie Output
0
-40000
0 100 200 300 400 500 600
-5e+04 5e+04
18 19 20 21 22 23 24 25 26 27 28 29 30
Locus_8_Transcript_112/253
Bowtie Output
-15000 -50000
0 100 200 300 400
-40000 0 20000
18 19 20 21 22 23 24 25 26 27 28 29 30
Locus_8_Transcript_113/253
Bowtie Output
0
-40000
0 200 400 600
-500000 50000
18 19 20 21 22 23 24 25 26 27 28 29 30
Number of reads
Coordinates/read size

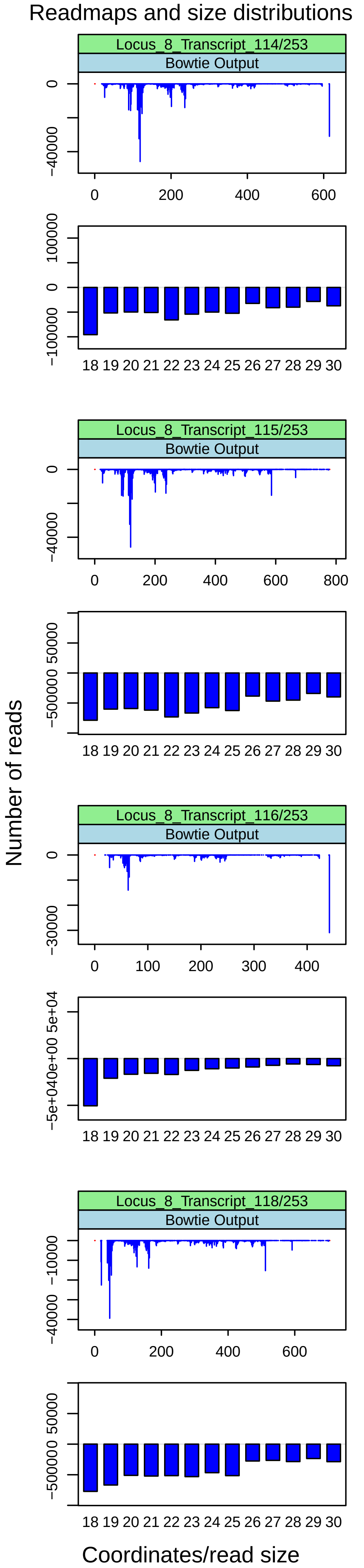

Readmaps and size distributions
Locus_8_Transcript_114/253
Bowtie Output
0
−40000
0 200 400 600
100000
0
−100000
18 19 20 21 22 23 24 25 26 27 28 29 30
Locus_8_Transcript_115/253
Bowtie Output
0
−40000
0 200 400 600 800
−500000 50000
18 19 20 21 22 23 24 25 26 27 28 29 30
Locus_8_Transcript_116/253
Bowtie Output
0
−30000
0 100 200 300 400
−5e+040e+00 5e+04
18 19 20 21 22 23 24 25 26 27 28 29 30
Locus_8_Transcript_118/253
Bowtie Output
0
−10000
−40000
0 200 400 600
−500000 50000
18 19 20 21 22 23 24 25 26 27 28 29 30
Number of reads
Coordinates/read size

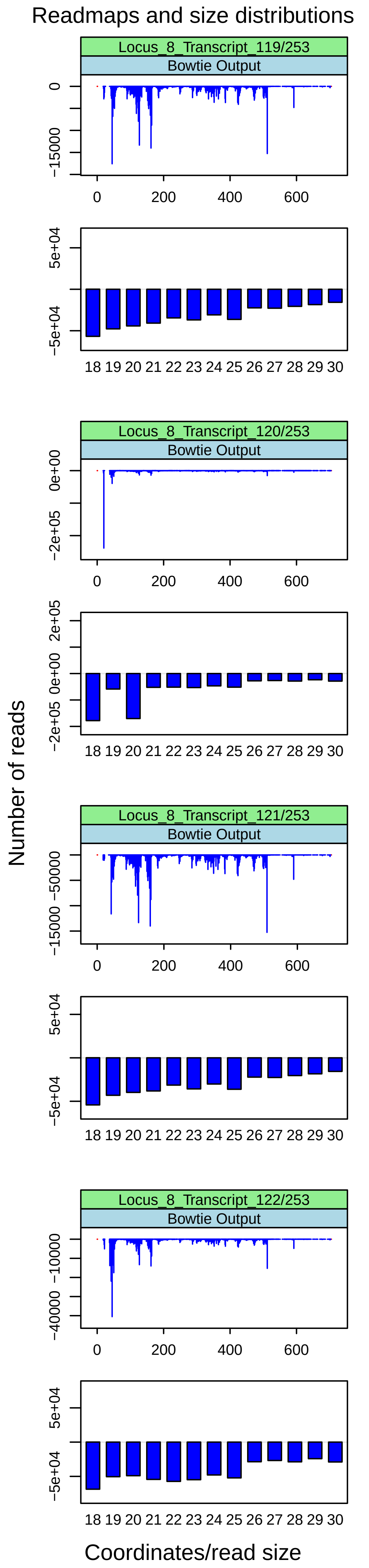

Readmaps and size distributions
Locus_8_Transcript_119/253
Bowtie Output
0
−15000
0
200
400
600
5e+04
−5e+04
18 19 20 21 22 23 24 25 26 27 28 29 30
Locus_8_Transcript_120/253
Bowtie Output
0e+00
−2e+05
0
200
400
600
2e+05
0e+00
−2e+05
18 19 20 21 22 23 24 25 26 27 28 29 30
Locus_8_Transcript_121/253
Bowtie Output
−50000
−150000
0
200
400
600
5e+04
−5e+04
18 19 20 21 22 23 24 25 26 27 28 29 30
Locus_8_Transcript_122/253
Bowtie Output
−10000
−40000
0
200
400
600
5e+04
−5e+04
18 19 20 21 22 23 24 25 26 27 28 29 30
Number of reads
Coordinates/read size

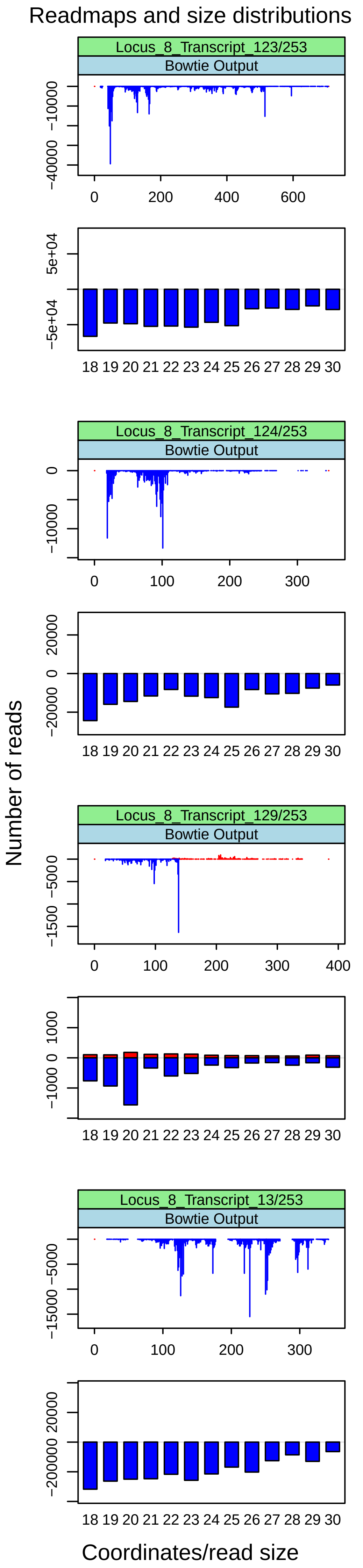

Readmaps and size distributions
Locus_8_Transcript_123/253
Bowtie Output
−40000
−10000
0
200
400
600
5e+04
−5e+04
18 19 20 21 22 23 24 25 26 27 28 29 30
Locus_8_Transcript_124/253
Bowtie Output
0
−10000
0
100
200
300
20000
0
−20000
18 19 20 21 22 23 24 25 26 27 28 29 30
Locus_8_Transcript_129/253
Bowtie Output
−1500
−5000
0
100
200
300
400
1000
0
−1000
18 19 20 21 22 23 24 25 26 27 28 29 30
Locus_8_Transcript_13/253
Bowtie Output
−15000
−5000
0
100
200
300
20000
0
−20000
18 19 20 21 22 23 24 25 26 27 28 29 30
Number of reads
Coordinates/read size

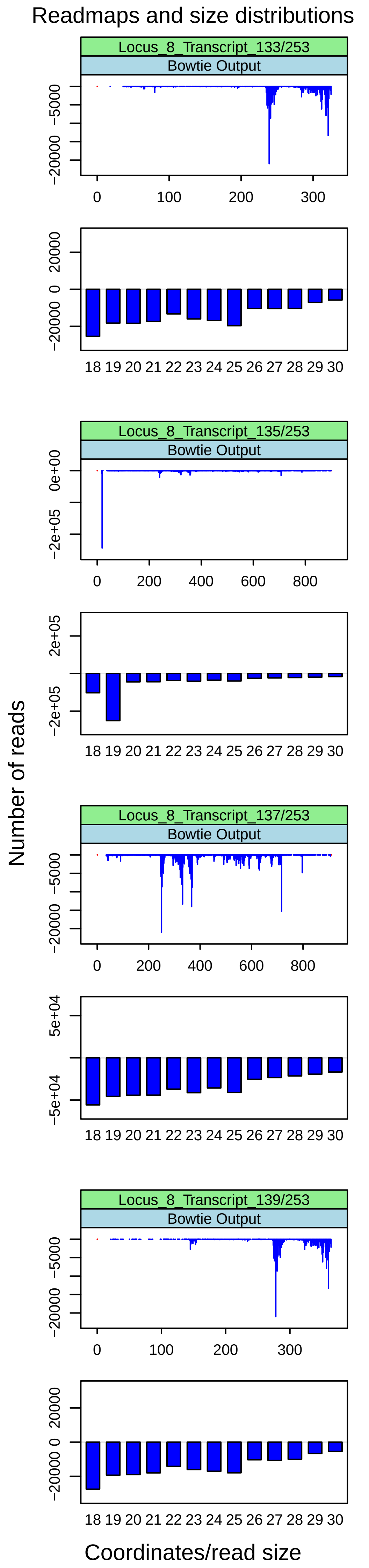

Readmaps and size distributions
Locus_8_Transcript_133/253
Bowtie Output
Locus_8_Transcript_135/253
Bowtie Output
Locus_8_Transcript_137/253
Bowtie Output
Locus_8_Transcript_139/253
Bowtie Output
Number of reads
Coordinates/read size

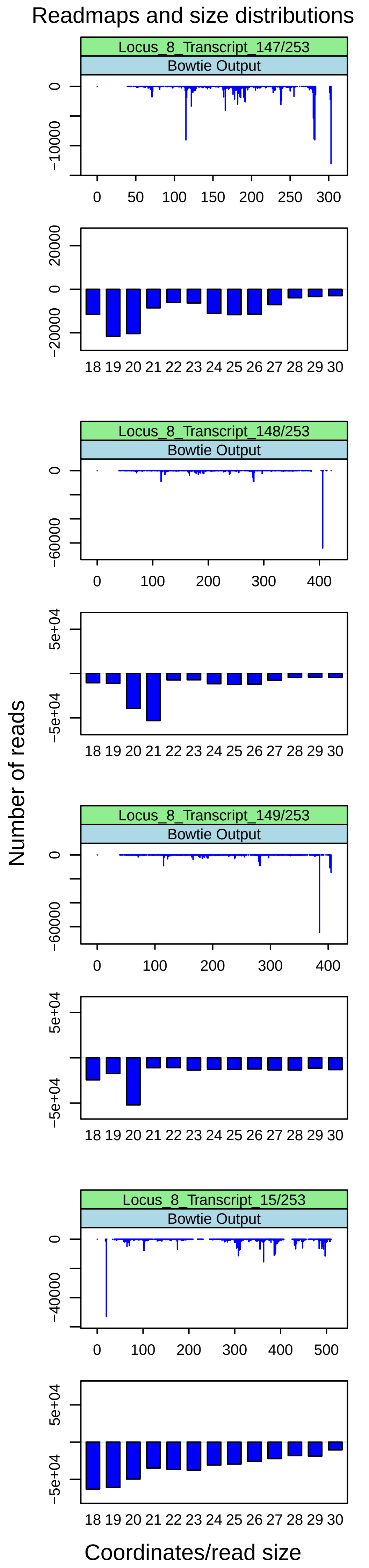
Readmaps and size distributions
Locus_8_Transcript_147/253
Bowtie Output
0
−10000
0 50 100 150 200 250 300
20000
0
−20000
18 19 20 21 22 23 24 25 26 27 28 29 30
Locus_8_Transcript_148/253
Bowtie Output
0
−60000
0 100 200 300 400
5e+04
−5e+04
18 19 20 21 22 23 24 25 26 27 28 29 30
Locus_8_Transcript_149/253
Bowtie Output
0
−60000
0 100 200 300 400
5e+04
−5e+04
18 19 20 21 22 23 24 25 26 27 28 29 30
Locus_8_Transcript_15/253
Bowtie Output
0
−40000
0 100 200 300 400 500
5e+04
−5e+04
18 19 20 21 22 23 24 25 26 27 28 29 30
Number of reads
Coordinates/read size

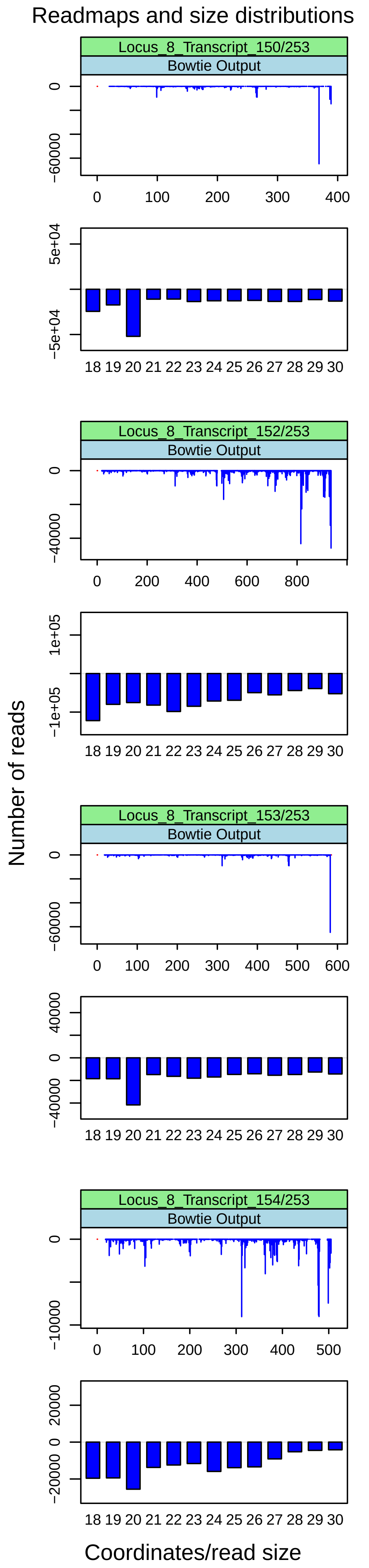

Readmaps and size distributions
Locus_8_Transcript_150/253
Bowtie Output
0
-60000
0 100 200 300 400
5e+04
-5e+04
18 19 20 21 22 23 24 25 26 27 28 29 30
Locus_8_Transcript_152/253
Bowtie Output
0
-40000
0 200 400 600 800
1e+05
-1e+05
18 19 20 21 22 23 24 25 26 27 28 29 30
Locus_8_Transcript_153/253
Bowtie Output
0
-60000
0 100 200 300 400 500 600
40000
0
-40000
18 19 20 21 22 23 24 25 26 27 28 29 30
Locus_8_Transcript_154/253
Bowtie Output
0
-10000
0 100 200 300 400 500
20000
0
-20000
18 19 20 21 22 23 24 25 26 27 28 29 30
Number of reads
Coordinates/read size

Readmaps and size distributions

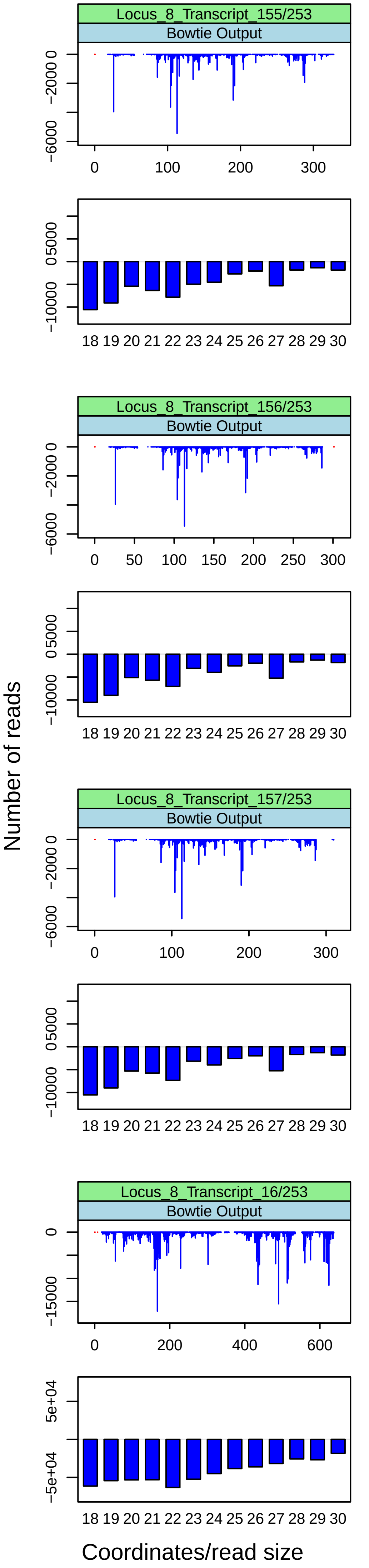

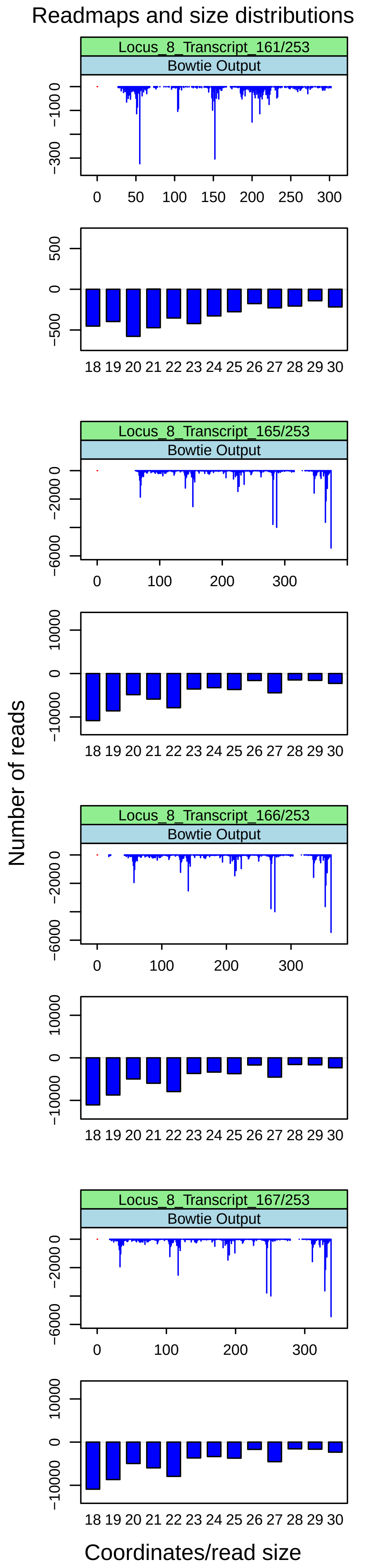

Readmaps and size distributions
Locus_8_Transcript_161/253
Bowtie Output
Locus_8_Transcript_165/253
Bowtie Output
Locus_8_Transcript_166/253
Bowtie Output
Locus_8_Transcript_167/253
Bowtie Output
Number of reads
Coordinates/read size

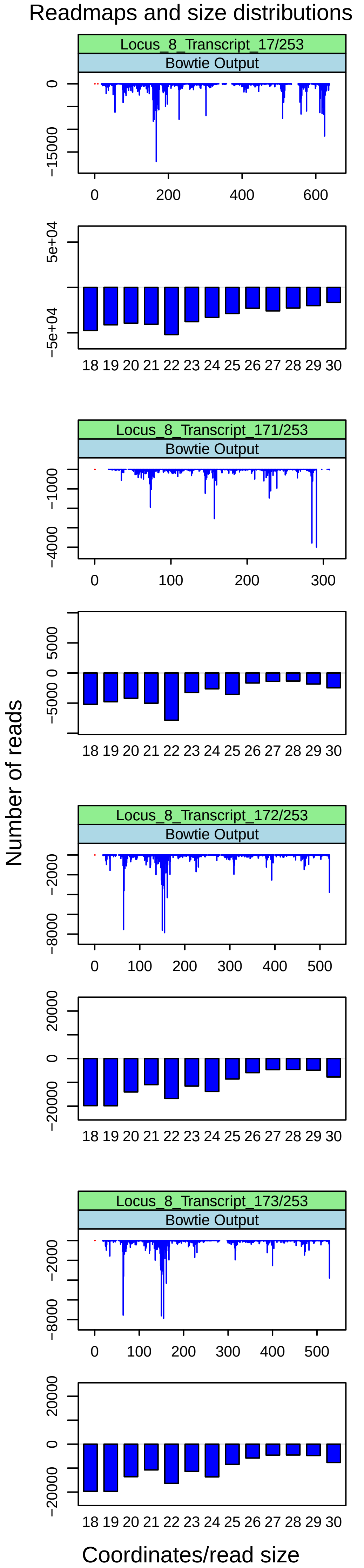
Readmaps and size distributions
Locus_8_Transcript_17/253
Bowtie Output
0
−15000
0
200
400
600
5e+04
−5e+04
18 19 20 21 22 23 24 25 26 27 28 29 30
Locus_8_Transcript_171/253
Bowtie Output
−1000
−4000
0
100
200
300
5000
−5000 0
18 19 20 21 22 23 24 25 26 27 28 29 30
Locus_8_Transcript_172/253
Bowtie Output
−2000
−8000
0
100
200
300
400
500
20000
0
−20000
18 19 20 21 22 23 24 25 26 27 28 29 30
Locus_8_Transcript_173/253
Bowtie Output
−2000
−8000
0
100
200
300
400
500
20000
0
−20000
18 19 20 21 22 23 24 25 26 27 28 29 30
Number of reads
Coordinates/read size

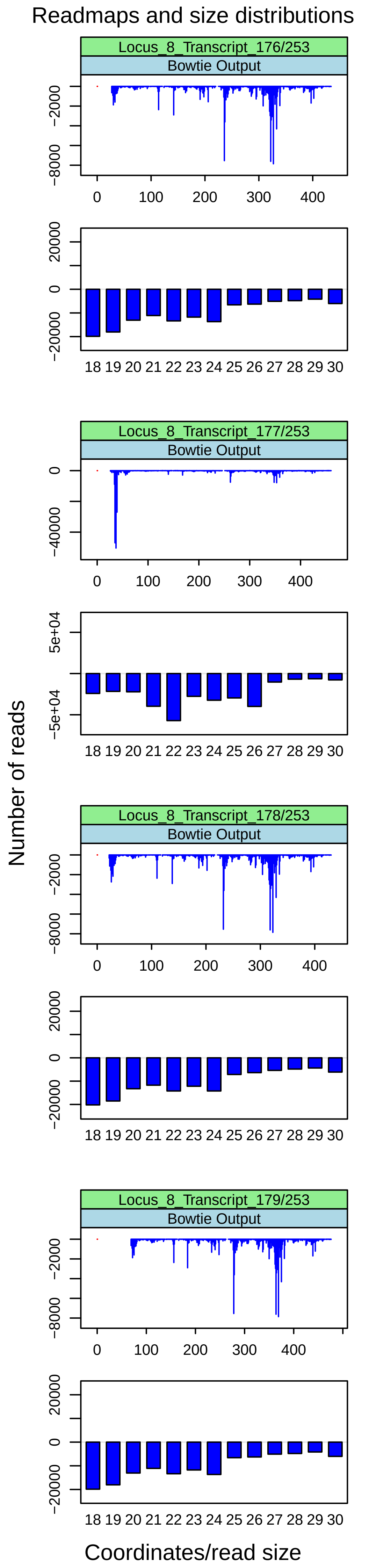

Readmaps and size distributions
Locus_8_Transcript_176/253
Bowtie Output
Locus_8_Transcript_177/253
Bowtie Output
Locus_8_Transcript_178/253
Bowtie Output
Locus_8_Transcript_179/253
Bowtie Output
Number of reads
Coordinates/read size

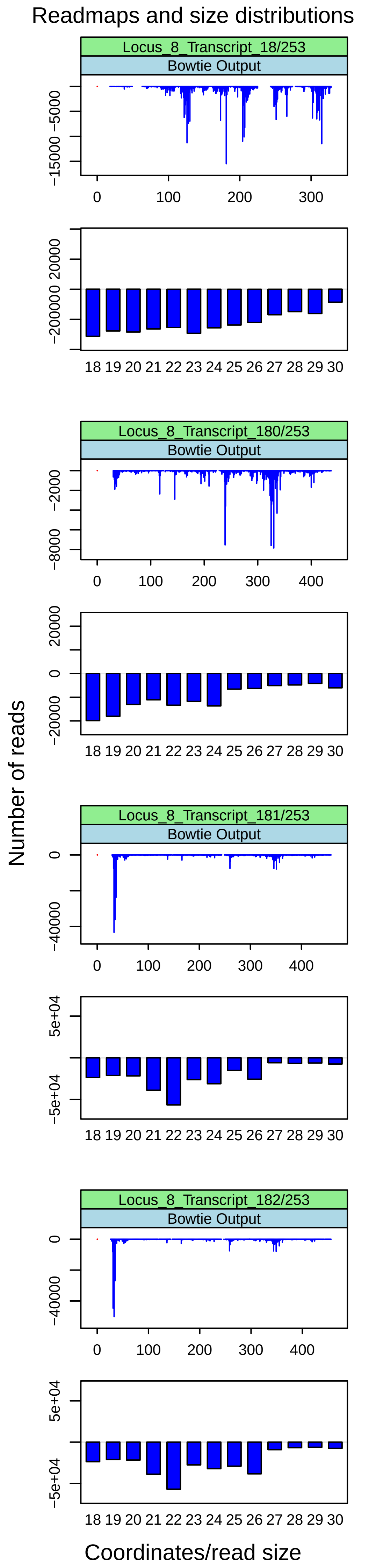

Readmaps and size distributions
Locus_8_Transcript_18/253
Bowtie Output
Locus_8_Transcript_180/253
Bowtie Output
Locus_8_Transcript_181/253
Bowtie Output
Locus_8_Transcript_182/253
Bowtie Output
Number of reads
Coordinates/read size

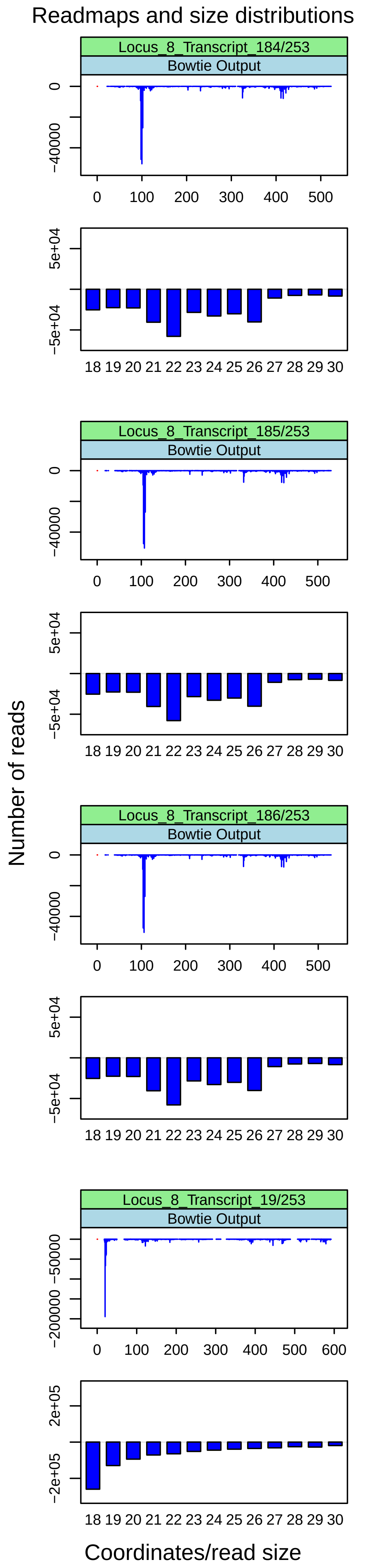

Readmaps and size distributions
Locus_8_Transcript_184/253
Bowtie Output
0
−40000
0 100 200 300 400 500
5e+04
−5e+04
18 19 20 21 22 23 24 25 26 27 28 29 30
Locus_8_Transcript_185/253
Bowtie Output
0
−40000
0 100 200 300 400 500
5e+04
−5e+04
18 19 20 21 22 23 24 25 26 27 28 29 30
Locus_8_Transcript_186/253
Bowtie Output
0
−40000
0 100 200 300 400 500
5e+04
−5e+04
18 19 20 21 22 23 24 25 26 27 28 29 30
Locus_8_Transcript_19/253
Bowtie Output
−50000
−200000
0 100 200 300 400 500 600
2e+05
−2e+05
18 19 20 21 22 23 24 25 26 27 28 29 30
Number of reads
Coordinates/read size

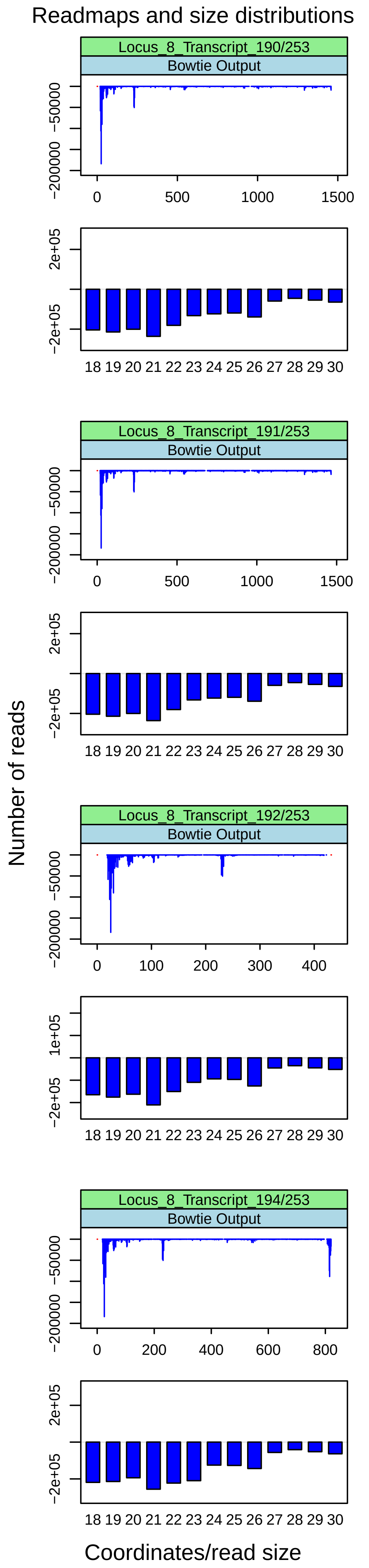
Readmaps and size distributions
Locus_8_Transcript_190/253
Bowtie Output
-200000
-50000
0
500
1000
1500
2e+05
-2e+05
18 19 20 21 22 23 24 25 26 27 28 29 30
Locus_8_Transcript_191/253
Bowtie Output
-200000
-50000
0
500
1000
1500
2e+05
-2e+05
18 19 20 21 22 23 24 25 26 27 28 29 30
Locus_8_Transcript_192/253
Bowtie Output
-200000
-50000
0
100
200
300
400
1e+05
-2e+05
18 19 20 21 22 23 24 25 26 27 28 29 30
Locus_8_Transcript_194/253
Bowtie Output
-200000
-50000
0
200
400
600
800
2e+05
-2e+05
18 19 20 21 22 23 24 25 26 27 28 29 30
Number of reads
Coordinates/read size

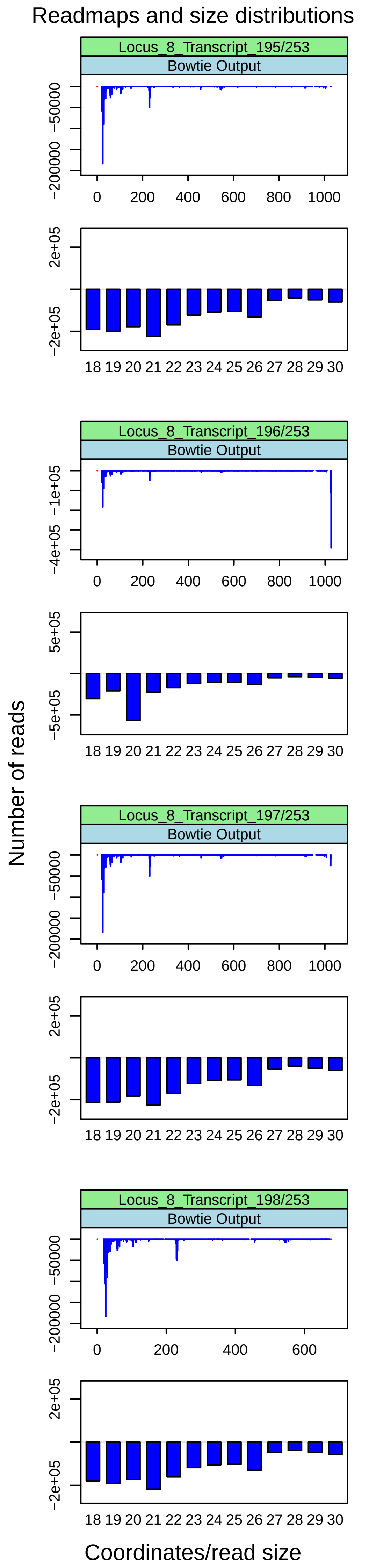
Readmaps and size distributions
Locus_8_Transcript_195/253
Bowtie Output
Locus_8_Transcript_196/253
Bowtie Output
Locus_8_Transcript_197/253
Bowtie Output
Locus_8_Transcript_198/253
Bowtie Output
Number of reads
Coordinates/read size

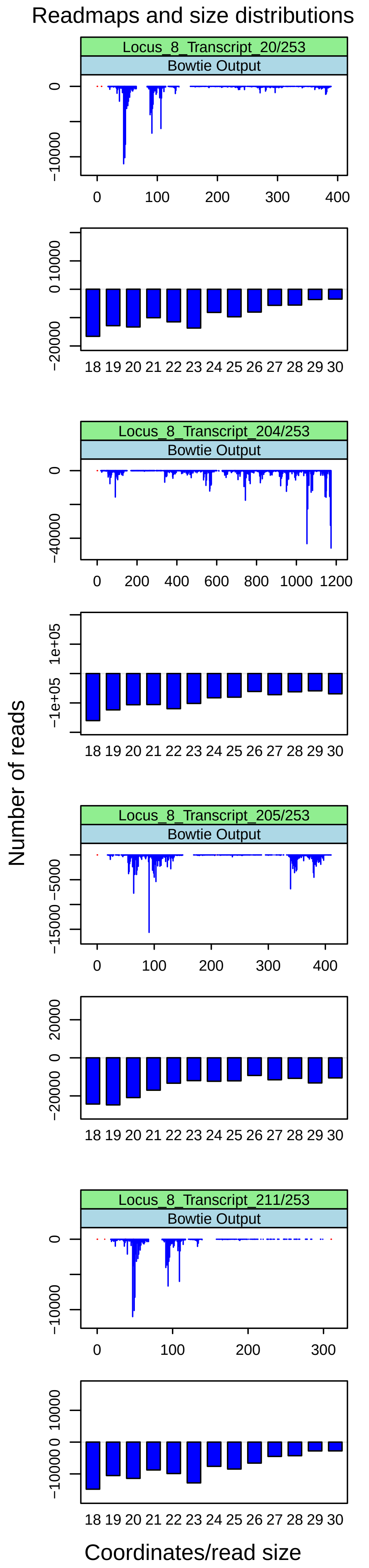
Readmaps and size distributions
Locus_8_Transcript_20/253
Bowtie Output
Locus_8_Transcript_204/253
Bowtie Output
Locus_8_Transcript_205/253
Bowtie Output
Locus_8_Transcript_211/253
Bowtie Output
Number of reads
Coordinates/read size
18 19 20 21 22 23 24 25 26 27 28 29 30

Readmaps and size distributions

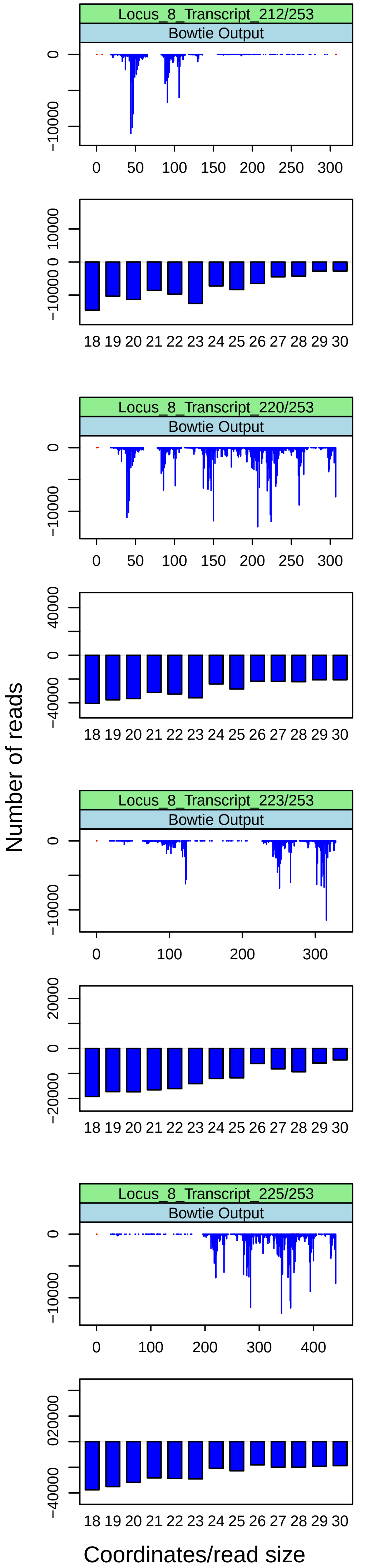

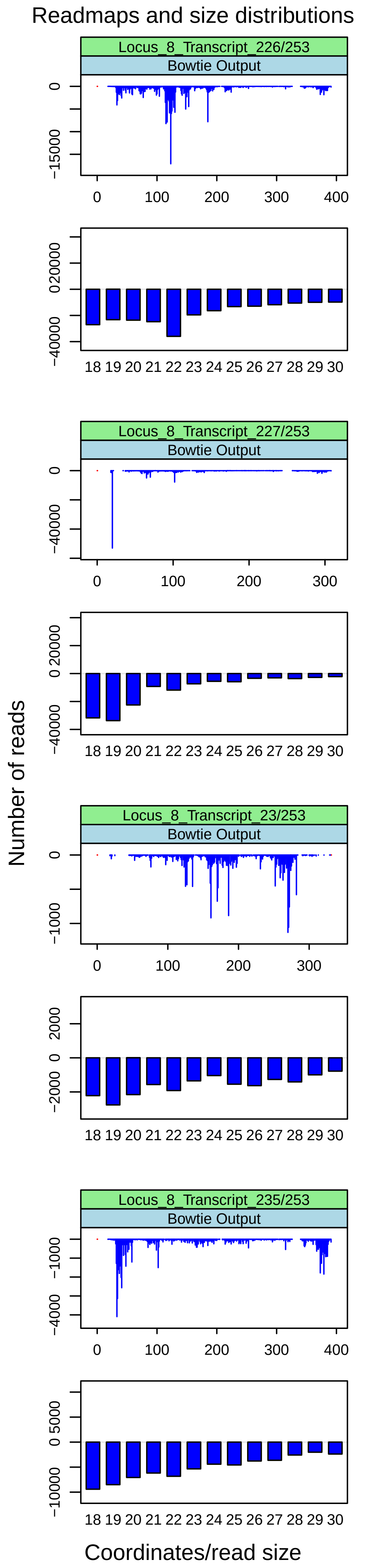
Readmaps and size distributions
Locus_8_Transcript_226/253
Bowtie Output
0
-15000
0 100 200 300 400
0 20000
-40000
18 19 20 21 22 23 24 25 26 27 28 29 30
Locus_8_Transcript_227/253
Bowtie Output
0
-40000
0 100 200 300
0 20000
-40000
18 19 20 21 22 23 24 25 26 27 28 29 30
Locus_8_Transcript_23/253
Bowtie Output
0
-1000
0 100 200 300
2000
0
-2000
18 19 20 21 22 23 24 25 26 27 28 29 30
Locus_8_Transcript_235/253
Bowtie Output
-1000
-4000
0 100 200 300 400
0 5000
-10000
18 19 20 21 22 23 24 25 26 27 28 29 30
Number of reads
Coordinates/read size

Readmaps and size distributions

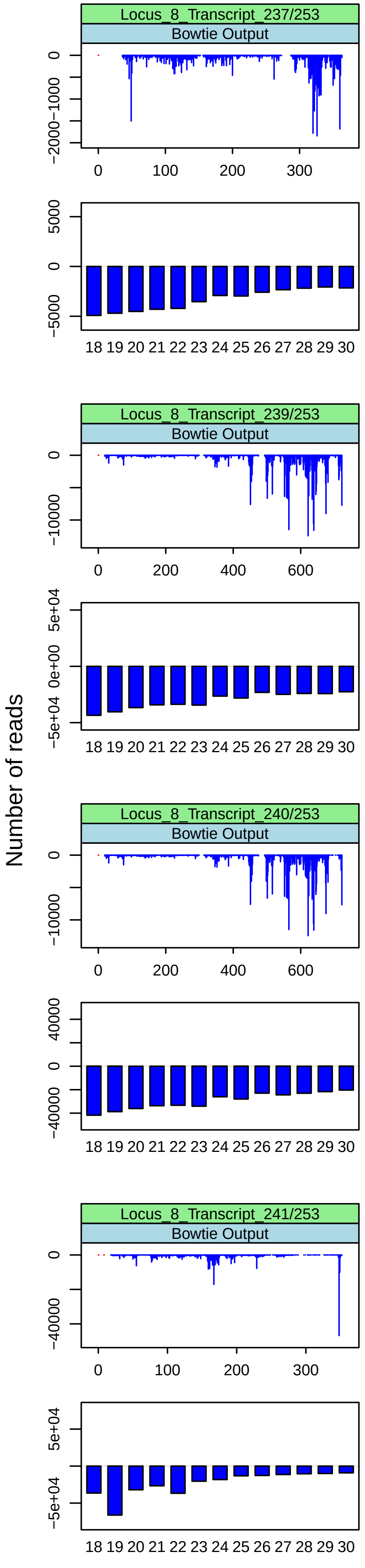

Coordinates/read size

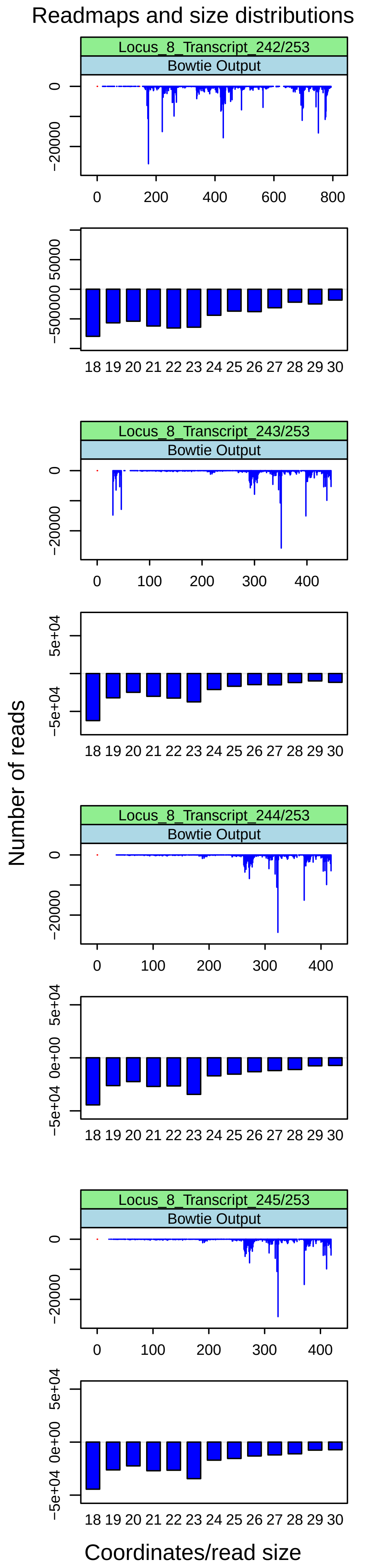
Readmaps and size distributions
Locus_8_Transcript_242/253
Bowtie Output
Locus_8_Transcript_243/253
Bowtie Output
Locus_8_Transcript_244/253
Bowtie Output
Locus_8_Transcript_245/253
Bowtie Output
Number of reads
Coordinates/read size

Readmaps and size distributions

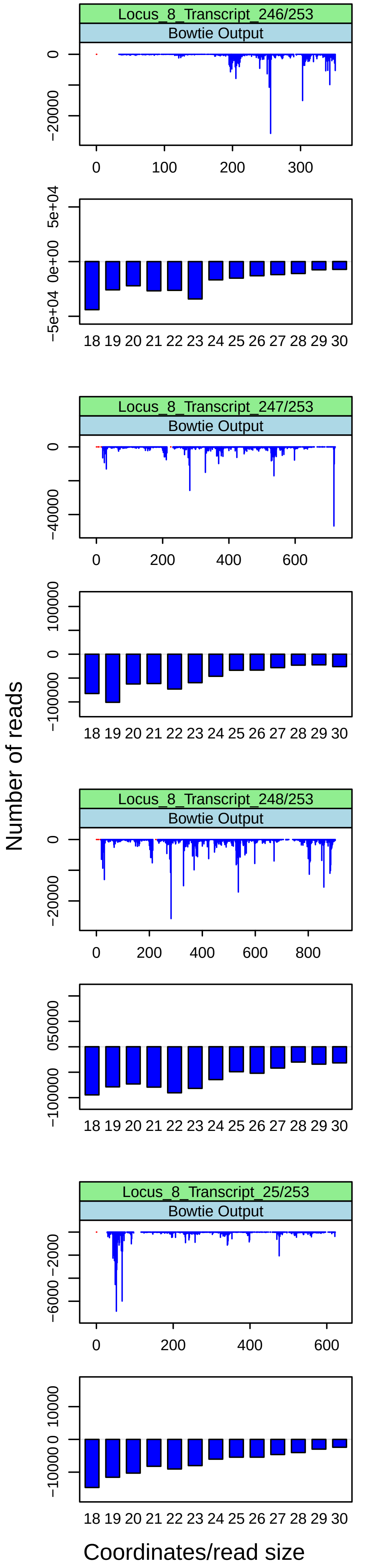

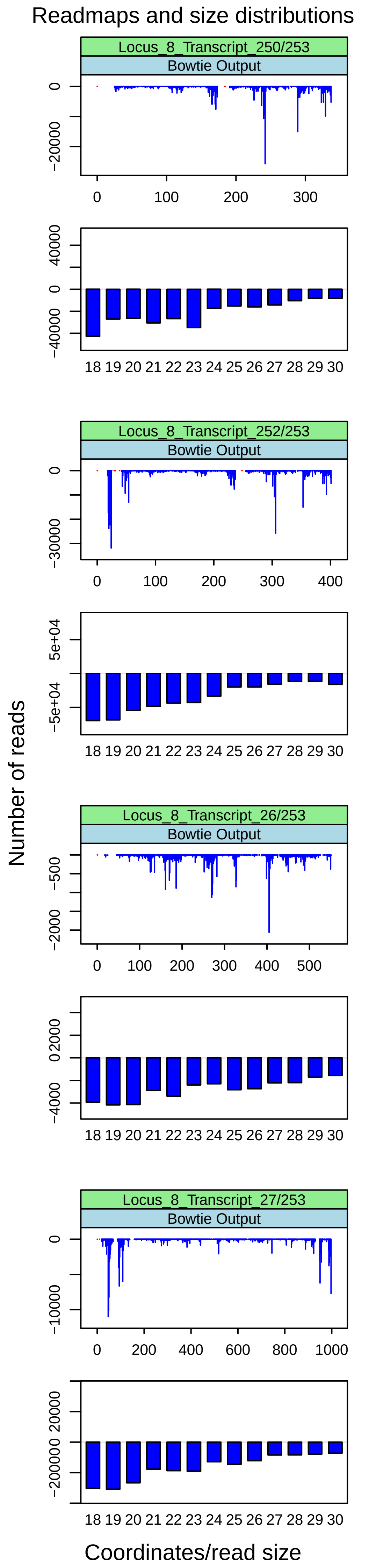

Readmaps and size distributions
Locus_8_Transcript_250/253
Bowtie Output
0
-20000
0 100 200 300
40000
0
-40000
18 19 20 21 22 23 24 25 26 27 28 29 30
Locus_8_Transcript_252/253
Bowtie Output
0
-30000
0 100 200 300 400
5e+04
-5e+04
18 19 20 21 22 23 24 25 26 27 28 29 30
Locus_8_Transcript_26/253
Bowtie Output
-500
-2000
0 100 200 300 400 500
02000
-4000
18 19 20 21 22 23 24 25 26 27 28 29 30
Locus_8_Transcript_27/253
Bowtie Output
0
-10000
0 200 400 600 800 1000
200000 20000
-200000
18 19 20 21 22 23 24 25 26 27 28 29 30
Number of reads
Coordinates/read size

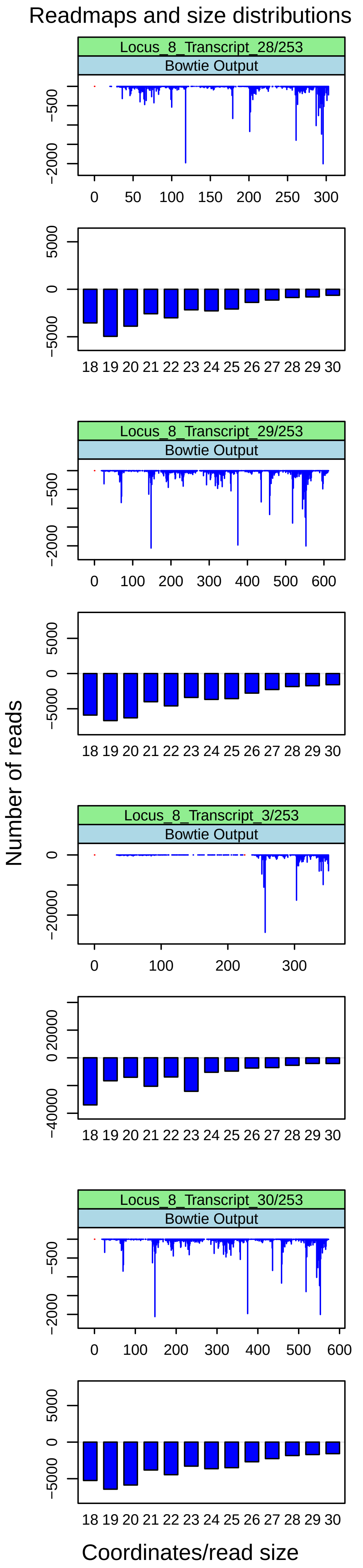
Readmaps and size distributions
Locus_8_Transcript_28/253
Bowtie Output
-500
-2000
0 50 100 150 200 250 300
5000
0
-5000
18 19 20 21 22 23 24 25 26 27 28 29 30
Locus_8_Transcript_29/253
Bowtie Output
-500
-2000
0 100 200 300 400 500 600
5000
0
-5000
18 19 20 21 22 23 24 25 26 27 28 29 30
Locus_8_Transcript_3/253
Bowtie Output
0
-20000
0 100 200 300
0 20000
-40000
18 19 20 21 22 23 24 25 26 27 28 29 30
Locus_8_Transcript_30/253
Bowtie Output
-500
-2000
0 100 200 300 400 500 600
5000
0
-5000
18 19 20 21 22 23 24 25 26 27 28 29 30
Number of reads
Coordinates/read size

Readmaps and size distributions

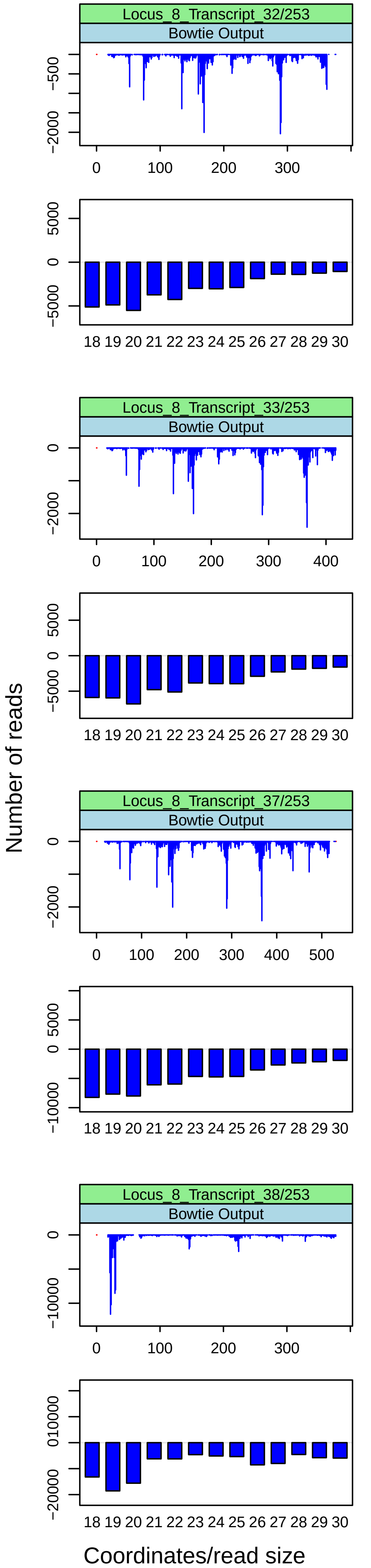

Readmaps and size distributions

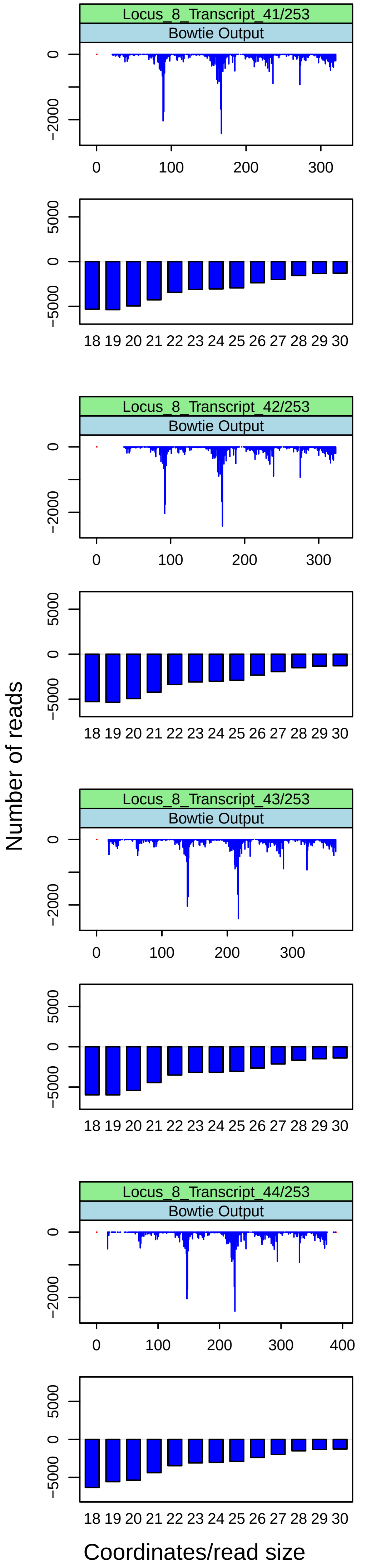

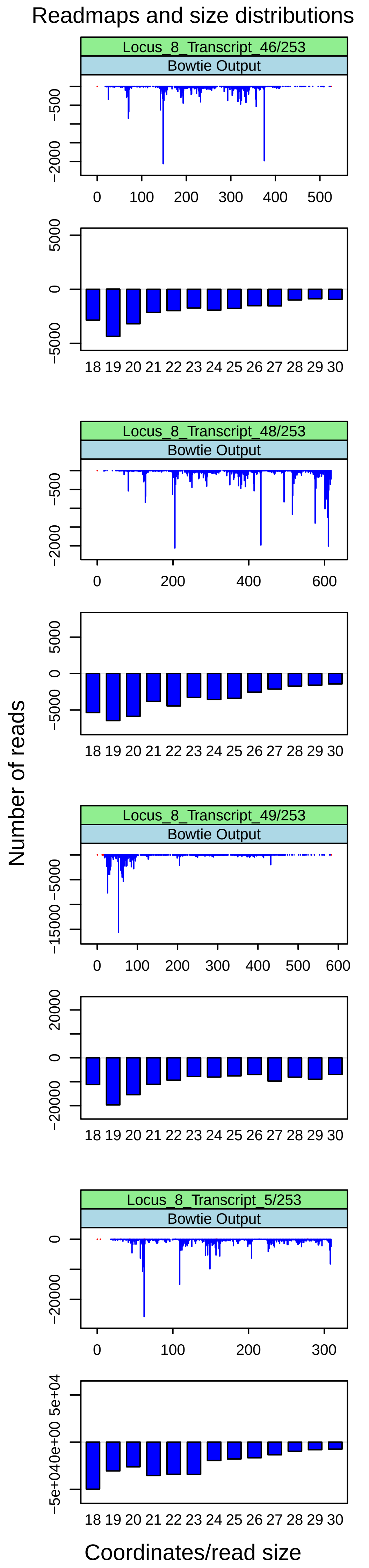
Readmaps and size distributions
Locus_8_Transcript_46/253
Bowtie Output
Locus_8_Transcript_48/253
Bowtie Output
Locus_8_Transcript_49/253
Bowtie Output
Locus_8_Transcript_5/253
Bowtie Output
18 19 20 21 22 23 24 25 26 27 28 29 30
Number of reads
Coordinates/read size

Readmaps and size distributions

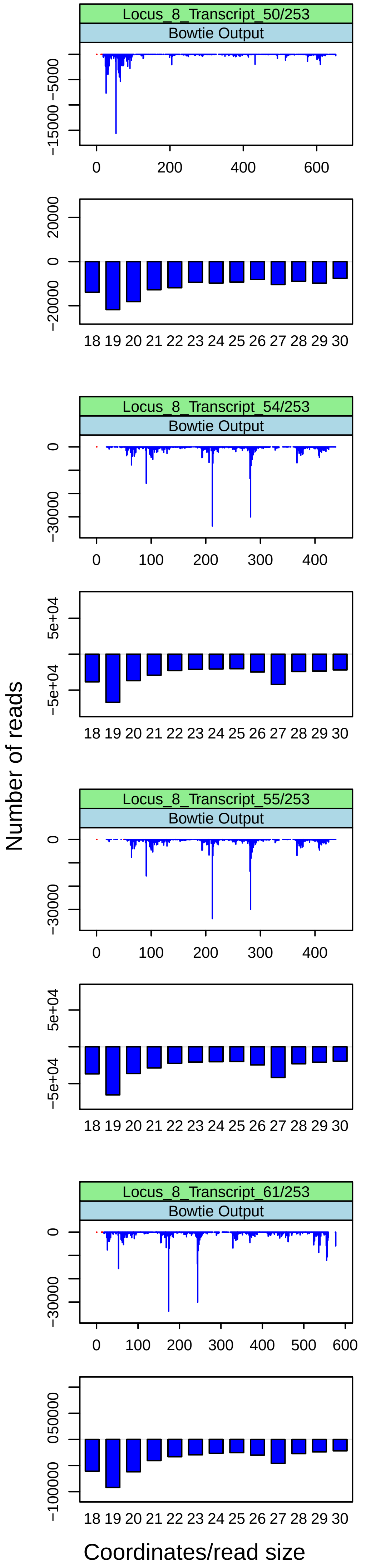

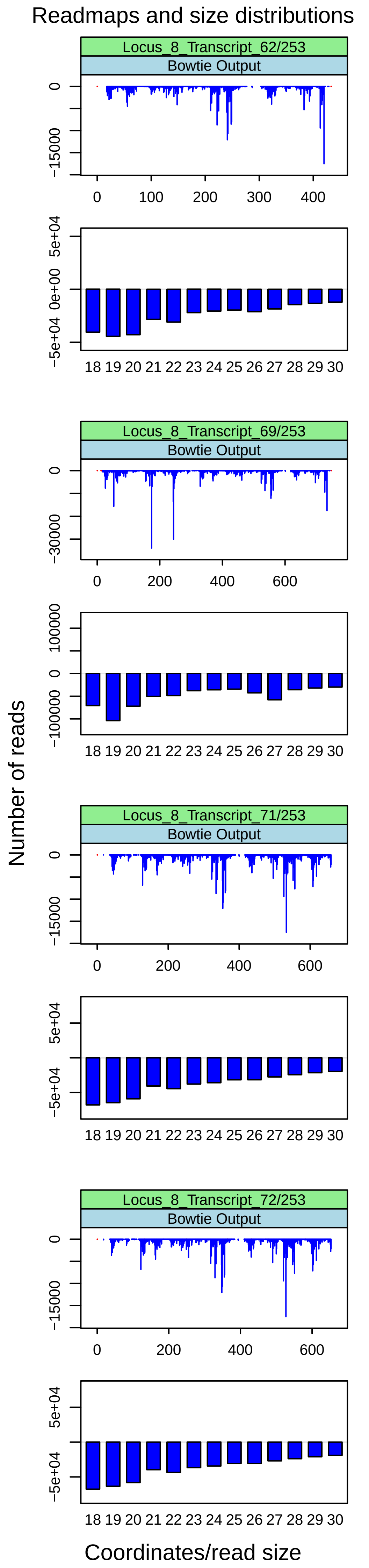
Readmaps and size distributions
Locus_8_Transcript_62/253
Bowtie Output
0
−15000
0
100
200
300
400
5e+04
0e+00
−5e+04
18 19 20 21 22 23 24 25 26 27 28 29 30
Locus_8_Transcript_69/253
Bowtie Output
0
−30000
0
200
400
600
100000
0
−100000
18 19 20 21 22 23 24 25 26 27 28 29 30
Locus_8_Transcript_71/253
Bowtie Output
0
−15000
0
200
400
600
5e+04
−5e+04
18 19 20 21 22 23 24 25 26 27 28 29 30
Locus_8_Transcript_72/253
Bowtie Output
0
−15000
0
200
400
600
5e+04
−5e+04
18 19 20 21 22 23 24 25 26 27 28 29 30
Number of reads
Coordinates/read size

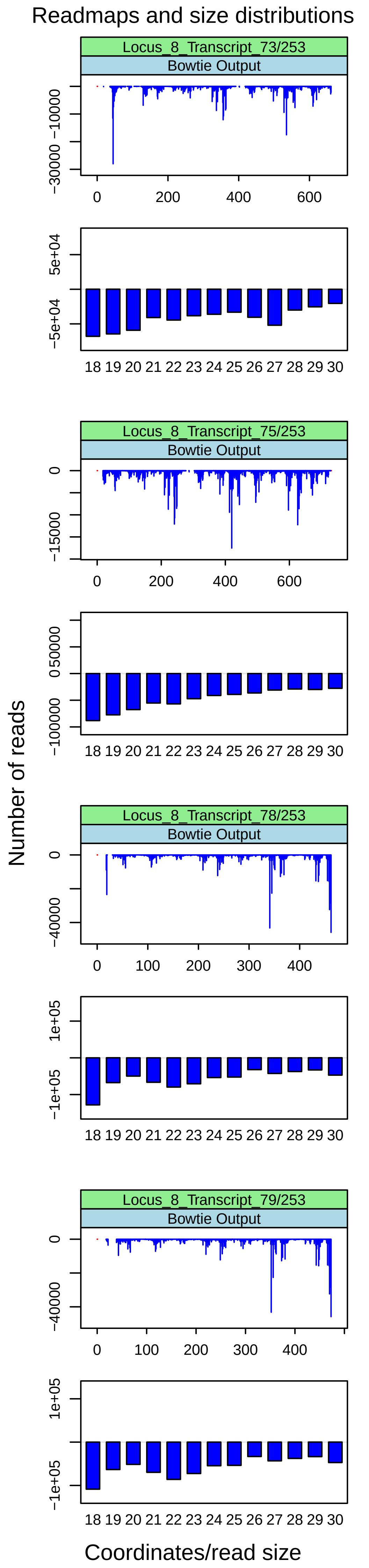
Readmaps and size distributions
Locus_8_Transcript_73/253
Bowtie Output
Locus_8_Transcript_75/253
Bowtie Output
Locus_8_Transcript_78/253
Bowtie Output
Locus_8_Transcript_79/253
Bowtie Output
Number of reads
Coordinates/read size
18 19 20 21 22 23 24 25 26 27 28 29 30

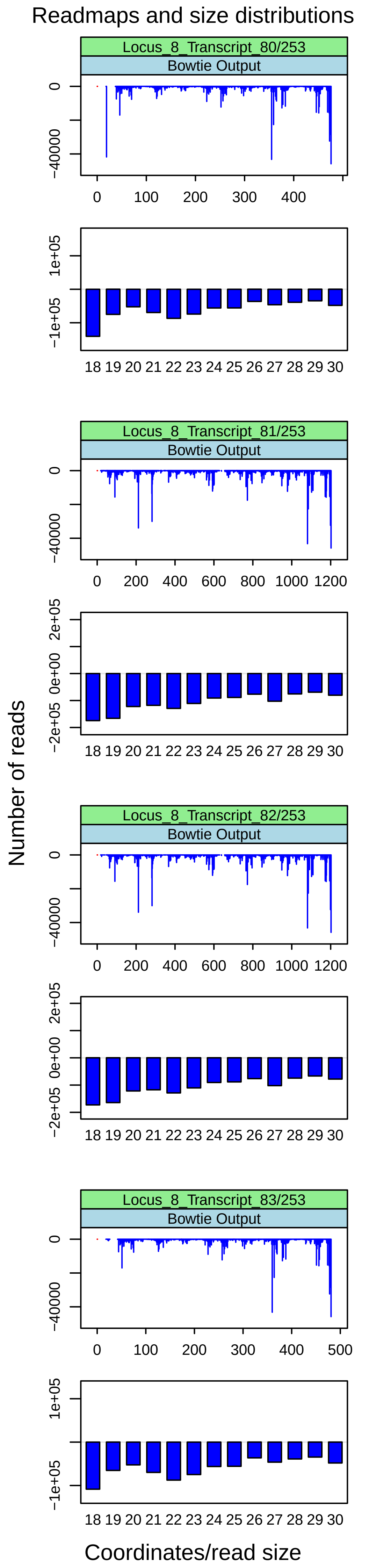
Readmaps and size distributions
Locus_8_Transcript_80/253
Bowtie Output
Locus_8_Transcript_81/253
Bowtie Output
Locus_8_Transcript_82/253
Bowtie Output
Locus_8_Transcript_83/253
Bowtie Output
Number of reads
Coordinates/read size

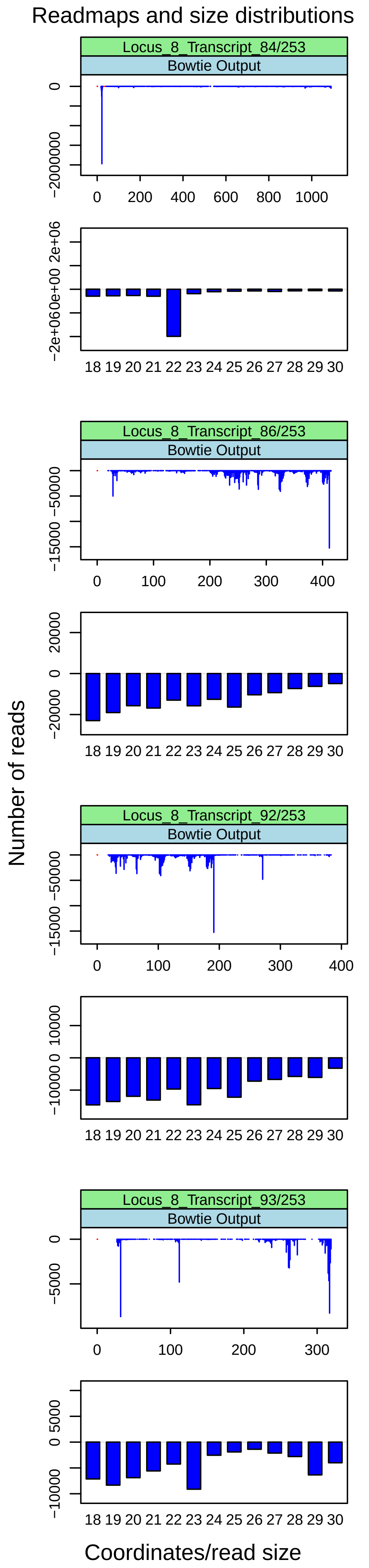
Readmaps and size distributions
Locus_8_Transcript_84/253
Bowtie Output
0
−2000000
0
200
400
600
800
1000
2e+06
0e+00
−2e+06
18 19 20 21 22 23 24 25 26 27 28 29 30
Locus_8_Transcript_86/253
Bowtie Output
0
−50000
−150000
0
100
200
300
400
20000
0
−20000
18 19 20 21 22 23 24 25 26 27 28 29 30
Locus_8_Transcript_92/253
Bowtie Output
0
−50000
−150000
0
100
200
300
400
10000
0
−10000
18 19 20 21 22 23 24 25 26 27 28 29 30
Locus_8_Transcript_93/253
Bowtie Output
0
−5000
0
100
200
300
5000
0
−10000
18 19 20 21 22 23 24 25 26 27 28 29 30
Number of reads
Coordinates/read size

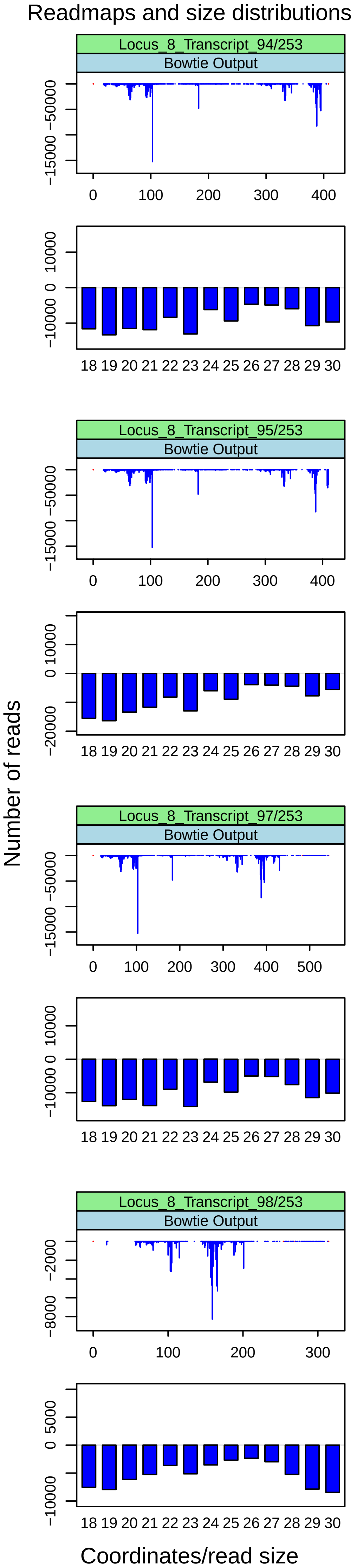

Readmaps and size distributions
Locus_8_Transcript_94/253
Bowtie Output
Locus_8_Transcript_95/253
Bowtie Output
Locus_8_Transcript_97/253
Bowtie Output
Locus_8_Transcript_98/253
Bowtie Output
Number of reads
Coordinates/read size

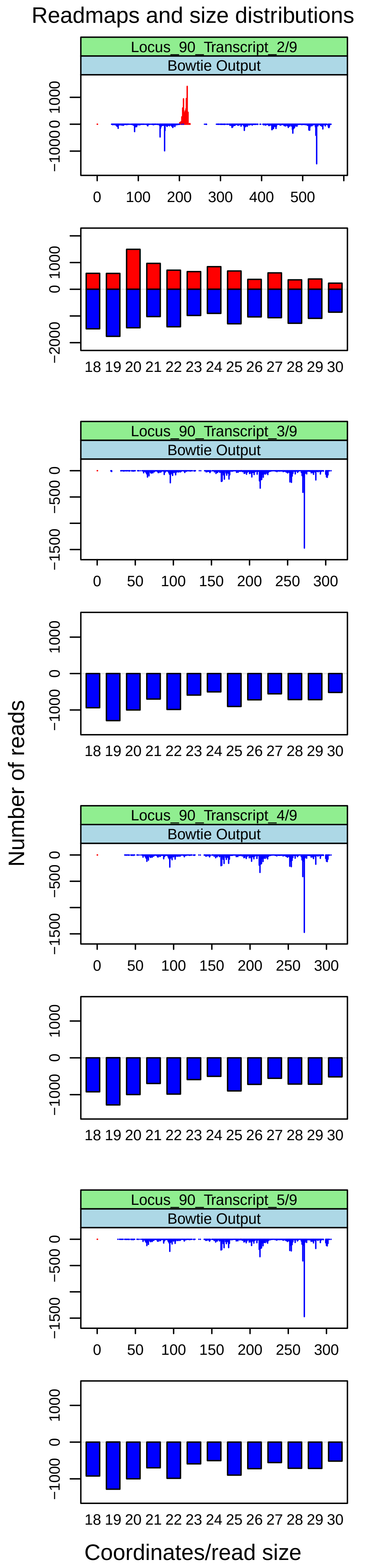

Readmaps and size distributions
Locus_90_Transcript_2/9
Bowtie Output
Locus_90_Transcript_3/9
Bowtie Output
Locus_90_Transcript_4/9
Bowtie Output
Locus_90_Transcript_5/9
Bowtie Output
Number of reads
Coordinates/read size

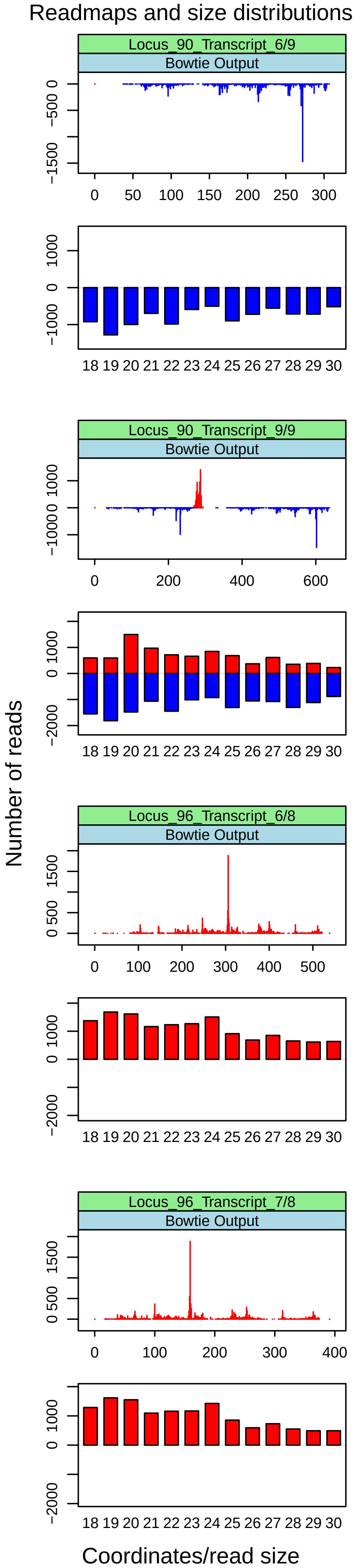

Readmaps and size distributions
Locus_90_Transcript_6/9
Bowtie Output
Locus_90_Transcript_9/9
Bowtie Output
Locus_96_Transcript_6/8
Bowtie Output
Locus_96_Transcript_7/8
Bowtie Output
Number of reads
Coordinates/read size

Readmaps and size distributions

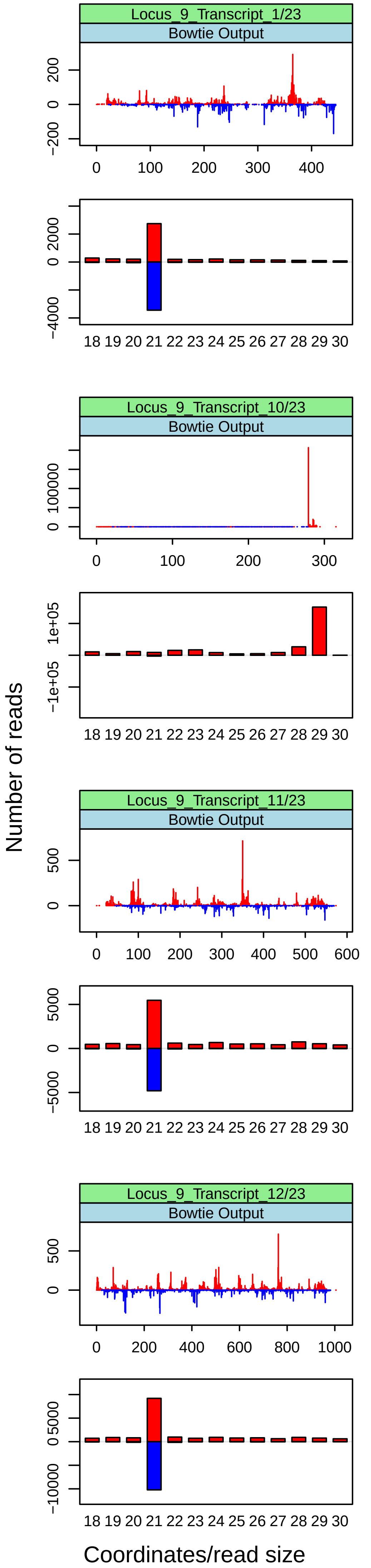

Readmaps and size distributions

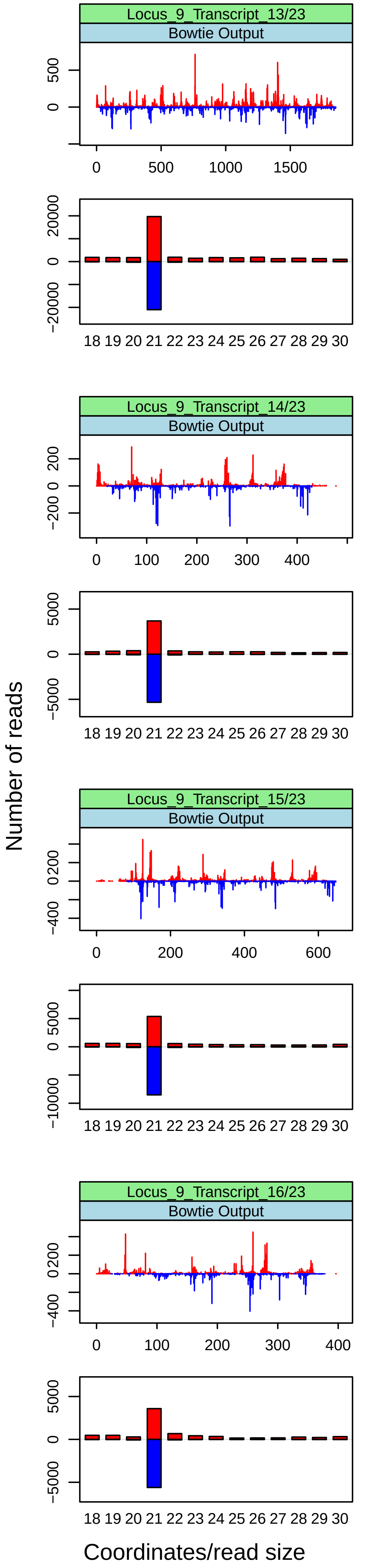

Readmaps and size distributions

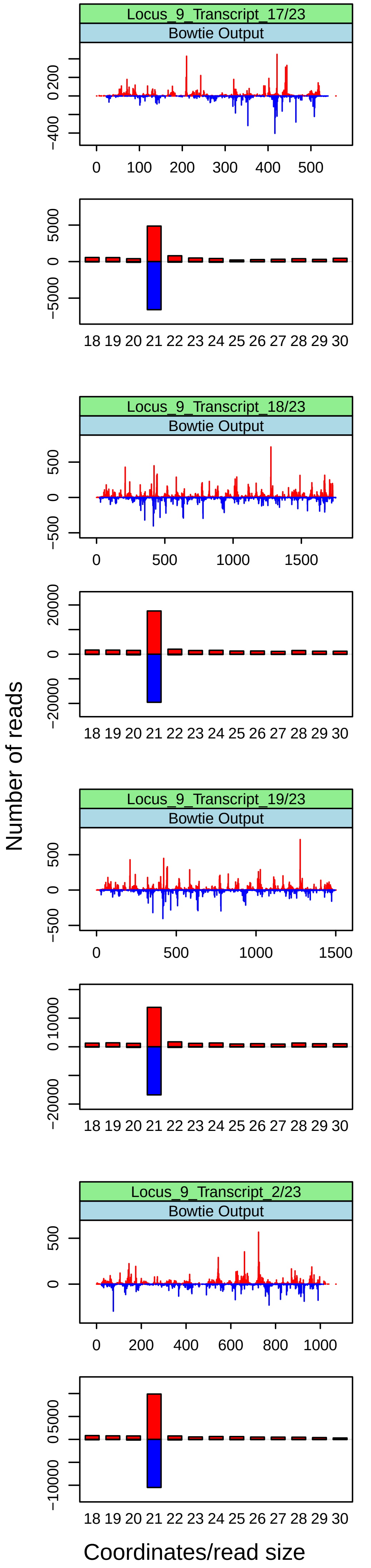

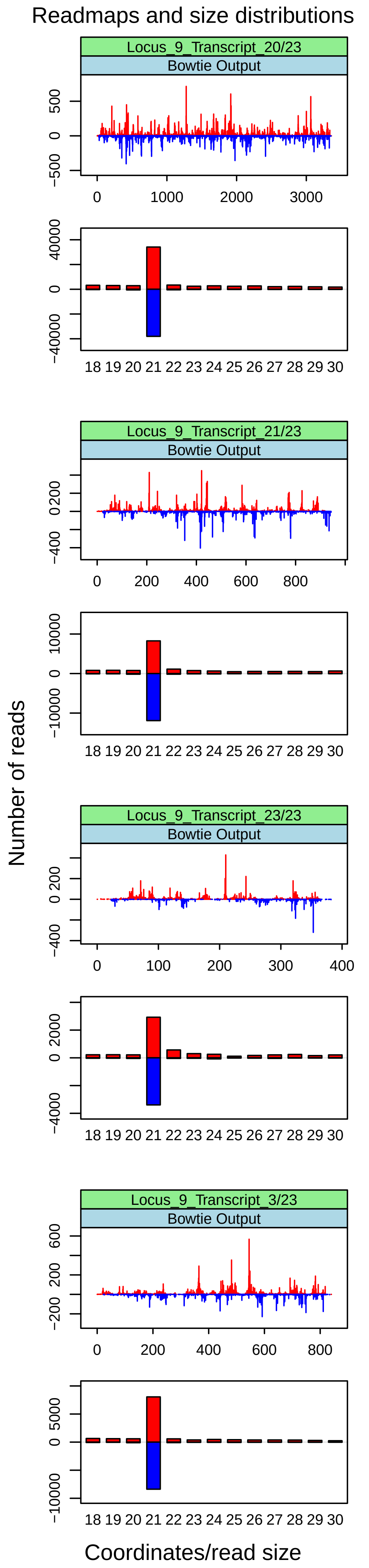

Readmaps and size distributions
Locus_9_Transcript_20/23
Bowtie Output
Locus_9_Transcript_21/23
Bowtie Output
Locus_9_Transcript_23/23
Bowtie Output
Locus_9_Transcript_3/23
Bowtie Output
Number of reads
Coordinates/read size

Readmaps and size distributions

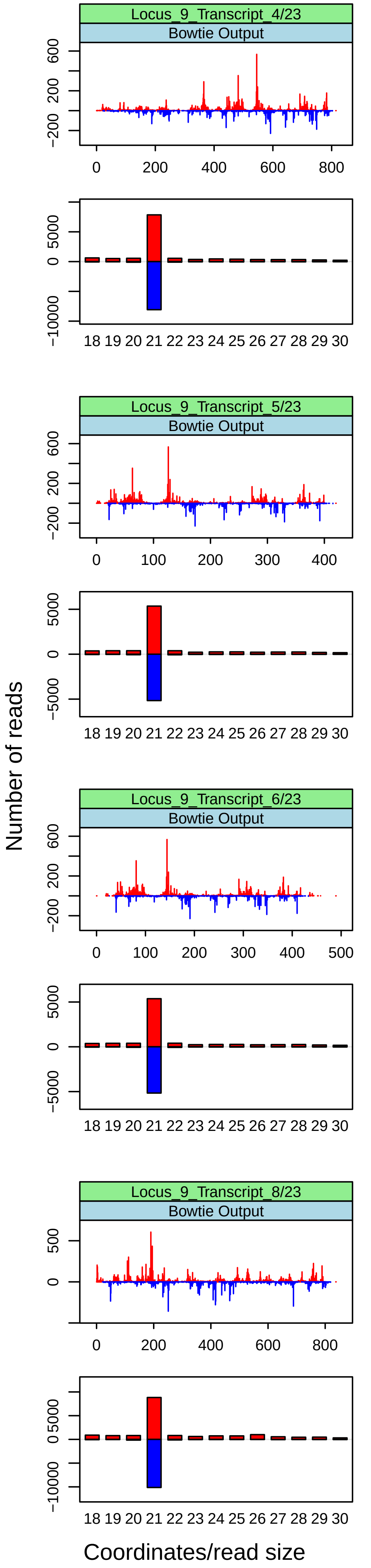

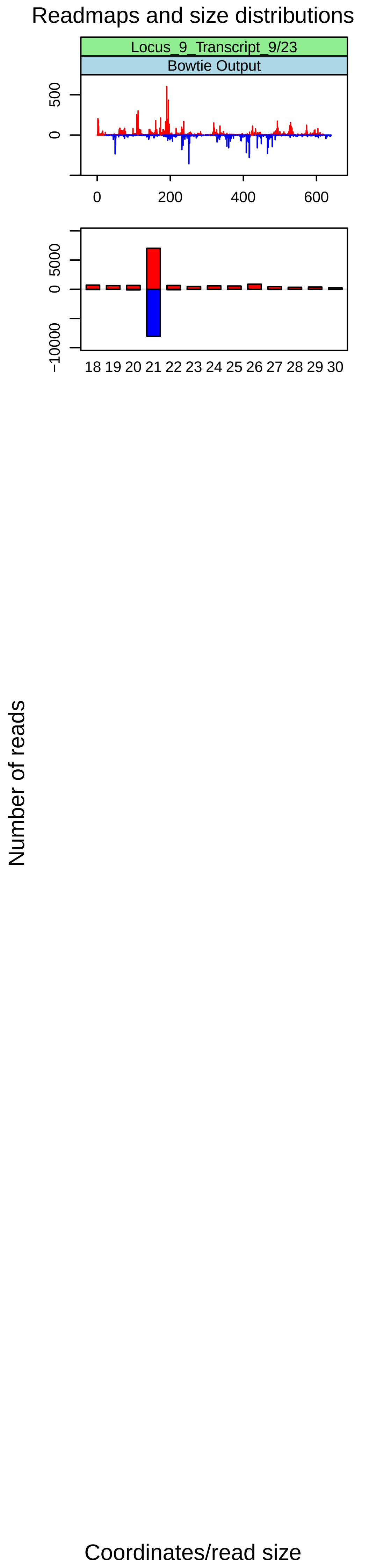

Readmaps and size distributions
Locus_9_Transcript_9/23
Bowtie Output
500
0
0
200
400
600
5000
0
−10000
18 19 20 21 22 23 24 25 26 27 28 29 30
Number of reads
Coordinates/read size
