# Supplementary material for: Metavisitor, a Suite of Galaxy Tools for Simple and Rapid Detection and Discovery of Viruses in Deep Sequence Data
Source: PLoS One. 2017 Jan 3;12(1):e0168397. doi: 10.1371/journal.pone.0168397 (PMC5207757; doi:10.1371/journal.pone.0168397)
Supplement: S1 Table — (PDF) [file pone.0168397.s038.pdf]

Supplementary Table S1

Galaxy tool names	Tool repository name	Tool wrapper developped by	Third party software (if applicable)	Purpose	Comments
Retrieve FASTA from NCBI	fetch_fasta_from_ncbi	The authors	NCBI E-utilities API	Retrieve nucleotide or protein sequences from the NCBI database using a string query	Download through http NCBI interface can take time
Clip adapter	yac_clipper	The authors		Removes adapter sequences from fastq sequence files	
Concatenate multiple datasets	concatenate_multiple_datasets	The authors		Merges multiple datasets tail-to-head	
FASTQ to FASTA	fastq_to_fasta	The Galaxy devteam	fastx_toolkit 0.0.13	Converts fastq sequences to fasta sequences	Required in some intances to feed de novo assembly tools
fasta - tabular	msp_fasta_tabular_converter	The authors		Performs various fasta to tabular conversions.	Used to reduce read datasets to a set of unique sequences
Normalize By Median	Pick Fasta sequences	The authors	khmer 2.0 [1]	Filter sequence reads using digital normalization via k-mer abundances	Used to reduce/normalize the abundance of sequence reads in sequence datasets
Regex Find And Replace	regex_find_replace	J. Johnson		Text replacement tool that help formating reports	
Pick Fasta sequences	cherry_pick_fasta	The authors		Filter a collection of fasta sequences and returns those that satisfy a query match in their name	
Filter sequences by length	fasta_filter_by_length	The Galaxy devteam		Outputs sequences between a minimal length and a maximum length	
Trim sequences	fastx_trimmer	The Galaxy devteam	fastx_toolkit 0.0.13		
sRbowtie	msp_sr_bowtie	The authors	bowtie 1.1.2, samtools 1.2	Aligns fasta sequence reads to indexed references.	Optimized for short reads
Bowtie2	bowtie2	The Galaxy devteam	bowtie2 2.2.6.2	Aligns fastq reads to reference genome	
Oases_optimiser	msp_oases	The authors	oases 0.2.08, velvet 1.2.10	De novo assembly of sequence reads	Performs iterative runs of velvet various kmer values and returns more significant contigs. Adapted to short read (<30 nt) assembly. Can also be run with longer reads. Memory consuming, can fail if the dataset is too large
Trinity	trinityrnaseq	A.J. Hemrom	trinityrnaseq r20140717, samtools 1.2	De novo assembly of sequence reads (>30 nt long)	Cannot be used for short reads (<30 nt)
spades	spades	Lionel Guy	spades 3.9.0	De novo assembly of sequence reads	An option for de novo assembly that can be used in Galaxy. Tested in Use Case 2-2: successfully identified the Anophele C virus genome sequence (Genebank Acc. Number KU169878)
cap3	msp_cap3	The authors	CAP3 [2] http://seq.cs.iastate.edu/CAP3/cap3.linux.x86_64.tar	De novo assembly of long DNA sequences	
NCBI BLAST+	ncbi_blast_plus	Cock et al. [3]	ncbi-blast-2.2.31+	Finds regions of similarity between nucleotide or protein sequences.	Compares sequences to sequence databases and calculates the statistical significance
Parse blast output and compile hits	msp_blastparser_and_hits	The authors		Parses blastn or tblastx tabular outputs and organizes hits to subjects, with various coverage informations<br>Return query hits sorted by their alignment to blast subjects, so that they can be picked for subsequent CAP3 assembly	
blast_to_scaffold	blast_to_scaffold	The authors		Integrates sequences of blastn or tblastx contig hits in a guide sequence taken as a scaffold	A reference sequence is required as a scaffold for integrated sequences
Generate readmap and histograms from alignment files	msp_sr_readmap_and_size_histograms	The authors	bowtie 1.0.0, pysam 0.9.0, numpy 1.9.3, r-optparse 1.3.0, r-latticeextra 0.6.26	Generates readmaps and size histograms from small RNA bowtie alignments	

1. Titus Brown C, Howe A, Zhang Q, Pyrkosz AB, Brom TH. A Reference-Free Algorithm for Computational Normalization of Shotgun Sequencing Data [Internet]. arXiv [q-bio.GN]. 2012. Available: http://arxiv.org/abs/1203.4802
2. Huang X, Madan A. CAP3: A DNA sequence assembly program. Genome Res. 1999;9: 868–877.
3. Cock PJA, Chilton JM, Grüning B, Johnson JE, Soranzo N. NCBI BLAST+ integrated into Galaxy. Gigascience. 2015;4: 39.
