# Supplementary material for: Metavisitor, a Suite of Galaxy Tools for Simple and Rapid Detection and Discovery of Viruses in Deep Sequence Data
Source: PLoS One. 2017 Jan 3;12(1):e0168397. doi: 10.1371/journal.pone.0168397 (PMC5207757; doi:10.1371/journal.pone.0168397)
Supplement: S2 Table — The times given correspond to execution of the workflows on a 16-core (2GHz) machine with 96 Mo RAM, Galaxy release 16.04. (PDF) [file pone.0168397.s039.pdf]

Supplementary Table S2

Workflow	execution time (h:mm:ss)
Use Case 1-1	0:12:14
Use Case 1-2	0:46:07
Use Case 1-3	0:48:23
Remapping in Use cases 1-1	0:08:35
Use Case 1-4	0:08:19
Use Case 2-1	1:49:24
Small RNA profiling of Oases contigs (Use Case 2-1)	0:29:55
Use Case 2-2	2:55:12
Trinity test in Use Case 2-2	0:50:05
Spades test in Use Case 2-2	0:40:28
Use Case 3-1	1:03:39
Use Case 3-2	0:50:43
Use Case 3-3 (Ebola)	0:04:41
Use Case 3-3 (Lassa L)	1:42:06
Use Case 3-3 (Lassa S)	1:41:31
