# Supplementary material for: Metavisitor, a Suite of Galaxy Tools for Simple and Rapid Detection and Discovery of Viruses in Deep Sequence Data
Source: PLoS One. 2017 Jan 3;12(1):e0168397. doi: 10.1371/journal.pone.0168397 (PMC5207757; doi:10.1371/journal.pone.0168397)
Supplement: S3 Table — Set of contigs (Loci) with clear (+), unclear (?) or no siRNA signature were manually selected from S10 Fig and tested for significant blastx alignment against the vir1 index and the Non-redundant NCBI protein database (october 201). (PDF) [file pone.0168397.s040.pdf]

Supplementary Table S3

Contigs (grouped by Oases locus)	siRNA signatur	Vir1 blastx hits (Metavisitor settings)	nr protein database hits using blastx (Word size "3" in "additional parameters)
Locus_1	+	cypovirus	cypovirus
Locus_2	+	cypovirus	cypovirus
Locus_4	+	cypovirus	cypovirus
Locus_5	+	cypovirus	cypovirus
Locus_7	+	cypovirus	cypovirus
Locus_9	+	cypovirus	cypovirus
Locus_38	+	cypovirus	cypovirus
Locus_53	+	cypovirus	cypovirus
Locus_17	+	AnCV	AnCV
Locus_3	+	-	-
Locus_46	+	-	-
Locus_68	?	-	WP_056074853.1 Chryseobacterium sp. (5e-49)
Locus_293	?	-	XP_317776.3 Anopheles Gambiae (2e-79)
Locus_29	-	-	KWW27340.1 Bacterium p20 (4e-44)
Locus_196	-	-	KZS01309.1 Daphnia magna (3e-38)
Locus_293	-	-	XP_317776.3 Anopheles gambiae (2e-79)
